# Quantifying the effects of the COVID-19 pandemic on gender equality on health, social, and economic indicators: a comprehensive review of data from March, 2020, to September, 2021

**DOI:** 10.1016/S0140-6736(22)00008-3

**Published:** 2022-06-25

**Authors:** Luisa S Flor, Joseph Friedman, Cory N Spencer, John Cagney, Alejandra Arrieta, Molly E Herbert, Caroline Stein, Erin C Mullany, Julia Hon, Vedavati Patwardhan, Ryan M Barber, James K Collins, Simon I Hay, Stephen S Lim, Rafael Lozano, Ali H Mokdad, Christopher J L Murray, Robert C Reiner, Reed J D Sorensen, Annie Haakenstad, David M Pigott, Emmanuela Gakidou

**Affiliations:** a Institute for Health Metrics and Evaluation, University of Washington, Seattle, WA, USA; b Department of Health Metrics Sciences, School of Medicine, University of Washington, Seattle, WA, USA

## Abstract

**Background:**

Gender is emerging as a significant factor in the social, economic, and health effects of COVID-19. However, most existing studies have focused on its direct impact on health. Here, we aimed to explore the indirect effects of COVID-19 on gender disparities globally.

**Methods:**

We reviewed publicly available datasets with information on indicators related to vaccine hesitancy and uptake, health care services, economic and work-related concerns, education, and safety at home and in the community. We used mixed effects regression, Gaussian process regression, and bootstrapping to synthesise all data sources. We accounted for uncertainty in the underlying data and modelling process. We then used mixed effects logistic regression to explore gender gaps globally and by region.

**Findings:**

Between March, 2020, and September, 2021, women were more likely to report employment loss (26·0% [95% uncertainty interval 23·8–28·8, by September, 2021) than men (20·4% [18·2–22·9], by September, 2021), as well as forgoing work to care for others (ratio of women to men: 1·8 by March, 2020, and 2·4 by September, 2021). Women and girls were 1·21 times (1·20–1·21) more likely than men and boys to report dropping out of school for reasons other than school closures. Women were also 1·23 (1·22–1·23) times more likely than men to report that gender-based violence had increased during the pandemic. By September 2021, women and men did not differ significantly in vaccine hesitancy or uptake.

**Interpretation:**

The most significant gender gaps identified in our study show intensified levels of pre-existing widespread inequalities between women and men during the COVID-19 pandemic. Political and social leaders should prioritise policies that enable and encourage women to participate in the labour force and continue their education, thereby equipping and enabling them with greater ability to overcome the barriers they face.

**Funding:**

The Bill & Melinda Gates Foundation.

## Introduction

The COVID-19 pandemic has affected women and men through direct and indirect health effects, and through shifts in key socioeconomic factors.^1^, ^2^, ^3^ To design effective interventions and policies to prevent the amplification of existing gender gaps across key domains of health and wellbeing, it is crucial to monitor the differential impacts of the pandemic across categories of sex (the biological categorisation of a person as male, female, or intersex) and gender (a relational concept that captures the socially and culturally constructed ideas of what it is to be male or female in an environment).^4^, ^5^

Gaps in key domains of health and wellbeing during the pandemic relating to sex and gender have not been adequately studied. Most studies that have explored gender gaps have focused on indicators that are directly related to COVID-19 (eg, COVID-19-attributable deaths, incidence, and cases) and have found higher death rates among men globally.^2^, ^3^, ^6^ This mortality advantage of women is still not well understood and many hypotheses to account for this difference have emerged, most of which implicate biological sex-specific characteristics.^7^, ^8^, ^9^

However, the overall toll of the pandemic is much greater than its direct impacts, and evidence suggests that the indirect effects are shaped by marginalisation and disadvantage, which are influenced by gender, and are health and non-health related.^10^, ^11^ These indirect impacts of the COVID-19 pandemic by gender have not been explored systematically or in detail across geographies. Health-related impacts of the pandemic have been reported in areas such as forgone use of care;^12^ increased disruptions in reproductive health services;^13^ increased violence against women and girls;^14^, ^15^, ^16^ as well as higher vaccine hesitancy,^17^ and higher vaccine uptake among women.^18^ As the COVID-19 pandemic has caused major disruptions in all aspects of life, its impacts on other sectors of wellbeing are expected to be profound and widespread, with some of the most significant negative consequences seen in employment and income loss, unpaid labour, education, food security, and personal safety.^11^, ^19^, ^20^, ^21^, ^22^, ^23^, ^24^, ^25^ Preliminary indications suggest that the economic downturn has affected women more severely than men, partly reversing progress towards gender equality that had been made in many countries.^19^ It is particularly important to monitor and intervene on indicators that show early adverse pandemic-related effects, as experience from previous financial crises suggest that the adverse effects on women tend to persist long after the initial disruption.^26^

**Research in context**

**Evidence before this study**

A substantial amount of research shows that the direct health effects of the COVID-19 pandemic have affected men more than women: COVID-19 incidence, hospitalisation, and death rates are higher among men than women across locations. Conversely, existing evidence indicates that the indirect effects of COVID-19 have affected women disproportionately. Cross-sectional studies in a small number of countries have reported health service disruptions, increased rates of maternal mortality and stillbirth, and an increase in the incidence of domestic violence in response to stay-at-home (lockdown) orders. The economic impacts of the COVID-19 pandemic have affected women more than men in some countries because they tend to be employed disproportionately in sectors that are harder-hit by COVID-19, such as the hospitality industry or the informal sector (eg, domestic workers). Before the rollout of vaccines, vaccine hesitancy was higher among women than men. This evidence base points to the potential for the pandemic to have lasting effects on gender disparities. Existing studies, however, tended to focus on high-income countries or only on a small number of low-income and middle-income countries. Few investigations have tracked how gender disparities have changed over the course of the pandemic. Given broad differences in gender norms, policy responses, and economic and social conditions around the world, the scope of existing studies limits knowledge about the implications of the pandemic for gender disparities globally.

**Added value of this study**

This study provides comparable estimates of gender disparities for a range of health, social, and economic indicators between March, 2020, and September, 2021, for 193 countries, spanning all geographical regions and income levels. We reviewed publicly available datasets that collected information during COVID-19 for all countries and present what is known about the impacts of the COVID-19 pandemic on gender equality in health and other core domains of wellbeing worldwide. Indicators for the following categories were analysed: (1) vaccine hesitancy and uptake, (2) health-care services, (3) economic and work-related concerns, (4) education, and (5) safety at home and in the community. This study provides the first comprehensive evidence on gender disparities for numerous countries and a wide range of health-related, social, and economic indicators throughout the pandemic.

**Implications of all the available evidence**

Despite the large amount of information collected during the pandemic, significant data gaps persist, particularly with regard to gender-disaggregated data. The available evidence indicates that the COVID-19 pandemic has tended to exacerbate previously existing social and economic disparities, rather than create novel inequities. The finding that women had higher levels of employment loss, of increases in chores, and of forgoing of work because of unpaid caretaking duties than men is a particular concern. Societies are at a pivotal moment in which investments in the empowerment of women and girls are needed to ensure that crucial progress towards gender equality does not get stalled or reversed because of the pandemic. The social and economic fallout from COVID-19 and its implications for the broader empowerment and equality of women should be prevented from making the world more unequal in the years to come.

In this study, we aimed to explore gender disparities in major health-related and non-health-related indicators at the regional and global level. We also aimed to comprehensively review and synthesise publicly-available datasets that describe the impact of the social and economic fallout of the COVID-19 pandemic on gender equality in health and other core domains of wellbeing worldwide.

## Methods

### Data sources

We searched for administrative and survey data that were disaggregated by sex or gender that described the effects of the COVID-19 pandemic on health and other domains of wellbeing. Sources were included when available for any period between Jan 1, 2020, and Sept 30, 2021. Specifically, we compiled data from 13 data sources with sex-specific or gender-specific data (appendix 1 p 51) and one additional gender-invariant source^27^ for vaccine uptake (appendix 2). Combined, these data sources provided information for 193 countries and contained indicators for at least one of the following priority areas:^28^ (1) vaccine hesitancy and uptake, (2) health-care services, (3) economic and work-related concerns, (4) education, and (5) safety at home and in the community. A description of data sources, including design, sampling strategy and coverage, are provided in the appendix (appendix 1 pp 6–8). As most of the survey data sources framed their questions in terms of gender (rather than sex; appendix 2) and our indicators were predominantly tied to gendered norms rather than biological aspects, we adopted the use of the term gender, and used the terms women and men throughout. Non-binary response options were usually not offered to respondents.

### Definitions and data processing

Definitions and data sources used for each of the indicators included in the analysis are shown in the table. We extracted relevant variables from individual-level records from each survey where possible, and otherwise used tabulations. We also mapped variables onto standardised definitions. For population-level outcomes extracted from administrative records, such as vaccine uptake rates, population estimates from the Global Burden of Diseases, Injuries, and Risk Factors Study (GBD) 2019^42^ were used as denominators to calculate rates. For vaccine uptake in particular, we used administrative data assembled by Our World In Data^27^ and the US Centers for Disease Control and Prevention.^43^ As officially-reported, gender-disaggregated data on vaccine uptake were not widely available, we used self-reported, location-gender-specific survey data to estimate the gender pattern applied to population-level administrative records (appendix 1 p 11).

**Table:** Definitions and data sources used for each of the indicators included in the analysis

	**Definition**	**Data sources**
**Vaccine hesitancy and uptake**		
Vaccine hesitancy	Proportion of individuals aged ≥18 years who refused vaccination, despite availability of vaccination services	University of Maryland Social Data Science Center Global COVID-19 Trends and Impact Survey (UMD Global CTIS);^29^ The Delphi Group at Carnegie Mellon University US COVID-19 Trends and Impact Survey (Delphi US CTIS);^30^ COVID-19 Behavior Tracker 2020 (YouGov)^31^
Fully vaccinated	Proportion of individuals that had received all doses prescribed by the vaccination protocol	University of Maryland Social Data Science Center Global COVID-19 Trends and Impact Survey (UMD Global CTIS);^29^ The Delphi Group at Carnegie Mellon University US COVID-19 Trends and Impact Survey (Delphi US CTIS);^30^ COVID-19 Behavior Tracker 2020 (YouGov);^31^ Centers for Disease Control and Prevention;^32^ Global Health 50/50;^33^ Our World in Data,*^27^ COVerAGE-DB^34^
**Health care**		
Any disruption in health care	Proportion of individuals who had any disruption in health care because of the COVID-19 pandemic	University of Maryland Social Data Science Center Global COVID-19 Trends and Impact Survey (UMD Global CTIS);^29^ COVID-19 Health Services Disruption Survey 2020;^35^ COVID-19 Health Services Disruption Survey 2021;^36^ Survey on Gender Equality at Home;^37^ COVID-19 Rapid Gender Assessment Survey^38^
Disruption in reproductive health	Proportion of individuals who had a disruption in sexual or reproductive health care (ie, contraception, testing, and treatment for sexually transmitted diseases and HIV, treatment infertility, care for survivors of gender-based violence, and care related to pregnancy) because of the COVID-19 pandemic among those who reported need for sexual or reproductive health care	COVID-19 Rapid Gender Assessment Survey^38^
Disruption in preventative care	Proportion of individuals who had a preventative health care (ie, immunisation, vaccination, family planning, prenatal care, postnatal care, routine check-up services) disruption because of the COVID-19 pandemic	University of Maryland Social Data Science Center Global COVID-19 Trends and Impact Survey (UMD Global CTIS)^29^
Disruption in medication access	Proportion of individuals that had a disruption in access to medication because of the COVID-19 pandemic	University of Maryland Social Data Science Center Global COVID-19 Trends and Impact Survey (UMD Global CTIS);^29^ COVID-19 Health Services Disruption Survey 2020;^35^ COVID-19 Health Services Disruption Survey 2021;^36^ Measuring COVID-19 Impacts, Mitigation and Awareness Survey (FINMRK)^39^
Disruption in health products access	Proportion of individuals who had a disruption in access to health products (eg, eyeglasses, hearing aid, and crutches) because of the COVID-19 pandemic	University of Maryland Social Data Science Center Global COVID-19 Trends and Impact Survey (UMD Global CTIS);^29^ COVID-19 Health Services Disruption Survey 2021;^36^ Survey on Gender Equality at Home;^37^ COVID-19 Rapid Gender Assessment Survey^38^
**Economic and work-related concerns**		
Employment loss	Proportion of individuals who worked before the pandemic and who are not currently working	University of Maryland Social Data Science Center Global COVID-19 Trends and Impact Survey (UMD Global CTIS);^29^ COVID-19 Health Services Disruption Survey 2020;^35^ COVID-19 Health Services Disruption Survey 2021;^36^ COVID-19 Behavior Tracker 2020 (YouGov);^31^ COVID-19 High Frequency Phone Survey;^40^ Survey on Gender Equality at Home^37^
Income loss	Proportion of individuals currently working who had loss of income since the COVID-19 pandemic	COVID-19 Rapid Gender Assessment Survey;^38^ COVID-19 Behavior Tracker 2020 (YouGov);^31^ Research for Effective Covid-19 Response Panel Survey 2020;^41^ COVID-19 Health Services Disruption Survey 2020^35^
Increase in chores	Proportion of individuals who are spending more time in different household chore activities since the COVID-19 pandemic	COVID-19 Rapid Gender Assessment Survey;^38^ Survey on Gender Equality at Home^37^
Increase in care for others	Proportion of individuals that are spending more time in different care activities since the COVID-19 pandemic	COVID-19 Rapid Gender Assessment Survey;^38^ Survey on Gender Equality at Home^37^
Not working to care for others	Proportion of individuals that left their job after the COVID-19 pandemic to care for someone out of those not currently working	University of Maryland Social Data Science Center Global COVID-19 Trends and Impact Survey (UMD Global CTIS);^29^ COVID-19 Health Services Disruption Survey 2020;^35^ COVID-19 Health Services Disruption Survey 2021^36^
**Education**		
School dropout	Proportion of learners (individuals previously enrolled in any level of school) no longer in school, not because of graduation or school break, among all learners in school before the COVID-19 pandemic	COVID-19 Health Services Disruption Survey 2021^36^
Adequate remote learning	Proportion of learners (individuals currently enrolled in any level of school) with good internet access among all learners learning remotely during the COVID-19 pandemic	COVID-19 Health Services Disruption Survey 2021^36^
**Safety at home and in the community**		
Perception of gender-based violence increase	Proportion of individuals who reported that they perceived that household or partner violence had increased in their community since the start of the COVID-19 pandemic	COVID-19 Health Services Disruption Survey 2021;^36^ COVID-19 Rapid Gender Assessment Survey^38^
Feeling unsafe at home	Proportion of individuals that reported feeling unsafe at home during the COVID-19 pandemic	Survey on Gender Equality at Home;^37^ COVID-19 Rapid Gender Assessment Survey^38^

* Data not disaggregated by sex or gender.

#### Data synthesis

Given that multiple data sources were available for most indicators, and often overlapped in terms of geography and time periods covered, we applied data synthesis methods to produce estimates by gender, country, and month. We took distinct modelling approaches for time-variant and time-invariant indicators.

For eight indicators with data available at multiple timepoints, we used a time series modelling approach (appendix 1 p 47). Briefly, we used mixed effects and Gaussian process regression to synthesise all available data sources, and estimate complete time series, accounting for uncertainty in the input data and the modelling process. We represented model outputs with 1000 iterations (draws) of the posterior distribution to propagate uncertainty through all final estimated quantities. We calculated absolute gaps and ratios between women and men for all indicators at the draw level. Mean and 95% uncertainty intervals (UIs) were calculated across draws, and summarised by month and geography.

For the other eight time-invariant indicators, we used bootstrapping data synthesis techniques. We calculated logit transformed, country-gender-indicator-specific proportions, and aggregated at the draw-level using country-gender-specific population weights to create point estimate proportions and 95% UIs at the regional and total levels (appendix 1 p 48).

#### Multivariate regression analyses

We used mixed effects logistic regression models to explore associations between each indicator and the gender of the respondent, adjusting for geography, age, educational attainment, and urbanicity (when reported; appendix 1 pp 49–50). For educational outcomes, a fixed effect for the gender of the learner was included. We ran separate regressions by data source, geography, and time period to explore the sensitivity of our findings to these dimensions (appendix 1 pp 69–70). Findings from the most recent time period are reported in the Results section.

Analyses were done in R. This study complies with the Guidelines for Accurate and Transparent Health Estimates Reporting (GATHER)^44^ recommendations (appendix 1 p 5).

### Role of the funding source

The funder of the study had no role in the study design, data collection, data analysis, data interpretation, or the writing of the report.

## Results

Overall, publicly available gender-disaggregated data are still incomplete for multiple aspects of health and wellbeing. Although we used data from 193 countries across sources, data availability varied widely by region and indicator. Geographic coverage was highest in north Africa and the Middle East, and south Asia, with the greatest data gaps seen in southeast Asia, east Asia, and Oceania (appendix 1 pp 54–61). The availability of data was greatest for indicators related to the direct impacts of the COVID-19 pandemic, as well as those related to economic and work-related impacts. The largest data gaps were found for sexual and reproductive health care, disruptions in schooling, safety, and gender-based violence.

**Figure 1:**
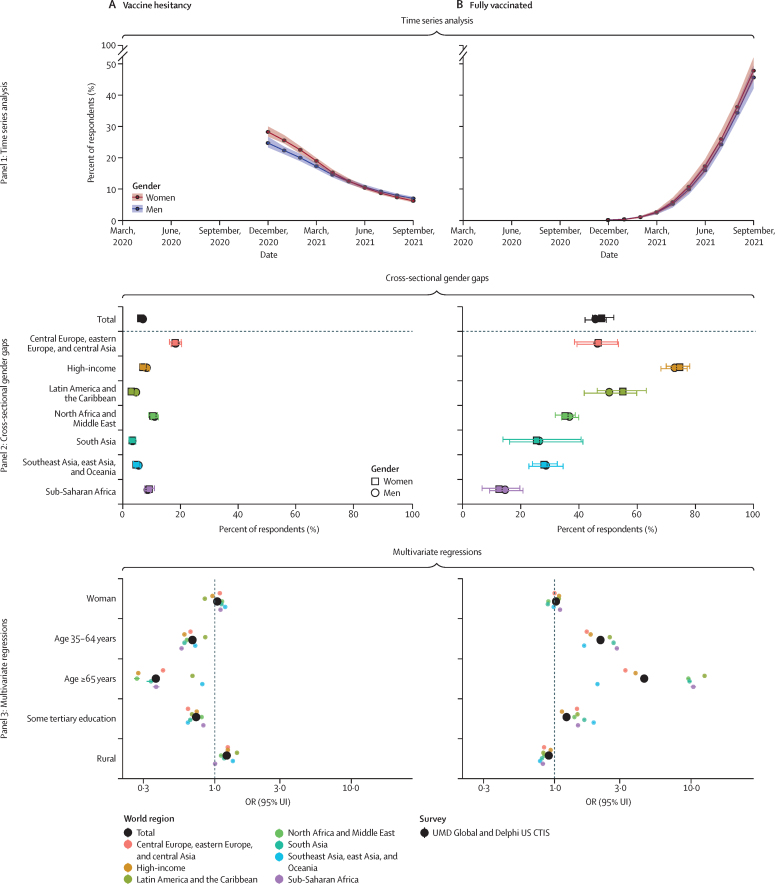
Time-series, cross-sectional, and multivariate logistic regression analyses for vaccination hesitancy and uptake indicators For vaccine hesitancy and uptake, input data were available for multiple time periods. Panel 1 (time-series analysis) shows the average estimated time trend across regions, with 95% prediction intervals. Panel 2 (cross-sectional gender gaps) shows cross-sectional estimates for indicators in September, 2021, summarised by gender and world region. Gender is indicated by point shape, and 95% uncertainty intervals (UIs) for each estimate are shown. Panel 3 (multivariate regressions) presents odds ratios (OR) and 95% UIs from mixed effects logistic regression models exploring the association between each indicator and gender, adjusting for geography, age, educational attainment, and urbanicity. We ran separate regressions for each data source that was available for each indicator to explore the sensitivity of our findings to the data source used. When possible, we additionally ran region-specific models to assess geographic variation in findings. Region is indicated by colour and data source is indicated by shape of the point. For each regression model covariate, the reference categories are listed in parentheses: woman (man); age 35–64 years (age <35 years); age ≥65 years (age <35); some tertiary education (less than tertiary education); and rural (urban). Delphi US CTIS=The Delphi Group at Carnegie Mellon University US COVID-19 Trends and Impact Survey, in partnership with Facebook. UMD Global CTIS=The University of Maryland Social Data Science Center Global COVID-19 Trends and Impact Survey, in partnership with Facebook.

In January, 2021, women reported significantly higher vaccine hesitancy (25·6% [95% UI 24·6–27·2]) compared with men (22·3% [21·1–23·5]). The gender gap decreased over time, and as of September, 2021, had largely closed at the global level (figure 1, panel 1A). Vaccine hesitancy was the highest in central Europe, eastern Europe, and central Asia, with 18·2% (16·2–20·3) of women and 18·3% (16·8–20·4) of men still hesitant as of September 2021 (figure 1, Part 2A). Controlling for country, age, education level, and urbanicity, we found a significant, albeit small-magnitude, difference in vaccine hesitancy between women and men in all regions from April to September, 2021 (figure 1, panel 3A). Women reported being less vaccine hesitant than men in Latin America and the Caribbean (odds ratio [OR] 0·85 [0·84–0·85]) and in high-income countries (0·96 [0·96–0·97]), while higher odds were observed for women in the other regions (appendix 1 p 71). Compared with individuals who were younger than 35 years, vaccine hesitancy was significantly lower among those aged 35–64 years (0·69 [0·68–0·69]) and those aged 65 years and older (0·37 [0·37–0·37]). Significantly lower rates of hesitancy were also seen among those with tertiary education (0·73 [0·73–0·73]). Individuals living in rural settings were more likely than those in urban settings to report vaccine hesitancy globally (1·22 [1·22–1·23]) and in most world regions except in sub-Saharan Africa (1·01 [0·98–1·04]; figure 1, panel 3A).

Vaccination rates have continued to increase for women and men globally (figure 1, panel 1B). By September 2021, 46·7% (95% UI 43·7–50·4) of adults were fully vaccinated, with the highest rates of vaccination seen in high-income countries and the lowest rates in sub-Saharan Africa (figure 1, panel 2B). Statistically significant gender differences in vaccine uptake were not seen in any region in September 2021 (appendix 1 p 62). However, multivariate regression results suggest that some significant, albeit small-magnitude, variation across regions does exist, with women having significantly higher odds than men of being fully vaccinated in Latin America and the Caribbean (OR 1·08 [1·07–1·08]), high-income countries (1·08 [1·08–1·08]), and sub−Saharan Africa (1·10 [1·07–1·13]). Conversely, women were less likely than men to be fully vaccinated in south Asia (0·89 [0·88–0·91]) and in north Africa and the Middle East (0·90 [0·89–0·91]; figure 1, panel 3B; appendix 1 p 71). In all regions, respondents who were older, had tertiary levels of education, and who lived in urban settings were significantly more likely to be fully vaccinated (figure 1, panel 3B).

**Figure 2:**
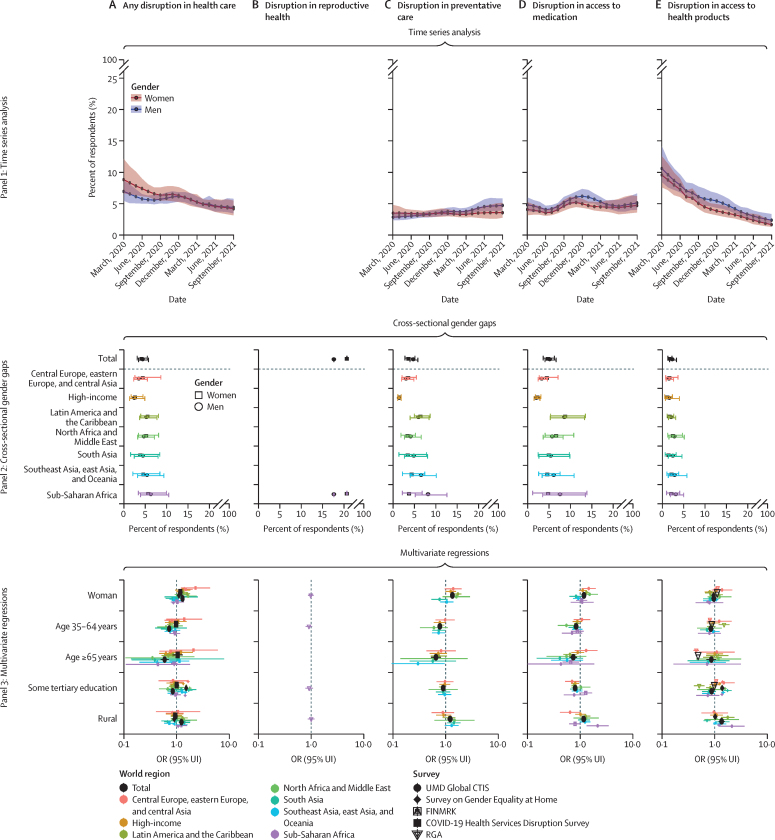
Time-series, cross-sectional, and multivariate logistic regression analyses for health-care access indicators For any disruption in health care, preventative care, access to medication, and access to health products, input data were available for multiple time periods. Panel 1 (time-series analysis) shows the average estimated time trend across regions for these indicators, with 95% prediction intervals. Panel 2 (cross-sectional gender gaps) shows cross-sectional estimates for these indicators in September, 2021, summarised by gender and world region. For disruption in reproductive health, input data were only available cross-sectionally, and results are summarised by gender and world region in panel 2. Gender is indicated by point shape, and 95% uncertainty intervals (UIs) for each estimate are shown. For all health-care access indicators, panel 3 (multivariate regressions) presents odds ratios (OR) and 95% UIs from mixed effects logistic regression models exploring the association between each indicator and gender, adjusting for geography, age, educational attainment, and urbanicity. We ran separate regressions for each data source that was available for each indicator to explore the sensitivity of our findings to the data source used. We additionally ran region-specific models to assess geographic variation in findings. Region is indicated by colour and data source is indicated by shape of the point. For each regression model covariate, the reference categories are listed in parentheses: woman (man); age 35–64 (age <35 years); age ≥65 years (age <35 years); some tertiary education (less than tertiary education); and rural (urban). Because of differences in how age was recorded by source, for FINMRK, COVID-19 Health Services Disruption Survey, and COVID-19 Rapid Gender Assessment Survey (RGA), the age covariates listed as ages 35–64 years represent age group 25–44 years and the age covariates listed as age ≥65 years represent age group ≥45 years (reference category: age<25 years). Disruption in reproductive health was only investigated among reproductive age categories (up to age 45 years). Age information was not available from the Survey on Gender Equality at Home. FINMRK=Measuring COVID-19 Impacts, Mitigation and Awareness Survey. UMD Global CTIS=The University of Maryland Social Data Science Center Global COVID-19 Trends and Impact Survey, in partnership with Facebook. RGA=COVID-19 Rapid Gender Assessment survey.

Self-reported rates of health-care disruption due to the COVID-19 pandemic have decreased over time and were gender-invariant (figure 2, panel 1A). In September 2021, 4·3% (95% UI 3·5–5·1) of respondents reported experiencing any disruption in health-care services due to the COVID-19 pandemic (figure 2, panel 2A), with no significant gender variation in any region (figure 2, panel 2A; appendix 1 p 62). In multivariable regression analyses, women were significantly more likely than men to report disruptions in health care overall, a finding mostly consistent across data sources (figure 2, panel 3A). Gender was associated with increased disruptions in all regions except in south Asia, southeast Asia, east Asia, and Oceania, and sub-Saharan Africa (appendix 1 pp 71–72).

Data for disruptions in sexual and reproductive health care were sparse. In sub-Saharan Africa, women reported sexual and reproductive health-care disruptions at significantly higher levels (20·6% [95% UI 20·6–20·7]) than men (17·6% [17·6–17·6]; figure 2, panel 2B). After adjusting for other sociodemographic characteristics, this difference was no longer statistically significant (OR 0·99 [0·87–1·11]; figure 2, panel 3B). Alternative models showed that women who were located in urban, non-capital cities and who were younger than 25 years were more likely to report disruptions in sexual and reproductive health care (appendix 1 p 53).

Reported levels of disruptions in preventative care and access to medication remained stable and relatively low over time (figure 2, panels 1C, D). In September 2021, 4·1% (95% UI 3·5–5·1) of respondents reported needing to delay or skip preventative health-care visits due to the COVID-19 pandemic, and 4·9% (4·1–5·7) of respondents reported difficulty accessing medications, with the lowest levels of both preventative healthcare disruptions and difficulty accessing medications seen in high-income countries (figure 2, panel 2C, D). Although significant gender gaps related to preventative care were seen in most regions earlier in the pandemic, these gaps had closed, except in sub-Saharan Africa, where disruptions disproportionately affected men in September 2021 (appendix 1 p 62). No recent significant gender gaps were observed in any world region for medication access. Multivariate regressions showed that women had significantly higher odds of reporting disruptions in preventative care and barriers to medications in Latin America and the Caribbean than men. In central Europe, eastern Europe, and central Asia and in north Africa and Middle East, women were also more likely to report issues in accessing medications than men due to the COVID-19 pandemic. Additionally, individuals residing in urban settings who were older and who had higher levels of education were less likely to report these health barriers (figure 2, panel 3C, D).

In September 2021, only 2·0% (95% UI 1·6–2·6) of respondents reported difficulty accessing health products, such as eyeglasses, hearing aids, or crutches because of the COVID-19 pandemic (figure 2, panel 1E, 2E). Although cross-sectional results for September 2021 indicated no significant gender gap globally (appendix 1 p 62), multivariate regression results were inconsistent across sources and regions (figure 2, panel 3E). The COVID-19 Rapid Gender Assessment Survey showed that women were significantly more likely than men to report disruptions in access to health products at the total level (1·11 [1·05–1·19]) due to the COVID-19 pandemic, while the Survey on Gender Equality at Home showed the opposite trend at the same geographic level (0·95 [0·93–0·97]), and data from the University of Maryland Social Data Science Center Global COVID-19 Trends and Impact Survey (UMD Global CTIS), in partnership with Facebook, suggested no significant difference. Across sources, respondents from rural areas in sub-Saharan Africa were more likely than those living in urban settings to report a barrier to accessing health products (figure 2, panel 3E).

**Figure 3:**
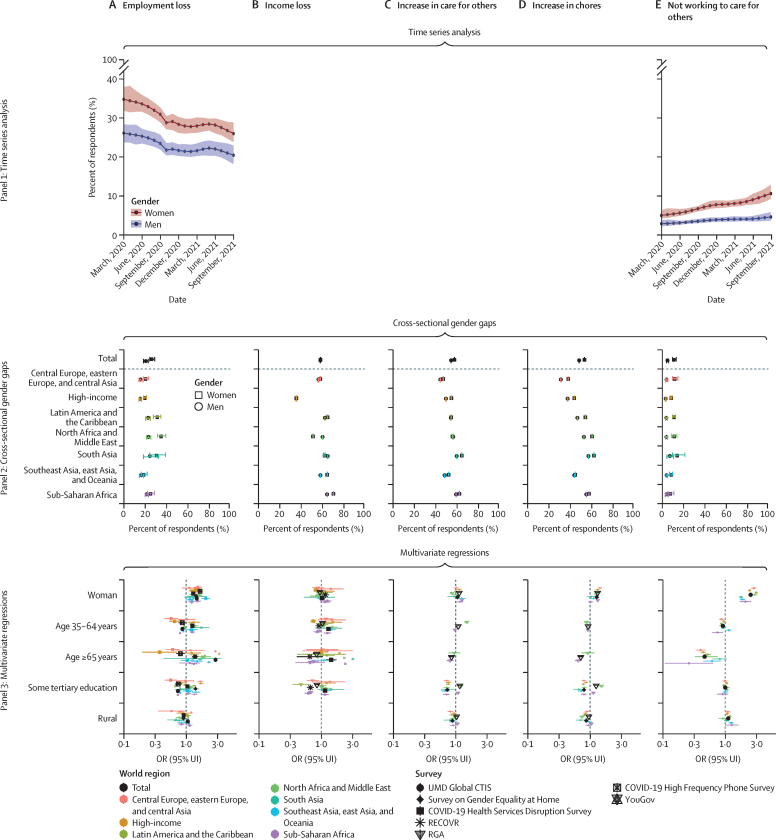
Time-series, cross-sectional, and multivariate logistic regression analyses for economic and work-related concerns indicators For employment loss indicator and not working to care for others indicator, input data were available for multiple time periods. Panel 1 (time-series analysis) shows the average estimated time trend across regions for these indicators, with 95% prediction intervals. Panel 2 (cross-sectional gender gaps) shows cross-sectional estimates for these indicators in September, 2021, summarised by gender and world region. For income loss, increase in care for others, and increase in chores, input data were only available cross-sectionally, and results are summarised by gender and world region in panel 2. Gender is indicated by point shape, and 95% uncertainty intervals (UIs) for each estimate are shown. For all economic and work-related concerns indicators, panel 3 (multivariate regressions) presents odds ratios (OR) and 95% uncertainty intervals from mixed effects logistic regression models exploring the association between each indicator and gender, adjusting for geography, age, educational attainment, and urbanicity. We ran separate regressions for each data source available for each indicator to explore the sensitivity of our findings to the data source used. When possible, we additionally ran region-specific models to assess geographic variation in findings. Region is indicated by colour and data source is indicated by shape of the point. For each regression model covariate, the reference categories are listed in parentheses: woman (man); age 35–64 years (age <35 years); age ≥65 years (age <35); some tertiary education (less than tertiary education); and rural (urban). Because of differences in how age was recorded by source, for the Measuring COVID-19 Impacts, Mitigation and Awareness Survey (FINMRK), COVID-19 Health Services Disruption Survey, and COVID-19 Rapid Gender Assessment survey (RGA), the age covariates listed as age 35–64 years represent age group 25–44 years and the age covariates listed as age ≥65 years represent age group ≥45 years (reference category: age <25 years). Age information was not available from the Survey on Gender Equality at Home. Delphi US CTIS=The Delphi Group at Carnegie Mellon University US COVID-19 Trends and Impact Survey, in partnership with Facebook. FINMRK=Measuring COVID-19 Impacts, Mitigation and Awareness Survey. UMD Global CTIS=The University of Maryland Social Data Science Center Global COVID-19 Trends and Impact Survey, in partnership with Facebook. RECOVR=Research for Effective Covid-19 Response Panel Survey. RGA=COVID-19 Rapid Gender Assessment survey.

Since the beginning of the pandemic, employment loss rates have been high, and significantly higher among women than men, with a steadily decreasing trend (figure 3, panel 1A). In September 2021, 26·0% (95% UI 23·8–28·8) of women and 20·4% (18·2–22·9) of men reported employment loss during the pandemic. In all regions, women report higher rates of employment loss than men (figure 3, panel 2A), with the largest gender gaps seen in north Africa and the Middle East (ratio of women to men: 1·52), and Latin America and the Caribbean (ratio of women to men: 1·38; appendix 1 p 62). Gender gaps remained statistically significant in multivariate regressions, although the magnitude varied by region. Results related to other predictors were less consistent across sources and geographies (figure 3, panel 3A).

Income loss was also very prevalent, reported by 58·4% (95% UI 58·3–58·5) of respondents, with the highest rates seen in sub-Saharan Africa and the lowest rates seen in high-income countries (figure 3, panel 2B). Although the overall rates were very similar between women and men, the gender gaps in income loss varied across regions. In descriptive results, women reported substantially higher rates of loss of income than men in southeast Asia, east Asia, and Oceania (ratio of women to men: 1·11) and sub-Saharan Africa (ratio of women to men: 1·09; appendix 1 p 62). Results from multivariate regressions showed that, after controlling for demographic characteristics and region, women were as likely as men to report income loss in nearly all sources and regions (figure 3, panel 3B; appendix 1 pp 71–72).

Over half of respondents also reported increases in chores and in caring for others during the COVID-19 pandemic, with women significantly more likely to report such increases than men in all regions, except in north Africa and the Middle East (figure 3, panel 2C, D). The largest gender gap was observed in high-income countries for increases in care for others (ratio of women to men: 1·10), and in central Europe, eastern Europe, and central Asia for increases in chores (ratio of women to men: 1·22). Throughout the course of the COVID-19 pandemic, women in every region were more likely than men to report forgoing work to care for other individuals, and this gender gap widened over time (figure 3, panel 1E). In March 2020, at the global level, women were 1·8 times more likely than men to report forgoing work to care for others, and this ratio increased to 2·4 by September 2021. Women presented higher rates of forgoing work to care for others than men in September, 2021, in 77 out of the 107 countries where data were available. Gender results remained significant for all three indicators in the multivariate regression analysis (figure 3, panels 3C–E).

**Figure 4:**
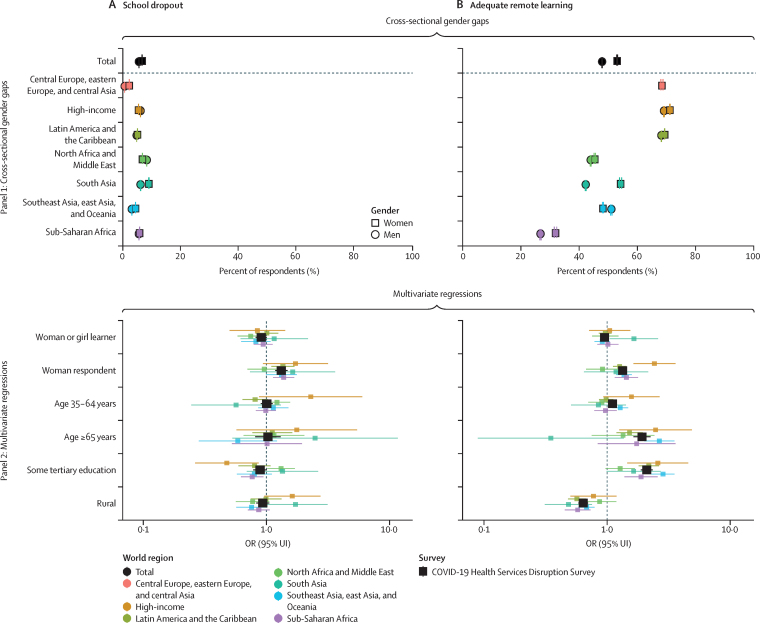
Cross-sectional and multivariate logistic regression analyses for education indicators For school dropout and adequate remote learning, input data were available cross-sectionally and are summarised by gender and world region in panel 1 (cross-sectional gender gaps). Gender is indicated by point shape, and 95% uncertainty intervals (UIs) for each estimate are shown. Panel 2 (multivariate regressions) presents odds ratios (OR) and 95% UIs from mixed effects logistic regression models exploring the association between each indicator and gender of the learner, adjusting for gender of respondent, geography, age, educational attainment, and urbanicity. We additionally ran region-specific models to assess geographic variation in findings. Region is indicated by colour and data source is indicated by shape of the point. For each regression model covariate, the reference categories are listed in parentheses: woman or girl learner (man or boy learner); woman respondent (man respondent); age 35–64 years (age <35 years); aged ≥65 years (age <35 years); some tertiary education (less than tertiary education); and rural (urban).

Data for the impacts of the COVID-19 pandemic on schooling have only been collected by one multinational source. Overall, respondents (typically a parent) reported that 6·0% (95% UI 5·98–6·02) of learners dropped out of school during the COVID-19 pandemic. Women and girl learners were 1·21 times (1·20–1·21) more likely than men and boys to reportedly drop out of school for reasons other than school closures (figure 4, panel 1A). The largest gender gaps for this indicator were seen in central Europe, eastern Europe, central Asia (ratio of women to men: 4·10), and south Asia (ratio of women to men: 1·48). In multivariate regression analyses, after controlling for other social, demographic, and geographic factors, the gender of the learner was no longer significantly associated with increased school dropout rates. Globally, women were more likely than men to report that their children had dropped out of school (1·33 [1·18–1·49]), particularly in Latin America and the Caribbean (1·37 [1·10–1·70]), and in sub-Saharan Africa (1·39 [1·13–1·69]). Compared with individuals with fewer years of schooling, those with more than 12 years of schooling in high-income countries (0·48 [0·26–0·87]) and in sub-Saharan Africa (0·77 [0·62–0·95]) were less likely to report that their children had dropped out of school (figure 4, panel 2A).

Among individuals learning online, only 50·4% (95% UI 50·3–50·5) of learners were reported to have adequate access to online learning technologies. Women and girl learners were 1·11 times (1·10–1·11) more likely than men and boy learners to be reported to have adequate access to online learning, with the largest gap seen in south Asia (ratio of women to men: 1·28). However, in central Europe, eastern Europe, and central Asia, and in southeast Asia, east Asia, and Oceania, an opposite trend was observed, with men and boy learners being more likely to be reported to have adequate access to online learning (figure 4, panel 1B). Globally, respondents who were women, respondents who had high levels of education, and respondents who lived in urban settings were also more likely to report that learners in their household had adequate access to online learning (figure 4, panel 2B).

**Figure 5:**
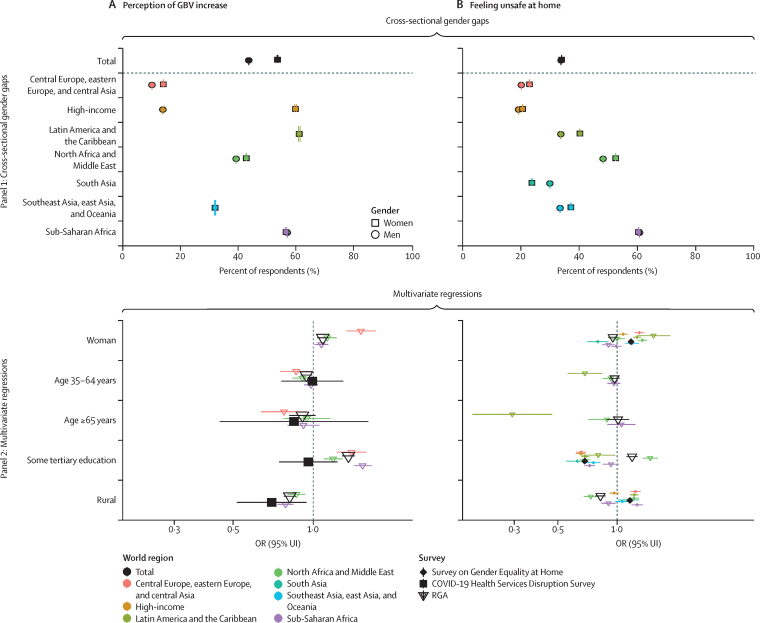
Cross-sectional and multivariate logistic regression analyses for safety at home and in the community indicators For perception of gender-based violence (GBV) increase and feeling unsafe at home, input data were available cross-sectionally and are summarised by gender and world region in panel 1 (cross-sectional gender gaps). Gender is indicated by point shape, and 95% uncertainty intervals (UIs) for each estimate are shown. Panel 2 (multivariate regressions) presents odds ratios (OR) and 95% UIs from mixed effects logistic regression models exploring the association between each indicator and gender, adjusting for geography, age, educational attainment, and urbanicity. When possible, we additionally ran region-specific models to assess geographic variation in findings. The geography of the finding is indicated by colour and the data source is indicated by the shape of the point. For each regression model covariate, the reference categories are listed in parentheses: woman (man); age 35–64 years (age <35 years); age ≥65 years (age <35 years); some tertiary education (less than tertiary education); and rural (urban). Because of differences in how age was recorded by source, for COVID-19 Health Services Disruption Survey, the age covariates listed as age 35–64 years represent age group 25–44 years and the age covariates listed as age ≥65 years represent age group ≥45 years (reference category: age <25 years). Age information was not available from the Survey on Gender Equality at Home. RGA=COVID-19 Rapid Gender Assessment survey.

Overall, 53·7% (95% UI 53·6–53·8) of women and 43·7% (43·7–43·8) of men reported that they perceived that gender-based violence had increased in their community during the COVID-19 pandemic (figure 5, panel 1A). The highest rates of perceived gender-based violence increase were reported by women in Latin America and the Caribbean (61·2% [60·9–61·5]), in high-income countries (59·9% [59·6–60·3]), and in sub-Saharan Africa (56·7% [56·4–56·9]). In multivariate regression analyses, women had higher odds (OR 1·08 [1·03–1·14]), while respondents living in rural settings had lower odds of reporting perceived increases in gender-based violence in both sources of data (figure 5, panel 2A).

Women (33·9% [95% UI 33·9–33·9]) and men (33·7% [33·7–33·7]) were equally likely to report feeling unsafe at home. However, at the regional level, significant gender gaps were observed, with women reporting higher rates than men in Latin America and the Caribbean (ratio of women to men: 1·20), central Europe, eastern Europe, and central Asia (ratio of women to men: 1·14), and southeast Asia, east Asia, and Oceania (ratio of women to men: 1·11), and men reporting higher rates than women in south Asia (figure 5, panel 1B). In multivariate regression, results varied by source and region (figure 5, panel 2B) with data from the Survey on Gender Equality at Home suggesting that women were more likely than men to feel unsafe at home, and data from the COVID-19 Rapid Gender Assessment Surveys showing higher odds for men.

The impact of the COVID-19 pandemic has varied greatly across regions. Results faceted by region are shown in the appendix (appendix 1 pp 73–74). Sub-Saharan Africa stood out as the region with the most pronounced differences compared with global totals, showing that this region was disproportionately impacted by the COVID-19 pandemic. Specifically, sub-Saharan Africa had the lowest rates of vaccine uptake and least access to adequate online learning, alongside having the highest rates of disruption in health care and income loss, and high rates of employment loss, increases in care for others, and perception of increases in gender-based violence. Rates of income loss among women in sub-Saharan Africa were significantly higher compared with men and the global average.

By contrast, in the high-income region, we observed the smallest deleterious effects of COVID-19 for most indicators, and highest rates of vaccine access. Perceptions of increased gender-based violence was a notable exception, as women in high-income countries reported significantly higher rates of perceived gender-based violence than the overall average.

In central Europe, eastern Europe, and central Asia, we observed the largest share of participants who reported adequate remote learning resources and the lowest rates of learners dropping out of school. These regions nevertheless presented the highest rate of vaccine hesitancy and women were more likely to report hesitancy than men. However, increases in chores and caring for others were smaller than rates observed in other regions.

In Latin America and the Caribbean, women were more likely to report disruptions in health care than men. Employment loss, income loss, feeling unsafe at home, and perceived increase in gender-based violence were also highly prevalent in these regions, with the feeling unsafe indicator showing significantly higher rates among women.

In north Africa and the Middle East, we observed lower than average rates of vaccine uptake, the second poorest access to adequate remote learning, and the highest rates of school dropout. In south Asia, rates of increases in chores and in caring for others, as well as forgoing work to care for others as a result of the pandemic, were the highest among all regions, while vaccination rates, vaccine hesitancy, and feeling unsafe at home were lower than the overall average. Southeast Asia, east Asia, and Oceania presented the third lowest vaccine uptake rates, and a higher than average disruption to any health care. Although the region showed comparatively lower rates of increases in chores and in caring for others, the gender gap in these indicators remained with women reporting higher rates than men.

## Discussion

The impact of the COVID-19 pandemic on health-related indicators and non-health-related indicators, and the resulting implications for gender disparities, has varied throughout the pandemic. Here, we show evidence of gender disparities in health, social, and economic aspects, with women being disproportionately affected across several dimensions. An encouraging finding is that, throughout the course of the pandemic, many of the initially observed gender gaps have decreased considerably. Across indicators, the greatest and most-persistent gender disparities were observed in workforce participation and uncompensated labour. Furthermore, although data for learning loss are sparse, our results highlight the worrying risk that increased school dropout and poor access to remote learning technologies will exacerbate existing learning gaps. The indirect impacts of the COVID-19 pandemic have also been experienced differentially across geographic regions, with the largest disruptions in sub-Saharan Africa and Latin America and Caribbean, and the smallest impacts among high-income countries.

Despite several large-scale global data collection efforts launched by UN agencies and the private sector, data available for comprehensive analyses of pandemic-related gender disparities remain extremely scarce across multiple domains of wellbeing, particularly for exploring factors that intersect with gender to amplify gender gaps. Data collection during the pandemic mainly occurred through dedicated apps and online survey platforms, which were exclusively available to individuals who owned smartphones and could read. Although smartphone ownership and mobile internet use are increasing across the world, significant gender, economic, and geographical inequalities persist,^45^ which has implications for our analysis and other analyses of pandemic-related inequalities; those in greater need of policy interventions, especially women, are significantly under-represented in analyses of available information.^45^, ^46^

Despite data limitations, the results presented here are noteworthy and demand action, as the impact of the COVID-19 pandemic across different sectors and regions has been pervasive. This study clarifies areas in which the pandemic has amplified gender gaps, and also areas in which gender disparities are small. Notably, gender equality has been achieved in vaccine uptake globally. Despite large inequities across regions and countries,^47^, ^48^ vaccination efforts have been largely carried out without gender gaps. Previous work has indicated that men are more inclined to accept COVID-19 vaccines,^17^ as women were particularly worried about side-effects and safety, raising concerns about uptake. Yet, in our study, we found no gender inequality in vaccine uptake, representing a significant achievement for a new and rapidly implemented intervention. Nevertheless, global uneven vaccine distribution has the potential to slow uptake among women and expand gender gaps, especially in low-income countries, if further action is not taken. Delays in getting COVID-19 vaccines to low-income countries provide an opportunity for misinformation to flourish, raising hesitancy, especially among women of childbearing age who are concerned about pregnancy and fertility,^49^ despite the absence of supporting evidence for such a risk.^50^, ^51^ Thus, national governments and international agencies need to continue to monitor differential uptake of vaccines, especially among economically disadvantaged populations and vulnerable groups, while also addressing supply constraints, structural issues, and logistical barriers to ensure equity in delivery.

Vaccine rollout has also imposed additional demands on health systems that are already overwhelmed with treating patients with COVID-19. Conversely, individuals are fearful of contracting COVID-19 and have had restricted mobility because of stay-at-home mandates and transportation disruptions. The implications for health-care-seeking behaviour, particularly for women and disadvantaged populations, have been inadequately investigated. Sizeable decreases in hospitalisations, as well as emergency and outpatient visits, have been observed across many locations during the pandemic.^52^, ^53^, ^54^ In low-income and middle-income countries, HIV, tuberculosis, and malaria programmes,^55^ as well as childhood routine immunisation,^56^ also face ongoing challenges, threatening decades of progress.^57^, ^58^ In addition to being more likely to report overall disruptions in health care,^11^ we found that women were also affected by disruptions in the provision of sexual, reproductive, and maternal health services. Although a population-based study in four sub-Saharan African areas did not find a negative effect of the COVID-19 pandemic on access to (and use of) contraceptive services by women in the earliest stages of the pandemic,^59^ a survey done by WHO showed that disruptions in maternal and reproductive care were still reported by more than 35% of countries in early 2021.^60^ These negative consequences of the pandemic raise concerns about progress towards gender equality in health. If left unaddressed, these inequalities are likely to deepen as the pandemic continues. Thus, as we prepare for years to come, gender-responsive preparedness plans that take the intersectional need of women, men, and gender minorities into account are necessary.^2^, ^28^

In addition to the health-related effects of the pandemic, the gender impacts on other key sectors of wellbeing have also been widely discussed.^19^ Consistent with previous studies,^61^, ^62^, ^63^, ^64^ we found that women were disproportionately affected by the economic fallout of the pandemic, with greater rates of employment loss and increases in uncompensated (and frequently unrecognised) labour. Unlike previous recessions,^65^ the most affected sectors during the COVID-19 pandemic were traditionally dominated by women, including retail and hospitality. Although evidence is sparse, immigrants, people from minority ethnic backgrounds, and women who are in poverty are most likely to be among the most severely impacted by the pandemic, as they are disproportionately represented in low-wage and informal positions, and frequently lack social support.^66^ Moreover, gendered social norms in many countries attribute household and childcare responsibilities preferentially to women and reduce their time and ability to engage in paid labour.^22^, ^67^ Based on the 2007 financial crisis, it is likely that, as the pandemic ebbs, men are likely to be rehired at higher rates than women. Unless mitigating policies are implemented, longer periods of unemployment for women will result, with severe implications for long-term income and career progression.^67^, ^68^ Without purposeful policy action to boost gender parity,^70^ reduced participation of women in the workforce is likely to persist in the future which some estimates indicate could lead to an international US$1 trillion reduction in global gross domestic product by 2030.^61^

Education and schooling of girls and women can also have profound and long-term implications for the economic and social development of societies. The scarcity of available evidence reinforces concerns about rising gender gaps and supports the widening of the previously known so-called double-sided gender disparity in education, in which girls are overtaking boys in schooling in many high-income settings, while large gaps remain in several low-income and middle-income countries.^71^ In our study, girls were slightly less likely than boys to have dropped out of school during the COVID-19 pandemic in high-income countries, although large gaps for girls can be seen in other world regions. Girls typically excel in school in many regions, but early indications during the COVID-19 pandemic substantiate fears that school closures will further exacerbate unpaid care work for girls and women^72^ and that teenage girls might disproportionately drop out of school, as has been the case in previous health crises.^21^, ^73^ Prioritising the closing of gender gaps in schooling and targeting interventions to ensure the return of girls and women to school remains a crucial goal for advancing the human capital of societies.

Lastly, gender-based violence, which disproportionately affects women and is challenging to monitor, has also garnered substantial attention during the COVID-19 pandemic. Media and domestic violence services reports have suggested an increase in violence and barriers to accessing services as mobility restrictions forced many to stay home.^15^, ^16^, ^74^ Yet, reliable information on safety and violence is uniquely difficult to collect via traditional data systems, and these challenges have been exacerbated during the COVID-19 pandemic.^75^ The administration of surveys via smartphones introduces various ethical and safety challenges for collecting information from respondents who are likely to be in the same physical space as their abusers. Although results show that the COVID-19 pandemic has exacerbated women's perceived risks of violence and levels of feeling unsafe at home, these indirect measures are hard to interpret in an actionable way. Inferences about the negative impact of the pandemic on violence against women are also commonly drawn from past outbreaks,^75^ such as Ebola or Zika virus.^76^, ^77^ Even though there are multiple indications that the COVID-19 pandemic has potentially exacerbated gender-based violence and complicated service provision for those experiencing violence,^15^, ^16^, ^78^ it is worth emphasising that challenges in addressing gender-based violence and inadequate service provision long predate the pandemic. The crucial need for better evidence and sufficient resources allocated to this important health, societal, and humanitarian problem has always been urgent, and has now become even more so.

The findings of this study should be interpreted in light of its limitations, the most important of which relates to the mode of data collection that has been used extensively throughout the pandemic. First, most available information was self-reported in surveys distributed via apps and online platforms to smartphone users. Although this method provides large sample sizes and timely information, the respondents do not represent a random sample, particularly in countries with low smartphone ownership and high levels of illiteracy. Second, self-reported data have additional concerns related to social desirability bias (particularly related to questions around COVID-19). In addition, we find that in self-administered surveys delivered via smartphone, information on demographic characteristics of respondents is missing at higher rates than in in-person or telephone surveys. Although we have sought to account for potential implications, high missingness of key indicators (eg, age, gender, and education) remains a challenge. Third, data for gender, while self-reported, are constrained by limited response options in most instruments, excluding gender and sexual minorities, which often conflate gender and sex. Thus, we have only been able to explore differences between respondents who self-identify as women and men, even though the pandemic might have exacerbated health and social disparities among already marginalised gender-minority populations.^7^, ^79^, ^80^ Fourth, because of data sparsity, we were not able to further explore the intersection of gender and other categories including race, and socioeconomic and migration status, despite evidence that the extent of the impact of the pandemic varies greatly according to these characteristics.^7^ Even some key pandemic-related indicators, such as schooling disruptions, have extremely sparse data coverage and small sample sizes, and the interpretation of findings should be conservative. Fifth, most of the data used in this analysis did not allow us to draw conclusions as to whether the observed differences and trends were due to COVID-19 or whether they existed before the pandemic. As a result, our ability to attribute causality of the observed differences to COVID-19 is limited. Sixth, this study was based exclusively on quantitative data and would benefit from being complemented with evidence from qualitative studies of individuals' perceptions and experiences throughout the pandemic. Seventh, we were not able to differentiate data sources on the basis of quality, and have effectively weighted them only by sample size and number of countries where data were collected. Finally, we focused on indirect social and economic pandemic outcomes, not direct health outcomes (eg, mental health or COVID-19 burden), which have been covered elsewhere^2^, ^81^ and remain crucial.

Gender-disaggregated data for the key impacts of COVID-19 in health and social sectors continue to be scarce. Reliable and timely data for gender gaps can have a crucial role in contributing to real-time evidence-based policies that promote gender equality. The findings of this study on significant and increasing gender gaps in workforce participation and unpaid labour highlight the need for all societies around the world to invest in the provision of gender-responsive social protection measures during the pandemic. Fostering women's economic empowerment can, in turn, also contribute to the ability of women to overcome barriers they face in health care and enable them to situate themselves in environments that minimise their risk of gender-based violence. As women have disproportionately shouldered the burden of caring for others and chores in the household during the COVID-19 pandemic, the importance of changing long-standing widespread attitudes about the role of women in society cannot be overstated. An immediate call to action, with both short-term and long-term gains, is for political and social leaders to focus on all girls and women returning to school; this would represent a significant investment in the human capital of all societies and ensure growth in the empowerment and equality of women in the coming years.

## Data sharing

Data sources are listed by location and institution in appendix 2. Data inputs and metadata are available at http://ghdx.healthdata.org. Additional information about this study, as well as tabulated results, are available at http://ghdx.healthdata.org/record/ihme-data/covid_19_gender_equality_impacts. All code used for generating the results are available at https://github.com/ihmeuw/gem.

**This online publication has been corrected. The corrected version first appeared at thelancet.com on June 25, 2022**

## Declaration of Interests

We declare no competing interests.
