# Supplementary material for: Quantifying the effects of the COVID-19 pandemic on gender equality on health, social, and economic indicators: a comprehensive review of data from March, 2020, to September, 2021
Source: Lancet. 2022 Jun 25;399(10344):2381–97. doi: 10.1016/S0140-6736(22)00008-3 (PMC8890763; doi:10.1016/S0140-6736(22)00008-3)
Supplement: Supplementary appendix [file mmc1.pdf]

THE LANCET

Supplementary appendix

This appendix formed part of the original submission and has been peer reviewed. We post it as supplied by the authors.

Supplement to: Flor LS, Friedman J, Spencer CN, et al. Quantifying the effects of the COVID-19 pandemic on gender equality on health, social, and economic indicators: a comprehensive review of data from March, 2020, to September, 2021. *Lancet* 2022; published online March 2. https://doi.org/10.1016/S0140-6736(22)00008-3.

Supplementary appendix 1 to "Quantifying the effects of the COVID-19 pandemic on gender equality on health, social, and economic indicators: a comprehensive review of data from March, 2020, to September, 2021"

This appendix provides further methodological detail, supplemental figures, and more detailed results. The appendix is organised into broad sections following the structure of the main paper.

Preamble

This appendix provides further methodological detail and more detailed results for "Quantifying the effects of the COVID-19 pandemic on gender equality on health, social, and economic indicators: a comprehensive review of data from March, 2020, to September, 2021". This study complies with the Guidelines for Accurate and Transparent Health Estimates Reporting (GATHER) recommendations. It includes detailed tables and information on data to maximise transparency in our estimation processes and provide a comprehensive description of analytical steps.

Table of Contents

1. Gather Checklist 5

2. Data sources 6

2.1 COVID-19 Behavior Tracker 2020 – YouGov 6

2.2 Global Health 50/50 COVID-19 Sex-Disaggregated Data Tracker 6

2.3 Centers for Disease Control and Prevention (CDC) 6

2.4 Our World in Data 6

2.5 COVerAGE-DB 6

2.6 University of Maryland Social Data Science Center Global COVID-19 Trends and Impact Survey (UMD Global CTIS) 6

2.7 The Delphi Group at Carnegie Mellon University US COVID-19 Trends and Impact Survey (Delphi US CTIS); 7

2.8 Premise General Population COVID-19 Health Services Disruption Survey 2020 7

2.9 Premise General Population COVID-19 Health Services Disruption Survey 2021 7

2.10 COVID-19 Rapid Gender Assessment Survey (RGA) 7

2.11 Survey on Gender Equality at Home 7

2.12 Measuring COVID-19 Impacts, Mitigation and Awareness Survey (FINMRK) 8

2.13 Research for Effective Covid-19 Response Panel Survey (RECOVR) 8

2.14 Covid-19 High Frequency Phone Survey 8

3. Data processing 9

3.1 Vaccine Hesitancy and Uptake 9

3.2 Healthcare services 13

3.3 Economic and work-related concerns 28

3.4 Education 39

3.5 Safety at Home and in the Community 42

4. Methods 47

4.1 Time Series Modeling Overview 47

4.2 Cross-sectional Data Synthesis 48

4.3 Multivariate Regression Analyses 49

5. Supplemental Tables and Figures 51

Supplemental Table S1. Total sample size and the percent women respondents for each indicator 51

Supplemental Table S2. Results from the mixed effects logistic regression for sexual and reproductive health 53

Figure S1. Data availability for each indicator by data source provider. 54

Figure S2. Number of data sources available by country and indicator for Central Europe, Eastern Europe, and Central Asia....55

Figure S3. Number of data sources available by country and indicator for Latin America and the Caribbean....56

Figure S4. Number of data sources available by country and indicator for High-income countries....57

Figure S5. Number of data sources available by country and indicator for South Asia. 58

Figure S6. Number of data sources available by country and indicator for North Africa and the Middle East....59

Figure S7. Number of data sources available by country and indicator for Southeast Asia, East Asia, and Oceania. 60

Figure S8. Number of data sources available by country and indicator for Sub-Saharan Africa....61

Figure S9. Regional-level relative gender gaps in time-varying Indicators....62

Figure S10. Country-Level Gender Disparities in Time-Varying Indicators in January 2021....63

Figure S11. Country-Level Gender Disparities in Time-Varying Indicators in September 2021 65

Figure S12. Country-Level Trends by Gender in Cross-Sectional Indicators....67

Figure S13. Time-specific multivariate logistic regression results from UMD Global CTIS and Delphi US CTIS Surveys, for Healthcare, Economic and work-related concerns, and Vaccine hesitancy and uptake indicators. 69

Figure S14. Significance of the gender coefficient across indicators, sources and regions....71

Figure S15. Cross-sectional gender gaps, presented by world region and indicator. 73

Figure S16. Timeseries Models and Input Data 75

1. Gather Checklist

Item #	Checklist item	Reported on page #
**Objectives and funding**		
1	Define the indicator(s), populations (including age, sex, and geographic entities), and time period(s) for which estimates were made.	*Methods (data sources); Table; Appendix 1 page 9-44*
2	List the funding sources for the work.	*Summary*
**Data Inputs**		
For all data inputs from multiple sources that are synthesized as part of the study:		
3	Describe how the data were identified and how the data were accessed.	*Methods (data sources); Appendix 1 page 9-44*
4	Specify the inclusion and exclusion criteria. Identify all ad-hoc exclusions.	*NA*
5	Provide information on all included data sources and their main characteristics. For each data source used, report reference information or contact name/institution, population represented, data collection method, year(s) of data collection, sex and age range, diagnostic criteria or measurement method, and sample size, as relevant.	*Appendix 1 page 6-8 and Appendix 2*
6	Identify and describe any categories of input data that have potentially important biases (e.g., based on characteristics listed in item 5).	*Methods (Definitions and data processing); Discussion*
For data inputs that contribute to the analysis but were not synthesized as part of the study:		
7	Describe and give sources for any other data inputs.	*NA*
For all data inputs:		
8	Provide all data inputs in a file format from which data can be efficiently extracted (e.g., a spreadsheet rather than a PDF), including all relevant meta-data listed in item 5. For any data inputs that cannot be shared because of ethical or legal reasons, such as third-party ownership, provide a contact name or the name of the institution that retains the right to the data.	*Appendix 2* https://github.com/ihmeuw/GEM
**Data analysis**		
9	Provide a conceptual overview of the data analysis method. A diagram may be helpful.	*Methods (Statistical analysis); Appendix 1 page 45-48*
10	Provide a detailed description of all steps of the analysis, including mathematical formulae. This description should cover, as relevant, data cleaning, data pre-processing, data adjustments and weighting of data sources, and mathematical or statistical model(s).	*Appendix 1 page 9-48*
11	Describe how candidate models were evaluated and how the final model(s) were selected.	*Appendix 1 page 48*
12	Provide the results of an evaluation of model performance, if done, as well as the results of any relevant sensitivity analysis.	*Appendix 1 page 48*
13	Describe methods for calculating uncertainty of the estimates. State which sources of uncertainty were, and were not, accounted for in the uncertainty analysis.	*Appendix 1 page 48*
14	State how analytic or statistical source code used to generate estimates can be accessed.	https://github.com/ihmeuw/GEM
**Results and Discussion**		
15	Provide published estimates in a file format from which data can be efficiently extracted.	http://ghdx.healthdata.org/record/ihme-data/covid_19_gender_equality_impacts
16	Report a quantitative measure of the uncertainty of the estimates (e.g. uncertainty intervals).	*Results*
17	Interpret results in light of existing evidence. If updating a previous set of estimates, describe the reasons for changes in estimates.	*Discussion*
18	Discuss limitations of the estimates. Include a discussion of any modelling assumptions or data limitations that affect interpretation of the estimates.	*Discussion*

2. Data sources

Data sources used for the analysis are described below. Detailed information on each source is also provided in Appendix 2.

2.1 COVID-19 Behavior Tracker 2020 – YouGov

This is a collaborative project between the Institute of Global Health Innovation at Imperial College London and YouGov, an Internet-based market research firm. The project administers surveys in 29 countries, areas, and territories, and it collects information on COVID-19 and its associated behaviors and consequences. This includes topics like mask use, healthcare disruptions, vaccinations, and more. The goal is to provide key insights on how different populations and regions are responding to the pandemic, which consequently informs the decisions public health agencies. The COVID-19 Behavior Tracker 2020 published anonymized data at the respondent level, which allows for analytical flexibility.

2.2 Global Health 50/50 COVID-19 Sex-Disaggregated Data Tracker

Global Health 50/50 created a COVID-19 Sex-Disaggregated Data Tracker to understand how sex and gender influence the development of and response to the pandemic. This tracker collects government-reported, sex-disaggregated data on COVID-19 vaccines, cases, and deaths, but only vaccine information was used in this report. The vaccine-specific data from Global Health 50/50 cover 17 unique locations.

2.3 Centers for Disease Control and Prevention (CDC)

The CDC reports the cumulative number of administered COVID-19 vaccinations in the United States by day. These numbers are disaggregated by sex, age, and race/ethnicity in an attempt to comprehensively describe the demographic profile of vaccine recipients. Here, vaccine recipients include those who have received any vaccination and those who have been fully vaccinated.

2.4 Our World in Data

Our World in Data is a complete COVID-19 dataset that includes non-sex-disaggregated information on COVID-19 vaccinations, tests, confirmed cases and deaths, and more. All data come from official reports, such as those published by governments and statistical agencies. Our World in Data reports both the number of people who have received any vaccination and the number of people who have been fully vaccinated. The dataset covers 190 unique locations.

2.5 COVerAGE-DB

The Max Planck Institute for Demographic Research created a database of COVID-19 cases, deaths, and vaccinations reported by various statistical agencies. Vaccination data includes the number of people who have received any vaccine and the number of people who have been fully vaccinated. COVerAGE-DB has information from four unique locations: Peru, Lithuania, Denmark, and New Zealand.

2.6 University of Maryland Social Data Science Center Global COVID-19 Trends and Impact Survey (UMD Global CTIS)

The UMD Global CTIS is a partnership between Facebook and academic institutions. The survey is available in 56 languages. A representative sample of Facebook users is invited on a daily basis to report on topics including, for example, symptoms, social distancing behavior, vaccine acceptance, mental health issues, and financial constraints. Facebook provides weights to reduce nonresponse and coverage

bias. Country and region-level statistics are published daily via public API and dashboards, and microdata is available for researchers via data use agreements. Over half a million responses are collected daily.

2.7 The Delphi Group at Carnegie Mellon University US COVID-19 Trends and Impact Survey (Delphi US CTIS);

In collaboration with Facebook, along with a consortium of universities and public health officials, the Delphi group at Carnegie Mellon University conducts research surveys to monitor the spread and impact of the COVID-19 pandemic in the United States. This survey is advertised through Facebook. It has run continuously since early April 2020, and about 50,000 people in the United States participate every day. Survey results are publicly available on our National COVID Survey Dashboard, COVIDcast Map, COVIDcast Exporter, and in our COVIDcast API.

2.8 Premise General Population COVID-19 Health Services Disruption Survey 2020

The COVID-19 Health Services Disruption Survey 2020 is a series of surveys developed to assess the level of disruption to a range of health services resulting from the COVID-19 pandemic and subsequent government mandates and changes in behavior to mitigate the spread of the disease. This survey was conducted in 76 countries using the smartphone-based Premise data collection platform. Respondents were individual members of the general population. Data using unique questionnaires were collected to address each of the following areas of focus: general population health services (52,490 respondents), malaria (14,615 respondents), maternal/infant health (2,129 respondents), children's health (7,228 respondents), and women's health (12,354 respondents).

2.9 Premise General Population COVID-19 Health Services Disruption Survey 2021

The COVID-19 Health Services Disruption Survey 2021 is a series of surveys developed to assess the level of disruption to a range of health services resulting from the COVID-19 pandemic and subsequent government mandates and changes in behavior to mitigate the spread of the disease. This survey was conducted in 76 countries using the smartphone-based Premise data collection platform. Respondents were individual members of the general population. Data were collected from 18,642 respondents. Data using unique questionnaires were collected to address each of the following areas of focus: general population health services (18,642 respondents), malaria (4,870 respondents), maternal/infant health (2,282 respondents), children's health (7,383 respondents), women's health (4,319 respondents) and education and gender (23,352 respondents).

2.10 COVID-19 Rapid Gender Assessment Survey (RGA)

The COVID-19 Rapid Gender Assessment Surveys, conducted in 52 countries, were developed by UN Women as a global effort to increase data availability regarding the gendered impacts of COVID-19. Country-level telephone surveys cover the following topics: demographics, economic activities, agriculture, education, gender-based violence, health, human rights. The population for these surveys are women and men aged 18 years or older with access to mobile phones.

2.11 Survey on Gender Equality at Home

This survey was conducted with the purpose of capturing gender disparities at home during the COVID-pandemic. Data was collected through an online survey on the Facebook platform once in July 2020 and it reached 208 countries, territories, and islands from all regions of the world.

2.12 Measuring COVID-19 Impacts, Mitigation and Awareness Survey (FINMRK)

Conducted by FinMark Trust, this survey has the purpose of measuring the impact of COVID-19 in Africa on finances, food security, healthcare access and behaviors towards and due to the pandemic. Data was collected through mobile computer-aided telephonic interviews (mCATI) since April of 2020 in Kenya, Nigeria, Rwanda and South Africa and later on expanded to Ghana, Uganda and Zambia. Multiple cross-sectional rounds of data were collected with the aim of having 5 waves per country.

2.13 Research for Effective Covid-19 Response Panel Survey (RECOVR)

Developed by Innovations for Poverty Action (IPA), this survey has the objective of tracking COVID-19 effects on people's lives to inform policy making. Two rounds of panel data were expected to be collected through Computer-assisted telephone interviewing (CATI). The first rounds between May and July of 2020 and the second wave between July and December of 2020. Data was collected in countries from diverse regions of the world such as Burkina Faso, Colombia, Cote d'Ivoire, Ghana, Mexico, Philippines, Rwanda, Sierra Leone, Uganda and Zambia.

2.14 Covid-19 High Frequency Phone Survey

The COVID-19 High Frequency Phone Survey is a collection of surveys produced by the Data Production and Methods Unit of the Development Data Group, World Bank. The surveys collect data for epidemics and pandemics, such as COVID-19, including data on the economic and social impacts of the COVID-19 pandemic through longitudinal telephone household interviews in 21 countries. Sampling is done from subsets of Living Standards Measurement Study (LSMS) Surveys.

3. Data processing

3.1 Vaccine Hesitancy and Uptake

Vaccine hesitancy

We used the sources listed in Table 1 to compute the proportion of respondents who were hesitant to get an existing COVID-19 vaccine. Each source sampled men and women 18 years and older.

While we identified another global survey of vaccine hesitancy (*A Global Survey of Potential Acceptance of a COVID-19 Vaccine*) as another possible data source, we were not able to use it due to differences in case definition. The survey collected information before the completion of human trials, so it asked respondents about their acceptance of a hypothetical vaccine. Conversely, *COVID-19 Behavior Tracker 2020 - YouGov* and the *UMD Global CTIS* and *Delphi US CTIS* surveys asked about the acceptance of any existing COVID-19 vaccine.

Table 1. Input data for the vaccine hesitancy indicator.

Data Source	Question	Response options
UMD Global CTIS	Would you get a COVID-19 Vaccine, or Have you already Gotten One?	Yes definitely, Yes probably, No probably note, No definitely not
	Do you have an appointment to receive a COVID-19 vaccine?	Yes, No
Delphi US CTIS	Would you get a COVID-19 Vaccine, or Have you already Gotten One?	Yes definitely, Yes probably, No probably note, No definitely not
	Do you have an appointment to receive a COVID-19 vaccine?	Yes, No
COVID-19 Behavior Tracker 2020 - YouGov	If a Covid-19 vaccine is available to you, will you get it?	Yes, No, Not sure
	Have you had the first or second doses of a Coronavirus (COVID-19) vaccine?	No neither, Yes one dose, Yes two doses

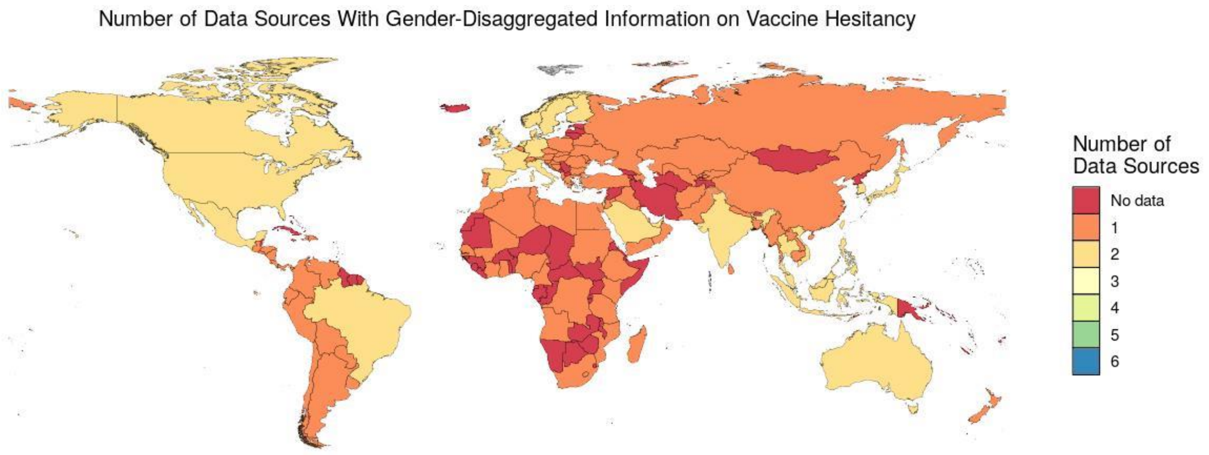

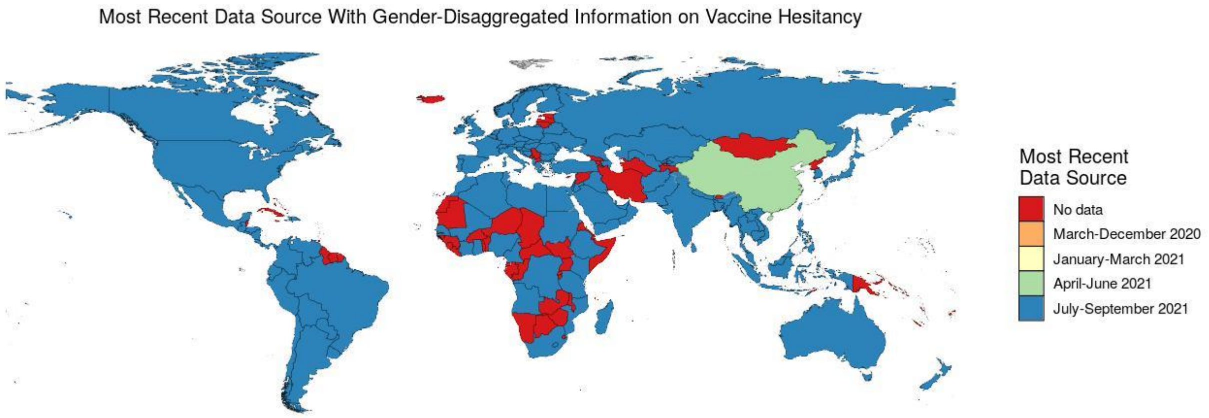

Data was processed to ensure that these indicators were comparable across data sources. Briefly, people were considered vaccine hesitant if they were not already vaccinated and if they were not planning to get vaccinated.

The *UMD Global CTIS* and *Delphi US CTIS* surveys have three questions related to vaccine reception, vaccine appointments, and vaccine hesitancy. Here, vaccine acceptance was defined as: having received a vaccine, having an appointment to get a vaccine, or intending to get a vaccine when it becomes available. Vaccine hesitancy was the opposite, and included those who were not already vaccinated, who did not have an appointment to get a vaccine, and did not intend to get the vaccine.

To create a vaccine hesitancy indicator from the *COVID-19 Behavior Tracker 2020*, two question were used: “If a Covid-19 vaccine is available to you, will you get it?” and “Have you had the first or second doses of a Coronavirus (COVID-19) vaccine?” Those who said they were willing to get a vaccine were characterized as “not hesitant.” Similarly, those who said they had already been vaccinated were characterized as “not hesitant.” If someone said they had not been vaccinated and responded “no” or “don’t know” to the hesitancy question, they were characterized as vaccine hesitant.

Both sources collected data on a daily basis. Daily responses were combined to create a weighted monthly proportion of vaccine hesitancy by age, gender, and country.

Fully Vaccinated

We used the sources listed in Table 2 to compute the proportion of people aged 18+ who are fully vaccinated. Official statistics from *COVerAGE-DB*, the *Centers for Disease Control and Prevention (CDC)*, *Our World in Data*, and *Global Health 50/50* were used in the analysis, and population estimates from IHME were used to determine the proportion of the population fully vaccinated. Unofficial reports—such as survey data from *YouGov's COVID-19 Behavior Tracker 2020* and the *UMD Global CTIS* and *Delphi US CTIS*

Surveys —were also included.

Gender-invariant data from *Our World in Data* and the *CDC* were used in the analysis, and all other sources were used to gender-split those official reports.

Table 2. Input data for the fully vaccinated indicator.

Data Source	Question	Response options
UMD Global CTIS	V1. Have you had a COVID-19 vaccination?	Yes, No, Don't know
	V2. How many COVID-19 vaccinations have you received?	1 vaccination or dose, 2 vaccinations or doses, I don't know
	V2a. Did you receive (or do you plan to received) all required doses?	Yes received all required doses, Yes plan to receive all required doses, No don't plan to receive all required doses
Delphi US CTIS	Have you had a COVID-19 vaccination?	Yes, No, Don't know
	How many COVID-19 vaccinations have you received?	1 vaccination or dose, 2 vaccinations or doses, I don't know
	Did you receive (or do you plan to received) all required doses?	Yes received all required doses, Yes plan to receive all required doses, No don't plan to receive all required doses
COVID-19 Behavior Tracker 2020 - YouGov	Have you had the first or second doses of a Coronavirus (COVID-19) vaccine?	No neither, Yes one dose, Yes two doses
CDC	NA	NA
Our World in Data	NA	NA
Global Health 50/50	NA	NA
COVerAGE-DB	NA	NA

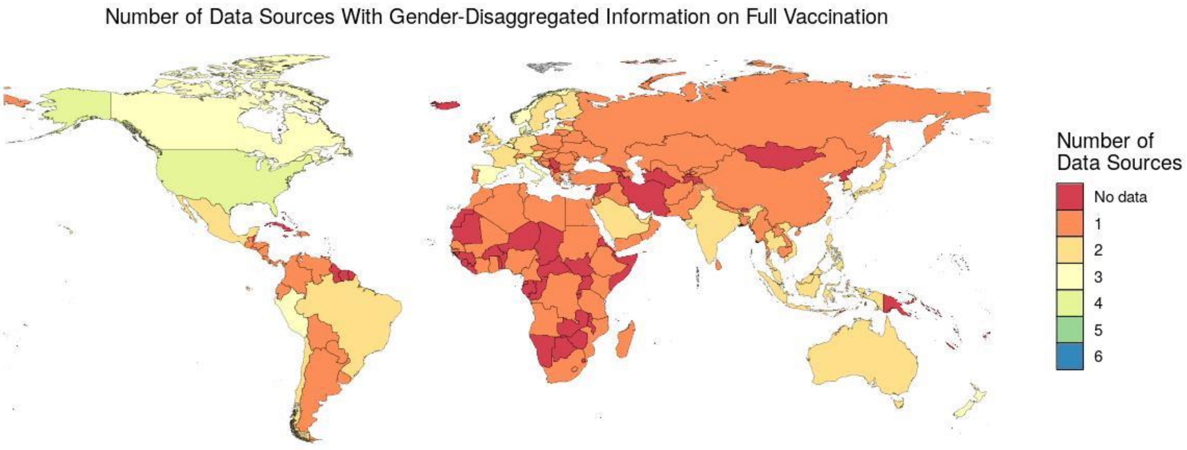

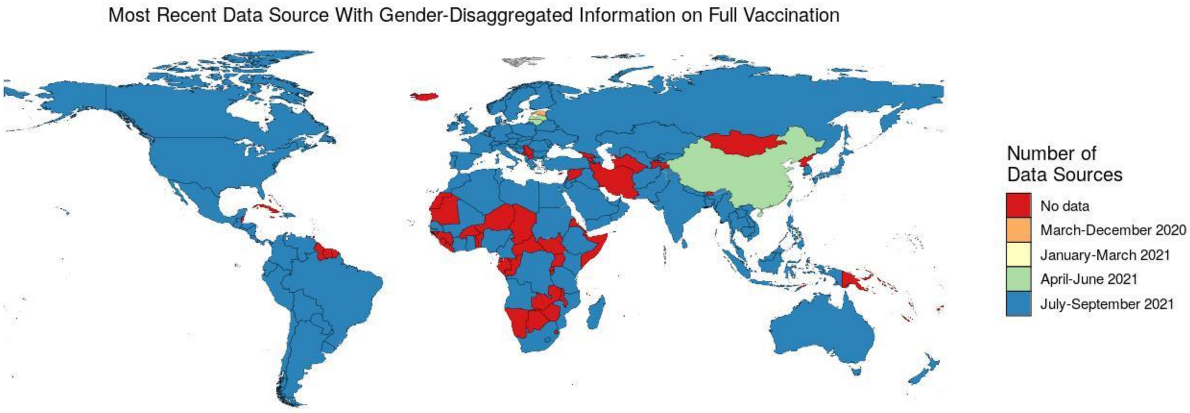

Data was processed to ensure that the indicators were comparable across data sources. For *COVID-19 Behavior Tracker 2020 - YouGov*, the *UMD Global CTIS* and the *Delphi US CTIS*, the proportion of respondents fully vaccinated was calculated using the questions listed in Table 2. Respondents who did not report sex or gender were excluded. Respondents who skipped or missed any of the vaccine questions were also excluded.

More specifically, the analysis of *YouGov's COVID-19 Behavior Tracker 2020* used the following logic: if someone said that they had received two vaccine doses, they were counted as fully vaccinated. If they reported only receiving one or zero doses, they were considered as partially vaccinated and not as fully vaccinated. Missing and skipped responses were excluded from the analysis.

The *UMD Global CTIS* and *Delphi US CTIS* surveys followed a similar logic, but there was an additional step needed to process the gateway question of "V1. Have you had a COVID-19 vaccination?" If someone said no, then they were not asked "V2. How many COVID-19 vaccinations have you received?" or "V2a. Did you receive (or do you plan to received) all required doses?" If a respondent stated that they had been vaccinated (V1), they were then prompted to report the number of doses they had received (V2). If they had received two, they skipped the last question, V2a. If they only received one dose or did not know how many doses they received, they were asked if they had received or if they planned to receive all required doses (V2a). Using that series of questions and gateways, the following logic was applied to calculate the number of people who had been fully vaccinated:

- If a respondent skipped or did not answer V1 they were excluded.
- If a respondent answered V1 by saying they were not vaccinated, they were included in the total sample but were not counted as a fully vaccinated individual.
- If someone answered V1 with “Yes” and V2 with “2 vaccinations or doses,” they were counted as fully vaccinated.
- If someone answered V1 with “Yes,” V2 with “1 vaccination or dose,” and V2a with “Yes received all required doses,” they were counted as fully vaccinated.
- If someone answered V1 with “Yes,” V2 with “1 vaccination or dose,” and V2a not with “Yes received all required doses,” they were not counted as fully vaccinated.

The *CDC*, *Our World in Data*, and *COVerAGE-DB*, on the other hand, reported the official number of fully vaccinated people for different countries. These numbers were divided by adult (18 or older) population estimates from IHME to calculate the proportion of people who have been fully vaccinated in a given country. Similarly, *Global Health 50/50* reported official fully vaccinated numbers, but this source also included its own population sizes. Again, the proportion of people fully vaccinated was calculated by dividing the number of people fully vaccinated by the total number of people in the population.

All sources collected data on a daily basis. Daily responses were combined to create monthly proportions of vaccine uptake by country. Gender-specific estimates were made using *YouGov’s COVID-19 Behavior Tracker 2020*, the *UMD Global CTIS*, and the *Delphi US CTIS*, *COVerAGE-DB*, and *Global Health 50/50*. The *CDC* and *Our World in Data*, on the other hand, were used to create overall measures that were not gender-specific. The gender specific measures, however, were later raked to match *Our World in Data* and *CDC* data.

3.2 Healthcare services

Any disruption in healthcare

We used the sources listed in Table 3 to compute the proportion of respondents who experienced a disruption in healthcare due to the COVID-19 pandemic among all respondents. The *UMD Global CTIS, COVID-19 Rapid Gender Assessment Survey (RGA),* and *Survey on Gender Equality at Home* sampled men and women 18 years and older. *Premise General Population COVID-19 Health Services Disruption Survey 2020 and 2021* sampled men and women 16 years and older.

Table 3. Input data for the any disruption in healthcare indicator.

<table>
<tr><th>Data Source</th><th>Question</th><th>Response options</th></tr>
<tr><td>UMD Global CTIS</td><td>In the last 30 days, was there any time when you needed any of the following health services or products but could not get it?</td><td></td></tr>
<tr><td></td><td>- Emergency transportation services or emergency rescue</td><td>Yes | No</td></tr>
<tr><td></td><td>- Medical care with overnight stay in any type of facility</td><td>Yes | No</td></tr>
<tr><td></td><td>- Medical or dental care or treatment without an overnight stay</td><td>Yes | No</td></tr>
<tr><td></td><td>- Preventive health services (including immunization/vaccination, family planning, prenatal/postnatal care, routine check-up services)</td><td>Yes | No</td></tr>
<tr><td></td><td>- Medication</td><td>Yes | No</td></tr>
<tr><td></td><td>- Mask, medical gloves, or other protective equipment</td><td>Yes | No</td></tr>
<tr><td></td><td>- Eyeglasses, hearing aid, crutches, band-aids/plasters, thermometer, or any other health product</td><td>Yes | No</td></tr>
<tr><td></td><td>In the last 30 days, were you unable to get needed treatment, services, medicine, or medical products for any of the following reasons?</td><td></td></tr>
<tr><td></td><td>- I didn't know where to go</td><td>Yes | No</td></tr>
<tr><td></td><td>- I couldn't afford the treatment, service, or product</td><td>Yes | No</td></tr>
<tr><td></td><td>- I was unable to travel to the health care provider (including because of transportation cost, safety, or physical limitations)</td><td>Yes | No</td></tr>
<tr><td></td><td>- I was afraid of being infected at the health care provider</td><td>Yes | No</td></tr>
<tr><td></td><td>- The treatment, service, or product was not available</td><td>Yes | No</td></tr>
<tr><td>COVID-19 Rapid Gender Assessment Survey (RGA) 2020 (Cote d’Ivoire, Central African Republic, Uganda, Mali, Guinea, Mozambique, Senegal, Ethiopia, Kenya, South Africa, Eswatini, Malawi)</td><td>As a result of COVID, I could not/it was difficult for me to access medical services/consultations or similar</td><td>Yes | No | Did not need services</td></tr>
<tr><td>Premise General Population COVID-19 Health Services Disruption Survey 2020</td><td>Since March 2020, did you have a need to see a health provider?</td><td>Yes | No</td></tr>
<tr><td></td><td>Were you able to see a health provider since March?</td><td>Yes | No</td></tr>
</table>

	What was the reason you were not able to see a health provider since March?	Health facility closed | Turned away from health facility | Treatment or tests unavailable | No transportation | Lack of money | Unable to access due to COVID-19 restrictions | Fear of being infected with COVID-19 | Partner or family does not approve | Other | Decline to respond
Premise General Population COVID-19 Health Services Disruption Survey 2021	In the last 30 days, was there any time when you needed any of the following health services or products but could not get it?<br>- Emergency transportation services or emergency rescue<br>- Medical care with overnight stay in any type of facility<br>- Medical or dental care or treatment without an overnight stay<br>- Preventive health services (including immunization/vaccination, family planning, prenatal/postnatal care, routine check-up services)<br>- Medication<br>- Mask, medical gloves, or other protective equipment<br>- Eyeglasses, hearing aid, crutches, band-aids/plasters, thermometer, or any other health product<br><br>In the last 30 days, were you unable to get needed treatment, services, medicine, or medical products for any of the following reasons?<br>- I didn't know where to go<br>- I couldn't afford the treatment, service, or product<br>- I was unable to travel to the health care provider (including because of transportation cost, safety, or physical limitations)<br>- I was afraid of being infected at the health care provider<br>- The treatment, service, or product was not available	Yes | No<br>Yes | No<br>Yes | No<br>Yes | No<br>Yes | No<br>Yes | No<br>Yes | No<br><br>Yes | No<br>Yes | No<br>Yes | No<br>Yes | No<br>Yes | No
Survey on Gender Equality at Home	Experienced because of COVID-19: Unable to seek medical care	Yes | No

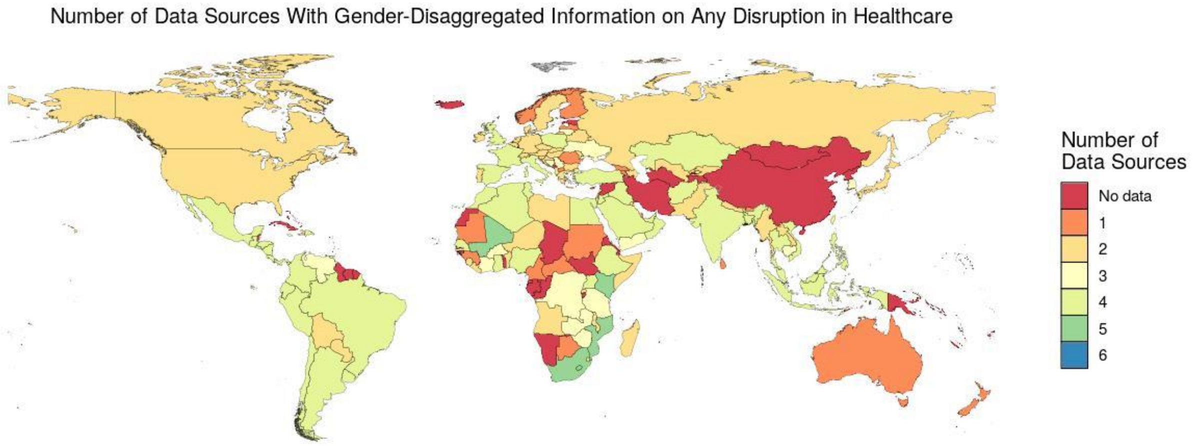

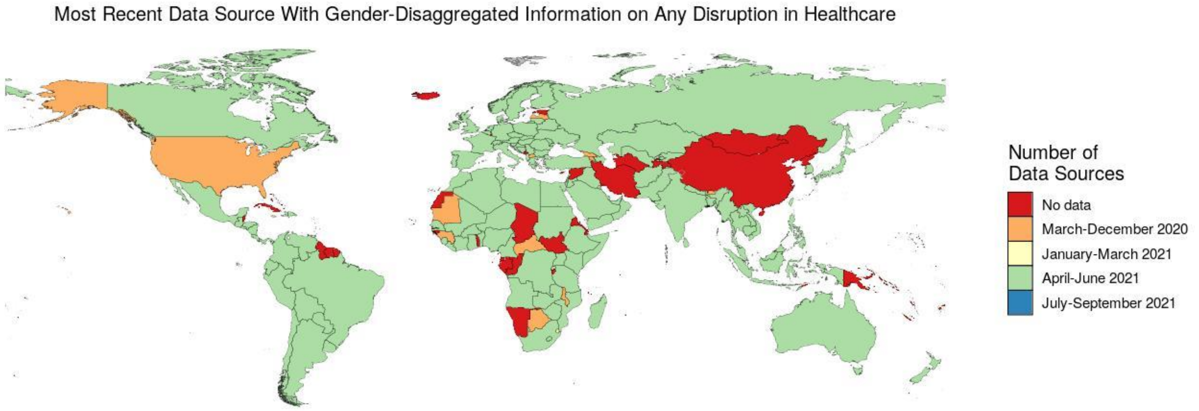

Data were processed to ensure that the indicator was comparable across data sources. Questions from the *UMD Global CTIS* and *Premise General Population COVID-19 Health Services Disruption Survey 2021* were processed such that respondents who answered yes to needing but not being able to get any one of: 1) medical care with overnight stay in any type of facility; 2) Medical or dental care or treatment without an overnight stay, or 3) Preventive health services (including immunization/vaccination, family planning, prenatal/postnatal care, routine check-up services) **and** answered yes to being unable to get treatment for either 1) I was afraid of being infected at the healthcare provider, or 2) I was unable to travel to the healthcare provider (including because of transportation cost, safety or physical limitations), were coded as positive responses (1s). The denominator was all respondents with non-missing answers to these questions.

Data from *COVID-19 Rapid Gender Assessment Surveys (RGA)* were processed such that answering 'yes' was coded as positive (1s) and the denominator was all respondents ('yes', 'no', 'did not need services').

Data from *Survey on Gender Equality at Home* were processed directly using the answer options listed (i.e. yes equates to a 1, no equates to a 0 for this indicator).

Data *from Premise General Population COVID-19 Health Services Disruption Survey 2020* were processed such that respondents who 1) answered 'yes' to needing to see a healthcare provider; 2) answered 'no' to being able to see a healthcare provider, and 3) listed their reason as either 'Unable to access due to COVID-19 restrictions' or 'Fear of being infected with COVID-19' were coded as a positive response (1). All respondents with non-missing data were included in the denominator.

Data from *Premise General Population COVID-19 Health Services Disruption Surveys 2020 and 2021*, *Survey on Gender Equality at Home*, and *COVID-19 Rapid Gender Assessment Surveys (RGA)* are cross-sectional in nature, thus cross-sectional proportions were calculated per source by collapsing across location and gender using weights provided by the respective survey. Data from the *UMD Global CTIS* are available in monthly intervals, and thus were collapsed by gender, location, and month to produce a time series of proportions calculated using survey weights. Cross-sectional and time-series proportions were only created for countries with a minimum of 30 observations per location and gender (and time, for indicators with monthly intervals available). Explanatory predictors for any disruption in healthcare were explored using individual level data from each source and the standard logistic regression framework described elsewhere in the appendix.

Disruption in reproductive health

We used *the COVID-19 Rapid Gender Assessment Surveys (RGAs)* (Table 4) to compute the proportion of respondents who reported a disruption in sexual or reproductive healthcare due to the COVID-19 pandemic among those with a need for this type of care. The *COVID-19 Rapid Gender Assessment Surveys (RGAs)* sampled men and women 18 years and older; however, we investigated this indicator only among adults of reproductive age, 18 to 44 years old. We additionally identified *Premise General Population COVID-19 Health Services Disruption Surveys 2020 and 2021* as potential data sources, but were not able to use these data to compute the disruption in reproductive health indicator due to low numbers of respondents reporting a need for this type of care, and thus too small of a sample to understand the role of barriers due to COVID-19.

Table 4. Input data for the disruption in reproductive health indicator.

Data Source	Question	Response options
COVID-19 Rapid Gender Assessment Survey (Cote d'Ivoire, Mali, Senegal, Guinea, Central African Republic)	As a result of COVID, I was unable/it was difficult to access the usual reproductive health or family planning (for example, contraception, testing and treatment for STDs and HIV, treatment infertility, care for survivors of gender-based violence, care related to pregnancy)	Yes | No | Did not need services

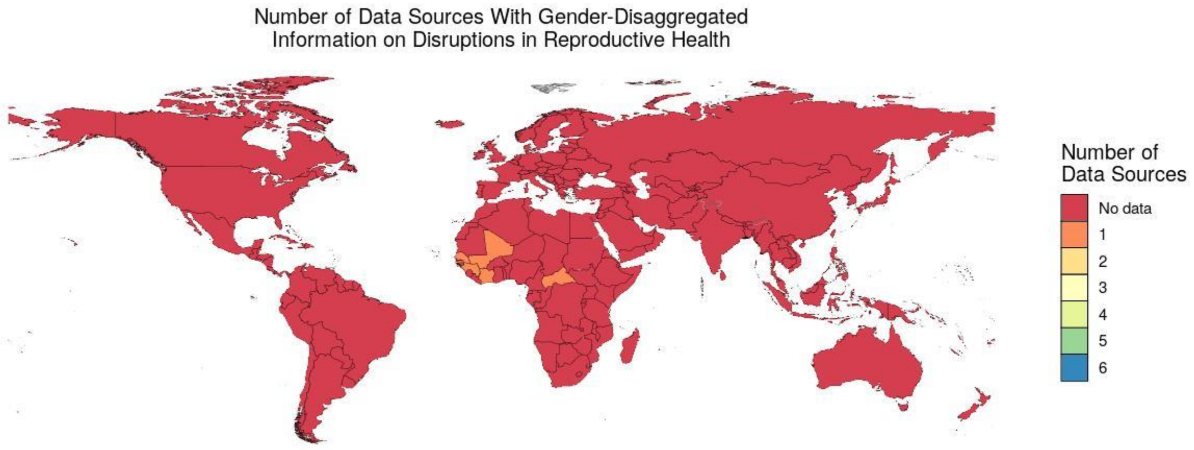

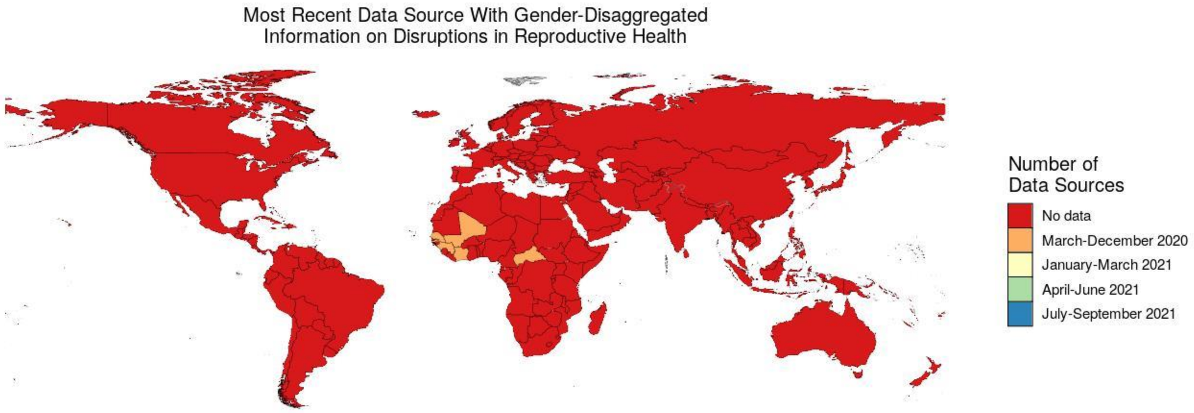

These data were investigated only among adults of reproductive age, 18 to 45 years old. Among these respondents, data were processed such that respondents who answered "yes" to the listed question were counted in the numerator (1s), and respondents who answered either "yes" or "no" were counted in the denominator, ensuring the indicator was contained to only those who needed care (i.e., those who reported not needing services were not included in the denominator).

The *COVID-19 Rapid Gender Assessment Surveys* provided cross-sectional data only, thus cross-sectional proportions were calculated by collapsing across location and gender using weights provided by the respective survey. Cross-sectional proportions were only created for countries with a minimum of 30 observations per location and gender. Explanatory predictors for experiencing a sexual or reproductive healthcare were explored using individual-level data and the standard logistic regression framework described elsewhere in the appendix.

Disruption in preventative care

We used the *UMD Global CTIS* listed in Table 5 to compute the proportion of respondents who experienced a disruption in preventive healthcare due to the COVID-19 pandemic among all respondents. The *UMD Global CTIS* sampled men and women 18 years and older. We additionally identified the

Premise General Population COVID-19 Health Services Disruption Survey 2021 as a potential data source but were unable to compute proportions due to a lack of sufficient respondents reporting a need for preventive healthcare services.

Table 5. Input data for the disruption in preventive care indicator.

Data Source	Question	Response options
UMD Global CTIS	In the last 30 days, was there any time when you needed any of the following health services or products but could not get it?	
	- Emergency transportation services or emergency rescue	Yes | No
	- Medical care with overnight stay in any type of facility	Yes | No
	- Medical or dental care or treatment without an overnight stay	Yes | No
	- Preventive health services (including immunization/vaccination, family planning, prenatal/postnatal care, routine check-up services)	Yes | No
	- Medication	Yes | No
	- Mask, medical gloves, or other protective equipment	Yes | No
	- Eyeglasses, hearing aid, crutches, band-aids/plasters, thermometer, or any other health product	Yes | No
	In the last 30 days, were you unable to get needed treatment, services, medicine, or medical products for any of the following reasons?	
	- I didn't know where to go	Yes | No
	- I couldn't afford the treatment, service, or product	Yes | No
	- I was unable to travel to the health care provider (including because of transportation cost, safety, or physical limitations)	Yes | No
	- I was afraid of being infected at the health care provider	Yes | No
	The treatment, service, or product was not available	Yes | No

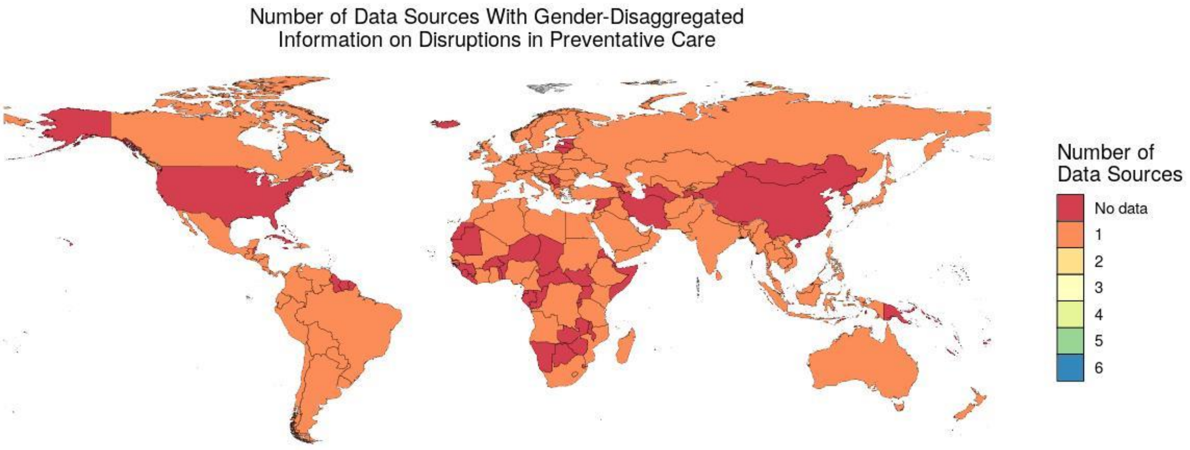

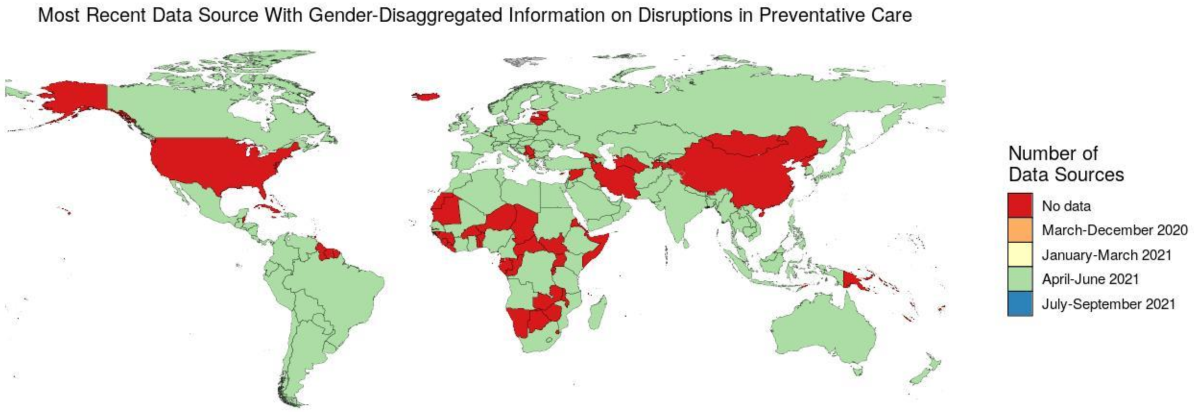

Data were processed such that respondents who answered yes to needing but not being able to get preventive health services (including immunization/vaccination, family planning, prenatal/postnatal care, routine check-up services) **and** answered yes to being unable to get treatment for either 1) I was afraid of being infected at the healthcare provider, or 2) I was unable to travel to the healthcare provider (including because of transportation cost, safety or physical limitations), were coded as positive responses (1s). The denominator was all respondents with non-missing answers to these questions.

Data from the *UMD Global CTIS* are available in monthly intervals, and thus were collapsed by gender, location, and month to produce a time series of proportions calculated using survey weights. Explanatory predictors for disruption in preventive care were explored using individual level data and the standard logistic regression framework described elsewhere in the appendix.

Disruption in medication access

We used the sources listed in Table 6 to compute the proportion of respondents who experienced a disruption in access to medication due to the COVID-19 pandemic among all respondents. The *UMD Global CTIS* and *Measuring COVID-19 Impacts, Mitigation and Awareness Survey (FINMRK)* sampled men and women 18 years and older. *Premise General Population COVID-19 Health Services Disruption Survey 2020 and 2021* sampled men and women 16 years and older.

Table 6. Input data for the any disruption in healthcare indicator.

Data Source	Question	Response options
UMD Global CTIS	In the last 30 days, was there any time when you needed any of the following health services or products but could not get it?<br>- Emergency transportation services or emergency rescue<br>- Medical care with overnight stay in any type of facility<br>- Medical or dental care or treatment without an overnight stay<br>- Preventive health services (including immunization/vaccination, family planning, prenatal/postnatal care, routine check-up services)<br>- Medication<br>- Mask, medical gloves, or other protective equipment<br>- Eyeglasses, hearing aid, crutches, band-aids/plasters, thermometer, or any other health product	 Yes | No<br>Yes | No<br>Yes | No<br>Yes | No<br>Yes | No<br>Yes | No<br>Yes | No
	In the last 30 days, were you unable to get needed treatment, services, medicine, or medical products for any of the following reasons?<br>- I didn't know where to go<br>- I couldn't afford the treatment, service, or product<br>- I was unable to travel to the health care provider (including because of transportation cost, safety, or physical limitations)<br>- I was afraid of being infected at the health care provider<br>- The treatment, service, or product was not available	 Yes | No<br>Yes | No<br>Yes | No<br>Yes | No<br>Yes | No
Premise General Population COVID-19 Health Services Disruption Survey 2020	In the last 6 months, did you have a health condition that required you to take medication?	Yes | No
	Since March 2020, did you miss any doses of medication?	Yes | No
	What was the reason you missed a dose of your medication since March 2020?	Health facility or pharmacy was closed | Turned away from health facility or pharmacy | Medication unavailable at facility | No transportation | Lack of money | Unable to access due to lockdown | Fear of being infected with COVID-19 | Partner or family does not approve | Forgot to take | Other | Decline to respond

Premise General Population COVID-19 Health Services Disruption Survey 2021	In the last 30 days, was there any time when you needed any of the following health services or products but could not get it?<br>- Emergency transportation services or emergency rescue<br>- Medical care with overnight stay in any type of facility<br>- Medical or dental care or treatment without an overnight stay<br>- Preventive health services (including immunization/vaccination, family planning, prenatal/postnatal care, routine check-up services)<br>- Medication<br>- Mask, medical gloves, or other protective equipment<br>- Eyeglasses, hearing aid, crutches, band-aids/plasters, thermometer, or any other health product<br><br>In the last 30 days, were you unable to get needed treatment, services, medicine, or medical products for any of the following reasons?<br>- I didn't know where to go<br>- I couldn't afford the treatment, service, or product<br>- I was unable to travel to the health care provider (including because of transportation cost, safety, or physical limitations)<br>- I was afraid of being infected at the health care provider<br>- The treatment, service, or product was not available	Yes | No<br>Yes | No<br>Yes | No<br>Yes | No<br>Yes | No<br>Yes | No<br>Yes | No<br><br>Yes | No<br>Yes | No<br>Yes | No<br>Yes | No<br>Yes | No
Measuring COVID-19 Impacts, Mitigation and Awareness Survey (FINMRK) (Ghana, Kenya, Nigeria, Rwanda, Uganda, South Africa, Zambia)	Have you needed medication in the past 7 days and could not get it?<br><br>Why could you not get medicine? Is it because...<br>- You did not have enough money<br>- Hospital/clinic/pharmacy was closed<br>- Travel is currently prohibited<br>- Medicines were unavailable<br>- Hospital/clinic/pharmacy was too crowded<br>- Was scared to go purchase it<br>- Too sick to travel	Yes | No<br><br>Yes | No<br>Yes | No<br>Yes | No<br>Yes | No<br>Yes | No<br>Yes | No<br>Yes | No

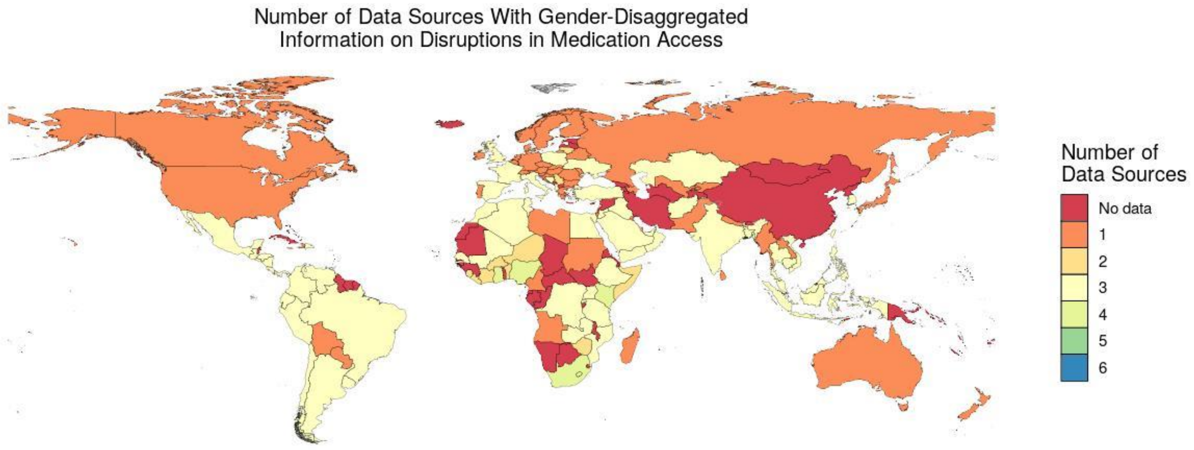

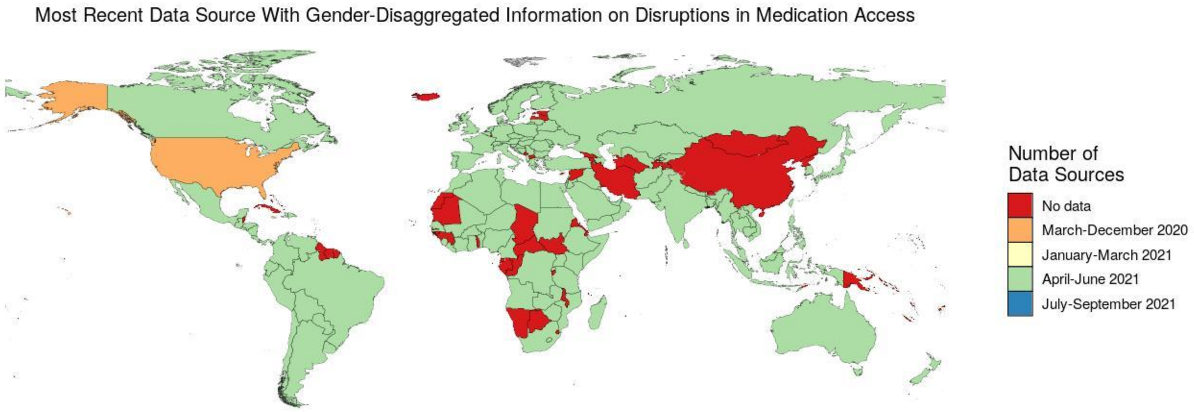

Data were processed to ensure that the indicator was comparable across data sources.

Questions from the *UMD Global CTIS and Premise General Population COVID-19 Health Services Disruption Survey 2021* were processed such that respondents who answered yes to needing but not being able to get medication **and** answered yes to being unable to get medicine for either 1) I was afraid of being infected at the healthcare provider, or 2) I was unable to travel to the healthcare provider (including because of transportation cost, safety or physical limitations), were coded as positive responses (1s). The denominator was all respondents with non-missing answers to these questions.

In data from *Premise General Population COVID-19 Health Services Disruption Survey 2020*, respondents were only asked about medication disruptions if they first responded that they had a health condition requiring them to take medication in the past 6 months. Medication disruptions from this source were coded such that respondents who answered yes to missing a dose of medication and the reason listed was either 'unable to access due to lockdown' or 'fear of being infected with COVID-19' were coded as positive responses (1s). The denominator was all respondents with non-missing answers to the medication questions listed.

Data from the *Measuring COVID-19 Impacts, Mitigation and Awareness Survey (FINMRK)* were processed such that respondents who responded yes to needing medication in the past 7 days and not being able to get it and yes to either 'travel is currently prohibited' or 'was scared to go purchase it' were coded as

positive responses (1s). The denominator was all respondents with non-missing answers to medication questions.

Data from *Premise General Population COVID-19 Health Services Disruption Survey 2020 and 2021* are cross-sectional in nature, thus cross-sectional proportions were calculated per source by collapsing across location and gender using weights provided by the respective survey. Data from the *UMD Global CTIS* are available in monthly intervals, and thus were collapsed by gender, location, and month to produce a time series of proportions calculated using survey weights. Data from the *Measuring COVID-19 Impacts, Mitigation and Awareness Survey (FINMRK)* are available in 'waved' intervals, and thus were collapsed by gender, location, and survey wave to produce a time series of proportions calculated using survey weights. Cross-sectional and time-series proportions were only created for countries with a minimum of 30 observations per location and gender (and time, for indicators with monthly intervals available). Explanatory predictors for disruption in medication access were explored using individual level data from the *UMD Global CTIS* and *Measuring COVID-19 Impacts, Mitigation and Awareness Survey (FINMRK)* and the standard logistic regression framework described elsewhere in the appendix. We were unable to carry out regressions using data from *Premise General Population COVID-19 Health Services Disruption Survey 2020 and 2021* due to a limited sample size and poor distribution of answers among all model covariates of interest.

Disruption in health products access

We used the sources listed in Table 7 to compute the proportion of respondents who experienced a disruption in access to health products due to the COVID-19 pandemic among all respondents. The *UMD Global CTIS and Survey on Gender Equality at Home* sampled men and women 18 years and older. *Premise General Population COVID-19 Health Services Disruption Survey 2021* sampled men and women 16 years and older.

Data were processed to ensure that the indicator was comparable across data sources. Questions from the *UMD Global CTIS* and *Premise General Population COVID-19 Health Services Disruption Survey 2021* were processed such that respondents who answered yes to needing but not being able to get eyeglasses, hearing aid, crutches, band-aids/plasters, thermometer, or any other health product **and** answered yes to being unable to get products for either 1) I was afraid of being infected at the healthcare provider, or 2) I was unable to travel to the healthcare provider (including because of transportation cost, safety or physical limitations), were coded as positive responses (1s). The denominator was all respondents with non-missing answers to these questions. Data from *Survey on Gender Equality at Home* and *COVID-19 Rapid Gender Assessment Surveys (RGA)* were processed directly using the answer options listed (i.e. 'yes' equated to 1 and 'no' equated to 0).

Table 7. Input data for the any disruption in healthcare indicator.

<table>
<tr><th>Data Source</th><th>Question</th><th>Response options</th></tr>
<tr><td>UMD Global CTIS</td><td>In the last 30 days, was there any time when you needed any of the following health services or products but could not get it?</td><td></td></tr>
<tr><td></td><td>- Emergency transportation services or emergency rescue</td><td>Yes | No</td></tr>
<tr><td></td><td>- Medical care with overnight stay in any type of facility</td><td>Yes | No</td></tr>
<tr><td></td><td>- Medical or dental care or treatment without an overnight stay</td><td>Yes | No</td></tr>
<tr><td></td><td>- Preventive health services (including immunization/vaccination, family planning, prenatal/postnatal care, routine check-up services)</td><td>Yes | No</td></tr>
<tr><td></td><td>- Medication</td><td>Yes | No</td></tr>
<tr><td></td><td>- Mask, medical gloves, or other protective equipment</td><td>Yes | No</td></tr>
<tr><td></td><td>- Eyeglasses, hearing aid, crutches, band-aids/plasters, thermometer, or any other health product</td><td>Yes | No</td></tr>
<tr><td></td><td>In the last 30 days, were you unable to get needed treatment, services, medicine, or medical products for any of the following reasons?</td><td></td></tr>
<tr><td></td><td>- I didn't know where to go</td><td>Yes | No</td></tr>
<tr><td></td><td>- I couldn't afford the treatment, service, or product</td><td>Yes | No</td></tr>
<tr><td></td><td>- I was unable to travel to the health care provider (including because of transportation cost, safety, or physical limitations)</td><td>Yes | No</td></tr>
<tr><td></td><td>- I was afraid of being infected at the health care provider</td><td>Yes | No</td></tr>
<tr><td></td><td>- The treatment, service, or product was not available</td><td>Yes | No</td></tr>
<tr><td>Premise General Population COVID-19 Health Services Disruption Survey 2021</td><td>In the last 30 days, was there any time when you needed any of the following health services or products but could not get it?</td><td></td></tr>
<tr><td></td><td>- Emergency transportation services or emergency rescue</td><td>Yes | No</td></tr>
<tr><td></td><td>- Medical care with overnight stay in any type of facility</td><td>Yes | No</td></tr>
<tr><td></td><td>- Medical or dental care or treatment without an overnight stay</td><td>Yes | No</td></tr>
<tr><td></td><td>- Preventive health services (including immunization/vaccination, family planning, prenatal/postnatal care, routine check-up services)</td><td>Yes | No</td></tr>
<tr><td></td><td>- Medication</td><td></td></tr>
</table>

	- Mask, medical gloves, or other protective equipment<br>- Eyeglasses, hearing aid, crutches, band-aids/plasters, thermometer, or any other health product<br><br>In the last 30 days, were you unable to get needed treatment, services, medicine, or medical products for any of the following reasons?<br>- I didn't know where to go<br>- I couldn't afford the treatment, service, or product<br>- I was unable to travel to the health care provider (including because of transportation cost, safety, or physical limitations)<br>- I was afraid of being infected at the health care provider<br>- The treatment, service, or product was not available	Yes | No<br>Yes | No<br>Yes | No<br><br>Yes | No<br>Yes | No<br>Yes | No<br>Yes | No<br>Yes | No
Survey on Gender Equality at Home	Experienced because of COIVD-19: Difficulty accessing medical or hygiene supplies	Yes | No
COVID-19 Rapid Gender Assessment Survey (RGA) 2020 (Colombia, Mexico)	As a result of the pandemic, have you had difficulty accessing (getting or buying) 2. Medical items; 3. Personal hygiene and sanitary products	Yes | No
COVID-19 Rapid Gender Assessment Survey (RGA) 2020 (Albania, Armenia, Azerbaijan, Bosnia and Herzegovina, Belarus, Serbia, North Macedonia, Georgia, Turkey, Moldova)	As a result of COVID19, did you (personally) experience difficulties in accessing basic services: hygiene and sanitary products (soap, water treatment tabs, menstrual products)	Yes | No

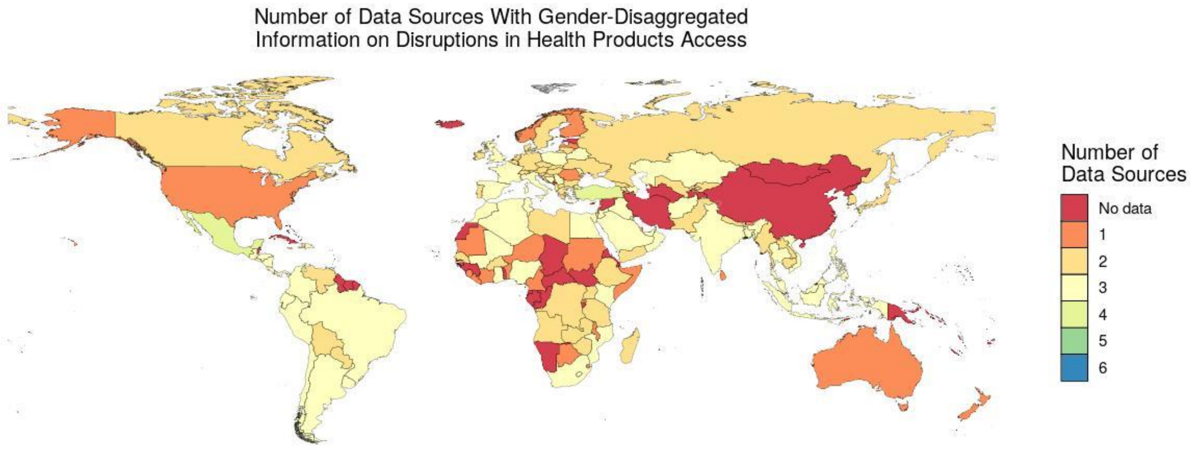

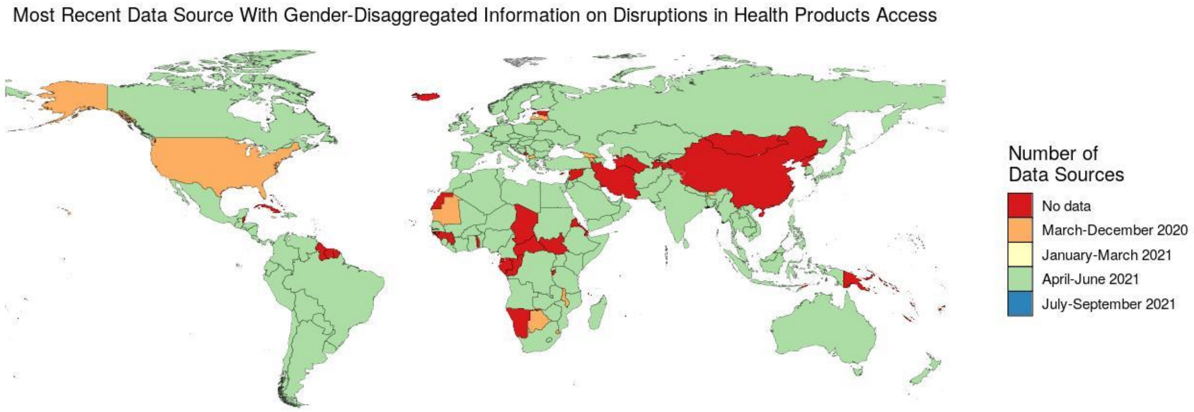

Data from *Premise General Population COVID-19 Health Services Disruption Survey 2021, COVID-19 Rapid Gender Assessment Surveys (RGAs),* and *Survey on Gender Equality at Home* are cross-sectional in nature, thus cross-sectional proportions were calculated per source by collapsing across location and gender using weights provided by the respective survey. Data from the *UMD Global CTIS* are available in monthly intervals, and thus were collapsed by gender, location, and month to produce a time series of proportions calculated using survey weights. Cross-sectional and time-series proportions were only created for countries with a minimum of 30 observations per location and gender (and time, for indicators with monthly intervals available). Explanatory predictors for disruption in health products access were explored using individual level data from the *Survey on Gender Equality at Home*, *COVID-19 Rapid Gender Assessment Survey (RGA)*, and the *UMD Global CTIS* and the standard logistic regression framework described elsewhere in the appendix. We were unable to carry out regressions using data from *Premise General Population COVID-19 Health Services Disruption Survey 2021* due to a limited sample size and poor distribution of answers among all model covariates of interest.

3.3 Economic and work-related concerns

Employment loss

We used the sources listed in Table 8 to compute the proportion of employment loss since the COVID-19 pandemic started. All surveys interviewed men and women 18 years old or above, except the *COVID-19 Health Services Disruption Survey* in 2020 and 2021 which interviewed men and women 16 years old or above.

Data were processed to make the employment loss indicator consistent across sources. Employment loss was calculated using two questions from the *UMD Global CTIS* (cross sectional time series), *COVID-19 High Frequency Survey* (cross sectional), *COVID-19 Health Services Disruption Survey* 2020 and 2021 (two repeated cross-sectionals), *Survey on Gender Equality at Home* (cross sectional), and *COVID-19 Behavior Tracker 2020*. Employment loss was calculated among those who worked before the pandemic, and it was defined as working before the pandemic but not currently working now. Cross-sectional proportions were calculated by collapsing across location and gender. Monthly proportions were calculated using data from the *UMD Global CTIS* collapsed across location and gender using their respective weights.

For the *Global COVID-19 Trends and Impacts Survey*, respondents who worked before February 2020 but were not currently working were included in the numerator and the denominator. All other currently working respondents were also included in the denominator, and we assumed that current employment indicated working before the pandemic as well. The denominator, in other words, included all respondents who worked before the pandemic, irrespective of their current employment status.

For both *COVID-19 Heath Services Disruption survey* and *COVID-19 High Frequency Phone Survey*, respondents who were working before the pandemic but were not working now were defined as 1 in the numerator, and those who were currently working were defined as 0.

In the case of the *Survey on Gender Equality at Home*, employment loss was defined as 1 if the individual selected “Lost a job” as a result of the COVID-19 pandemic, and 0 otherwise. When calculating the proportion of those who lost their job, the denominator for this variable was respondents currently working and individuals currently not working who lost their job.

Employment loss in *COVID-19 Behavior Tracker 2020 – YouGov* was defined as 1 if the individual selected “Yes, within the past 2 weeks”, “Yes, between 2 weeks and 2 months ago” or “Yes, more than two months ago but since 1st February 2020” on the question that asked whether they lost their job, and they were currently not working. Individuals were defined as 0 when they answered “No” to the question if they lost their job and were currently working.

Table 8. Input data for the employment loss indicator.

Data Source	Question	Response options
UMD Global CTIS	In the last 7 days, did you do any work for pay, or do any kind of business, farming, or other activity to earn money, even if only for one hour?	Yes<br>No
UMD Global CTIS	Before February 2020, were you working for pay, or doing any kind of business, farming, or other activity to earn money?	Yes<br>No
COVID-19 High Frequency Phone Survey	Were you working before mid-March?*	Yes<br>No
COVID-19 High Frequency Phone Survey	Last week, that is from Monday up to Sunday, did you do any work for pay, do any kind of business, farming or other activity to generate income, even if only for one hour?*	Yes<br>No
Survey on Gender Equality at Home	As a result of coronavirus (COVID-19) pandemic, did you personally experience any of the following? Select all that apply<br>1)School was cancelled or reduced<br>2)Migrated to different geographical area<br>3)Difficulty accessing medical or hygiene supplies<br>4)Longer wait times to visit doctors/seek medical care<br>5)Lost a job<br>6)Lost access to/could not use public transport<br>7)Unable to perform usual personal care/health routines<br>8)Unable to seek medical care<br>9)Isolate or follow a quarantine order<br>10)None of the above<br>11)Other	Yes<br>No
Survey on Gender Equality at Home	Last week, did you do any work for pay, do any kind business, farming or other activity to generate income, even if only for one hour?	Yes<br>No
COVID-19 Behavior Tracker 2020 - YouGov	Employment status	Full time employment<br>Part time employment<br>Full time student<br>Retired<br>Unemployed<br>Not working<br>Other
COVID-19 Behavior Tracker 2020 - YouGov	Have you lost a job since 1st February 2020? If yes, when was that? If more than once, choose the most recent.	No<br>Yes, within the past 2 weeks<br>Yes, between 2 weeks and 2 months ago<br>Yes, more than two months ago but since 1st February 2020
COVID-19 Health Services Disruption Survey 2020 and 2021	Last week, did you do any work for pay, do any kind of business, farming or other activity to generate income, even if only for one hour? (SELECT_ONE)	Yes<br>No
COVID-19 Health Services Disruption Survey 2020	Were you working during December-February? (SELECT_ONE)	Yes<br>No
COVID-19 Health Services Disruption Survey 2021	Were you working during the 3 months before the COVID-19 pandemic began to affect your local area? (SELECT_ONE)	Yes<br>No

*Slight variation in the wording of the question per country

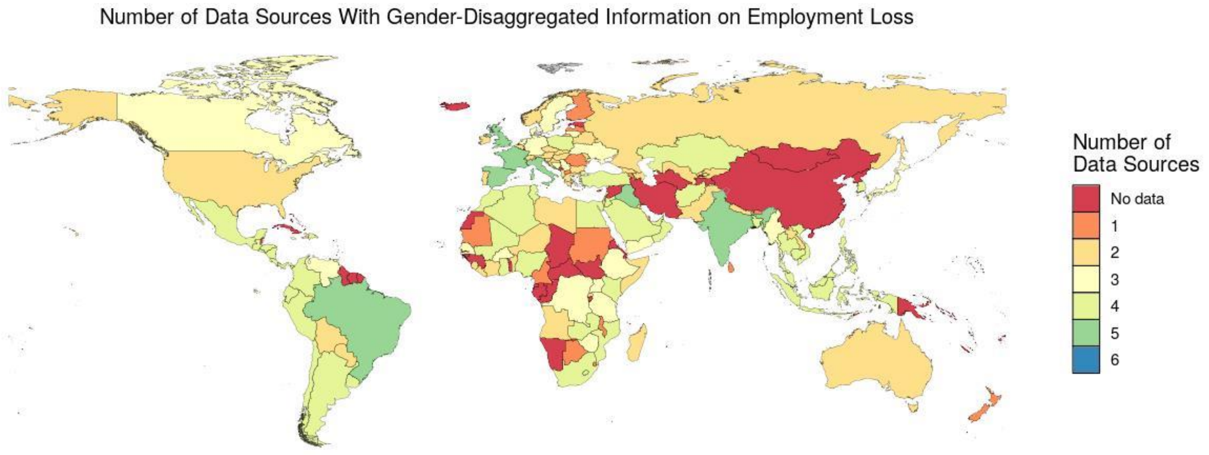

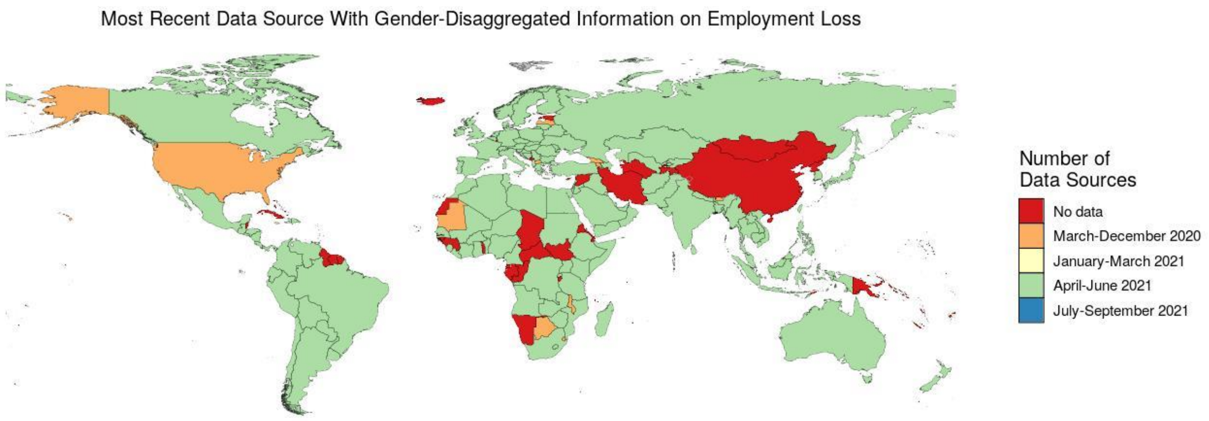

Although the *COVID-19 Rapid Gender Assessment Survey 2020* and the *Research for Effective Covid-19 Response Panel Survey 2020 (RECOVR)* provided data on employment loss, we are not using it in our analysis because it wasn't clear what denominator is being used to compute this variable (whether currently not working or all surveyed).

Data were processed to make the employment loss indicator consistent across sources. Employment loss was calculated using two questions from the *UMD Global CTIS* (cross sectional time series), *COVID-19 High Frequency Survey* (cross sectional), *COVID-19 Health Services Disruption Survey* 2020 and 2021 (two repeated cross-sectionals), *Survey on Gender Equality at Home* (cross sectional), and *COVID-19 Behavior Tracker 2020*. Employment loss was calculated among those who worked before the pandemic, and it was defined as working before the pandemic but not currently working now. Cross-sectional proportions were calculated by collapsing across location and gender. Monthly proportions were calculated using data from the *UMD Global CTIS* collapsed across location and gender using their respective weights.

For the *Global COVID-19 Trends and Impacts Survey*, respondents who worked before February 2020 but were not currently working were included in the numerator and the denominator. All other currently working respondents were also included in the denominator, and we assumed that current employment indicated working before the pandemic as well. The denominator, in other words, included all respondents who worked before the pandemic, irrespective of their current employment status.

For both *COVID-19 Heath Services Disruption survey* and *COVID-19 High Frequency Phone Survey*, respondents who were working before the pandemic but were not working now were defined as 1 in the numerator, and those who were currently working were defined as 0.

In the case of the *Survey on Gender Equality at Home*, employment loss was defined as 1 if the individual selected "Lost a job" as a result of the COVID-19 pandemic, and 0 otherwise. When calculating the proportion of those who lost their job, the denominator for this variable was respondents currently working and individuals currently not working who lost their job.

Employment loss in *COVID-19 Behavior Tracker 2020 – YouGov* was defined as 1 if the individual selected "Yes, within the past 2 weeks", "Yes, between 2 weeks and 2 months ago" or "Yes, more than two months ago but since 1st February 2020" on the question that asked whether they lost their job, and they were currently not working. Individuals were defined as 0 when they answered "No" to the question if they lost their job and were currently working.

Although the *COVID-19 Rapid Gender Assessment Survey 2020* and the *Research for Effective Covid-19 Response Panel Survey 2020 (RECOVR)* provided data on employment loss, we are not using it in our analysis because it wasn't clear what denominator is being used to compute this variable (whether currently not working or all surveyed).

Income loss

We used the sources listed in Table 9 to compute the proportion of income loss since the COVID-19 pandemic started. All surveys listed below sampled men and women. The *COVID-19 Rapid Gender Assessment Survey* in 2020, and the *COVID-19 Behavior Tracker 2020 – YouGov* sampled 18 years and older. The *COVID-19 Health Services Disruption Survey 2020* interviewed individuals 16 years and older.

Data was processed to combine the different ways in which surveys captured income loss since the pandemic. For surveys that directly asked how the respondent's income changed in comparison to pre-pandemic times, all answers that referred to decrease in income were coded as 1 in our income loss indicator. In contrast, answers that referred to increase in income or no change in income since the pandemic were coded as 0 in our income loss indicator. In surveys where the respondents were asked to recall the amount of income they made in the previous week and in typical week before the pandemic, we first calculated the absolute difference in the earned income from the current time period minus the same time period before the pandemic. If the change of income was positive, we coded our income loss indicator as 1. If the change of income was 0 or negative, we coded our income loss indicator as 0. The indicator for *COVID-19 Rapid Gender Assessment Survey* was pre-processed by UN women as 1 when the individual reported loss of income due to the pandemic and 0 if not.

For all indicators, the denominator was everyone surveyed who reported being currently working.

All survey sources for this indicator collected data for one point in time, the proportions of income loss per source are collapsed at the location and gender levels. Weighted proportions were estimated for all sources.

Although the *Measuring COVID-19 Impacts, Mitigation and Awareness Survey 2020* collected data on income loss, we decided not to use this source in our analysis given errors with the currently employed variable available in the raw data available to us.

Table 9. Input data for the income loss indicator.

Data Source	Question	Response options
COVID-19 Rapid Gender Assessment Survey 2020	Since the outbreak of COVID 19, did you have any changes in your personal income?	No changes<br>Increase of income<br>Decrease of income<br>I have lost all my income
COVID-19 Behavior Tracker 2020 - YouGov	Employment status	Full time employment<br>Part time employment<br>Full time student<br>Retired<br>Unemployed<br>Not working<br>Other
COVID-19 Behavior Tracker 2020 - YouGov	Have you experienced a large (more than 25%) reduction in earnings/income since 1st February 2020? If yes, when did that begin or happen?	No<br>Yes, that started within the past 2 weeks<br>Yes, between 2 weeks and 2 months ago<br>Yes, more than two months ago but since 1st February 2020
Research for Effective Covid-19 Response Panel Survey 2020 (RECOVR)	During the past 7 days, did you earn more, the same, or less pay than you did in a typical week before mid-March (I.e., before government closed the schools)?	More<br>Same<br>Less<br>None<br>Refused to answer
COVID-19 Health Services Disruption Survey 2020	Last week, did you do any work for pay, do any kind of business, farming or other activity to generate income, even if only for one hour? (SELECT_ONE)	Yes<br>No
COVID-19 Health Services Disruption Survey 2020	During December-February, what was your personal average income in a month?	Number
COVID-19 Health Services Disruption Survey 2020	Since March, what was your personal average income in a month?	Number

Number of Data Sources With Gender-Disaggregated Information on Income Loss

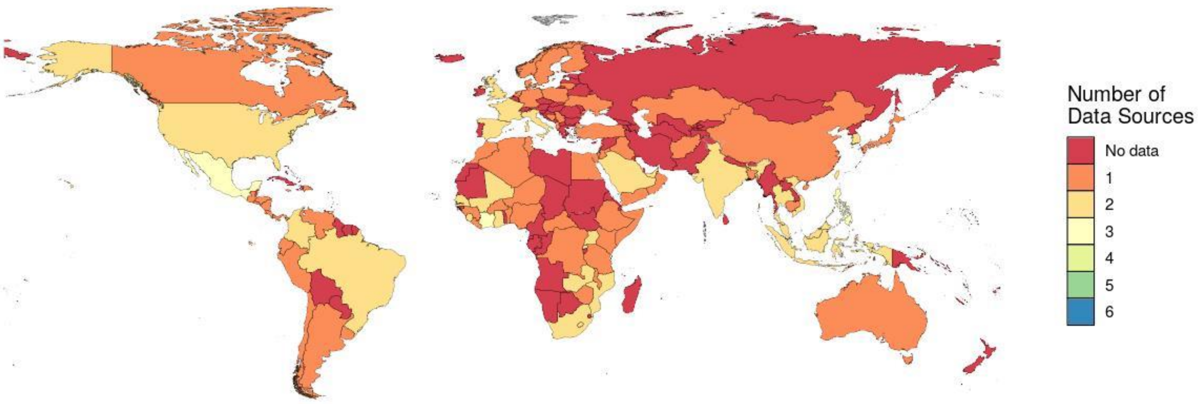

Most Recent Data Source With Gender-Disaggregated Information on Income Loss

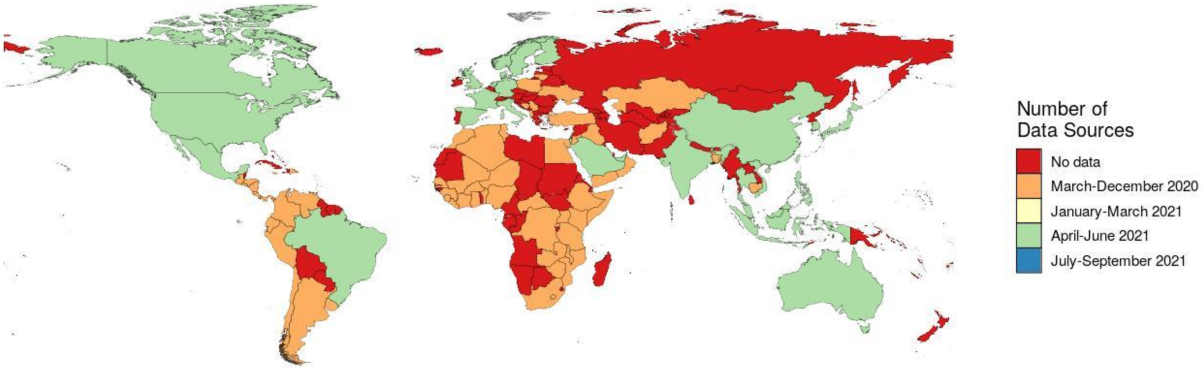

Increase in chores

We used the sources listed in Table 10 to compute the proportion of individuals who are spending more time in household related chores since the pandemic started. Both sources sample men and women 18 years and older.

Table 10. Input data for the increase in chores indicator.

Data Source	Question	Response options	Countries
Survey on Gender Equality at Home	How did the amount of time you spend on household chores change during the coronavirus (COVID-19) pandemic compared to before? Select one	Increased<br>Stayed the same<br>Decreased<br>Not applicable	All
COVID-19 Rapid Gender Assessment Survey 2020	As a result of COVID19, has the number of hours devoted to the following activities changed?<br><br>1Cooking and serving meals]<br>2)Cleaning [e.g. clothes / household]<br>3Decoration, repair and household management (e.g. paying bills)<br>4)Shopping for my household /family members<br>5)Collecting water/fuel<br>6)Minding children while doing other tasks (e.g. paid work)<br>7)Playing with, talking to and reading to children<br>8)Instructing, teaching, training children<br>9)Caring for children, including feeding, cleaning physical care<br>10)Assisting elderly / sick / disabled adults with medical care, feeding, cleaning, physical care<br>11) Assisting elderly / sick / disabled adults with administration and accounts<br>12)Affective/emotional support for adult family members<br>13)Pet care	Do not usually do it<br>Increased<br>Unchanged<br>Decreased<br>DON'T KNOW<br>REFUSED	Asia and the Pacific, similar for East Southern Africa, and Europe and Central Asia
COVID-19 Rapid Gender Assessment Survey 2020	Have there been any changes in the time you usually spend on:<br>Q26. Food and meal management and preparation?<br>Q27. Cleaning and maintaining own dwelling and surroundings?<br>Q28. Fetching water/collect food?<br>Q29. Shopping for own household and family members?	I don't do it<br>Didn't do it before but now I'm spending time on it<br>More time<br>Same time as before<br>Less time than before	West Central Africa
COVID-19 Rapid Gender Assessment Survey 2020	As a result of COVID-19, has the time devoted by you to the following activity changed?<br>Q7. Doing household chores (e.g. cooking food, cleaning, serving meals...etc)	Increased time spent<br>No change in time spent<br>Decreased time spent<br>I never do these activities	Arab states

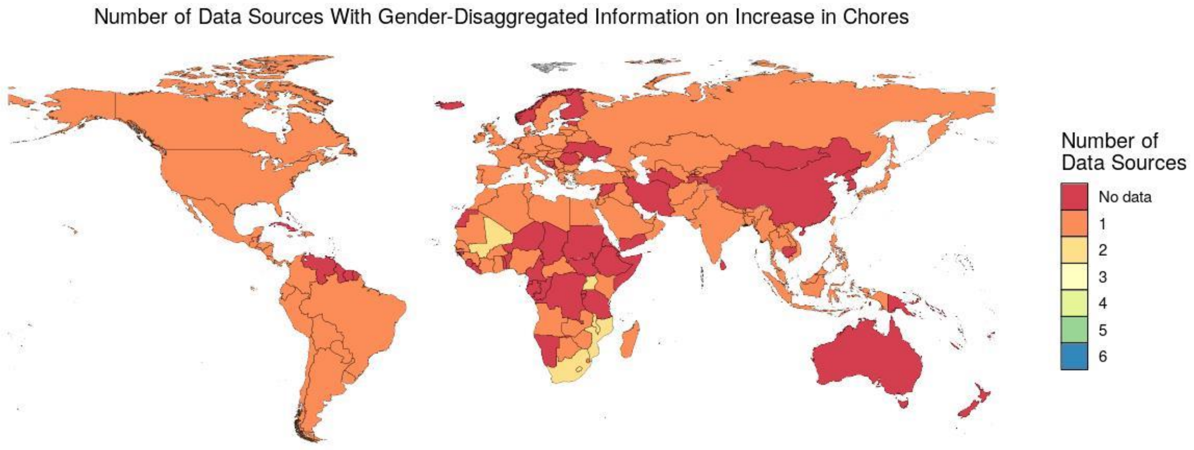

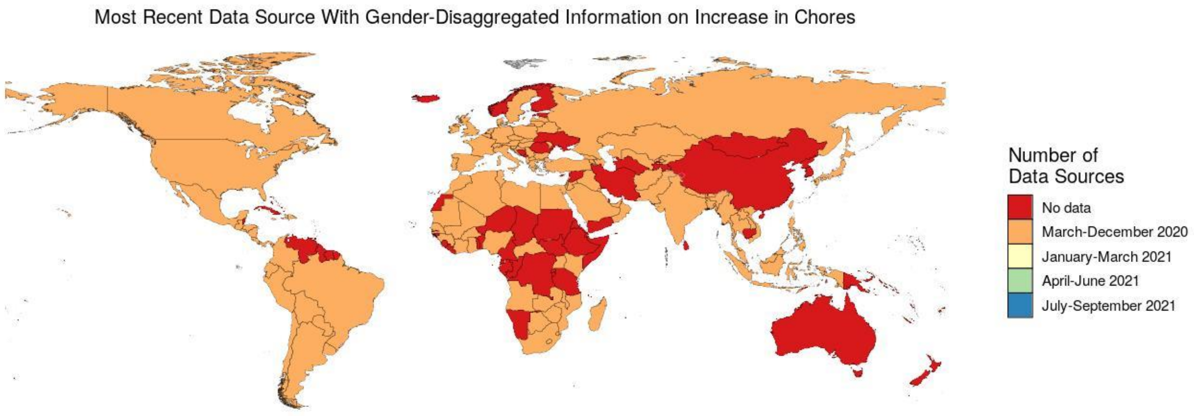

For the *Survey on Gender Equality at home*, the increase in chore indicators was denoted as 1 whenever the respondent answered “Increased”, and 0 if the respondent answered “Stayed the same” or “Decreased”. The indicator for *COVID-19 Rapid Gender Assessment Survey* was processed by UN women as 1 when the individual increased the time spent in at least one domestic activity and 0 if not.

All survey sources for this indicator collected data for one point in time. Weighted proportions were estimated for all sources at the location and gender levels.

Increase in care for others

We used the sources listed in Table 11 to compute the proportion of individuals who are spending more time caring for family members since the pandemic started. Both sources sample men and women 18 years and older.

Table 11. Input data for the increase in care for others indicator.

Data Source	Question	Response options	Countries
Survey on Gender Equality at Home	How has the amount of time you spend caring for your family members change during the coronavirus (COVID-19) pandemic compared to before? Select one	Increased<br>Stayed the same<br>Decreased<br>Not applicable	All
COVID-19 Rapid Gender Assessment Survey 2020	As a result of COVID19, has the number of hours devoted to the following activities changed?<br><br>1Cooking and serving meals]<br>2)Cleaning [e.g. clothes / household]<br>3Decoration, repair and household management (e.g. paying bills)<br>4)Shopping for my household /family members<br>5)Collecting water/fuel<br>6)Minding children while doing other tasks (e.g. paid work)<br>7)Playing with, talking to and reading to children<br>8)Instructing, teaching, trining children<br>9)Caring for children, including feeding, cleaning physical care<br>10)Assisting elderly / sick / disabled adults with medical care, feeding, cleaning, physical care<br>11) Assisting elderly / sick / disabled adults with administration and accounts<br>12)Affective/emotional support for adult family members<br>13)Pet care	Do not usually do it<br>Increased<br>Unchanged<br>Decreased<br>DON'T KNOW<br>REFUSED	Asia and the Pacific, similar for East Southern Africa, and Europe and Central Asia
COVID-19 Rapid Gender Assessment Survey 2020	Have there been any changes in the time you usually spend on:<br>Q30. Caring for children 0-4 years?<br>Q31. Caring for children 5-17 years?<br>Q32. Instructing, teaching, training children?<br>Q33. Caring for elderly and caring for a sick family/household member?	I don't do it<br>Didn't do it before but now I'm spending time on it<br>More time<br>Same time as before<br>Less time than before	West Central Africa
COVID-19 Rapid Gender Assessment Survey 2020	As a result of COVID-19, has the time devoted by you to the following activity changed?<br>Q8. Assisting children in doing homework?<br>Q9. Caring for children, including feeding, cleaning, physical care?<br>Q10. Caring for the elderly, disabled or sick members of the family?	Increased time spent<br>No change in time spent<br>Decreased time spent<br>I never do these activities	Arab states

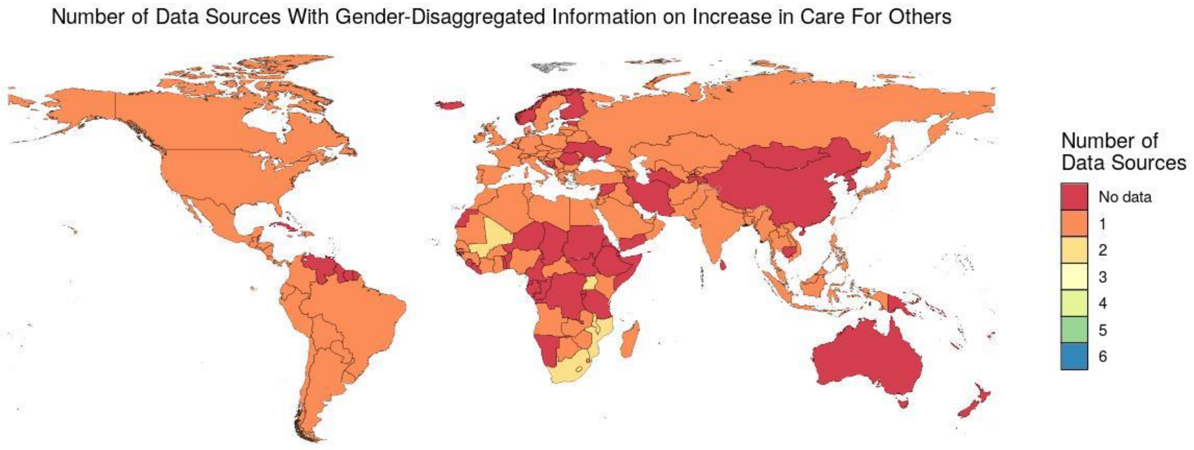

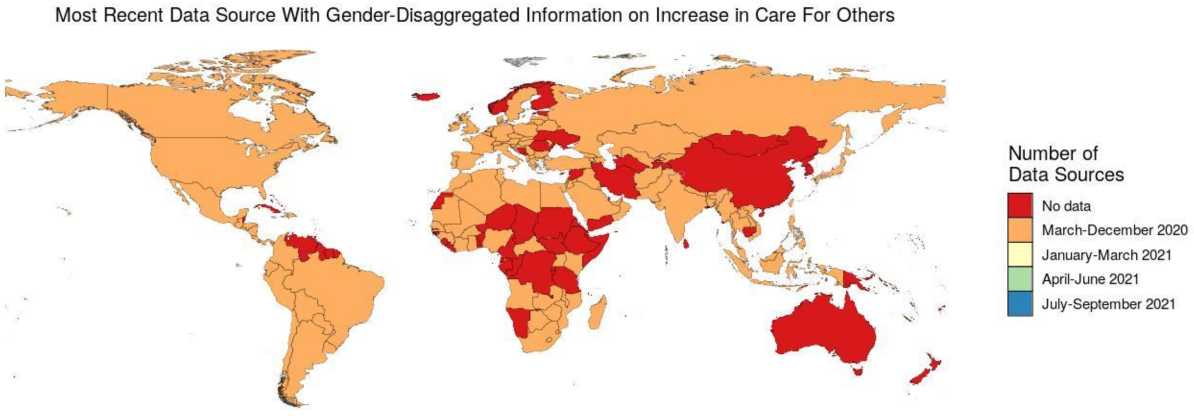

For the *Survey on Gender Equality at home*, the increase in care for others indicators was denoted as 1 whenever the respondent answered "Increased", and 0 if the respondent answered "Stayed the same" or "Decreased". The indicator for *COVID-19 Rapid Gender Assessment Survey* was processed by UN women as 1 when the individual increased the time spent in at least one childcare activity or one adult care activity

All survey sources for this indicator collected data for one point in time. Weighted proportions were estimated for all sources at the location and gender levels.

Not working to care for others

We used the sources listed in Table 12 to compute the proportion of individuals that left their job since the pandemic started to take care for someone. The *UMD Global CTIS* sampled men and women 18 years old and above. The *COVID-19 Health Services Disruption Survey* for 2020 and 2021 sampled men and women 16 years old and above.

Table 12. Input data for the not working to care for others indicator.

Data Source	Question	Response options
UMD Global CTIS	Why did you stop working?	My employer closed for coronavirus-related reasons<br>My employer closed for another reason<br>I was laid off or furloughed<br>I am a seasonal worker<br>I was ill or quarantined<br>I needed to care for someone<br>Other
COVID-19 Health Services Disruption Survey 2020 and 2021	What was the main reason you stopped working since the start of the COVID-19 pandemic? (SELECT_ONE)	Business / office closed<br>Laid off while business continues<br>Furlough (temporarily laid off)<br>Vacation<br>Ill or quarantined<br>Need to care for ill relative<br>Seasonal worker<br>Retired<br>Not able to go to work due to government mandated movement restrictions<br>To avoid exposure to COVID-19<br>Other

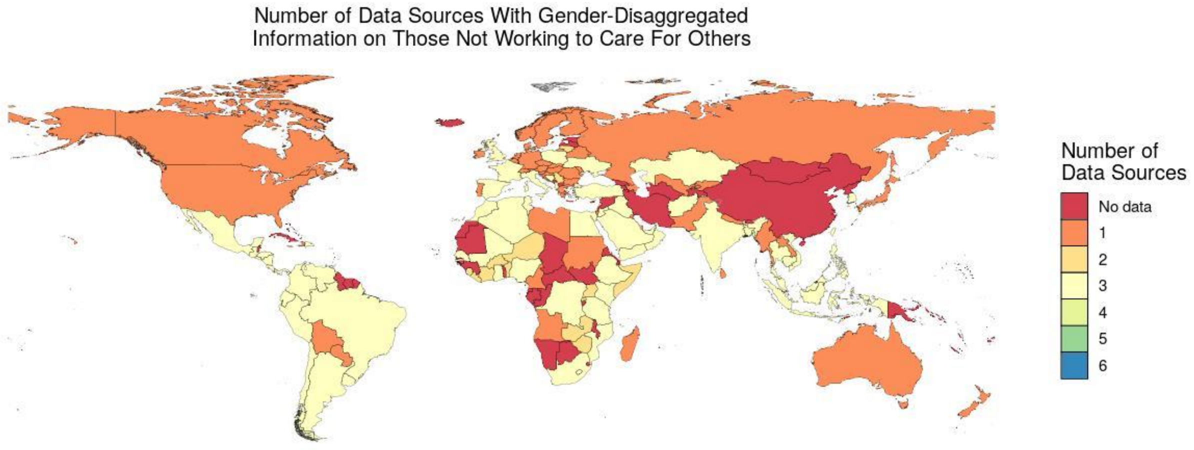

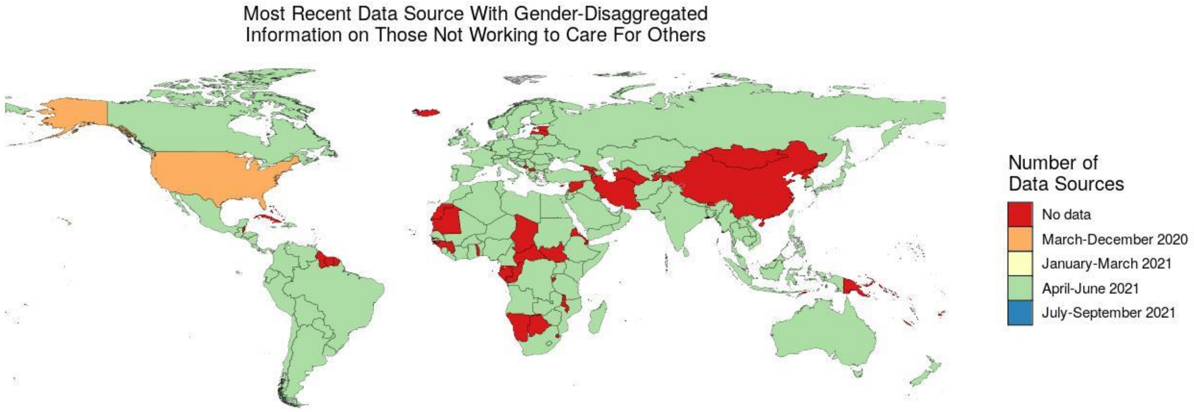

At the individual level, data was processed to create a binary indicator where 1 represents whether an individual that had a job before COVID-19 decided to leave her job to take care of someone for the *UMD Global CTIS*, and 1 if she left her job to care for ill relative in the *COVID-19 Health Services Disruption Surveys*. All other answer options for both surveys were defined as 0. In other words, the denominator for the indicator are individuals who are currently not working. *UMD Global CTIS* collects daily data which was aggregated at the location, month and gender to estimate weighted proportions. The weights used were provided by the survey. Only *UMD Global CTIS* was used for the mixed effects regression given sample size issues with the *COVID-19 Health Services Disruption Surveys* for the indicator and covariates of interest.

3.4 Education

School drop out

We used the sources listed in Table 13 to compute the proportion of learners no longer in school not due to graduation or school break among all learners in school prior to the pandemic. The *Premise Education COVID-19 Health Services Disruption Survey 2021* is a learner-level dataset that asked adults premise contributors answer questions about the schooling of children, adolescents, and adults in their household. Observations in this dataset refer to all people enrolled in school at any level in February 2020 in households of premise contributors.

Table 13. Input data for the school dropout indicator.

Data Source	Question	Response options
Premise Education COVID-19 Health Services Disruption Survey 2021	How many individuals in your household were going to school or university before the global COVID-19 pandemic started in March 2020-April 2020? This includes school for children, or university for adults	Numeric response
	For each person in your household who was enrolled in school or university in February 2020, what gender are they?	Male | Female | Non-binary | Decline to respond
	For each person, which of the following best describes their current schooling situation (or their schooling situation in March-April 2021, if their school session has ended for the year):	In person class | Learning online or remotely at home | Mix of in-person and online/remote | Not currently in school
	For each person, which of the following best describes why they are no longer in school?	School is closed due to COVID-19 | School is closed due to break/vacation | Already graduated | School is too far away | Cannot afford to go to school | School does not have clean, safe buildings and bathrooms | Had to leave school to work | Had to leave school to care for a family member | Had to leave school for other reason

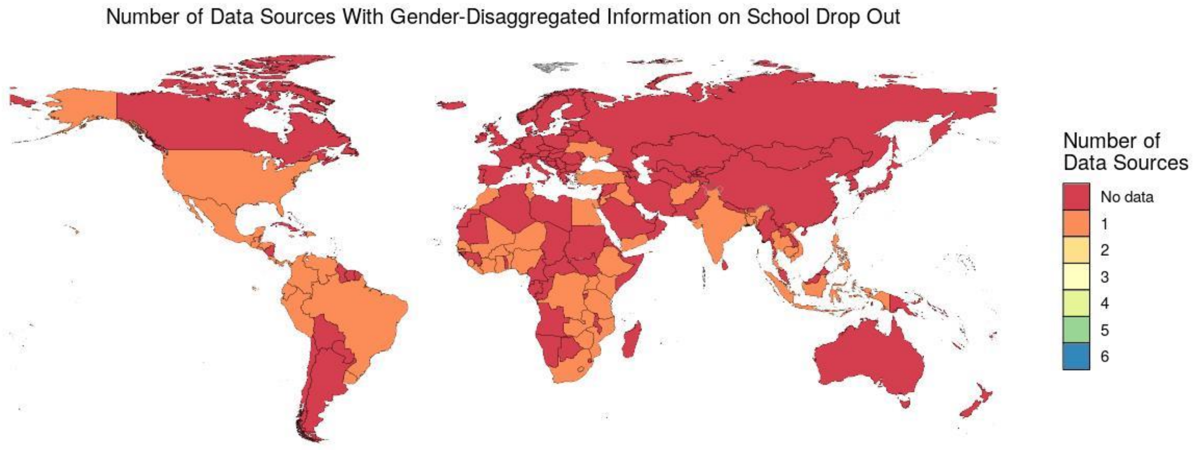

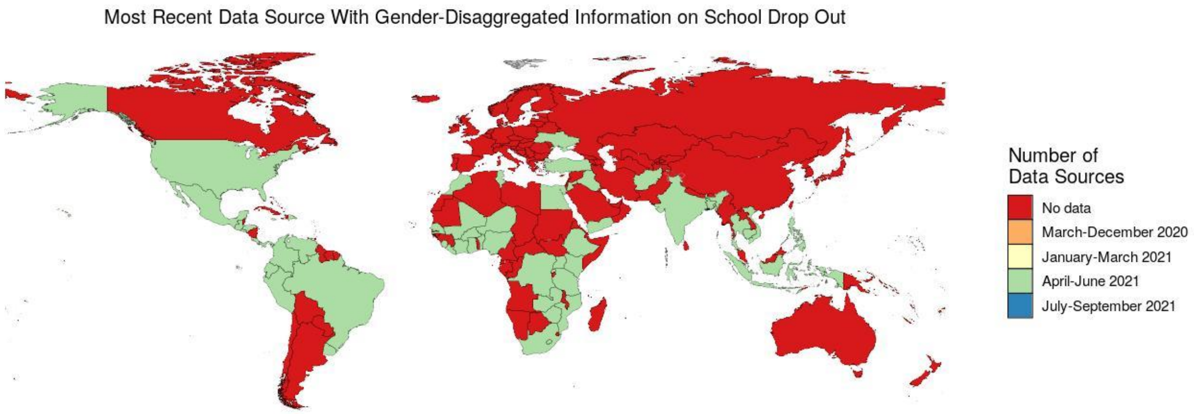

Data were processed such that any pre-pandemic learner whose current learning situation was reported as being “not currently in school” and the reason provided was **not** “already graduated” was coded as a positive response (1) for the school dropout indicator. The denominator was all pre-pandemic learners (i.e. those identified by the respondent as having been going to school before the pandemic).

These data are cross-sectional in nature and available for 2021 only, thus cross-sectional proportions were calculated by collapsing across location and gender. Survey weights were not provided *from Premise Education COVID-19 Health Services Disruption Survey 2021*; therefore, proportions from this source are unweighted. Explanatory predictors for school dropout were explored using individual-level data and the standard logistic regression framework described elsewhere in the appendix.

Adequate Remote Learning

We used the sources listed in Table 14 to compute the proportion of learners with good internet access among all online learners during the pandemic. The *Premise Education COVID-19 Health Services Disruption Survey 2021* is a learner-level dataset that asked adults premise contributors answer questions about the schooling of children, adolescents, and adults in their household. Observations in this dataset refer to all people enrolled in school at any level in February 2020 in households of premise contributors.

Table 14. Input data for the adequate remote learning indicator.

Data Source	Question	Response options
Premise Education COVID-19 Health Services Disruption Survey 2021	How many individuals in your household were going to school or university before the global COVID-19 pandemic started in March 2020-April 2020? This includes school for children, or university for adults	Numeric response
	For each person in your household who was enrolled in school or university in February 2020, what gender are they?	Male | Female | Non-binary | Decline to respond
	For each person, which of the following best describes their current schooling situation (or their schooling situation in March-April 2021, if their school session has ended for the year)?	In person class | Learning online or remotely at home | Mix of in-person and online/remote | Not currently in school
	For each person who is participating in online or remote school, which of the following best describes how they are learning online or remotely (when school is in session)?	Online (Using computer/tablet) | Online (Using smartphone) | Paper materials sent home | Classes on television | Classes on radio
	For each person, which of the following best describes how often they have access to a working internet connection and a working device to be able to learn (when school is in session)?	Never (0 days per week) | Rarely (1-2 days per week) | Usually (3-4 days per week) | Most of the time (5-6 days per week) | Always (7 days per week)

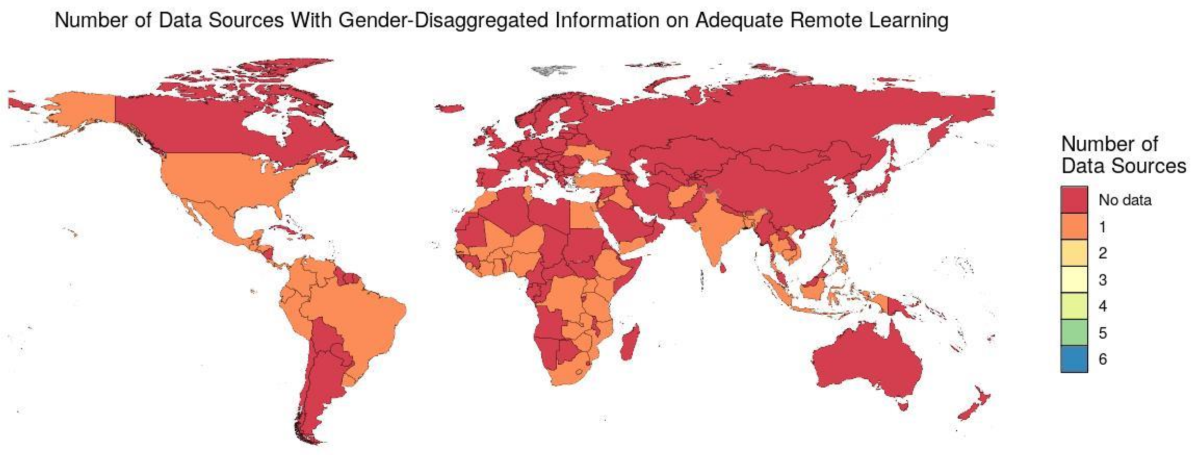

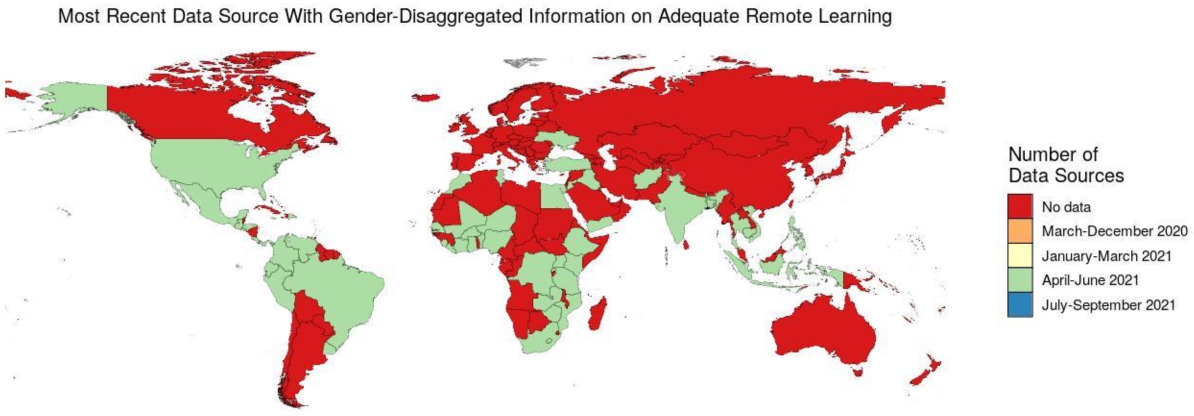

Respondents were only asked about a learner's internet connection and working device access if they first reported that a learner was learning fully or partially online/remote and using a computer/tablet or smartphone to learn. Data were processed such that any pre-pandemic learner whose current learning situation was online/remote through the use of a computer/tablet/smartphone and had access to a working internet connection and working device "Most of the time" or "Always" was coded as a positive response (1) for the adequate remote learning indicator. The denominator was all learners learning online/remotely and using a computer/tablet/smartphone to learn online.

These data are cross-sectional in nature and available for 2021 only, thus cross-sectional proportions were calculated by collapsing across location and gender. Survey weights were not provided *from Premise Education COVID-19 Health Services Disruption Survey 2021*; therefore, proportions from this source are unweighted. Explanatory predictors for adequate remote learning were explored using individual-level data and the standard logistic regression framework described elsewhere in the appendix.

3.5 Safety at Home and in the Community

Feeling Unsafe at Home

We used the sources listed in Table 15 to compute the proportion of respondents who reported feeling unsafe at home during the COVID-19 pandemic among all respondents. *Survey on Gender Equality at Home* and *COVID-19 Rapid Gender Assessment Surveys* sampled men and women 18 years and older.

Table 15. Input data for the feeling unsafe at home indicator.

Data Source	Question	Response options
Survey on Gender Equality at Home	There are times when I feel uncomfortable or even unsafe in my house	Strongly Agree | Agree | Neither | Disagree | Strongly Disagree
COVID-19 Rapid Gender Assessment Survey (RGA) 2020 (Cote d'Ivoire, Central African Republic, Guinea, Mali, Senegal)	Do you feel safe from violence in your home?	Yes | No
COVID-19 Rapid Gender Assessment Survey (RGA) 2020 (Ethiopia, Kenya, Mozambique, South Africa, Eswatini, Malawi)	Do you feel safe in your home?	Yes | No
COVID-19 Rapid Gender Assessment Survey (RGA) 2020 (Colombia, Mexico)	Since the beginning of the COVID-19 pandemic, have you felt safe in your home?	Yes | No
COVID-19 Rapid Gender Assessment Survey (RGA) 2020 (Egypt, Iraq, Jordan, Lebanon, Libya, Morocco, Palestine, Tunisia, Yemen)	At the moment, do you feel safe in your home?	Yes | No

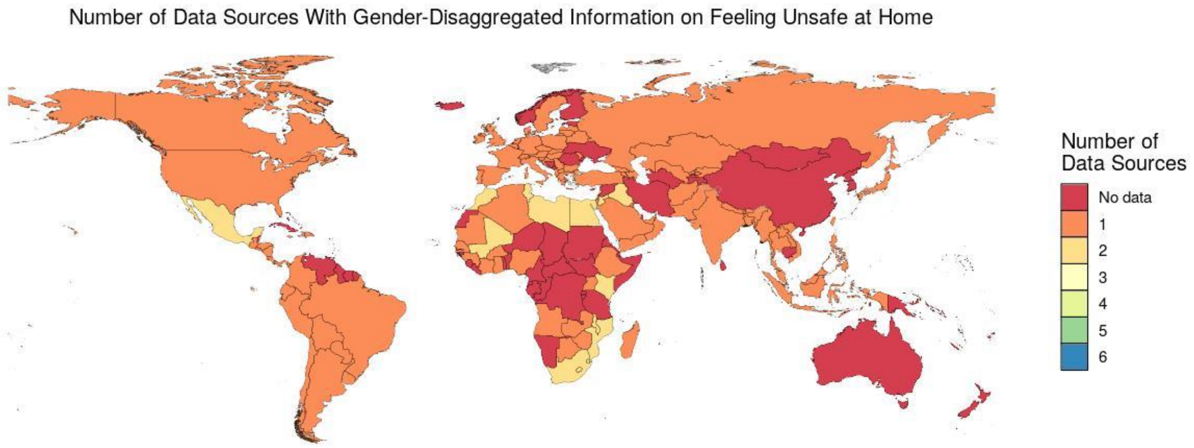

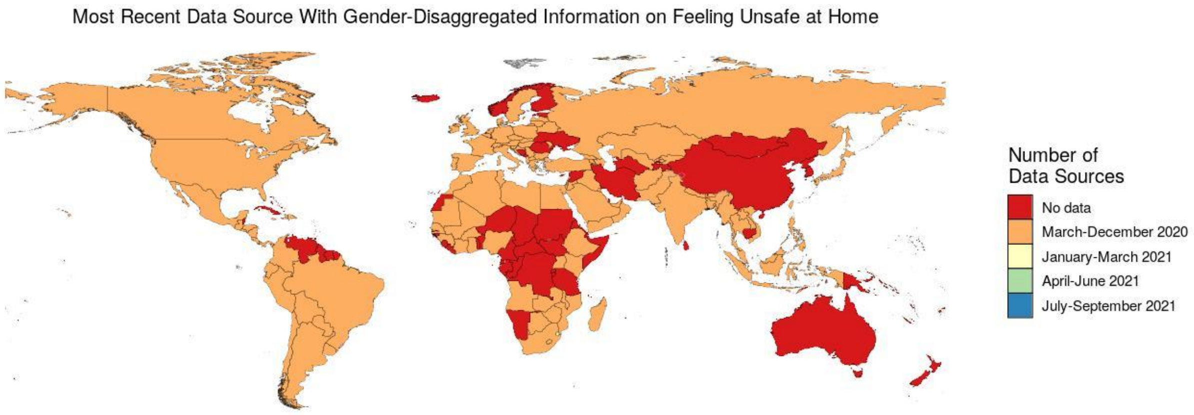

Data were processed to ensure that the indicator was comparable across data sources. Data from the *Survey on Gender Equality at Home* were re-coded to binary format such that answering “strongly agree” or “agree” equated to a positive response (1) to feeling unsafe at home whereas answering “neither,” “disagree,” or “strongly disagree” equated to a negative response (0). For each data source, the denominator of the indicator was all respondents with a non-NA answer to the question listed.

Each source provided cross-sectional data only, thus cross-sectional proportions were calculated per source by collapsing across location and gender using weights provided by the respective survey. Cross-sectional proportions were only created for countries with a minimum of 30 observations per location and gender. Explanatory predictors for feeling unsafe at home were explored using individual level data from each source and the standard logistic regression framework described elsewhere in the appendix.

Perception of GBV Increase

We used the sources listed in Table 16 to compute the proportion of respondents who reported a perception that household or partner violence had increased in their community since the start of the COVID-19 pandemic among all respondents. *Premise Women's Health COVID-19 Health Services Disruption Survey 2021* sampled women 16 years and older, and *COVID-19 Rapid Gender Assessment Surveys* sampled men and women 18 years and older.

Data were processed to ensure that the indicator was comparable across data sources. Thus, data from each source was re-coded to equate to a binary indicator. For *Premise Women's Health COVID-19 Health Services Disruption Survey 2021*, answer options of “Don’t know” or “Decline to respond” were re-coded as NAs. Respondents who answered “More common than before the pandemic” were coded as positive responses (1) whereas all other answer options were coded as negative responses (0s). For the *COVID-19 Rapid Gender Assessment Surveys*, respondents who answered “Yes” or “Yes – increased” (depending on survey country) were coded as positive responses (1s) and all other options coded as negative responses (0s). For both data sources, the denominator of the indicator was all respondents with a non-missing answer to the question listed.

Each source provided cross-sectional data only, thus cross-sectional proportions were calculated per source by collapsing across location and gender using weights provided by the respective survey, when available. Survey weights were provided by the *COVID-19 Rapid Gender Assessment Surveys* but not from *Premise Women's Health COVID-19 Health Services Disruption Survey 2021*; therefore, proportions from this source are unweighted. Cross-sectional proportions were only created for countries with a minimum of 30 observations per location and gender. Explanatory predictors for perception of GBV increase were explored using individual level data from each source and the standard logistic regression framework described elsewhere in the appendix.

Table 16. Input data for the perception of GBV increase indicator.

Data Source	Question	Response options
Premise Women's Health COVID-19 Health Services Disruption Survey 2021	Since the start of the global COVID-19 pandemic in March/April 2020, would you say this sort of violence* has become more common, less common, or stayed the same?<br><br>*defined in previous question as, "violence from an intimate partner"	More common than before the pandemic | Less common than before the pandemic | About the same as before the pandemic | Don't know | Decline to respond
COVID-19 Rapid Gender Assessment Survey (RGA) 2020 (Mozambique, Uganda, Kenya, South Africa, Eswatini, Malawi)	Do you think gender-based violence in [country] has changed since the COVID-19 state of emergency started? If yes, how did it change?	Yes – increased | No – stayed the same | Yes – decreased
COVID-19 Rapid Gender Assessment Survey (RGA) 2020 (Cote d'Ivoire, Guinea, Senegal, Mali, Central African Republic)	Are you aware of any increase in violence in the households of your community since COVID-19 forced people to stay at home more?	Yes | No
COVID-19 Rapid Gender Assessment Survey (RGA) 2020 (Albania, Armenia, Azerbaijan, Bosnia and Herzegovina, Belarus, Serbia, North Macedonia, Georgia, Turkey, Moldova)	Have you felt/heard about increase of domestic violence since the spread of COVID-19?	Yes | No
COVID-19 Rapid Gender Assessment Survey (RGA) 2020 (Egypt, Iraq, Jordan, Lebanon, Libya, Morocco, Palestine, Tunisia, Yemen)	Have you witnessed, or do you know a woman who has experienced domestic violence by her spouse since the lockdown?	Yes | No

Number of Data Sources With Gender-Disaggregated Information on Perceptions of GBV Increase

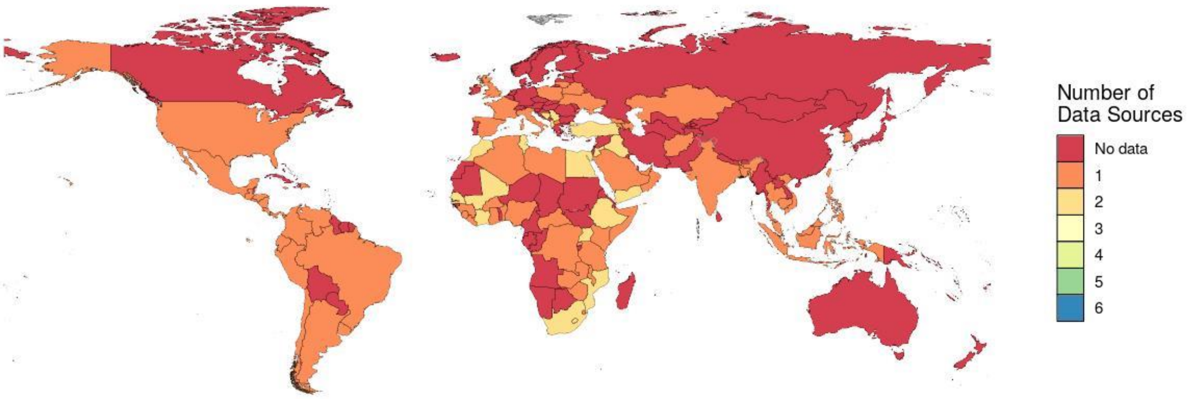

Most Recent Data Source With Gender-Disaggregated Information on Perceptions of GBV Increase

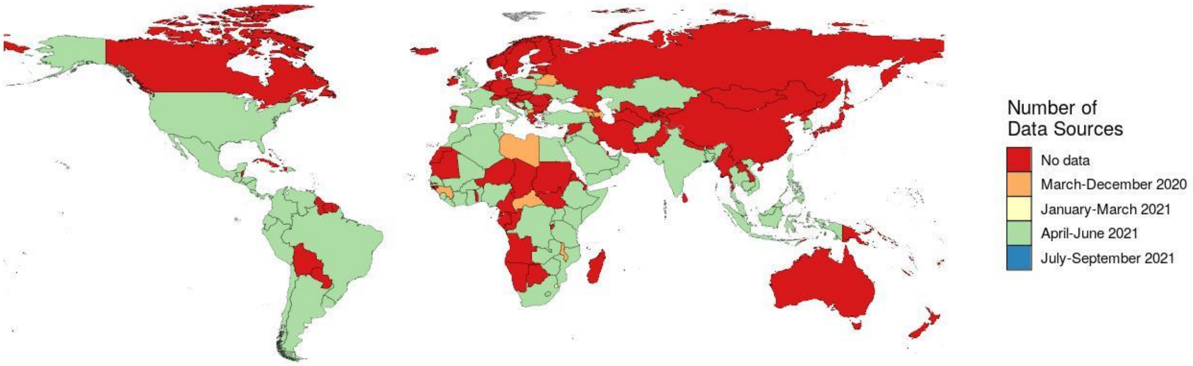

4. Methods

4.1 Time Series Modeling Overview

For eight indicators with input data available across multiple time points since March 2020 (vaccine hesitancy, fully vaccinated, any disruption in healthcare, disruption in medication access, disruption in health products access, disruption in preventative care, employment loss, and not working to care for others), we employed a time series modeling approach to produce time trends and investigate gender disparities for each indicator-age-month from December 2020 to September 2021 for the vaccine indicators and from March 2020 to September 2021 for the remaining outcomes. These models draw on 15 surveys with information collected during the COVID-19 pandemic. In the first stage, we model each indicator and gender using a mixed effects linear prior fit using all available data sources. We then use Gaussian process regression to estimate a complete time series for each indicator-gender and account for uncertainty in input data and the modeling process.

Mixed Effects Regression

Logistic mixed effects regression models were fit on each indicator-gender. Data for each indicator available from multiple points in time was first collapsed by country, gender, and month to create indicator proportions, using survey weights when available. Separately for each gender *g*, age *a*, and indicator of interest *i*, trends by month *m* and country *c* were modelled, $p_{c,g,m,i}$ was estimated:

$$logit(p_{c,g,m,i}) = \beta_{g,i} + \beta_{1_{c,g,i}} month + \alpha_{g,m,i,c} \qquad (1)$$

Where:

- $\beta_{g,i}$ is an indicator and gender specific global intercept
- $\beta_{1_{c,g,i}}$is an indicator and gender specific secular trend
- $\alpha_{g,m,i,c,}$ is a time series-specific random intercept

Additionally, gender-indicator specific fixed intercepts on age group were included for models that had input data that were age-specific (vaccine hesitancy). The aforementioned mixed effect models were then used to predict complete time series of $p_{c,s,m,i}$ for all locations-indicator pairs for which input data were available.

Gaussian Process Regression

Gaussian process regression (GPR) was used to ensure final model results are consistent with input data and incorporate model and data uncertainty to produce uncertainty intervals. GPR has been used extensively as a data synthesis tool. GPR uses a covariance function to smooth the residuals from the mixed effects regression model, taking into account the uncertainty in each data point. GPR also synthesises both data and model uncertainty, in order to produce estimate uncertainty intervals. GPR

assumes that the trend in the underlying data follows a Gaussian process, which is defined using a mean function $m(\cdot)$ and a covariance function $Cov(\cdot)$. Therefore, separately for each Q quantity being estimated, the location-gender-month specific outcome measures are defined:

$$logit(y_{Q,L,S,C,A}) = g_{Q,L,S,A,Y} + \epsilon_{Q,L,S,A,Y} \qquad (2.1)$$

Where the error term is normally distributed:

$$\epsilon_{Q,L,S,A,Y} = Normal(0, \sigma_p^2) \qquad (2.2)$$

The error variance, σ_p^2 is composed of the squared standard error of the observed data point, as well as the prediction errors from the mixed effects regression process. The mean function of the model is defined as the mixed effects regression predictions, as detailed above. The covariance function of the model is derived using a Matérn covariance function, consistent with prior applications of GPR:

$$M(y, y') = \sigma^2 \frac{2^{1-\nu}}{\Gamma(\nu)} \left(\frac{d(y,y')\sqrt{2\nu}}{l}\right)^{\nu} \mathrm{K}\nu \left(\frac{d(y,y')\sqrt{2\nu}}{l}\right) \qquad (2.3)$$

Where $d(\cdot)$ is a distance function, σ^2 is the marginal variance, ν is a smoothness hyper parameter defining the differentiability of the function, l is a link-scale parameter approximately equivalent to the number of months at which two points are no longer correlated, K_ν is the Bessel function, and $\Gamma(\cdot)$ is the gamma function. Similar to previous applications of GPR, we approximate σ_p^2 as the location and gender-specific residual from the mean function, set ν to 2, and l to 4, to reflect the inherent smoothness of indicator trends overtime.

4.2 Cross-sectional Data Synthesis

For eight indicators (disruption in reproductive health, income loss, increase in chores, increase in care for others, school dropout, adequate remote learning, perception of GBV increase, feeling unsafe at home) with input data available at only one or two points in time, we calculated indicator proportions across input data using bootstrapping methods. Data for each indicator were collapsed into proportions by country, gender, and source. Country-gender-indicator specific proportions were logit-transformed and point estimates and sample size used to sample 1000 draws, constrained between zero and one. Using country-gender specific population weights, draws were then aggregated to the world regions and total levels. At the draw-level and for country, world regions, and total aggregates, indicator-gender point estimates and 95% UIs were calculated. Additionally, absolute and relative gaps between men and

women estimates were calculated per indicator at each of the country, super-region, and total aggregate levels:

$$absolute\ gap = (Woman\ estimate - Man\ estimate) * 100 \qquad (3.1)$$

$$relative\ gap = ((Woman\ estimate/Man\ estimate) - 1) * 100 \qquad (3.2)$$

4.3 Multivariate Regression Analyses

For 17 indicators (vaccine hesitancy, fully vaccinated, disruption in health products access, any disruption in health care, disruption in preventative care, disruption in medication access, disruption in reproductive health, school dropout, adequate remote learning, employment loss, income loss, not working to care for others, increase in care for others, increase in chores, feeling unsafe at home, perception of GBV increase) available in individual-level survey microdata, we employed a mixed effects logistic regression model to investigate associations between each indicator and gender, adjusting for geography, age, educational attainment, and urbanicity, when these factors were available from the data source. Survey weights provided from data sources were not included in the regression models, as the same variables used to create survey weights were accounted for in the model covariates.

Separately for each source available per indicator, the probability of each outcome, Y_i, was estimated:

$$logit(P(Y_i = 1 \mid X_i)) = \beta_0 + \beta_1 woman_i + \sum_{j=2}^{3} \beta_j I_{A[a]} + \beta_4 rural_i + \beta_5 some\ tertiary\ education_i + \sum_{k=6}^{18} \beta_k I_{T[t]} + \alpha_g \quad (4)$$

Where:

- $woman_i$ is a binary indicator of female gender
- $I_{A[a]}$ is a dummy variable indicating the specific age group A to which the observation belongs;
- $rural_i$ Is a binary indicator of residing in a rural area;
- $some\ tertiary\ education_i$ is a binary indicator of having greater than high school education; and
- $I_{T[t]}$ is a dummy variable indicating the time period $T$of data collection (only included for sources in which multiple time points were available in input data); and
- α_g is a random effect on location.

For each indicator and source, a model using standard covariate specifications and all available data was used for primary estimation, and exponentiated regression coefficient estimates and 95% uncertainty intervals presented in the main text (Figures 2, 3, 4, 5 and 6). For sources with multiple world regions represented in the input data, regressions models were run separately by world region to assess geographic variation in results. Region-specific regressions from certain sources were not always possible and were excluded due to low regional sample sizes or poor distribution of the dependent variable among covariate categories of interest. Exponentiated regression coefficient estimates and 95% uncertainty intervals from region-specific models are presented in the main text (Figures 2, 3, 4, 5 and 6).

For educational outcomes (school dropout and adequate remote learning), the probability of each outcome was estimated with an additional fixed effect for gender of child learner.

Information on respondent age was not available from the *Survey on Gender Equality at Home*. Accordingly, all models using this data source do not include a covariate for age.

Individual-level data from *UMD Global CTIS* and *Delphi US CTIS* was available from June 2020. To test the sensitivity of results over time, we separated all available data by time period and ran independent regression models on these subsets. For vaccine hesitancy and fully vaccinated, we separated data into two time-periods: (1) January 2021-March 2021 and (2) April 2021-September 2021. For employment loss, not working to care for others, any disruption in health care, disruption in preventative care, disruption in medication access, and disruption in health products access, we separated data into three time-periods: (1) June 2020-December 2020, (2) January 2021 – March 2021, and (3) April 2021-June 2021. Results using data from April 2021 through the most recent month of data available for the indicator are presented in the main text (Figures 2, 3, 4, 5 and 6). A comparison of estimations by time period per indicator are additionally presented in the appendix (Supplemental Figure 8).

Due to the limited overall sample size available from the Women's health module of the *COVID-19 Health Services Disruption Surveys*, we conducted a robustness check of the regression model for perception of GBV increase using this source by including only countries with a minimum of 30 observations in the model. We found similar results when limiting the input data by location-specific sample size versus using all input data, regardless of location-specific sample size.

Lastly, we investigated disruptions in reproductive healthcare beyond the main regression framework using more detailed demographic available from the *COVID-19 Rapid Gender Assessment Surveys*. Exponentiated regression coefficient estimates and 95% uncertainty intervals from these models are provided in Table S2.

5. Supplemental Tables and Figures

Supplemental Table S1. Total sample size and the percent women respondents for each indicator

Indicator	Data Source	Number of Respondents	% Women	Number of Countries
**Vaccine hesitancy and uptake**				
Vaccine hesitancy	UMD Global CTIS and Delphi US CTIS	13,456,672	49.9	196
Fully vaccinated	UMD Global CTIS and Delphi US CTIS	28,306,877	50.1	196
**Healthcare**				
Any disruption in health care	Survey on Gender Equality at Home	184,889	51.0	111
Any disruption in health care	COVID-19 Health Services Disruption Survey	67,836	32.5	76
Any disruption in health care	UMD Global CTIS	66,923	50.5	129
Disruption in preventative care	UMD Global CTIS	66,202	50.5	129
Disruption in medication access	UMD Global CTIS	66,387	50.5	129
Disruption health product access	Survey on Gender Equality at Home	184,889	51.0	111
Disruption health product access	UMD Global CTIS	66,058	50.5	129
Disruption health product access	COVID-19 Rapid Gender Assessment Survey	18,228	54.0	12
**Economic and work-related concerns**				
Employment loss	UMD Global CTIS and U.S. COVID-19 Trends and Impact Survey	2,552,288	47.8	188
Employment loss	Survey on Gender Equality at Home	104,217	47.3	105
Employment loss	COVID-19 Health Services Disruption Survey	47,576	30.2	76
Employment loss	COVID-19 High Frequency Phone Survey	11,113	43.8	7

Supplemental Table S1. Total sample size and the percent women respondents for each indicator

Indicator	Data Source	Number of Respondents	% Women	Number of Countries
Income loss	COVID-19 Rapid Gender Assessment Survey	30,940	37.5	32
Income loss	COVID-19 Health Services Disruption Survey	15,583	27.8	75
Income loss	Research for Effective Covid-19 Response Panel Survey	4,982	65.9	4
Not working to care for others	UMD Global CTIS and Delphi US CTIS	540,567	53.0	176
Increase in care for others	Survey on Gender Equality at Home	40,931	54.4	105
Increase in care for others	COVID-19 Rapid Gender Assessment Survey	35,096	43.9	20
Increase in chores	Survey on Gender Equality at Home	72,396	54.4	105
Increase in chores	COVID-19 Rapid Gender Assessment Survey	33,931	45.9	20
**Education**				
School drop out	COVID-19 Health Services Disruption Survey	31,941	34.1	51
Adequate remote learning	COVID-19 Health Services Disruption Survey	14,268	41.7	51
**Safety at home and in the community**				
Perception of GBV Increase	COVID-19 Rapid Gender Assessment Survey	36,187	42.9	29
Perception of GBV Increase	COVID-19 Health Services Disruption Survey	1,221	100.0	70
Feeling unsafe at home	Survey on Gender Equality at Home	156,240	54.3	105
Feeling unsafe at home	COVID-19 Rapid Gender Assessment Survey	35,759	42.8	21

Supplemental Table S2. Results from the mixed effects logistic regression for sexual and reproductive health

Indicator	Women	Age 18-24	Age 35-44	Age 35-64	More than High School	No Education	Primary Education	Tertiary+ Education	Rural	Capital City	Urban Non-capital City	Number of Respondents	% Women	Number of Countries	Data Source
Disruption in reproductive health [1]	0.99 (0.87-1.11)	-	-	0.91 (0.79-1.05)	0.92 (0.78-1.08)	-	-	-	1.01 (0.87-1.16)	-	-	6,405	46.4%	5	COVID-19 Rapid Gender Assessment Survey (RGA)
Disruption in reproductive health [2]	-	1.35 (1.09-1.68)	1.18 (0.94-1.47)	-	-	1.23 (0.95-1.59)	1.10 (0.87-1.40)	1.14 (0.86-1.53)	-	1.24 (0.86-1.80)	1.95 (1.31-2.90)	2,970	100%	5	COVID-19 Rapid Gender Assessment Survey (RGA)
Disruption in reproductive health [3]	0.98 (0.87-1.11)	1.11 (0.95-1.29)	1.00 (0.86-1.15)	-	-	1.14 (0.95-1.35)	1.03 (0.87-1.20)	0.99 (0.82-1.19)	-	1.73 (1.34-2.24)	2.31 (1.76-3.04)	6,405	46.4%	5	COVID-19 Rapid Gender Assessment Survey (RGA)

[1] Model run using men and women and covariate categories comparable to other main text models
[2] Model run on a subset of women only, using more detailed demographic information available uniquely from source
[3] Model run on men and women, using more detailed demographic information available uniquely from source

Supplemental Table S2. Presents odds ratios (ORs) and 95% uncertainty intervals (UIs) from mixed effects logistic regression models exploring the association between disruption in reproductive health and gender, adjusting for geography, age, education attainment, and urbanicity. The first row presents ORs from the model reported in the main text that uses covariate specifications aligned with the regression framework used for other indicators. Because this indicator was only available from one source (*COVID-19 Rapid Gender Assessment Surveys*), we investigated disruptions in reproductive health care beyond the main regression framework using more detailed covariate specifications of age, educational attainment, and urbanicity. The second row presents results of a model employing more detailed specifications on a woman-only subset of participants. The third row presents results of the model employing more detailed covariate specifications on the full sample (men and woman). For each regression model covariate, the reference categories are listed in parentheses: woman (men); age 18-24 (age 25-34); age 35-44 (age 25-34); no education (secondary education); primary education (secondary education); tertiary+ education (secondary education); capital city (rural); urban non-capital city (rural).

Figure S1. Data availability for each indicator by data source provider.

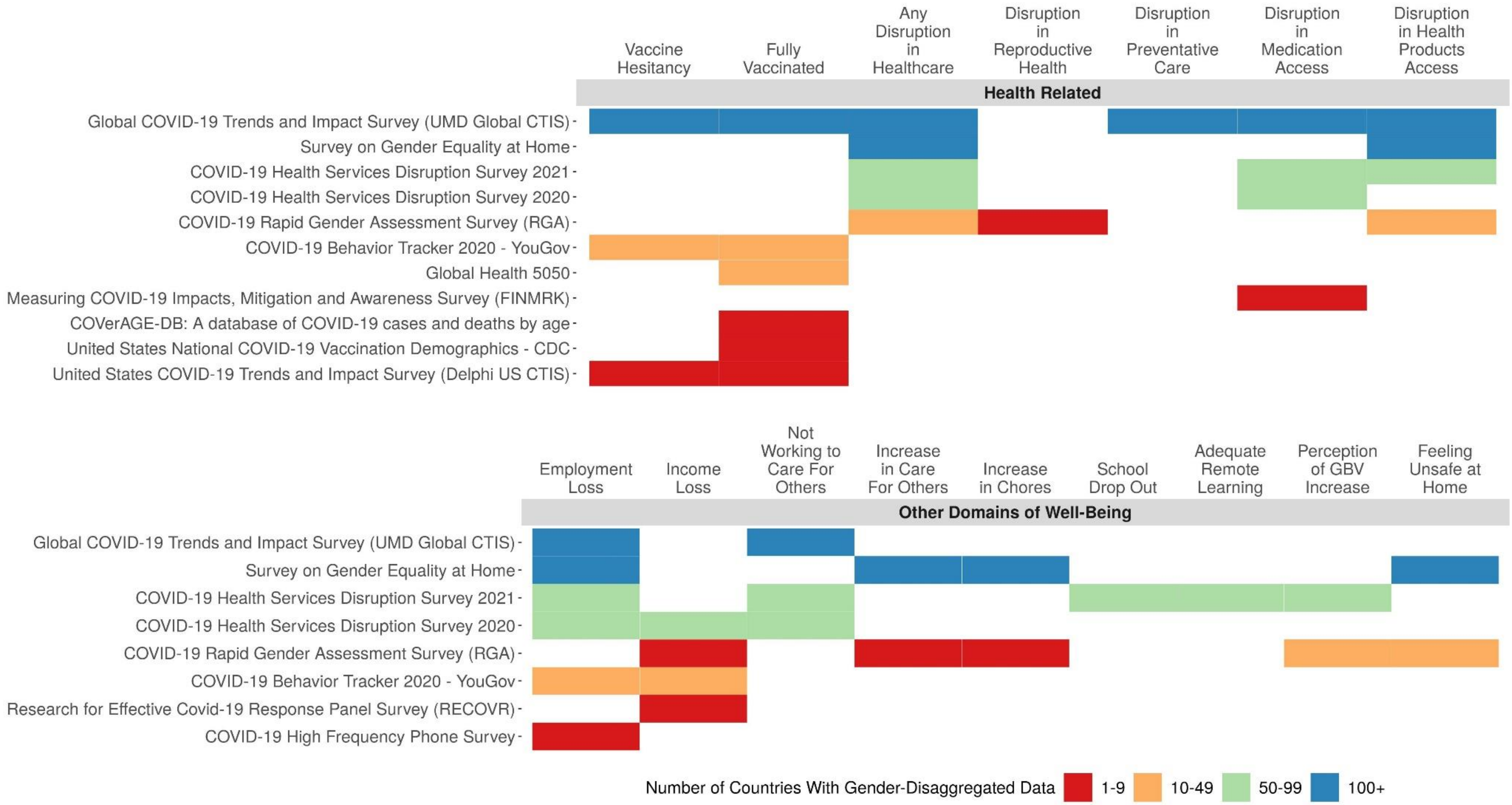

Figure S2. Number of data sources available by country and indicator for Central Europe, Eastern Europe, and Central Asia.

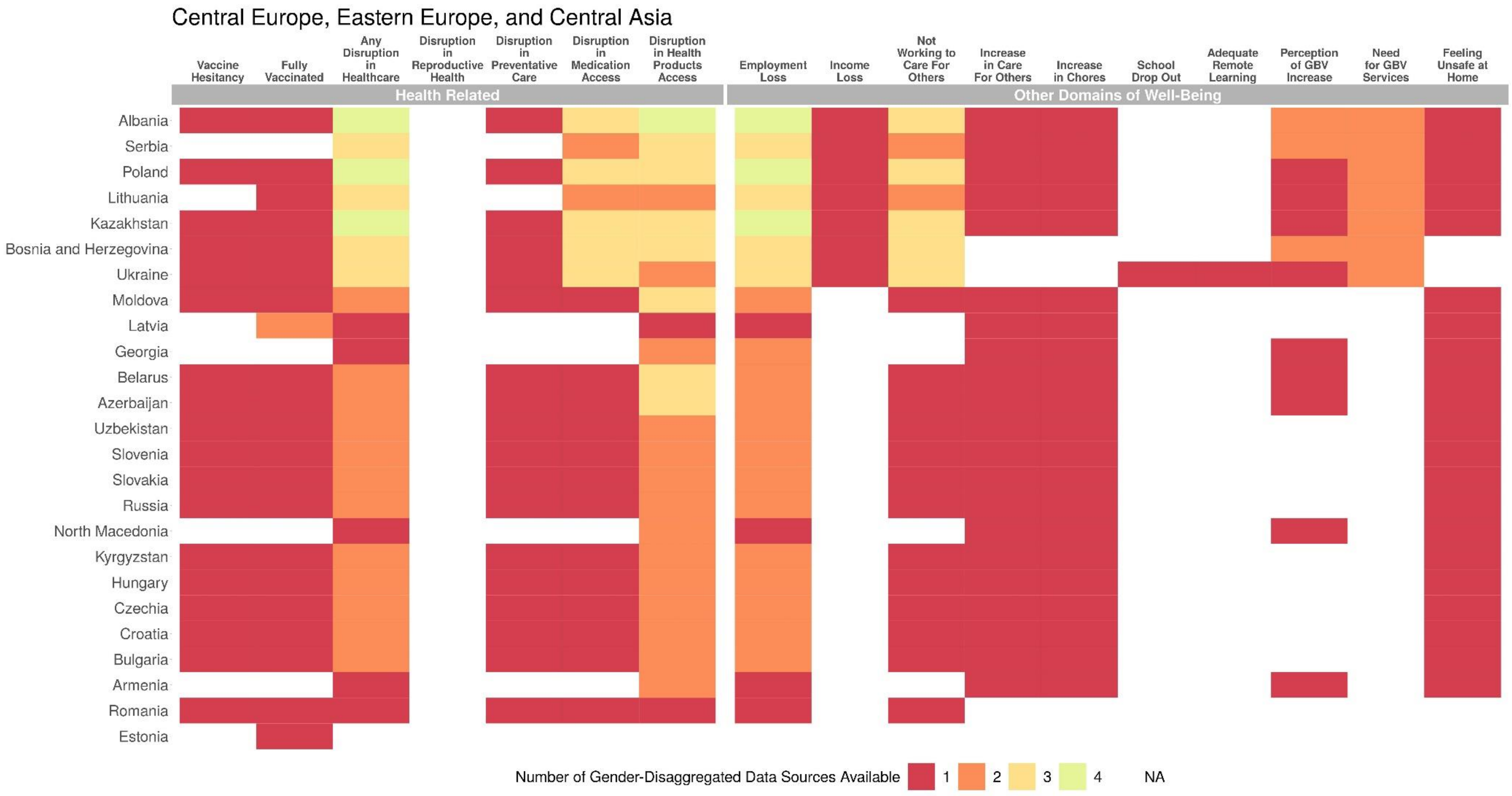

Figure S3. Number of data sources available by country and indicator for Latin America and the Caribbean.

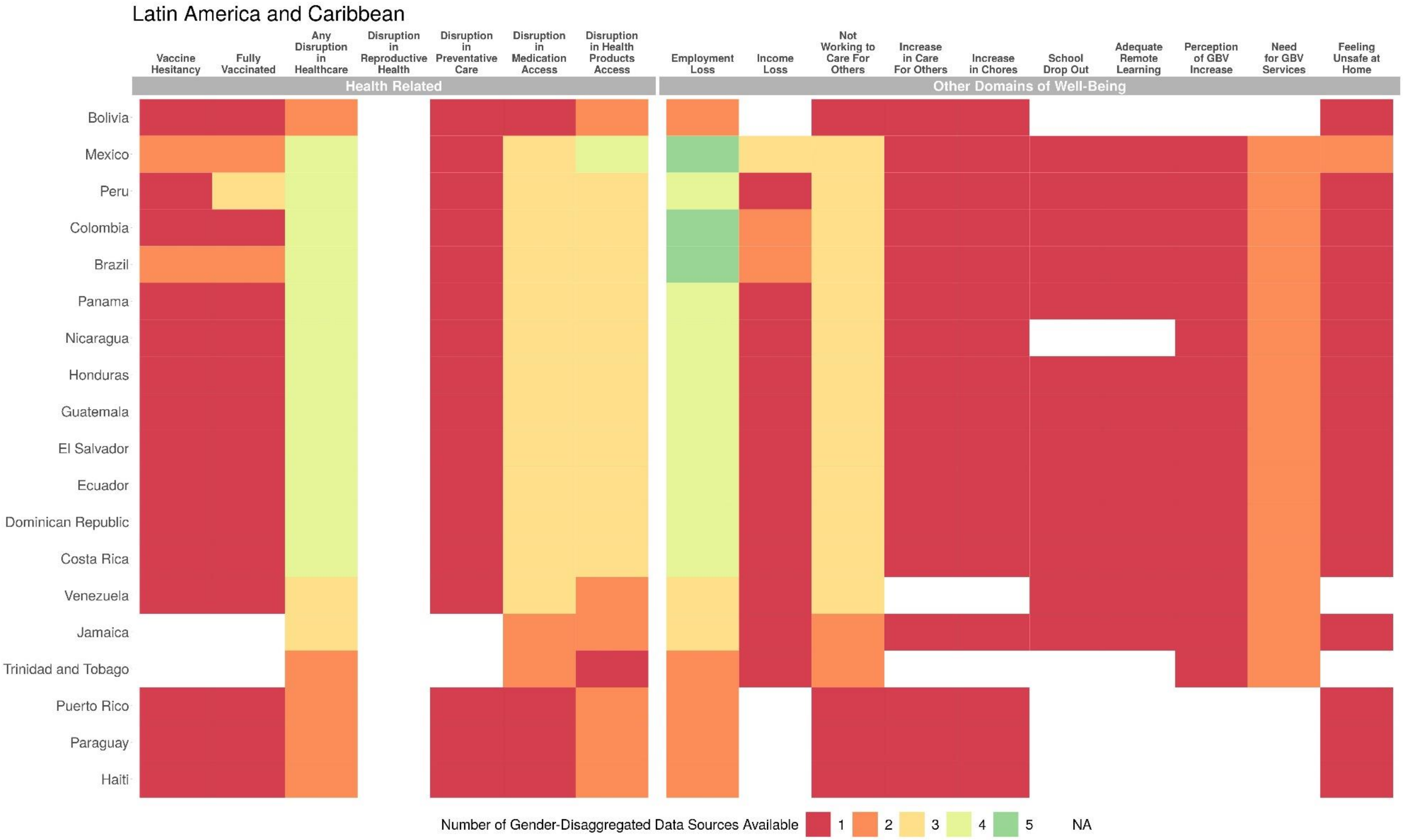

Figure S4. Number of data sources available by country and indicator for High-income countries.

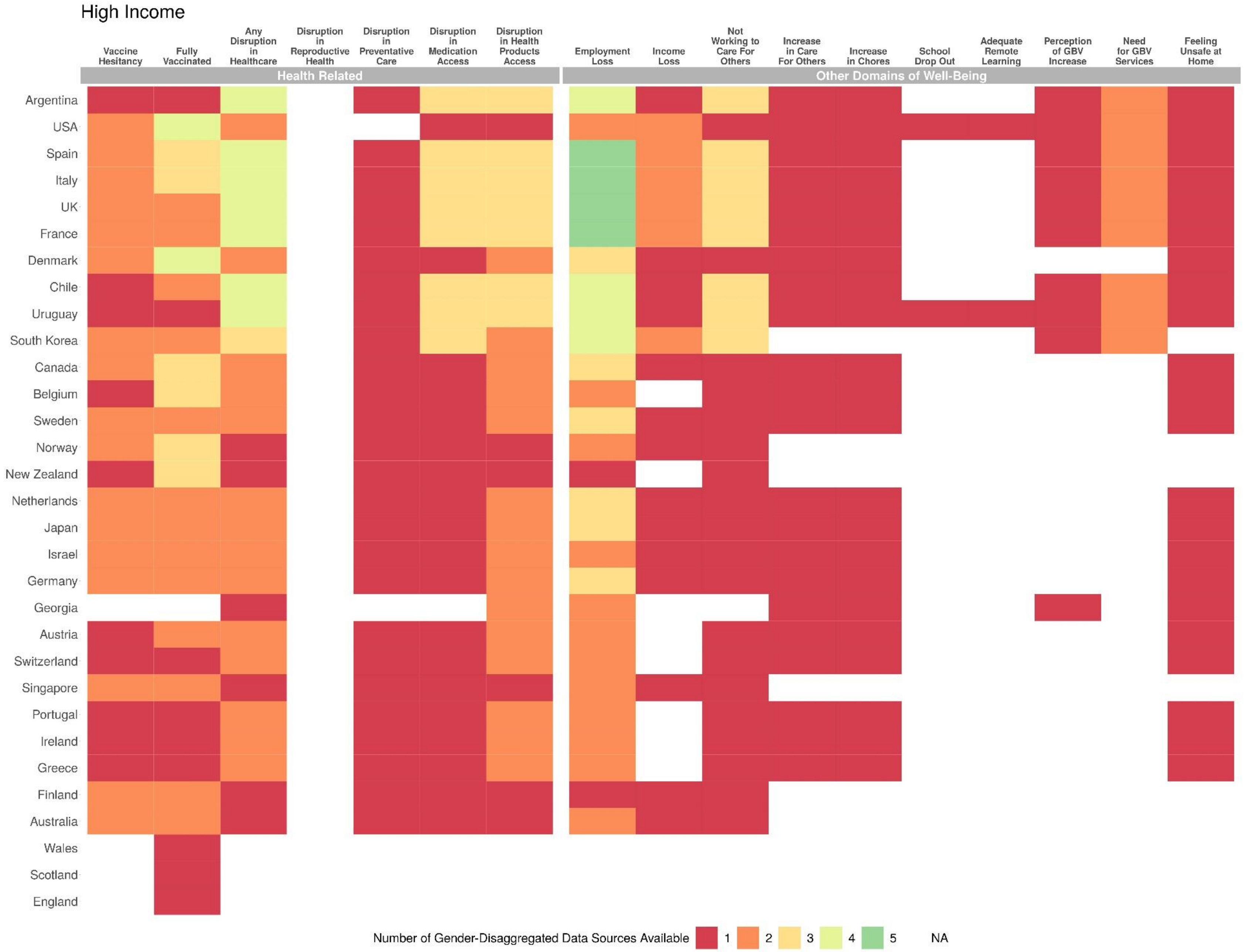

Figure S5. Number of data sources available by country and indicator for South Asia.

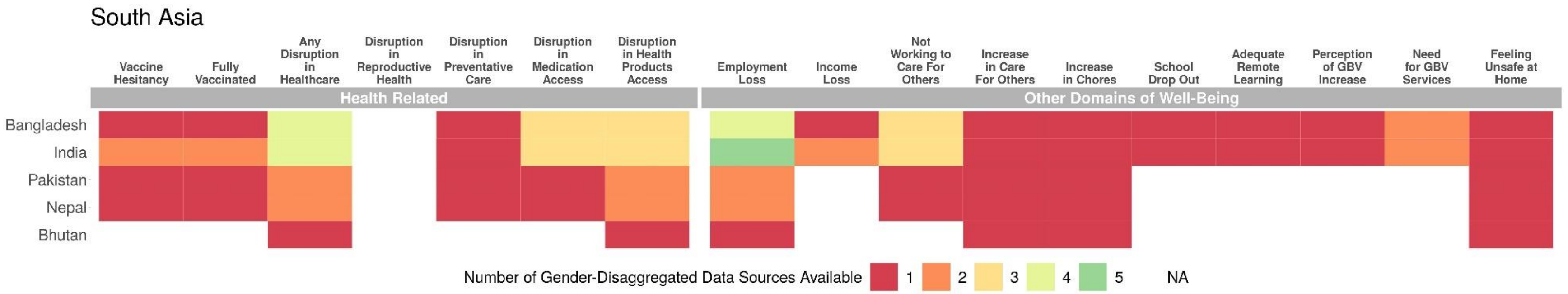

Figure S6. Number of data sources available by country and indicator for North Africa and the Middle East.

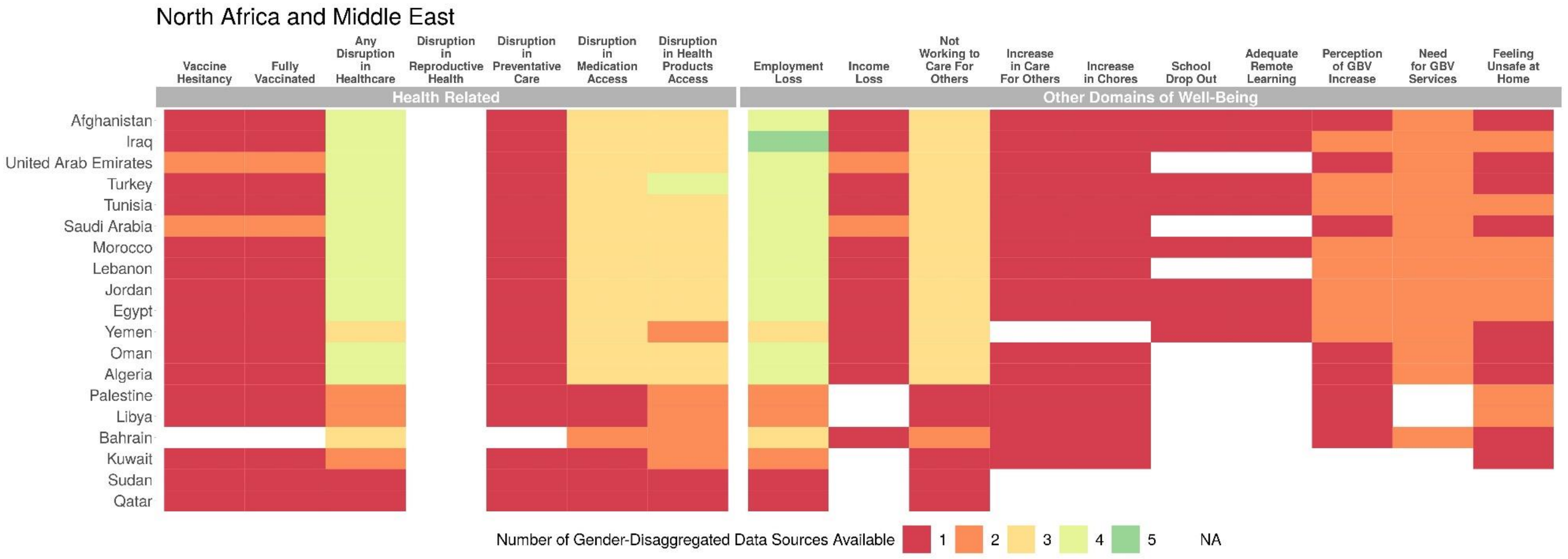

Figure S7. Number of data sources available by country and indicator for Southeast Asia, East Asia, and Oceania.

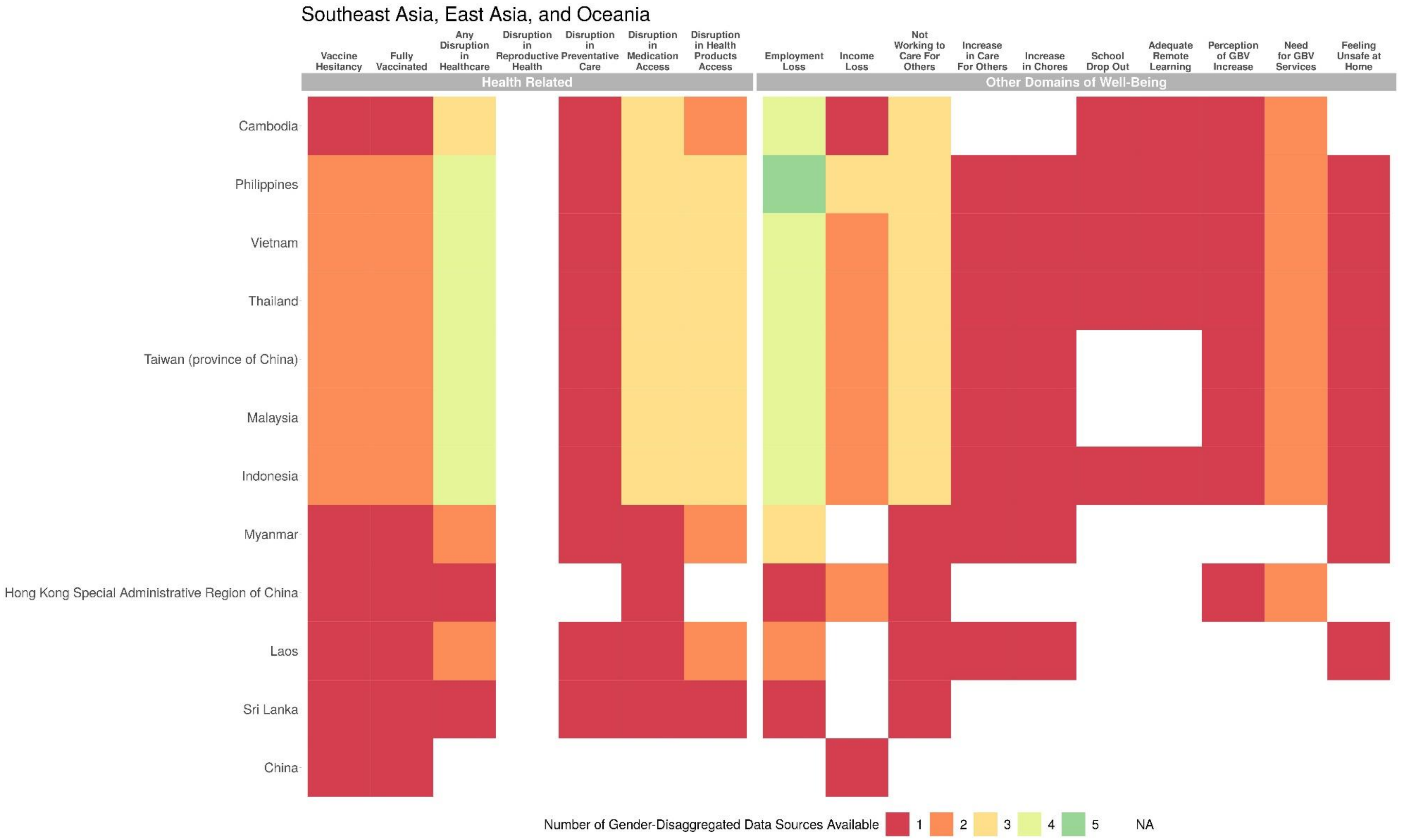

Figure S8. Number of data sources available by country and indicator for Sub-Saharan Africa.

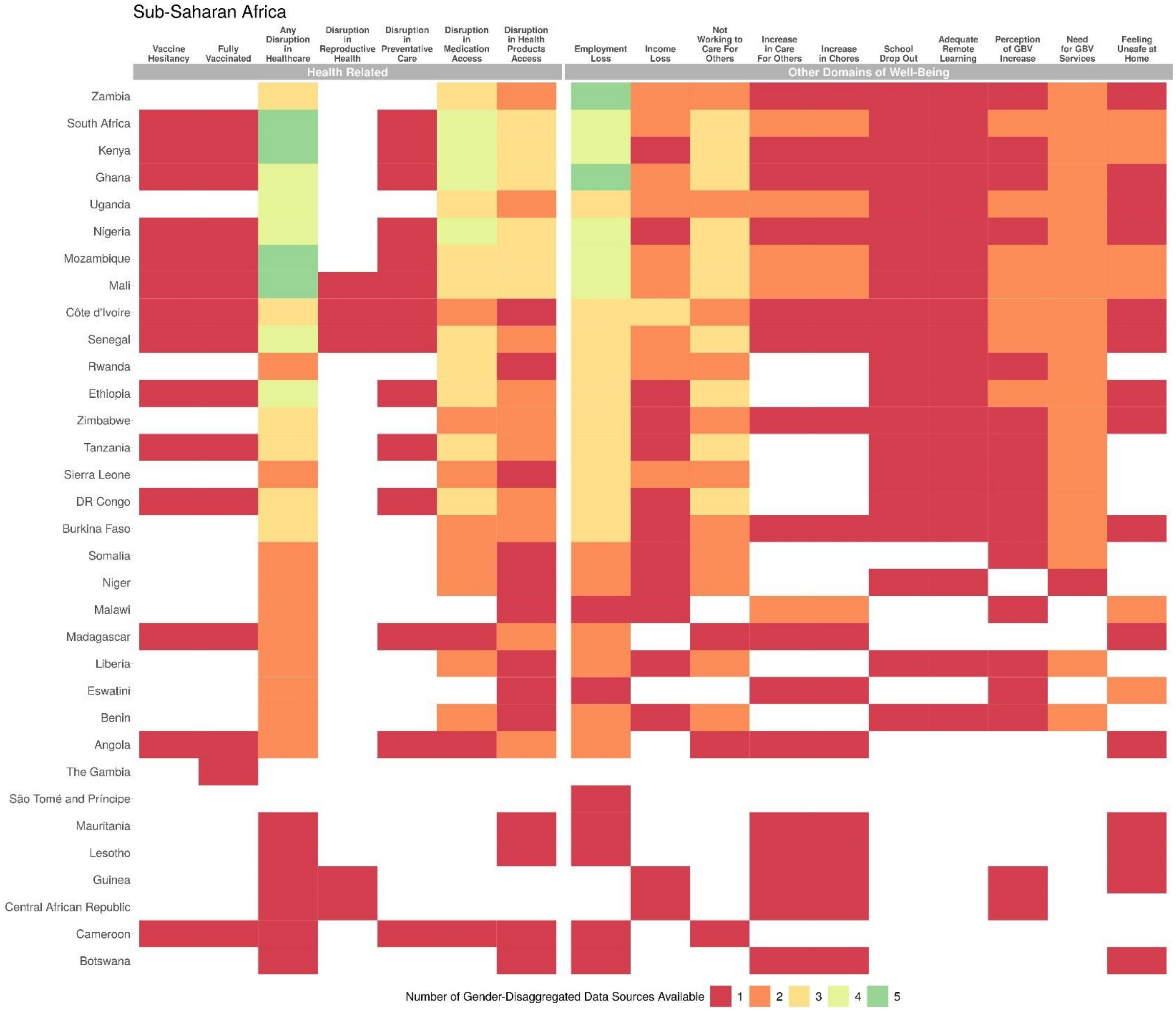

Figure S9. Regional-level relative gender gaps in time-varying Indicators.

Country-level gender-specific estimates were population-weighted and aggregated to total and regional levels for January 2021 and September 2021. The direction of disparities is shown by color, and the transparency level indicates statistical significance at the alpha=.05 level.

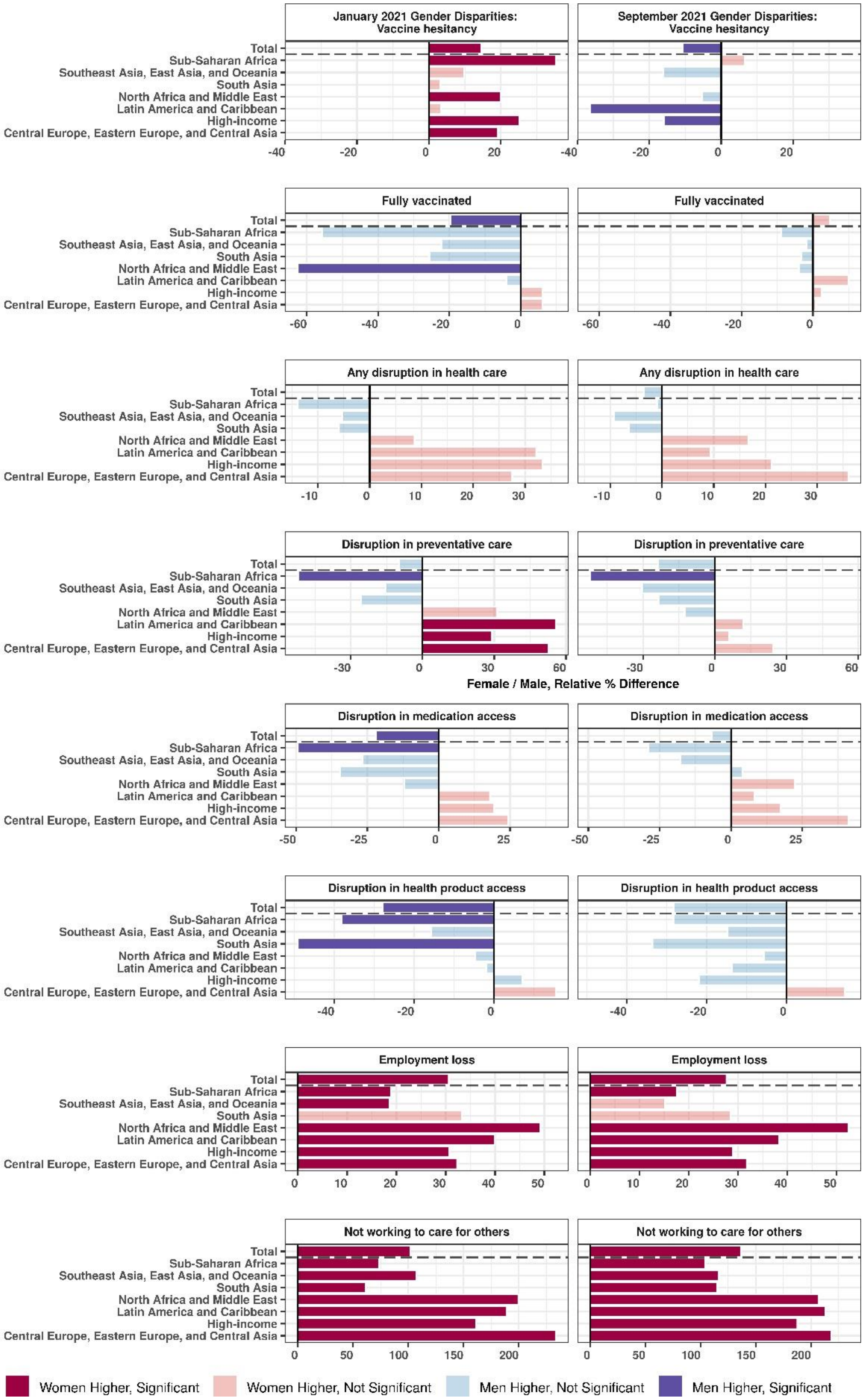

Figure S10. Country-Level Gender Disparities in Time-Varying Indicators in January 2021.
Estimates for indicators for which data were available for multiple time periods are summarized by gender and country, for January 2021. The direction of disparities is shown by color, and the transparency level indicates statistical significance at the alpha=.05 level.

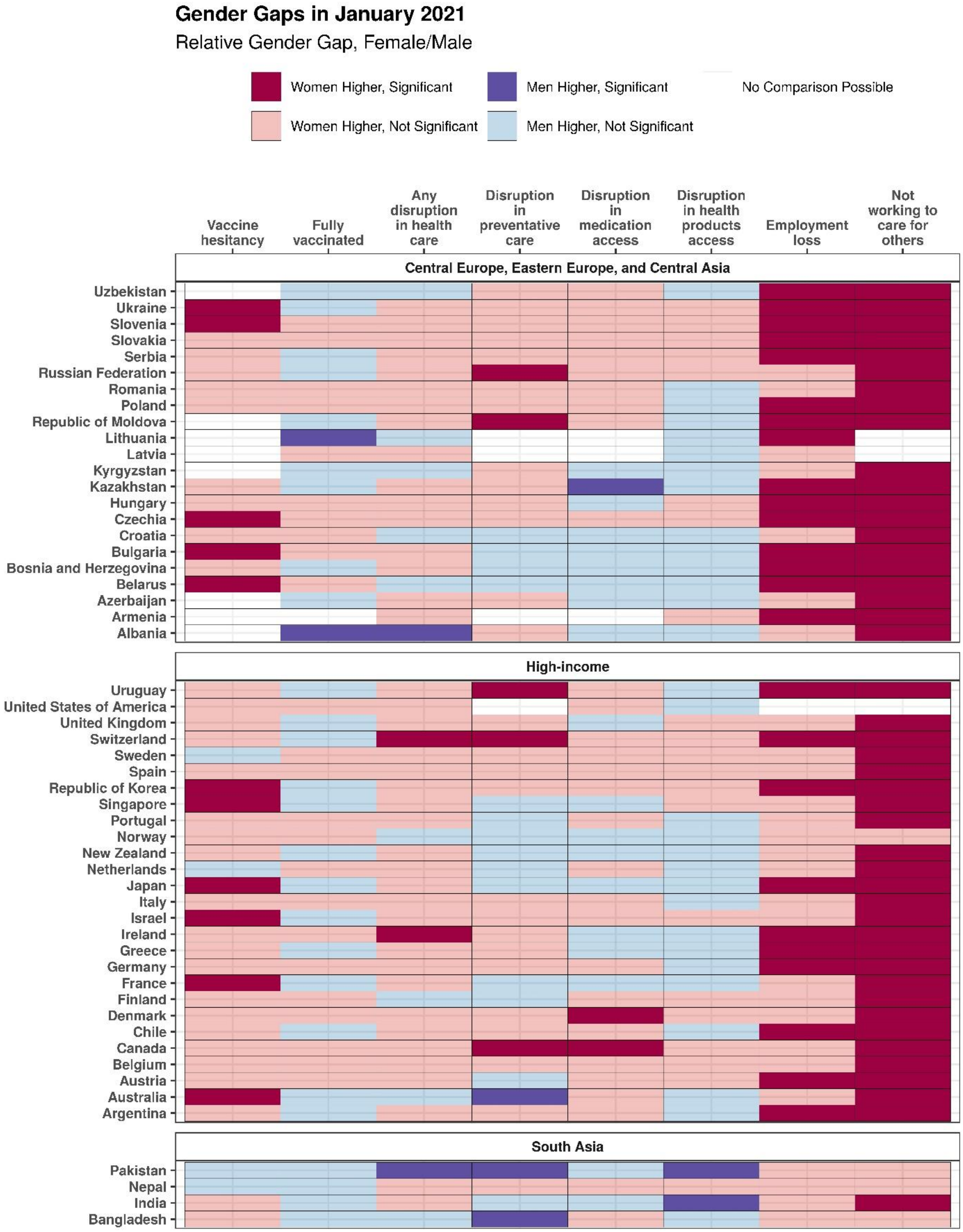

Figure S10. Country-Level Gender Disparities in Time-Varying Indicators in January 2021 (Cont). Estimates for indicators for which data were available for multiple time periods are summarized by gender and country, for January 2021. The direction of disparities is shown by color, and the transparency level indicates statistical significance at the alpha=.05 level.

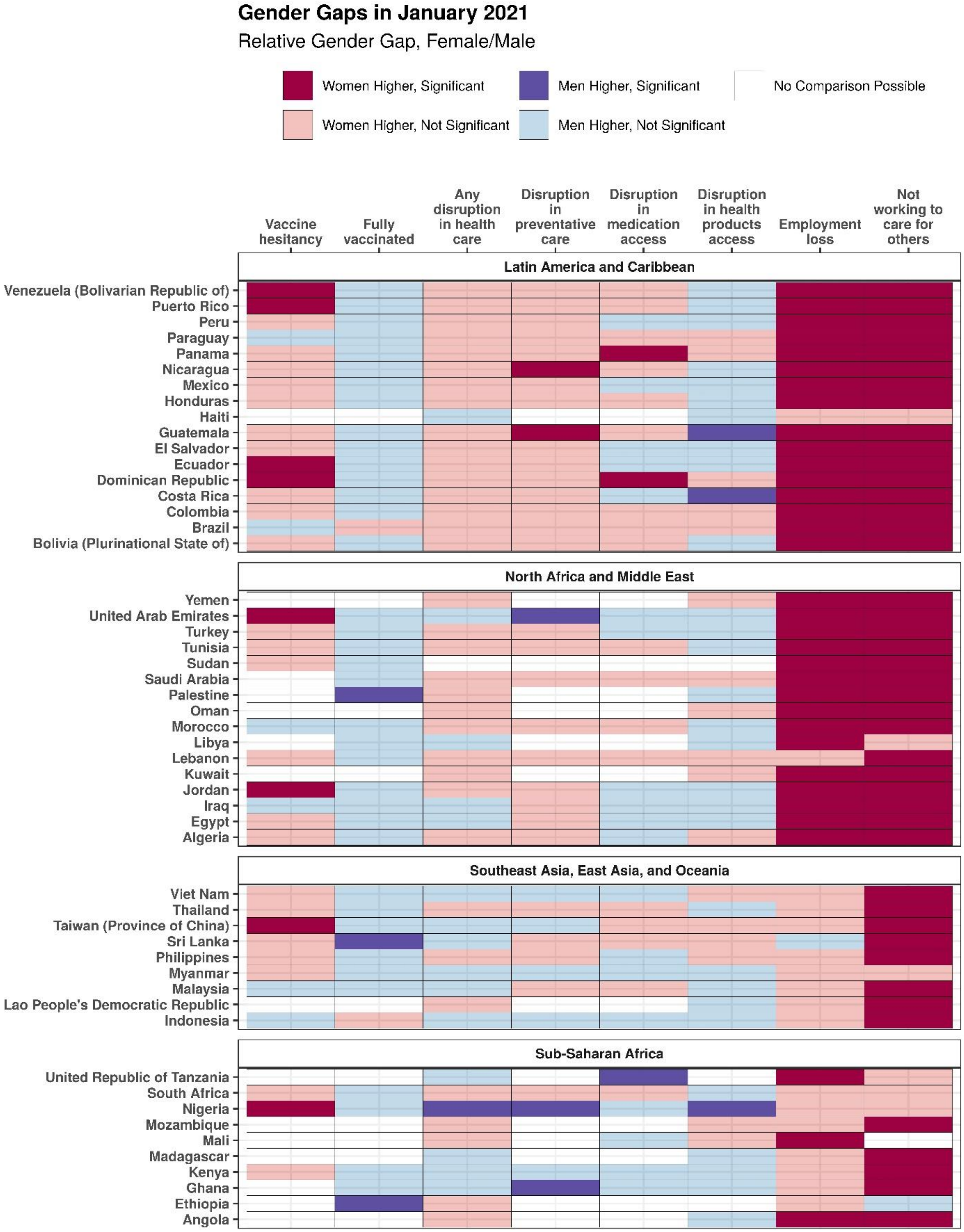

Figure S11. Country-Level Gender Disparities in Time-Varying Indicators in September 2021
Estimates for indicators for which data were available for multiple time periods are summarized by gender and country, for September 2021. The direction of disparities is shown by color, and the transparency level indicates statistical significance at the alpha=.05 level.

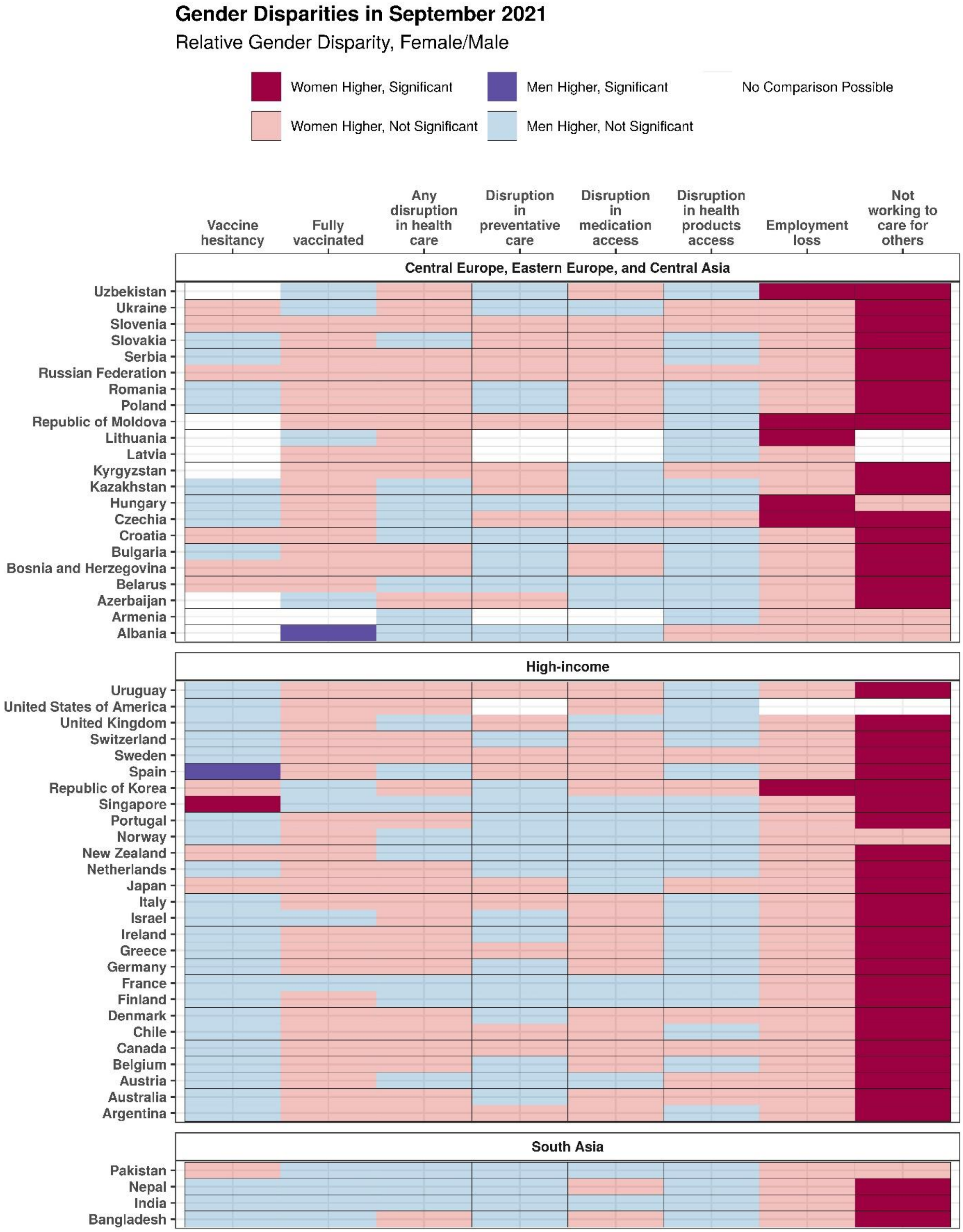

Figure S11. Country-Level Gender Disparities in Time-Varying Indicators in September 2021 (Cont).

Estimates for indicators for which data were available for multiple time periods are summarized by gender and country, for September 2021. The direction of disparities is shown by color, and the transparency level indicates statistical significance at the alpha=.05 level.

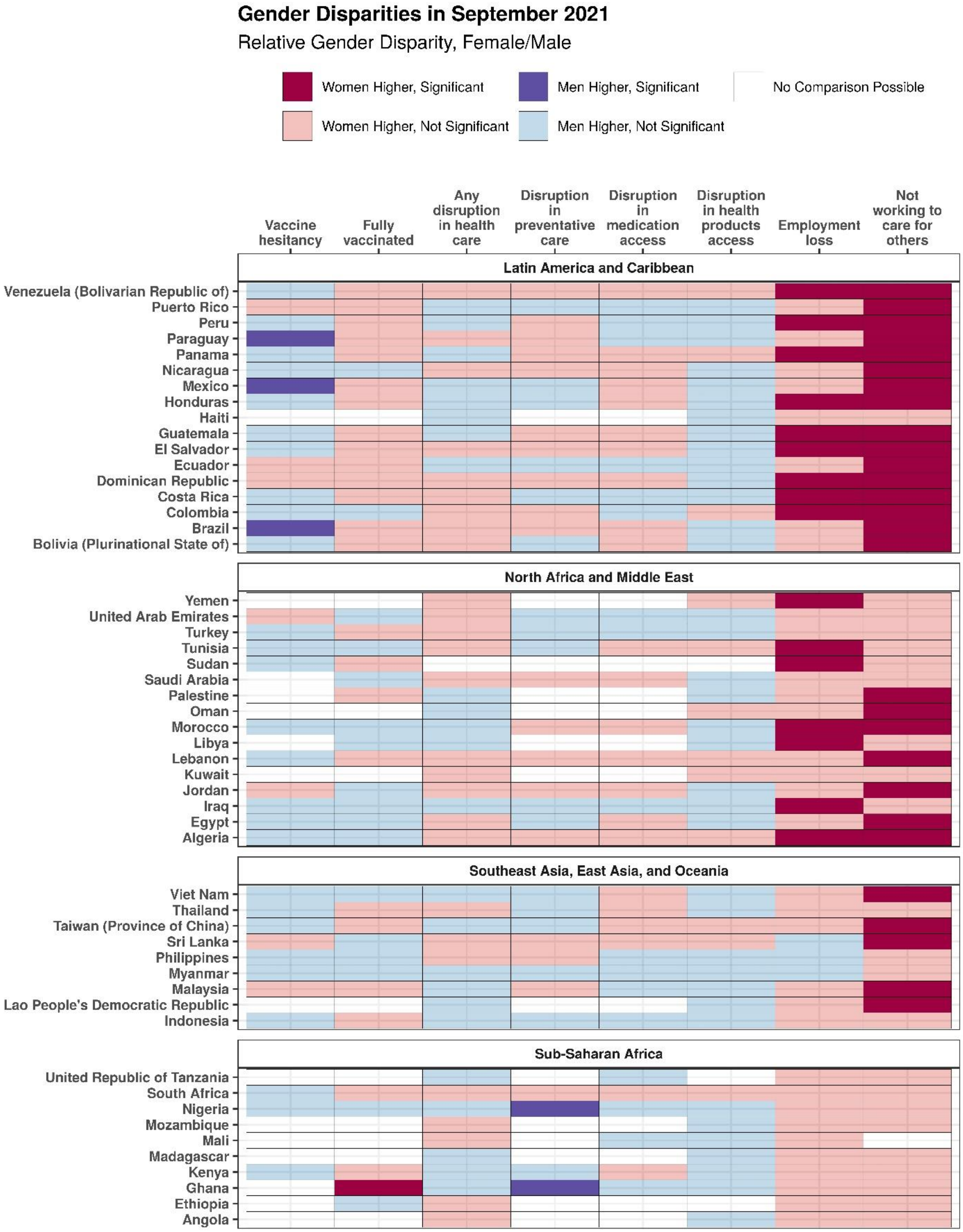

Figure S12. Country-Level Trends by Gender in Cross-Sectional Indicators.

Estimates for indicators available cross-sectionally are summarized by gender and country. Gender is indicated by point color. 95% uncertainty intervals for each estimate are also shown. Countries are categorized according to major world region.

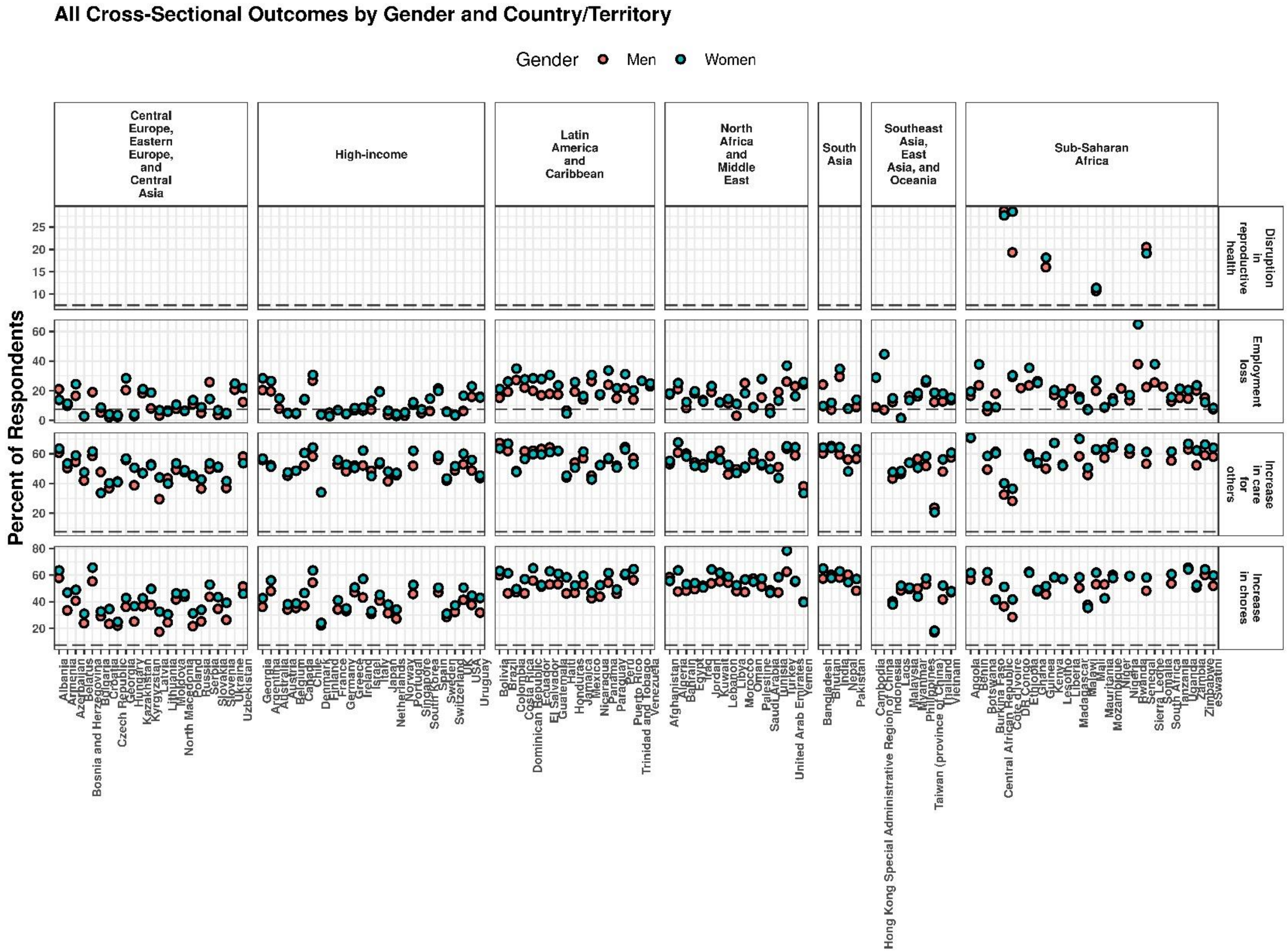

Figure S12. Country-Level Trends by Gender in Cross-Sectional Indicators (Cont.).

Estimates for indicators available cross-sectionally are summarized by gender and country. Gender is indicated by point color. 95% uncertainty intervals for each estimate are also shown. Countries are categorized according to major world region.

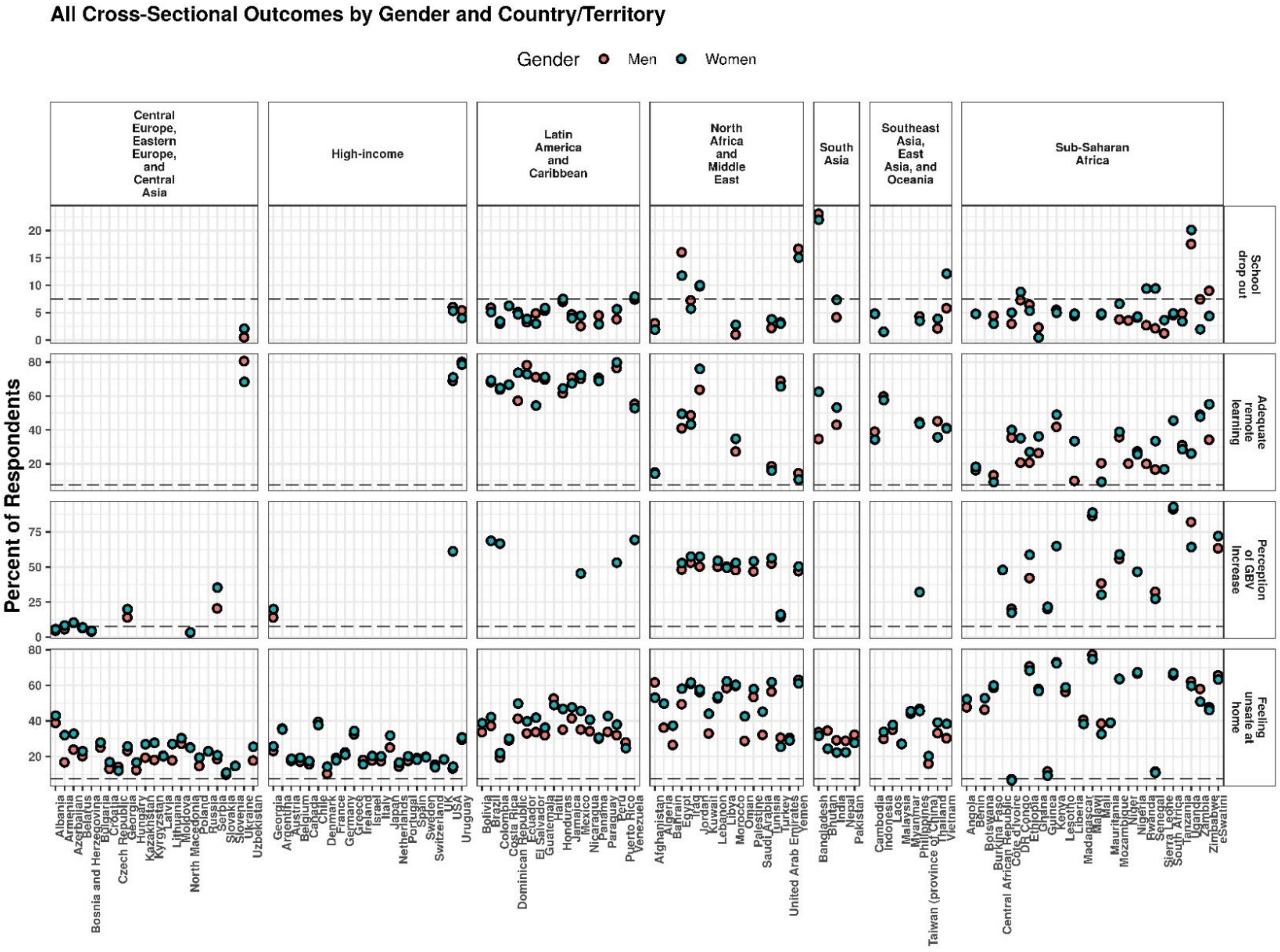

Figure S13. Time-specific multivariate logistic regression results from UMD Global CTIS and Delphi US CTIS Surveys, for Healthcare, Economic and work-related concerns, and Vaccine hesitancy and uptake indicators.

Time-specific odds ratios (ORs) and 95% uncertainty intervals (UIs) from mixed effects logistic regression models using available data from the University of Maryland Social Data Science Center Global COVID-19 Trends and Impact Survey (UMD Global CTIS) and the Delphi Group and Carnegie Mellon University US COVID-19 Trends and Impact Survey (Delphi US CTIS) are shown. Time-specificity of the result is indicated by the color of the points and error bars. For healthcare and economic and work-related concerns indicators (rows 1 and 2), data was available only from UMD Global CTIS and split into three time periods: (1) June 2020 – November 2020; (2) December 2020 – March 2021; and (3) April 2021+; for these indicators, data was available through June 2021. For vaccine hesitancy and uptake indicators (row 3), data was available from both UMD Global CTIS and Delphi US CTIS and split into two time periods: (1) January 2021 – March 2021; and (2) April 2021+; for these indicators data was available through September 2021. For each regression model covariate, the reference categories are listed in parentheses: woman (man); age 35-64 (age less than 35); age 65+ (age less than 35); some tertiary education (less than tertiary education); rural (urban).

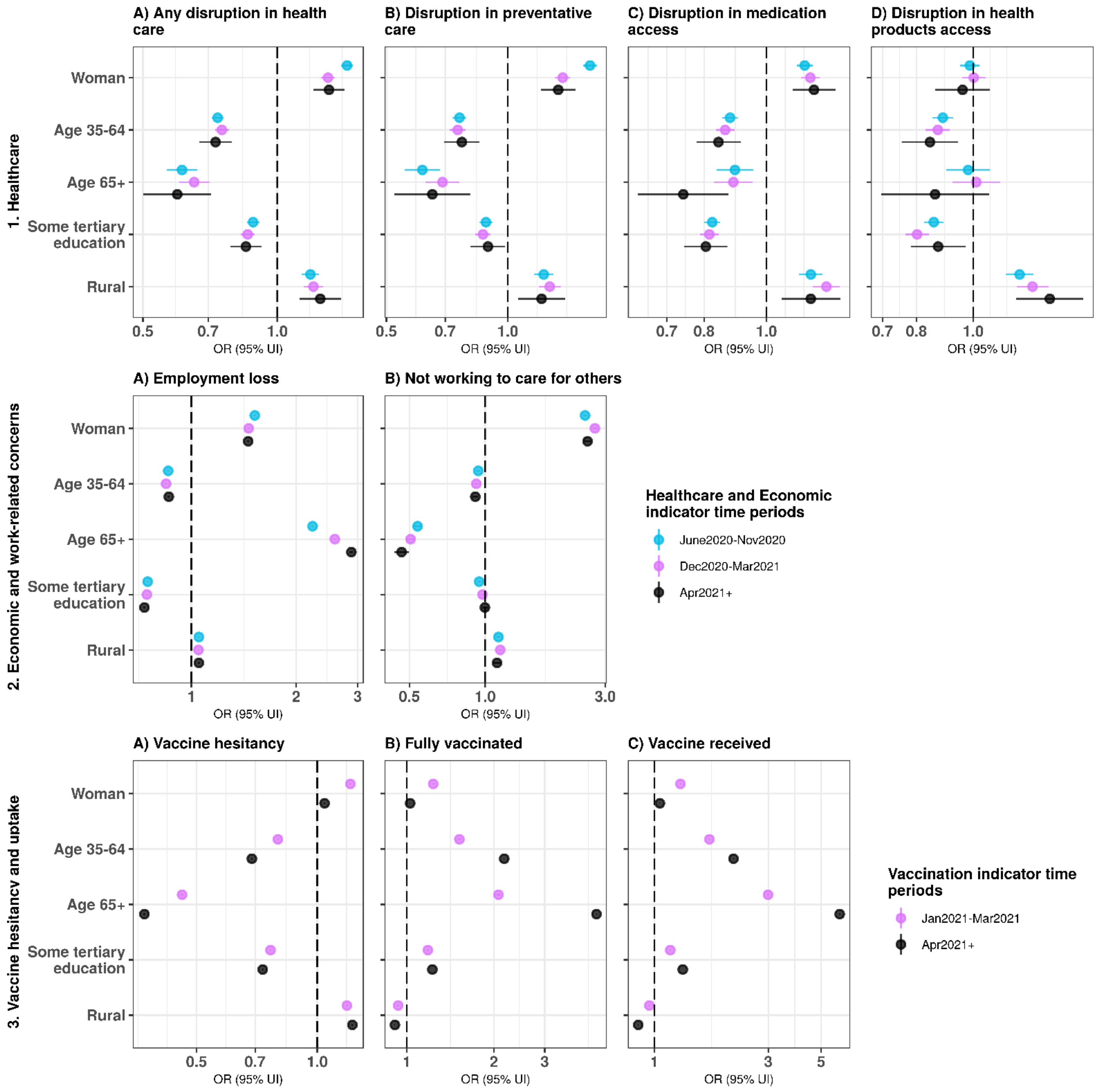
A) Any disruption in health care
B) Disruption in preventative care
C) Disruption in medication access
D) Disruption in health products access
1. Healthcare
Woman
Age 35-64
Age 65+
Some tertiary education
Rural
OR (95% UI)
A) Employment loss
B) Not working to care for others
2. Economic and work-related concerns
Healthcare and Economic indicator time periods
June2020-Nov2020
Dec2020-Mar2021
Apr2021+
A) Vaccine hesitancy
B) Fully vaccinated
C) Vaccine received
3. Vaccine hesitancy and uptake
Vaccination indicator time periods
Jan2021-Mar2021
Apr2021+

Figure S14. Significance of the gender coefficient across indicators, sources and regions

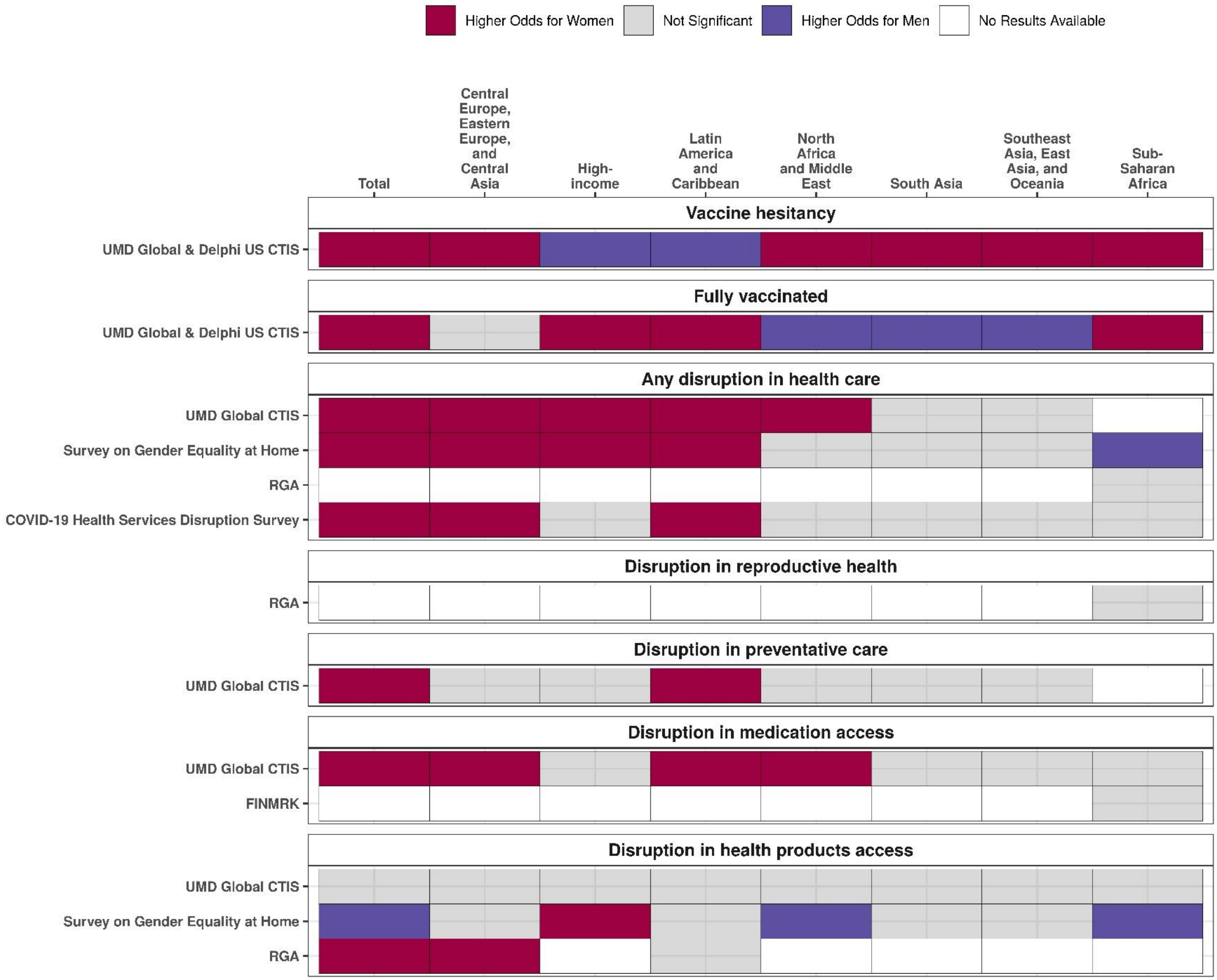

Figure S14. Significance of the gender coefficient across indicators, sources and regions [Cont.]

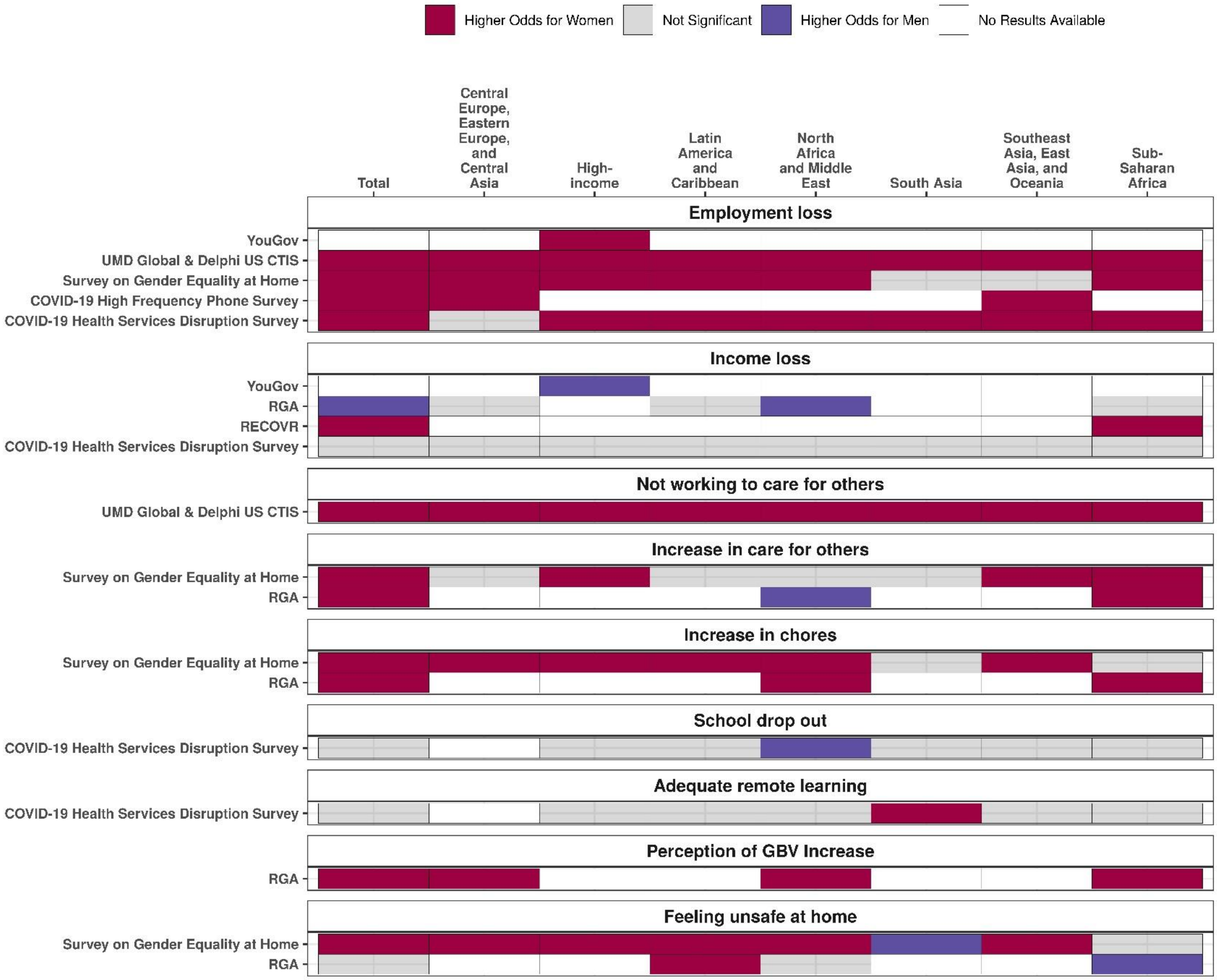

Figure S15. Cross-sectional gender gaps, presented by world region and indicator.

Cross-sectional estimates are shown by world region, indicator, and gender. Estimates are shown with 95% uncertainty intervals. World region is indicated by color of the point and uncertainty intervals and gender is indicated by shape. Each result is plotted in comparison to the gender-indicator-specific total estimate, shown in gray above to the region-specific estimate. Row 1 (vaccine hesitancy and uptake) shows results for vaccine hesitancy and uptake indicators, for which input data was available over time and time series analysis was used to create September 2021 estimates shown in the figure. Row 2 (healthcare) shows results for healthcare indicators. For disruption in preventative care, disruption in medication access, any disruption in healthcare, and disruption in health product access, input data was available over time and time series analysis was used to create September 2021 estimates shown in the figure. For disruption in reproductive health, input data was only available cross-sectionally, and data synthesis methods were used to create cross-sectional estimates shown in the figure. Row 3 (education) shows results for education indicators, for which input data was only available cross-sectionally, and data synthesis methods were used to create cross-sectional estimates shown in the figure. Row 4 (economic and work-related) shows results for economic and work-related concerns indicators. For employment loss and not working to care for others, input data was available over time and time series analysis was used to create September 2021 estimates shown in the figure. For income loss, increase in chores, and increase in care for others, input data was only available cross-sectionally, and data synthesis methods were used to create cross-sectional estimates shown in the figure. Row 5 (Safety, home and community) shows results for safety at home and in the community indicators, for which input data was only available cross-sectionally and data synthesis methods were used to create cross-sectional estimates shown in the figure.

Supplemental Figure 14. Cross−sectional gender gaps, presented by world region and indicator.

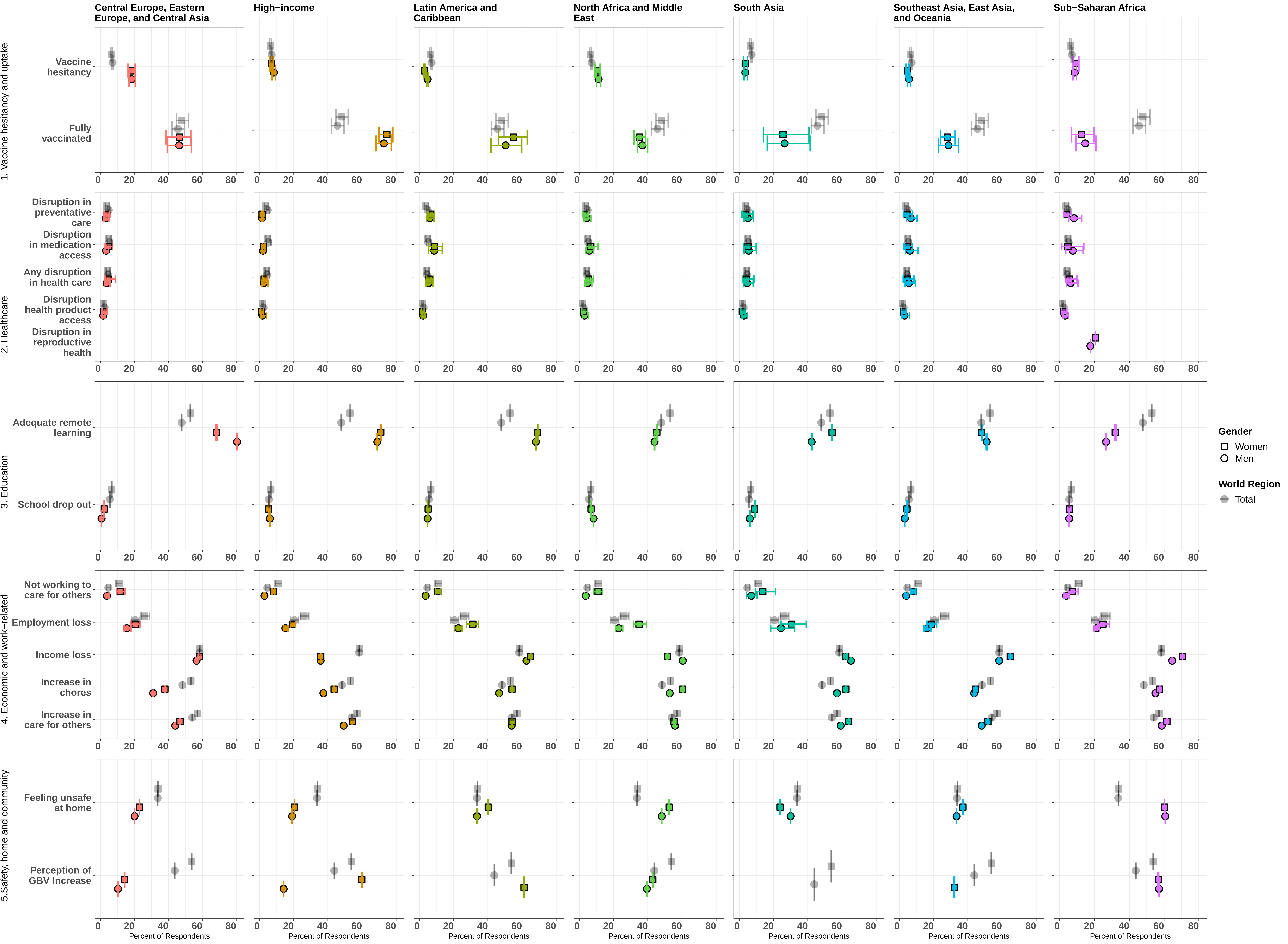

Figure S16. Timeseries Models and Input Data

Timeseries model inputs and estimates are shown by indicator, country, gender, and age (where relevant). Input data are shown with 95% uncertainty intervals. Model fits from gaussian process regression are shown over time with 95% prediction intervals. Data source is indicated by point type. Gender is indicated by data and model color.

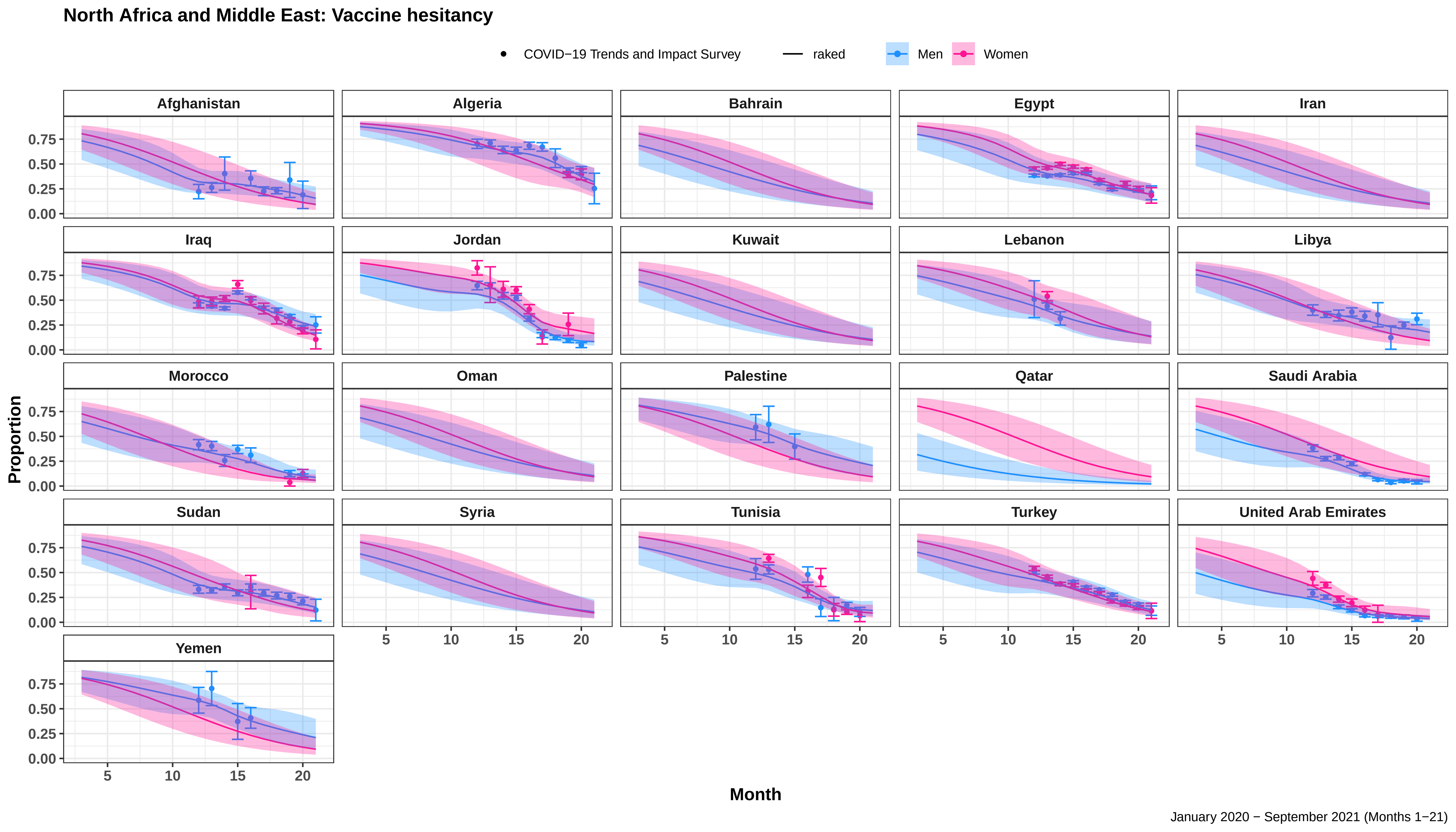
North Africa and Middle East: Vaccine hesitancy
COVID−19 Trends and Impact Survey
raked
Men
Women
Afghanistan
Algeria
Bahrain
Egypt
Iran
Iraq
Jordan
Kuwait
Lebanon
Libya
Morocco
Oman
Palestine
Qatar
Saudi Arabia
Sudan
Syria
Tunisia
Turkey
United Arab Emirates
Yemen
Proportion
0.00
0.25
0.50
0.75
Month
5
10
15
20
January 2020 − September 2021 (Months 1−21)

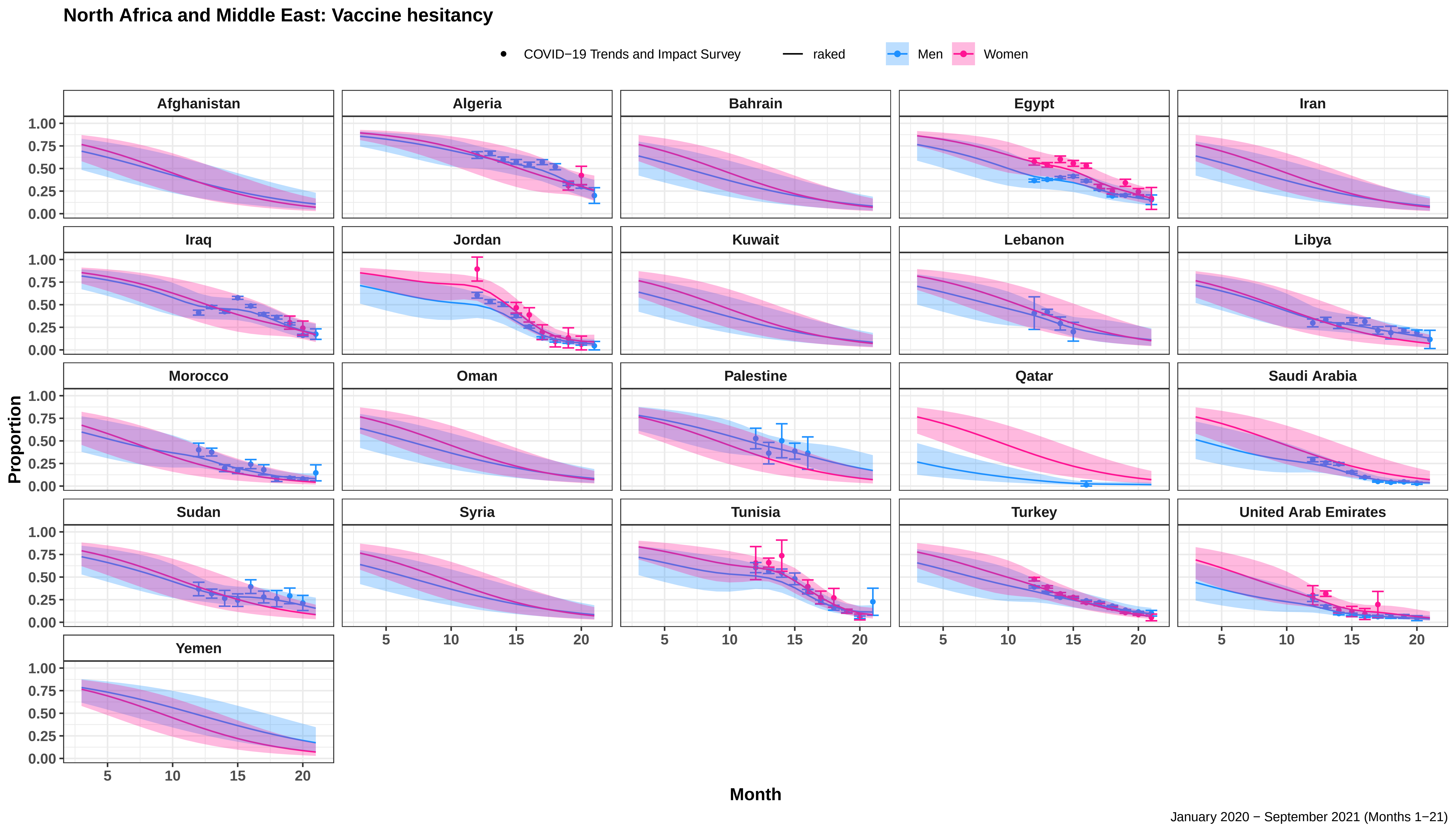

North Africa and Middle East: Vaccine hesitancy
COVID−19 Trends and Impact Survey
raked
Men
Women
Afghanistan
Algeria
Bahrain
Egypt
Iran
Iraq
Jordan
Kuwait
Lebanon
Libya
Morocco
Oman
Palestine
Qatar
Saudi Arabia
Sudan
Syria
Tunisia
Turkey
United Arab Emirates
Yemen
Proportion
1.00
0.75
0.50
0.25
0.00
5
10
15
20
Month
January 2020 − September 2021 (Months 1−21)

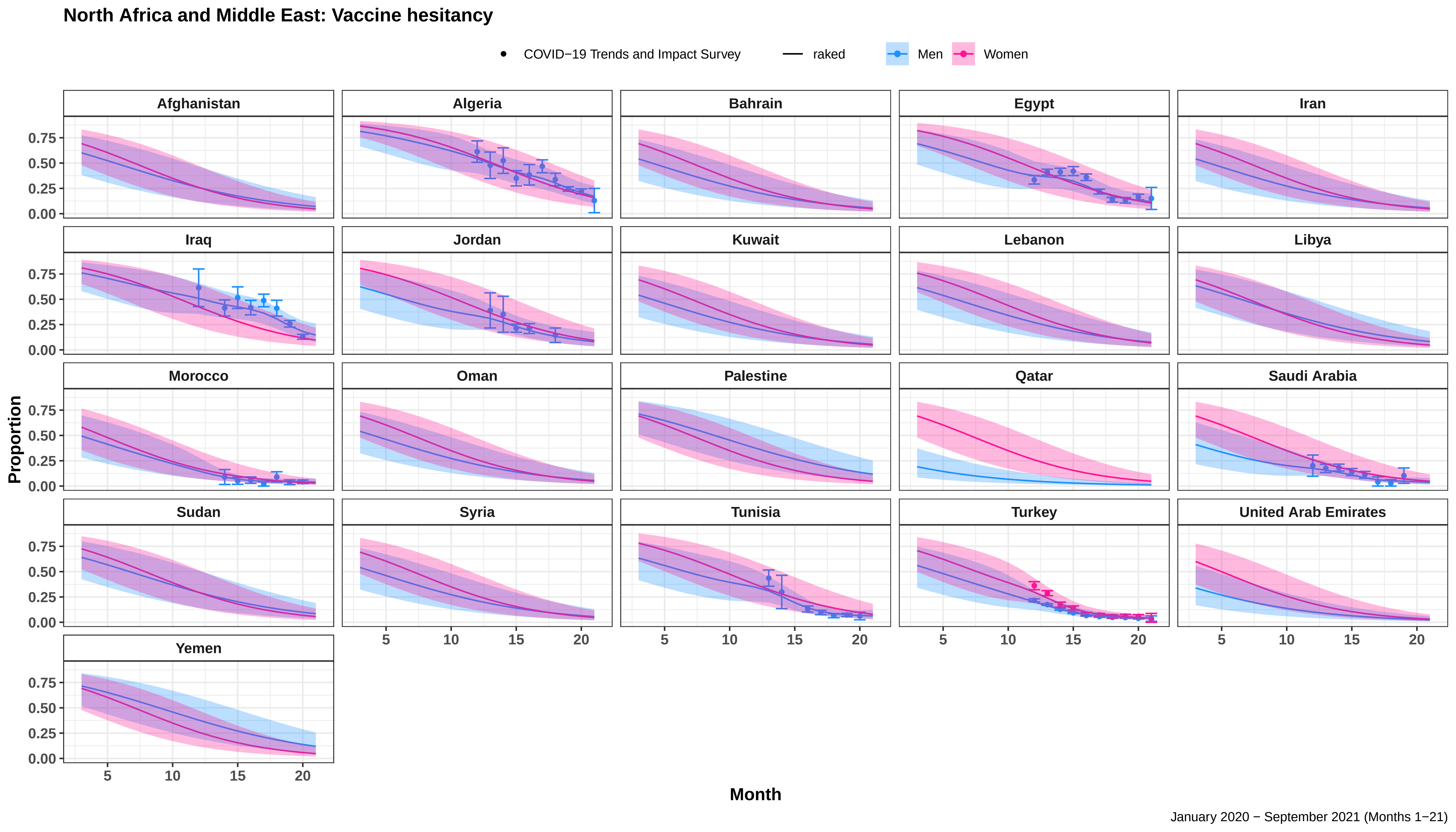
North Africa and Middle East: Vaccine hesitancy
COVID−19 Trends and Impact Survey
raked
Men
Women
Afghanistan
Algeria
Bahrain
Egypt
Iran
Iraq
Jordan
Kuwait
Lebanon
Libya
Morocco
Oman
Palestine
Qatar
Saudi Arabia
Sudan
Syria
Tunisia
Turkey
United Arab Emirates
Yemen
Proportion
0.00
0.25
0.50
0.75
Month
5
10
15
20
January 2020 − September 2021 (Months 1−21)

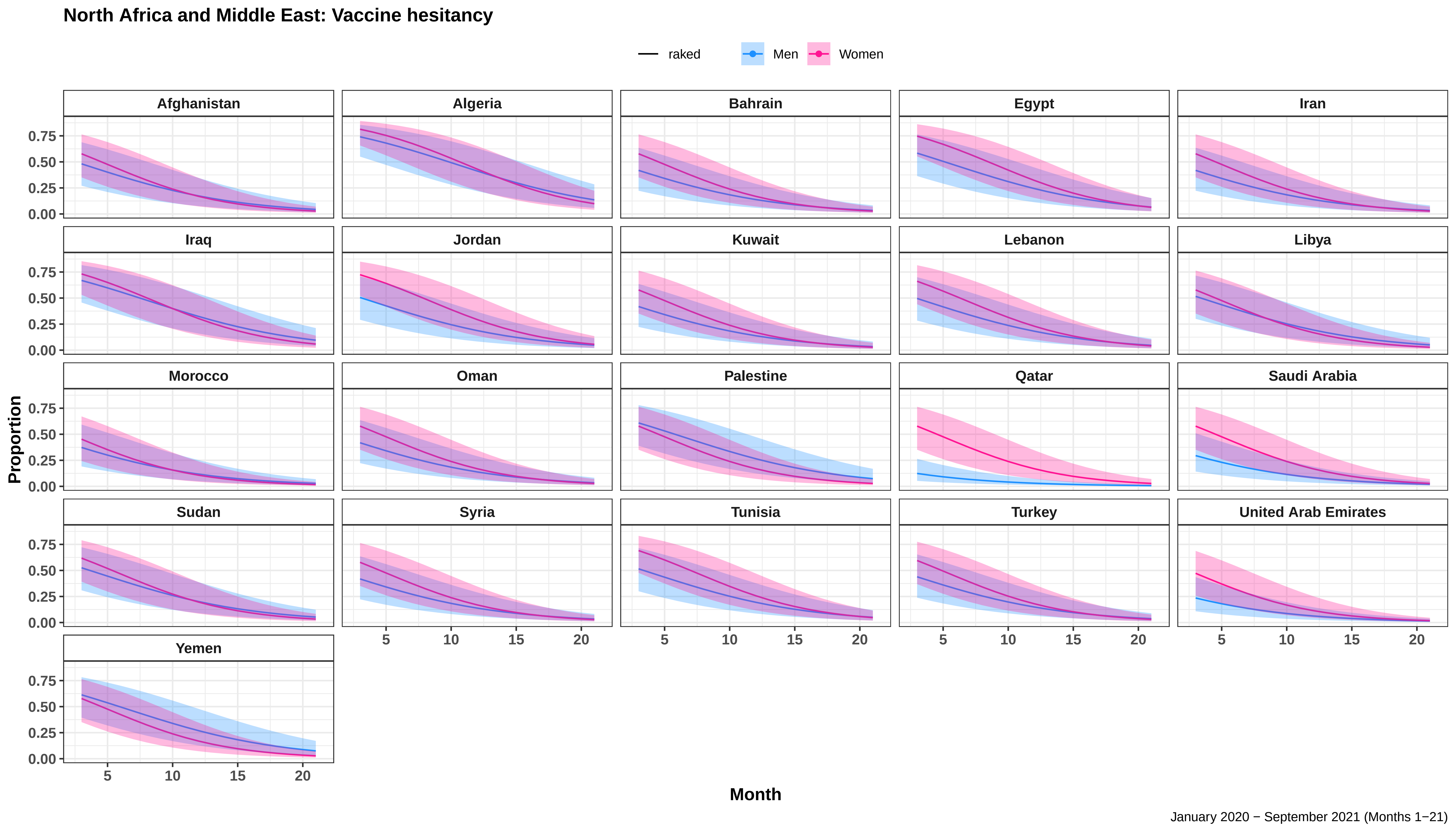

North Africa and Middle East: Vaccine hesitancy
raked
Men
Women
Afghanistan
Algeria
Bahrain
Egypt
Iran
Iraq
Jordan
Kuwait
Lebanon
Libya
Morocco
Oman
Palestine
Qatar
Saudi Arabia
Sudan
Syria
Tunisia
Turkey
United Arab Emirates
Yemen
Proportion
0.00
0.25
0.50
0.75
Month
5
10
15
20
January 2020 − September 2021 (Months 1−21)

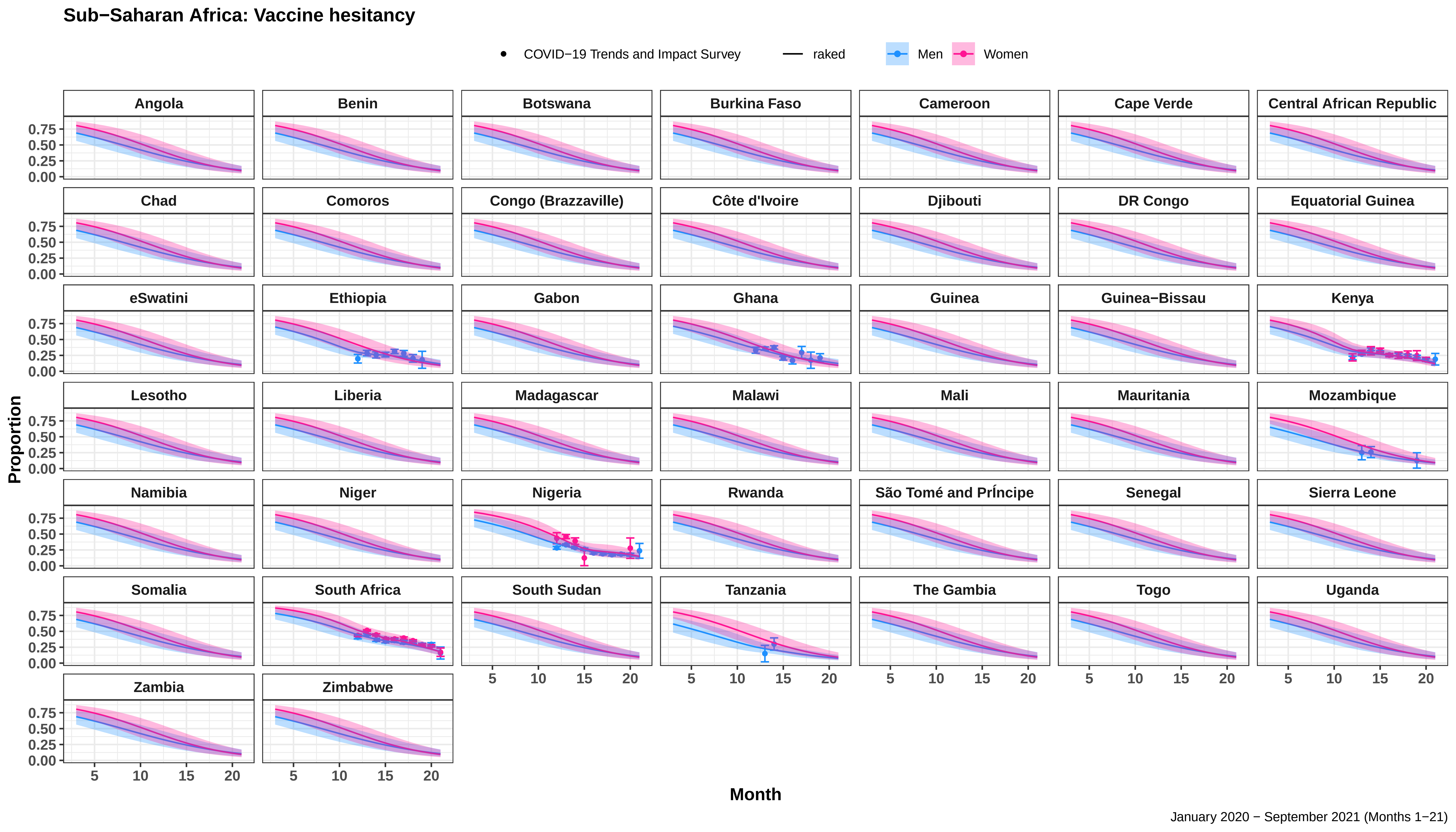

Sub−Saharan Africa: Vaccine hesitancy
COVID−19 Trends and Impact Survey
raked
Men
Women
Angola
Benin
Botswana
Burkina Faso
Cameroon
Cape Verde
Central African Republic
Chad
Comoros
Congo (Brazzaville)
Côte d'Ivoire
Djibouti
DR Congo
Equatorial Guinea
eSwatini
Ethiopia
Gabon
Ghana
Guinea
Guinea−Bissau
Kenya
Lesotho
Liberia
Madagascar
Malawi
Mali
Mauritania
Mozambique
Namibia
Niger
Nigeria
Rwanda
São Tomé and PrÍncipe
Senegal
Sierra Leone
Somalia
South Africa
South Sudan
Tanzania
The Gambia
Togo
Uganda
Zambia
Zimbabwe
Proportion
0.00
0.25
0.50
0.75
5
10
15
20
Month
January 2020 − September 2021 (Months 1−21)

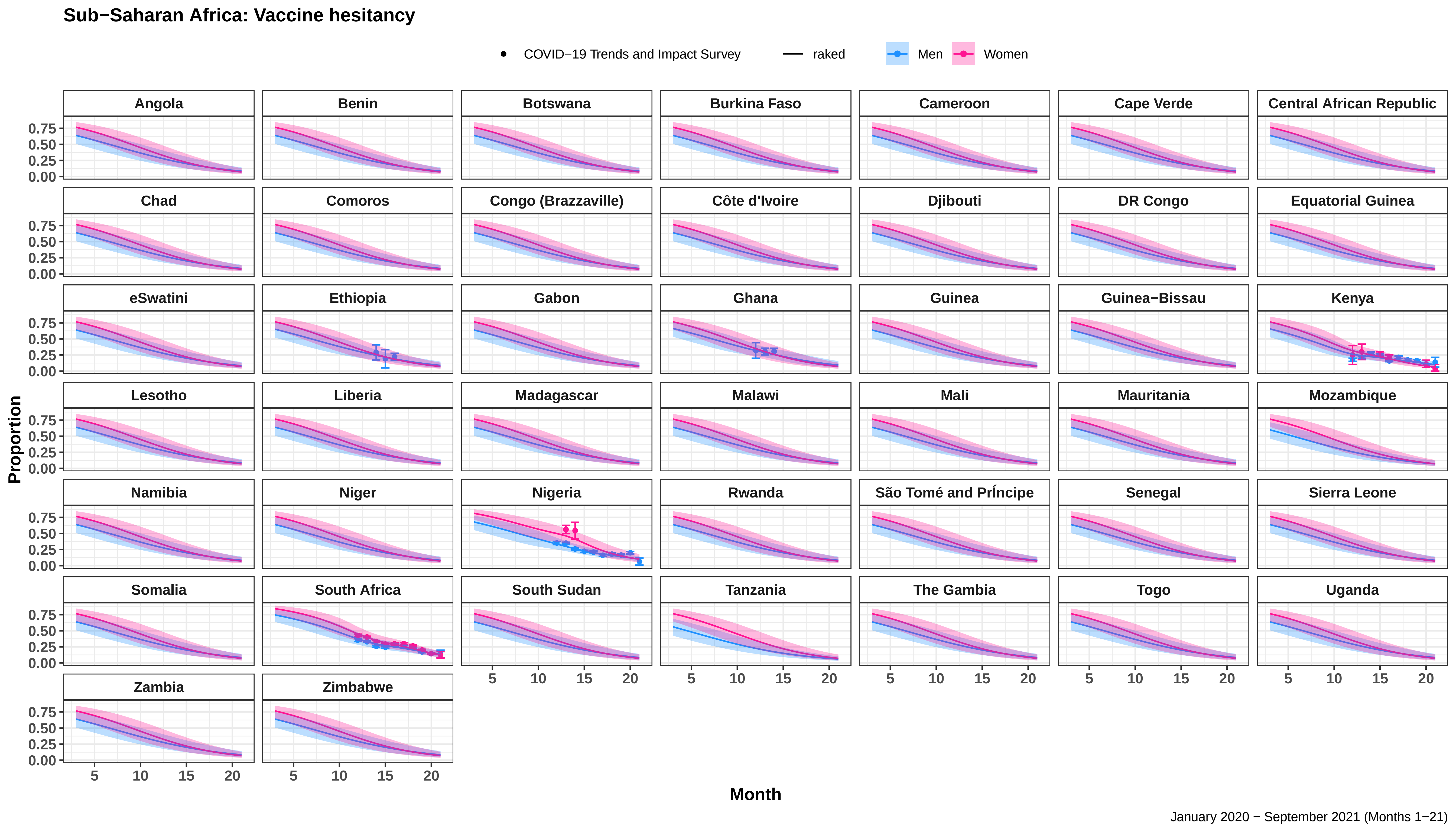

Sub−Saharan Africa: Vaccine hesitancy
COVID−19 Trends and Impact Survey
raked
Men
Women
Angola
Benin
Botswana
Burkina Faso
Cameroon
Cape Verde
Central African Republic
Chad
Comoros
Congo (Brazzaville)
Côte d'Ivoire
Djibouti
DR Congo
Equatorial Guinea
eSwatini
Ethiopia
Gabon
Ghana
Guinea
Guinea−Bissau
Kenya
Lesotho
Liberia
Madagascar
Malawi
Mali
Mauritania
Mozambique
Namibia
Niger
Nigeria
Rwanda
São Tomé and PrÍncipe
Senegal
Sierra Leone
Somalia
South Africa
South Sudan
Tanzania
The Gambia
Togo
Uganda
Zambia
Zimbabwe
Proportion
0.00
0.25
0.50
0.75
Month
5
10
15
20
January 2020 − September 2021 (Months 1−21)

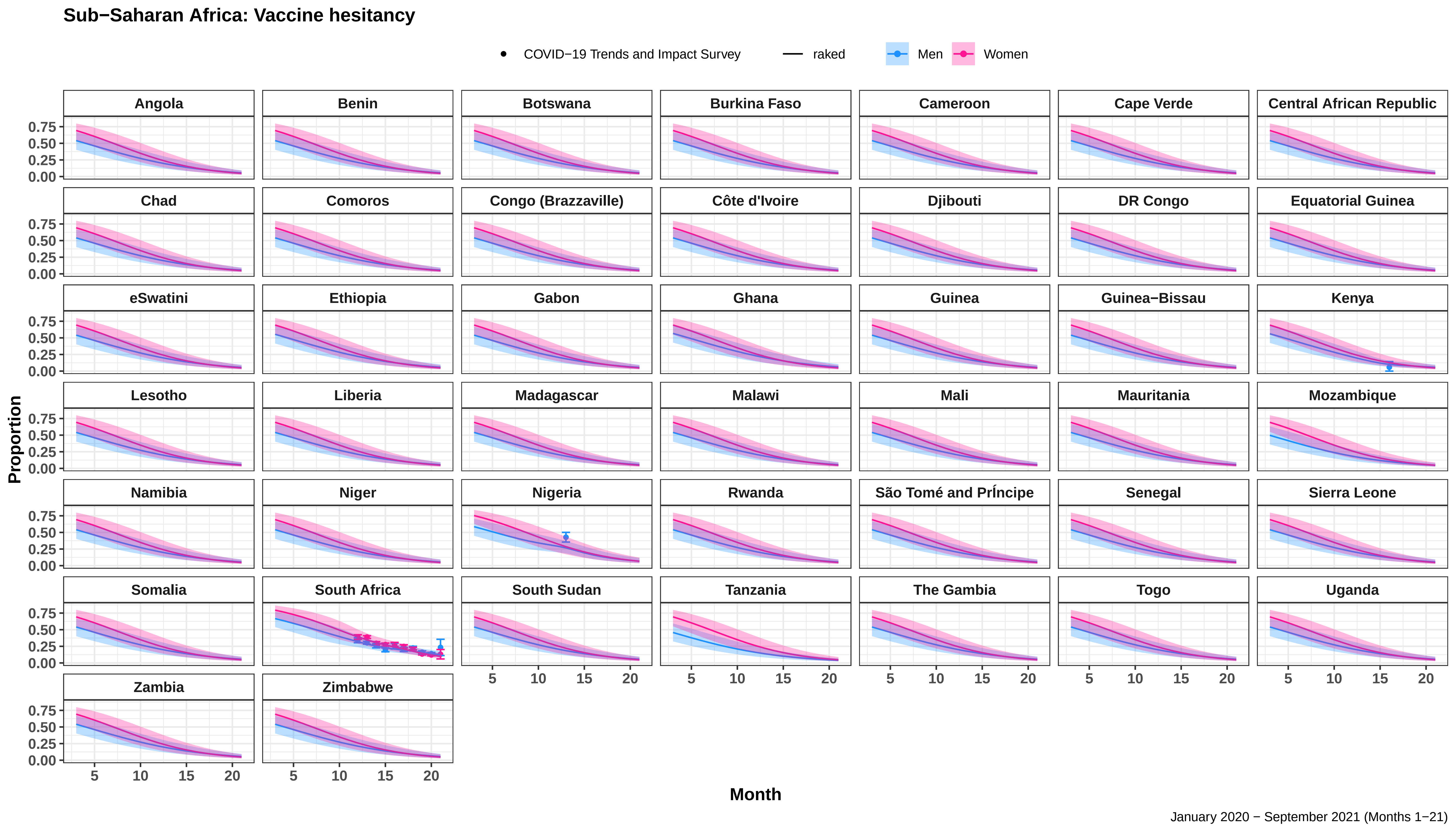
Sub−Saharan Africa: Vaccine hesitancy
COVID−19 Trends and Impact Survey
raked
Men
Women
Angola
Benin
Botswana
Burkina Faso
Cameroon
Cape Verde
Central African Republic
Chad
Comoros
Congo (Brazzaville)
Côte d'Ivoire
Djibouti
DR Congo
Equatorial Guinea
eSwatini
Ethiopia
Gabon
Ghana
Guinea
Guinea−Bissau
Kenya
Lesotho
Liberia
Madagascar
Malawi
Mali
Mauritania
Mozambique
Namibia
Niger
Nigeria
Rwanda
São Tomé and PrÍncipe
Senegal
Sierra Leone
Somalia
South Africa
South Sudan
Tanzania
The Gambia
Togo
Uganda
Zambia
Zimbabwe
Proportion
0.00
0.25
0.50
0.75
Month
5
10
15
20
January 2020 − September 2021 (Months 1−21)

Sub−Saharan Africa: Vaccine hesitancy

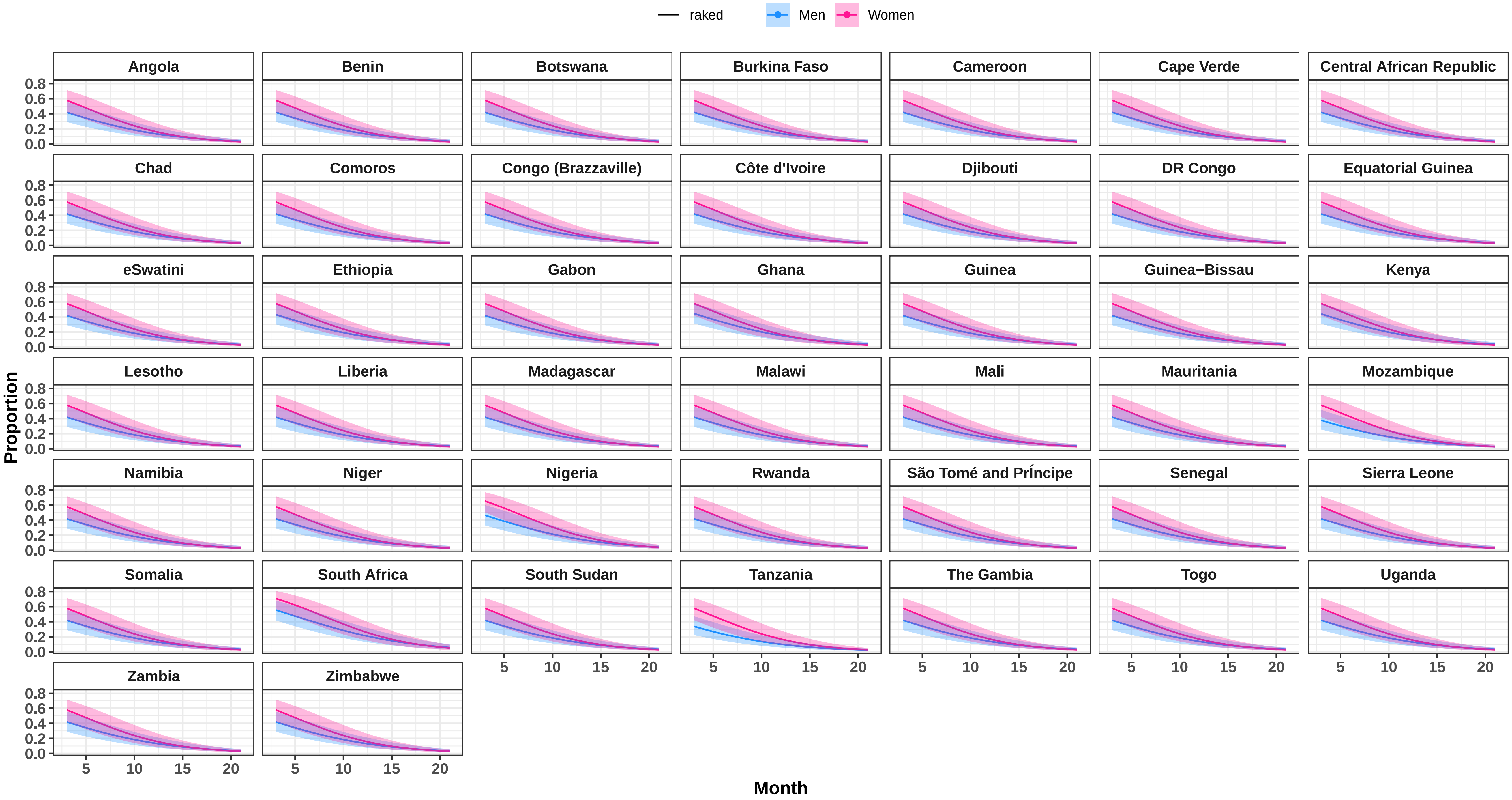

January 2020 − September 2021 (Months 1−21)

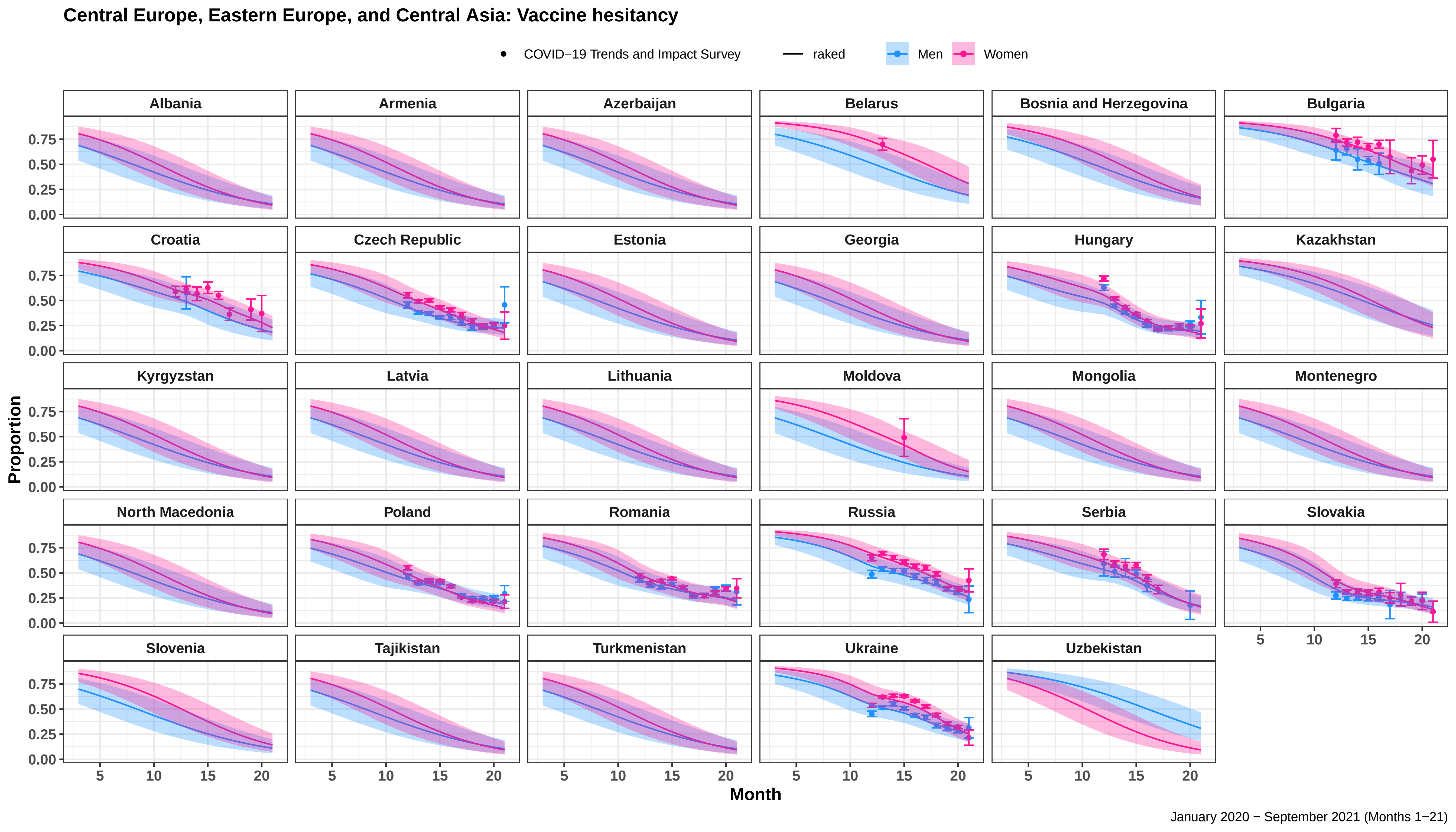

Central Europe, Eastern Europe, and Central Asia: Vaccine hesitancy
COVID−19 Trends and Impact Survey
raked
Men
Women
Albania
Armenia
Azerbaijan
Belarus
Bosnia and Herzegovina
Bulgaria
Croatia
Czech Republic
Estonia
Georgia
Hungary
Kazakhstan
Kyrgyzstan
Latvia
Lithuania
Moldova
Mongolia
Montenegro
North Macedonia
Poland
Romania
Russia
Serbia
Slovakia
Slovenia
Tajikistan
Turkmenistan
Ukraine
Uzbekistan
Proportion
Month
January 2020 − September 2021 (Months 1−21)

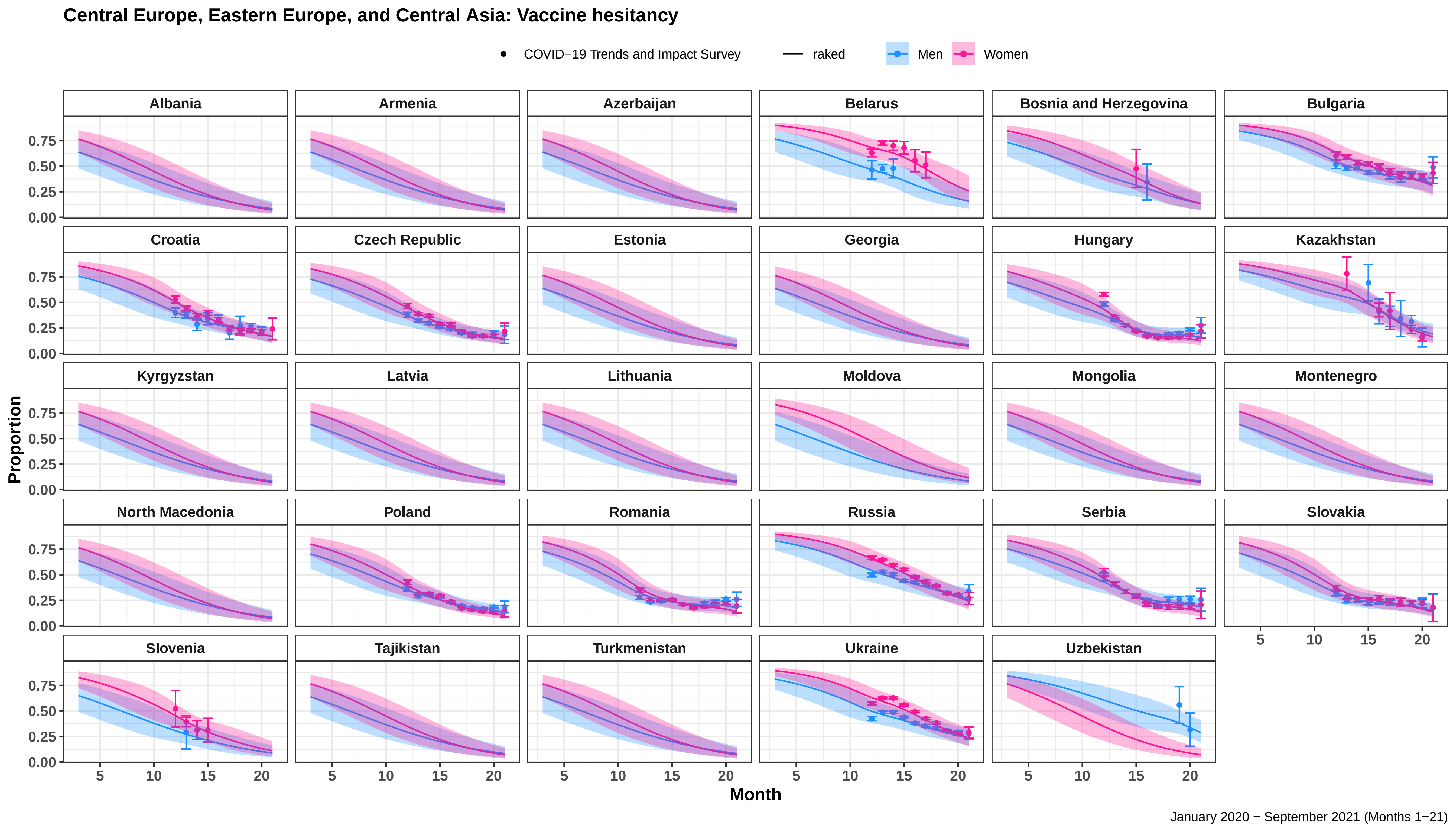
Central Europe, Eastern Europe, and Central Asia: Vaccine hesitancy
COVID−19 Trends and Impact Survey
raked
Men
Women
Albania
Armenia
Azerbaijan
Belarus
Bosnia and Herzegovina
Bulgaria
Croatia
Czech Republic
Estonia
Georgia
Hungary
Kazakhstan
Kyrgyzstan
Latvia
Lithuania
Moldova
Mongolia
Montenegro
North Macedonia
Poland
Romania
Russia
Serbia
Slovakia
Slovenia
Tajikistan
Turkmenistan
Ukraine
Uzbekistan
Proportion
0.00
0.25
0.50
0.75
Month
5
10
15
20
January 2020 − September 2021 (Months 1−21)

Central Europe, Eastern Europe, and Central Asia: Vaccine hesitancy

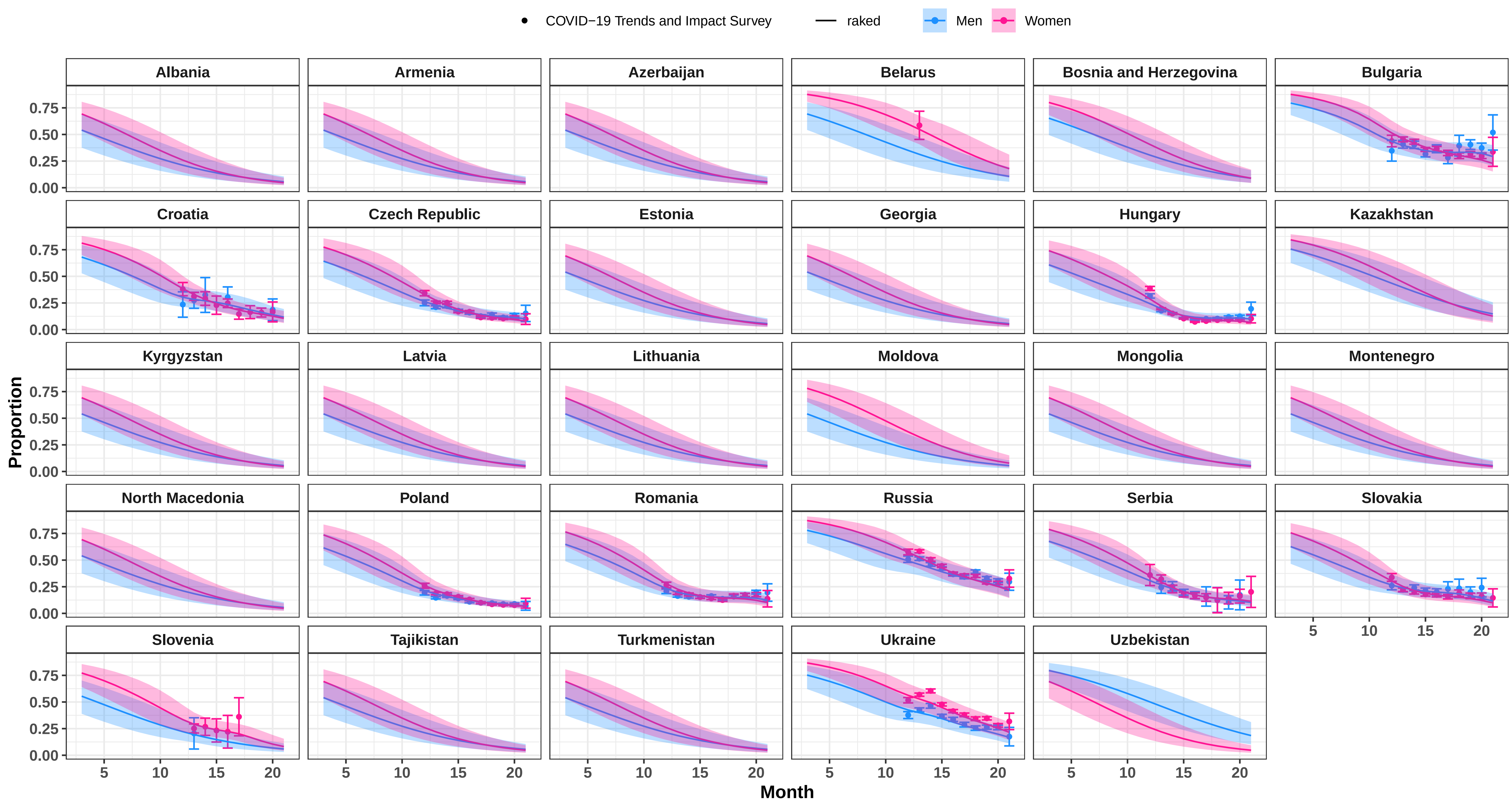

January 2020 − September 2021 (Months 1−21)

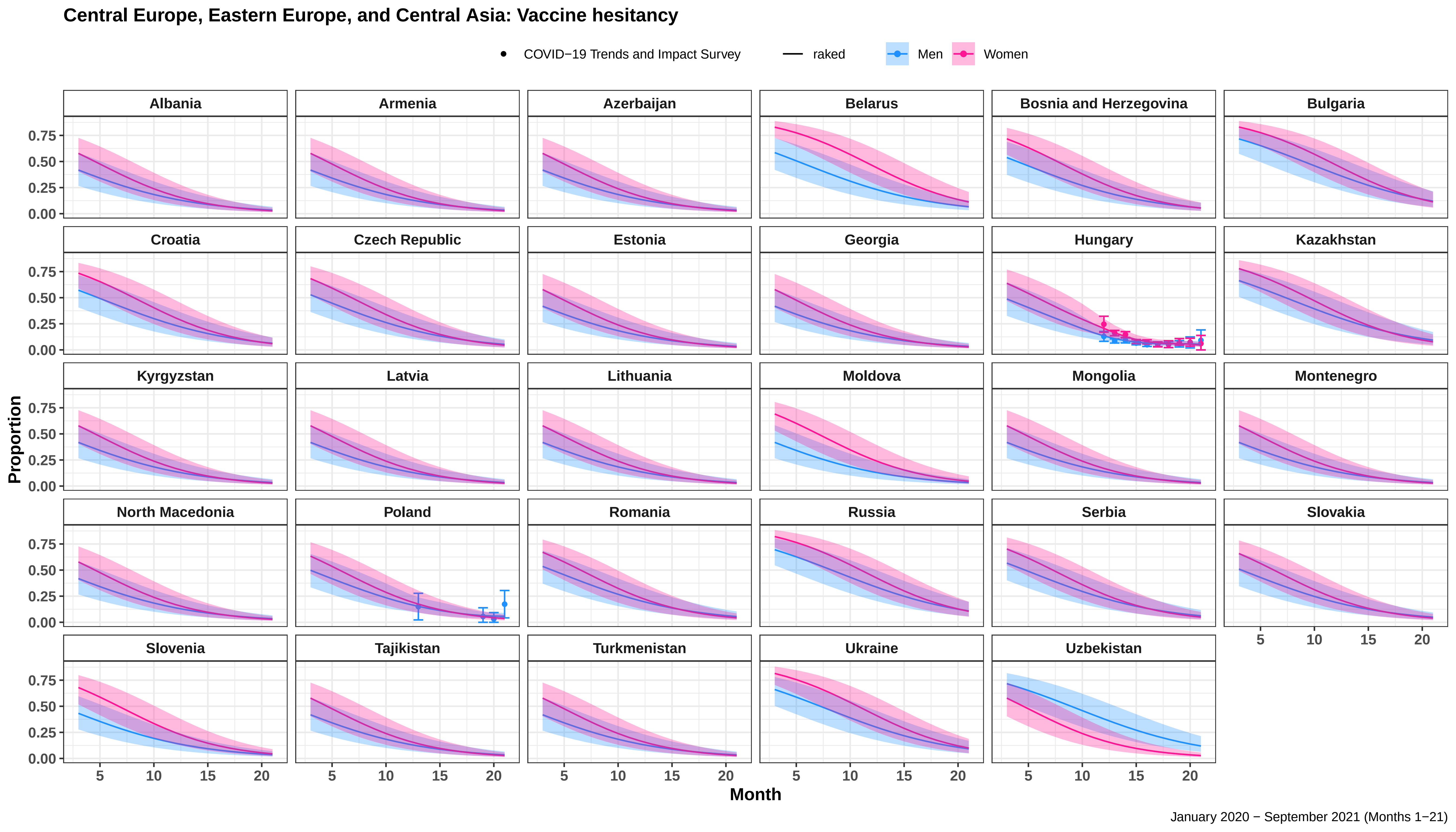
Central Europe, Eastern Europe, and Central Asia: Vaccine hesitancy
COVID−19 Trends and Impact Survey
raked
Men
Women
Albania
Armenia
Azerbaijan
Belarus
Bosnia and Herzegovina
Bulgaria
Croatia
Czech Republic
Estonia
Georgia
Hungary
Kazakhstan
Kyrgyzstan
Latvia
Lithuania
Moldova
Mongolia
Montenegro
North Macedonia
Poland
Romania
Russia
Serbia
Slovakia
Slovenia
Tajikistan
Turkmenistan
Ukraine
Uzbekistan
Proportion
0.00
0.25
0.50
0.75
Month
5
10
15
20
January 2020 − September 2021 (Months 1−21)

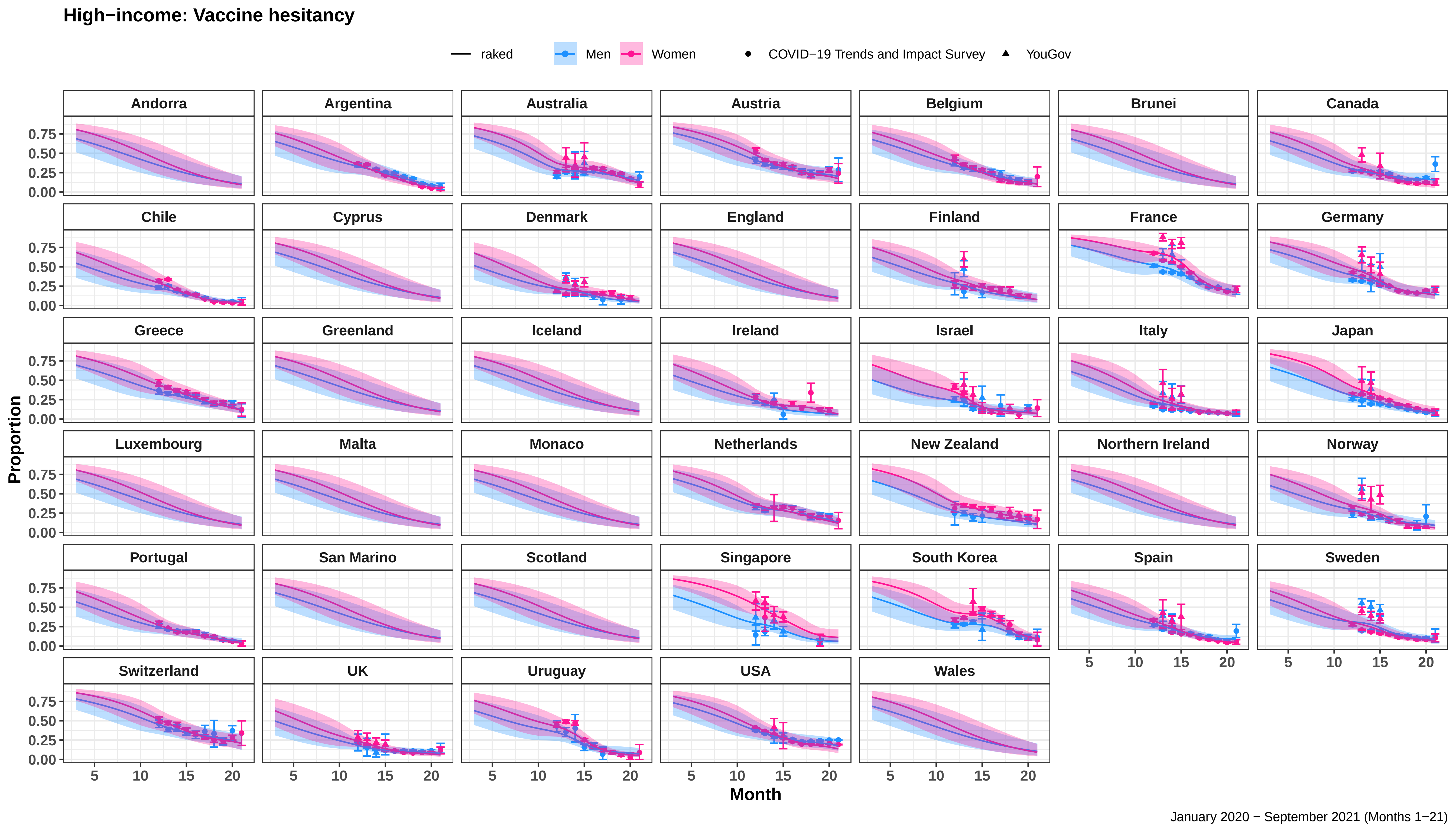

High−income: Vaccine hesitancy
raked
Men
Women
COVID−19 Trends and Impact Survey
YouGov
Andorra
Argentina
Australia
Austria
Belgium
Brunei
Canada
Chile
Cyprus
Denmark
England
Finland
France
Germany
Greece
Greenland
Iceland
Ireland
Israel
Italy
Japan
Luxembourg
Malta
Monaco
Netherlands
New Zealand
Northern Ireland
Norway
Portugal
San Marino
Scotland
Singapore
South Korea
Spain
Sweden
Switzerland
UK
Uruguay
USA
Wales
Proportion
0.00
0.25
0.50
0.75
5
10
15
20
Month
January 2020 − September 2021 (Months 1−21)

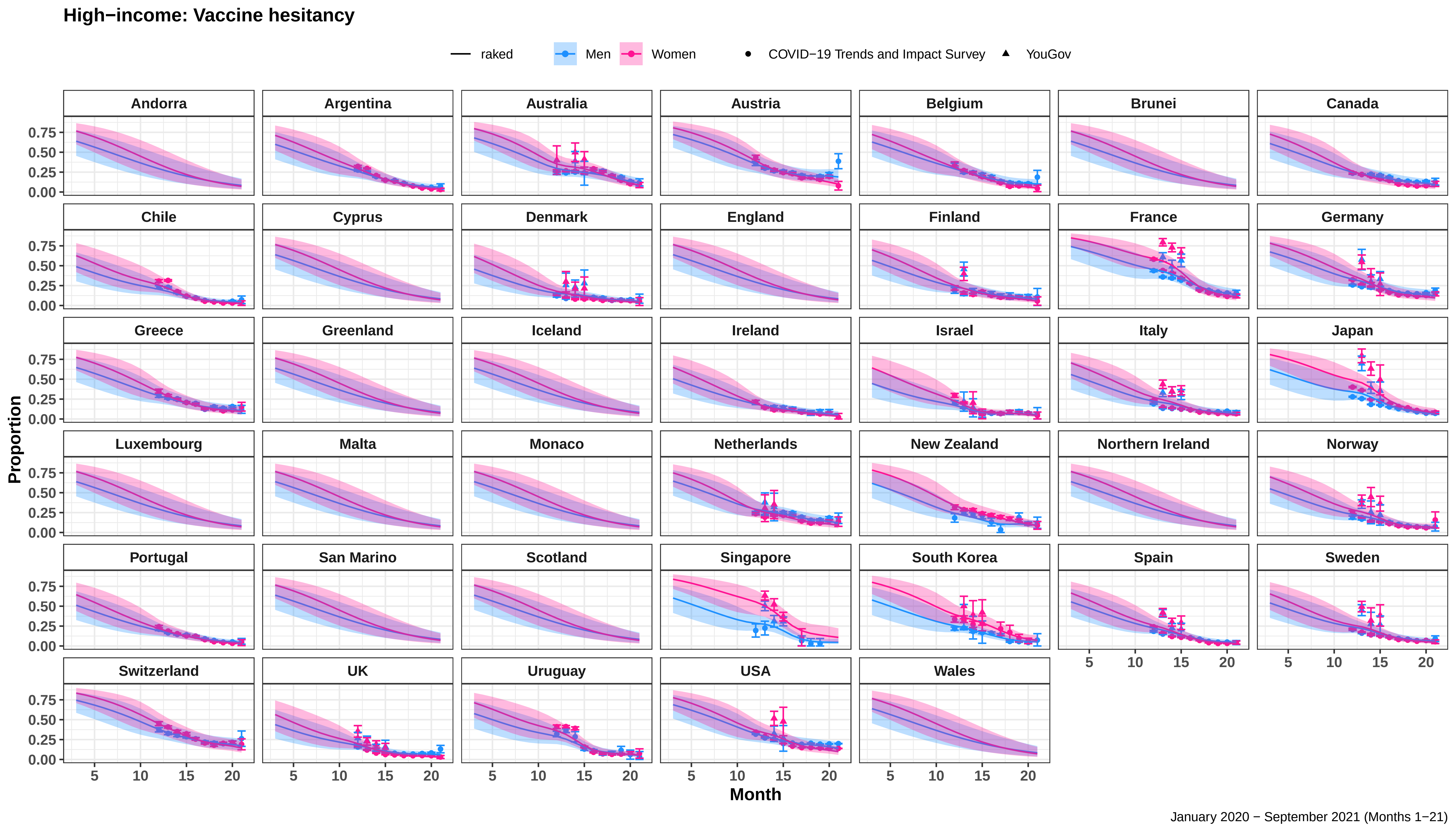

High−income: Vaccine hesitancy
raked
Men
Women
COVID−19 Trends and Impact Survey
YouGov
Andorra
Argentina
Australia
Austria
Belgium
Brunei
Canada
Chile
Cyprus
Denmark
England
Finland
France
Germany
Greece
Greenland
Iceland
Ireland
Israel
Italy
Japan
Luxembourg
Malta
Monaco
Netherlands
New Zealand
Northern Ireland
Norway
Portugal
San Marino
Scotland
Singapore
South Korea
Spain
Sweden
Switzerland
UK
Uruguay
USA
Wales
Proportion
0.00
0.25
0.50
0.75
Month
5
10
15
20
January 2020 − September 2021 (Months 1−21)

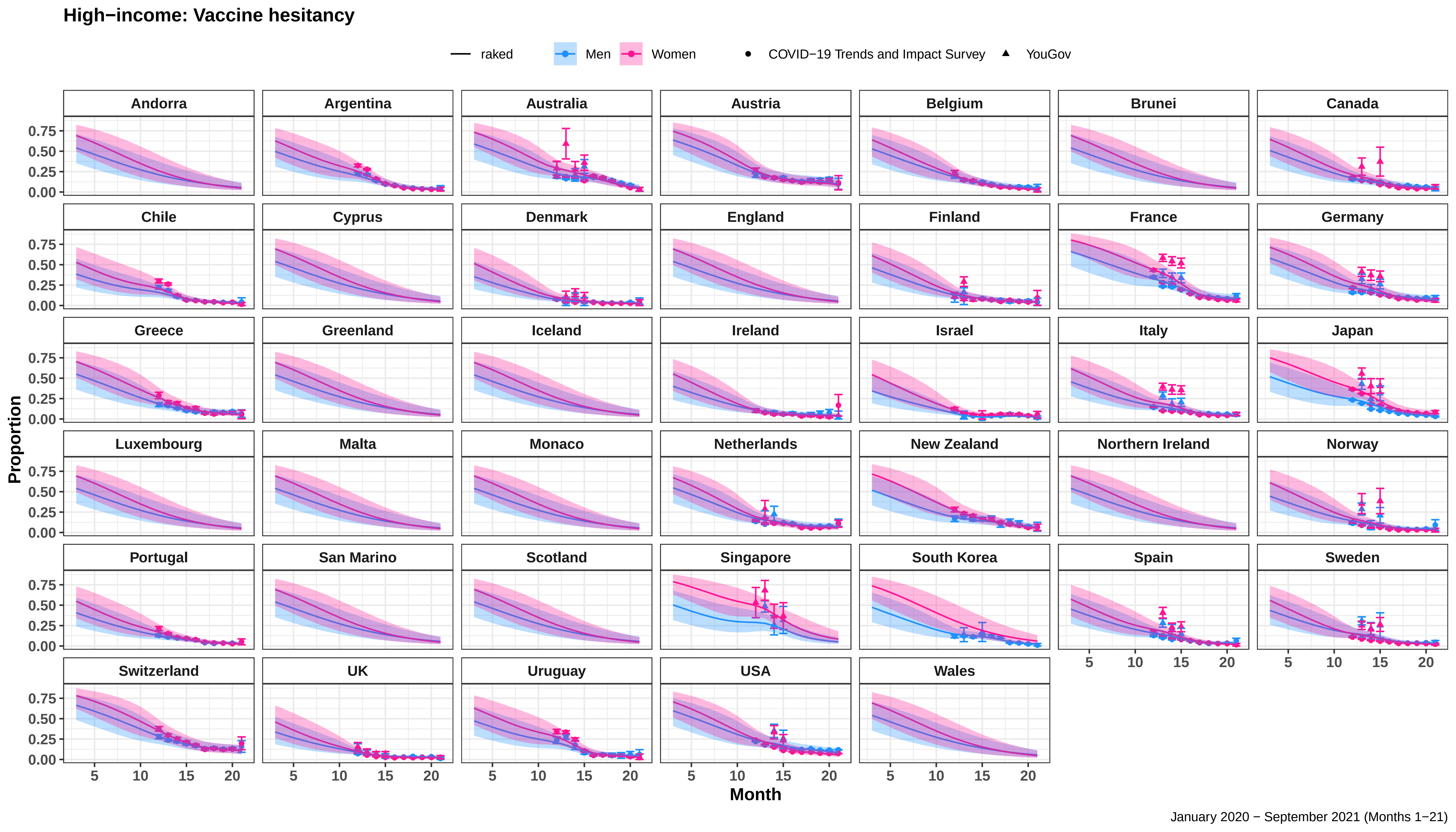
High−income: Vaccine hesitancy
raked
Men
Women
COVID−19 Trends and Impact Survey
YouGov
Andorra
Argentina
Australia
Austria
Belgium
Brunei
Canada
Chile
Cyprus
Denmark
England
Finland
France
Germany
Greece
Greenland
Iceland
Ireland
Israel
Italy
Japan
Luxembourg
Malta
Monaco
Netherlands
New Zealand
Northern Ireland
Norway
Portugal
San Marino
Scotland
Singapore
South Korea
Spain
Sweden
Switzerland
UK
Uruguay
USA
Wales
Proportion
0.00
0.25
0.50
0.75
Month
5
10
15
20
January 2020 − September 2021 (Months 1−21)

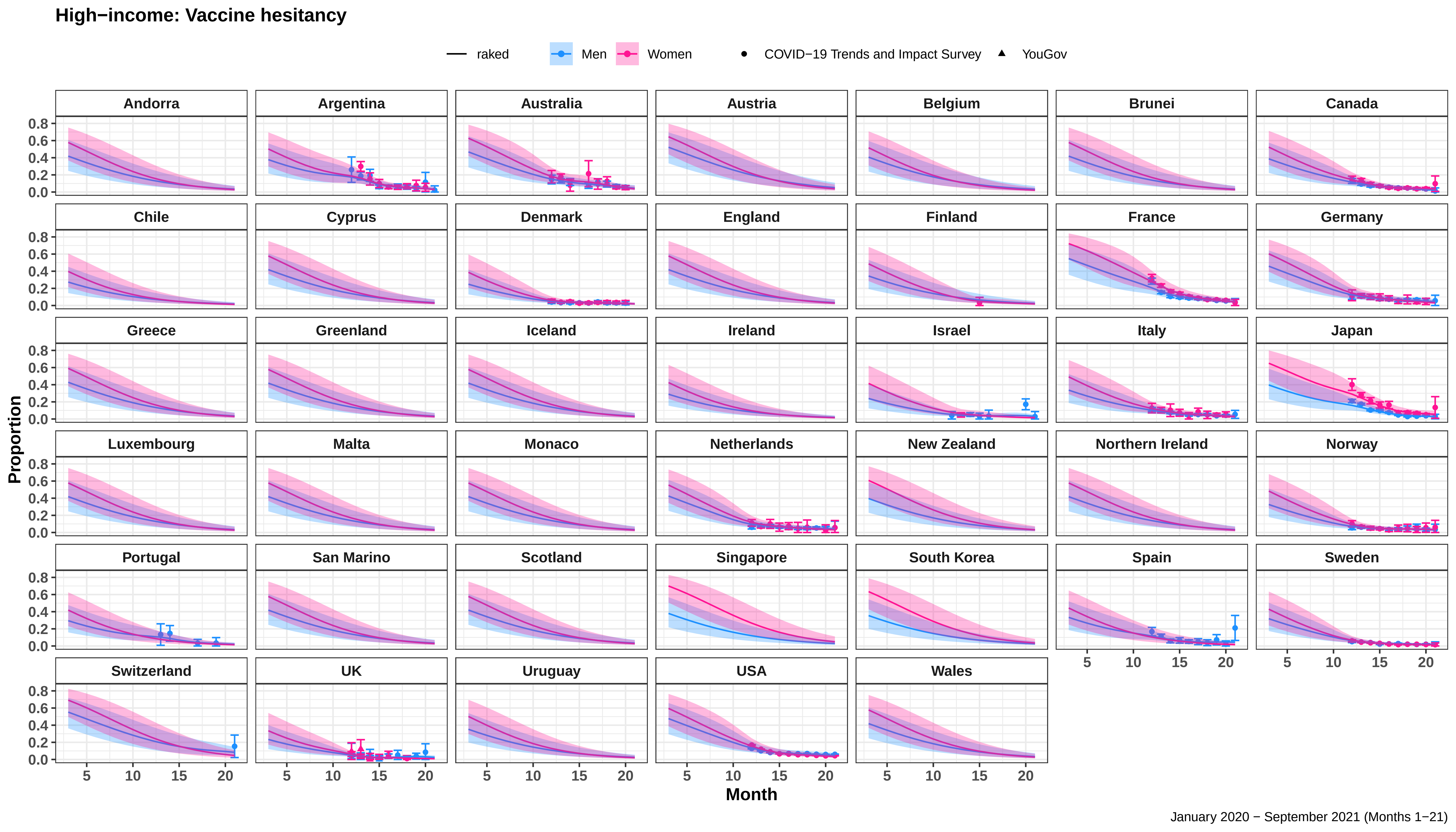

High−income: Vaccine hesitancy
raked
Men
Women
COVID−19 Trends and Impact Survey
YouGov
Andorra
Argentina
Australia
Austria
Belgium
Brunei
Canada
Chile
Cyprus
Denmark
England
Finland
France
Germany
Greece
Greenland
Iceland
Ireland
Israel
Italy
Japan
Luxembourg
Malta
Monaco
Netherlands
New Zealand
Northern Ireland
Norway
Portugal
San Marino
Scotland
Singapore
South Korea
Spain
Sweden
Switzerland
UK
Uruguay
USA
Wales
Proportion
0.0
0.2
0.4
0.6
0.8
Month
5
10
15
20
January 2020 − September 2021 (Months 1−21)

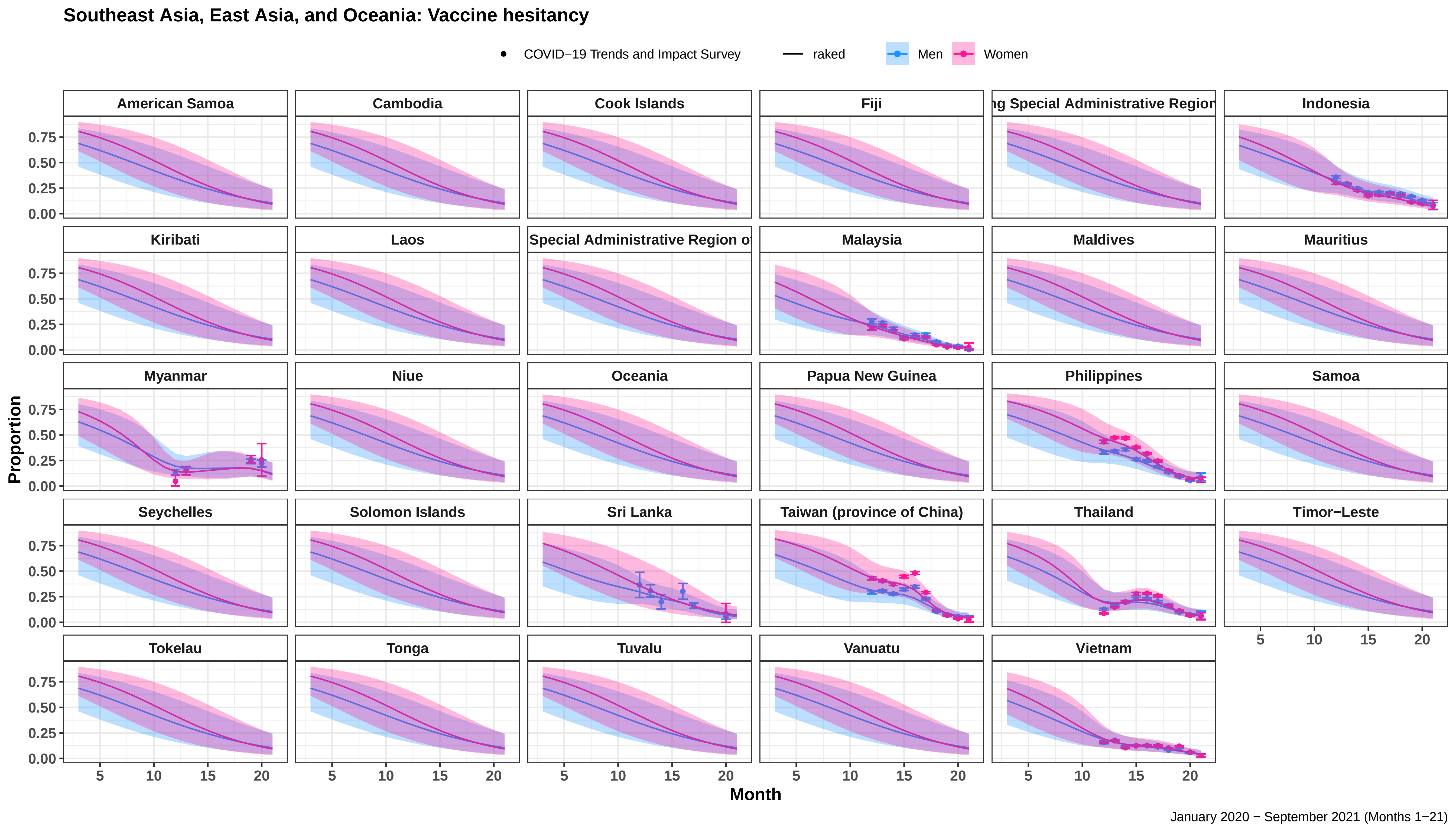

Southeast Asia, East Asia, and Oceania: Vaccine hesitancy
COVID−19 Trends and Impact Survey
raked
Men
Women
American Samoa
Cambodia
Cook Islands
Fiji
ng Special Administrative Region
Indonesia
Kiribati
Laos
Special Administrative Region of
Malaysia
Maldives
Mauritius
Myanmar
Niue
Oceania
Papua New Guinea
Philippines
Samoa
Seychelles
Solomon Islands
Sri Lanka
Taiwan (province of China)
Thailand
Timor−Leste
Tokelau
Tonga
Tuvalu
Vanuatu
Vietnam
Proportion
Month
0.00
0.25
0.50
0.75
5
10
15
20
January 2020 − September 2021 (Months 1−21)

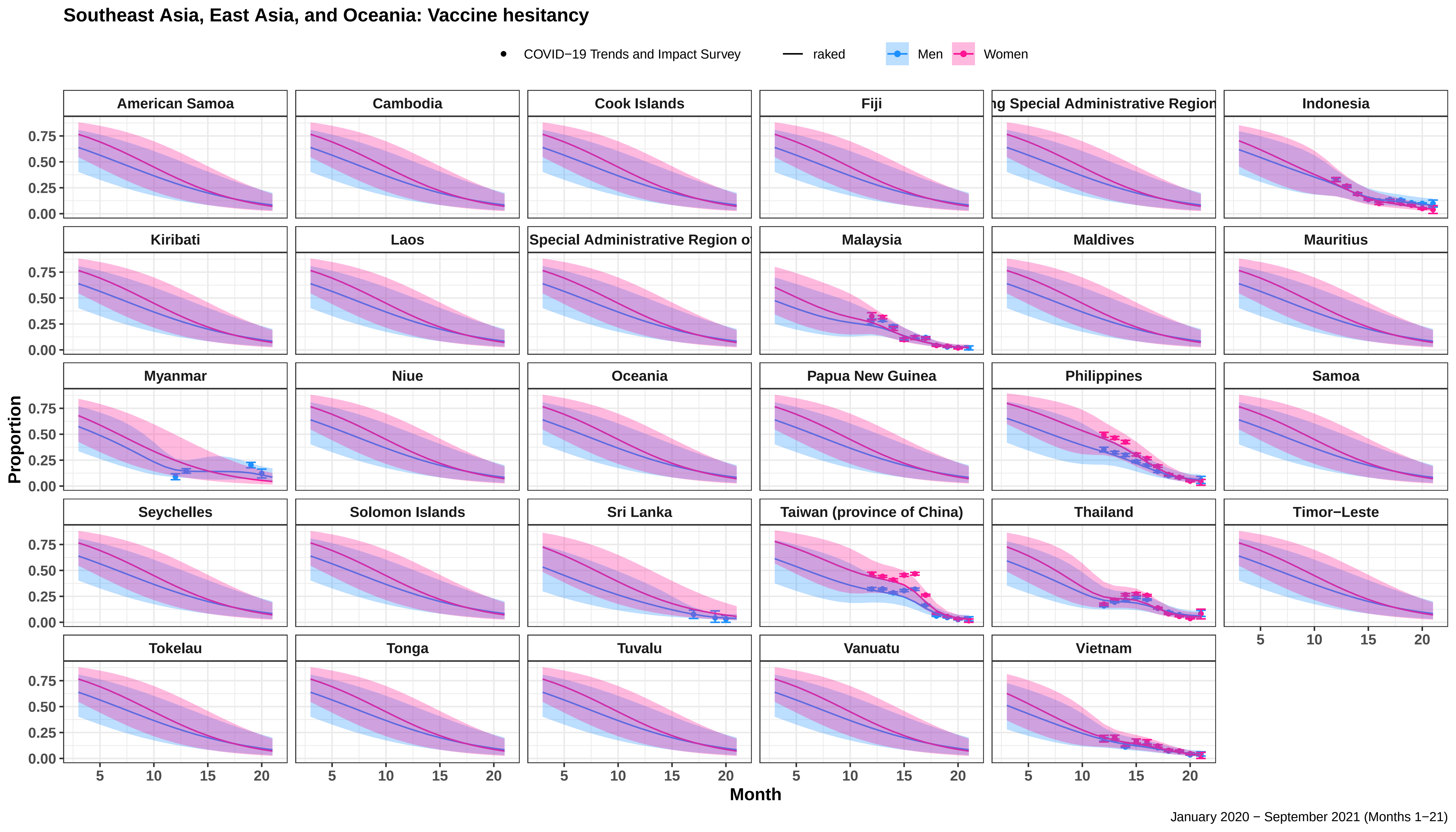
Southeast Asia, East Asia, and Oceania: Vaccine hesitancy
COVID−19 Trends and Impact Survey
raked
Men
Women
American Samoa
Cambodia
Cook Islands
Fiji
ng Special Administrative Region
Indonesia
Kiribati
Laos
Special Administrative Region of
Malaysia
Maldives
Mauritius
Myanmar
Niue
Oceania
Papua New Guinea
Philippines
Samoa
Seychelles
Solomon Islands
Sri Lanka
Taiwan (province of China)
Thailand
Timor−Leste
Tokelau
Tonga
Tuvalu
Vanuatu
Vietnam
Proportion
Month
0.00
0.25
0.50
0.75
5
10
15
20
January 2020 − September 2021 (Months 1−21)

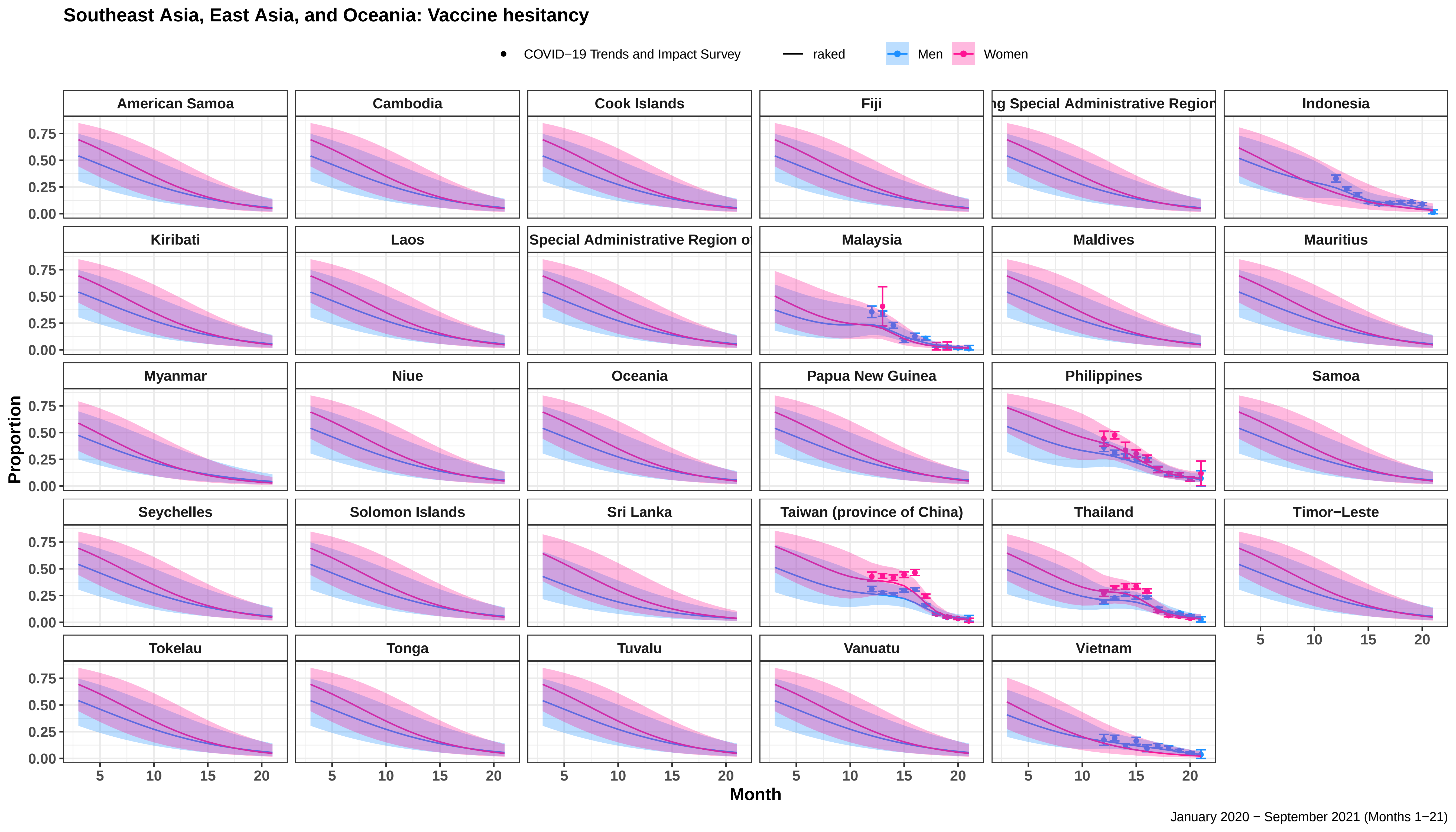

Southeast Asia, East Asia, and Oceania: Vaccine hesitancy
COVID−19 Trends and Impact Survey
raked
Men
Women
American Samoa
Cambodia
Cook Islands
Fiji
ng Special Administrative Region
Indonesia
Kiribati
Laos
Special Administrative Region o
Malaysia
Maldives
Mauritius
Myanmar
Niue
Oceania
Papua New Guinea
Philippines
Samoa
Seychelles
Solomon Islands
Sri Lanka
Taiwan (province of China)
Thailand
Timor−Leste
Tokelau
Tonga
Tuvalu
Vanuatu
Vietnam
Proportion
0.00
0.25
0.50
0.75
Month
5
10
15
20
January 2020 − September 2021 (Months 1−21)

Southeast Asia, East Asia, and Oceania: Vaccine hesitancy

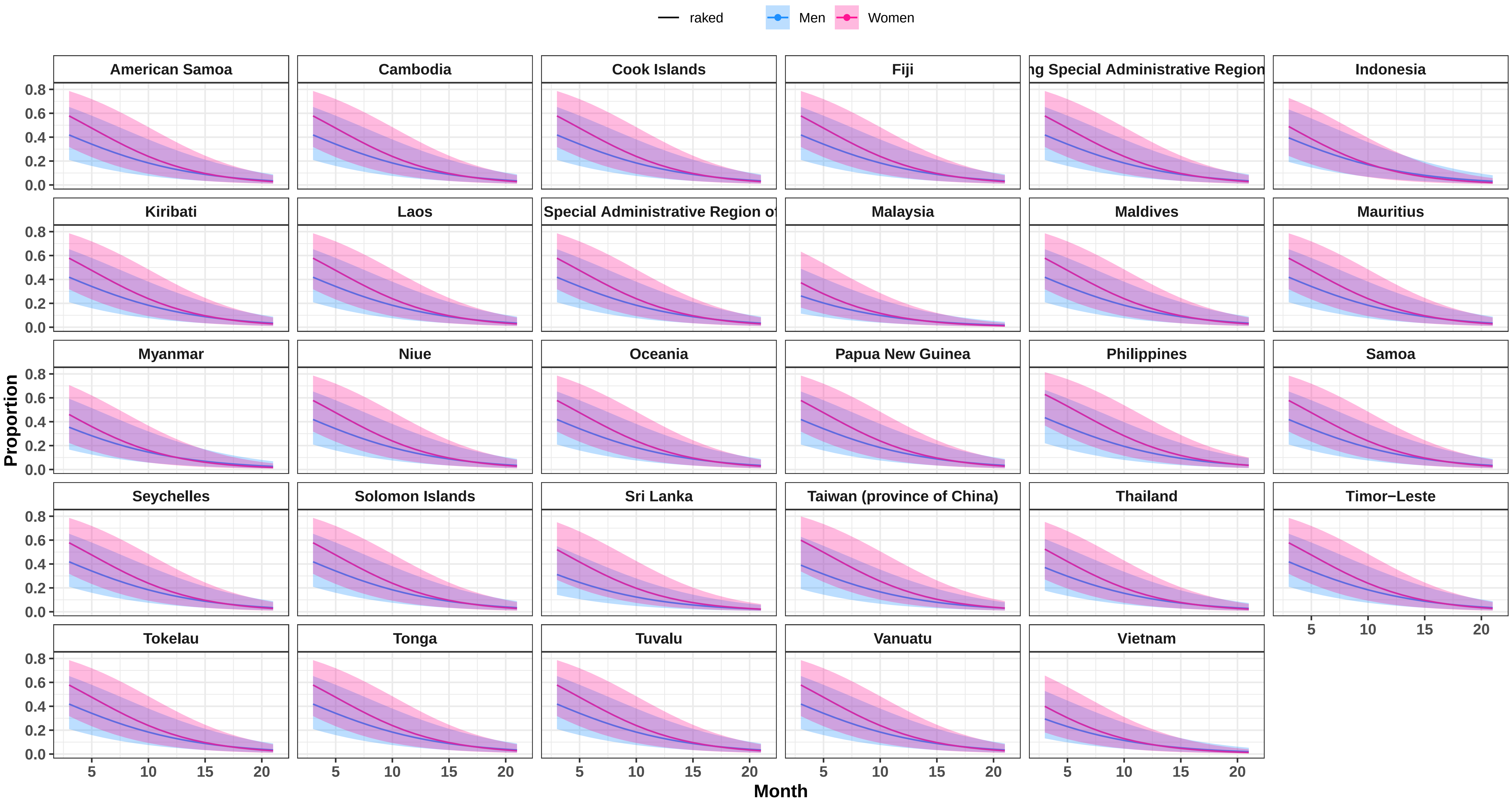

January 2020 − September 2021 (Months 1−21)

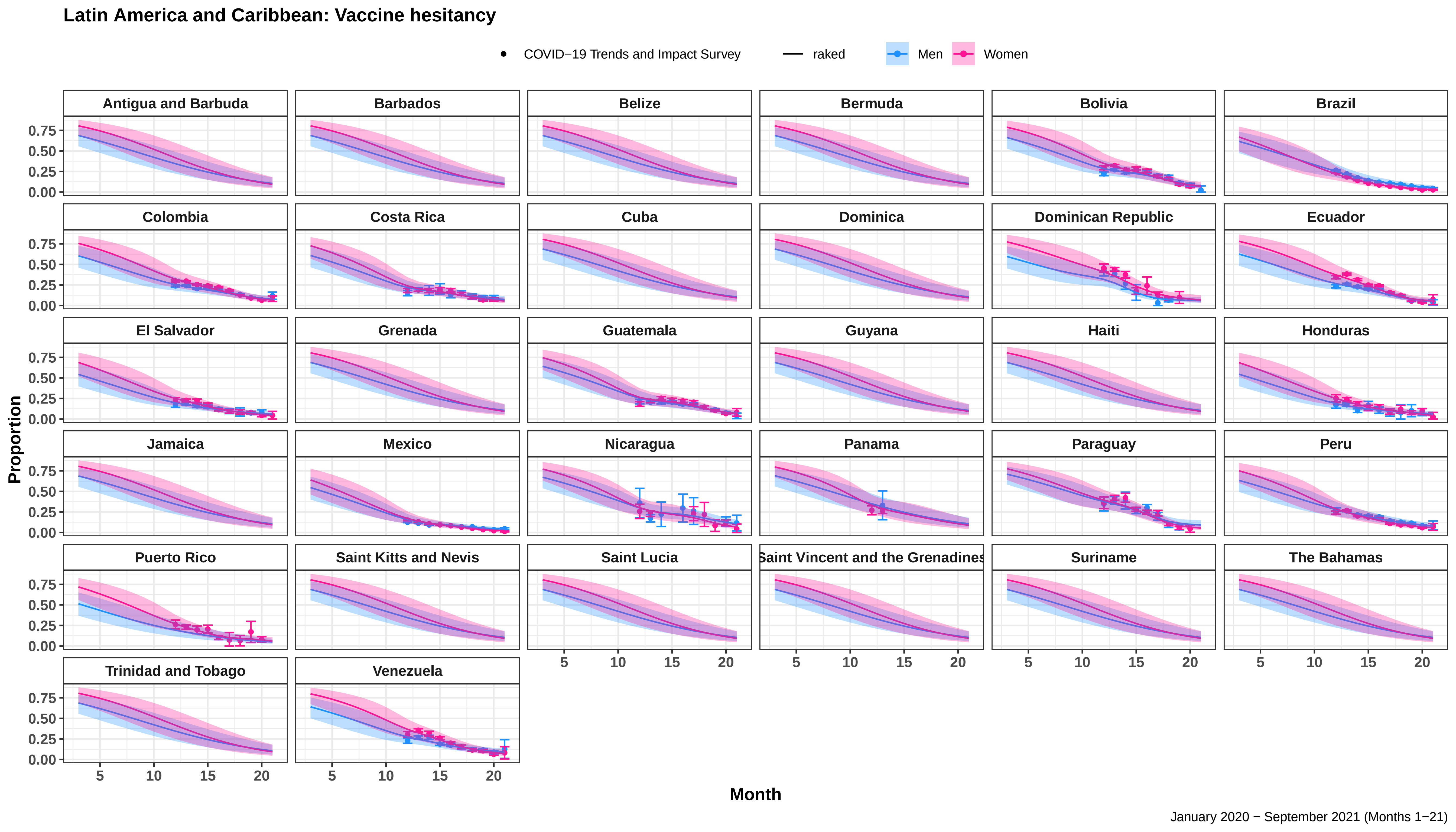

Latin America and Caribbean: Vaccine hesitancy
COVID−19 Trends and Impact Survey
raked
Men
Women
Antigua and Barbuda
Barbados
Belize
Bermuda
Bolivia
Brazil
Colombia
Costa Rica
Cuba
Dominica
Dominican Republic
Ecuador
El Salvador
Grenada
Guatemala
Guyana
Haiti
Honduras
Jamaica
Mexico
Nicaragua
Panama
Paraguay
Peru
Puerto Rico
Saint Kitts and Nevis
Saint Lucia
Saint Vincent and the Grenadines
Suriname
The Bahamas
Trinidad and Tobago
Venezuela
Proportion
0.00
0.25
0.50
0.75
Month
5
10
15
20
January 2020 − September 2021 (Months 1−21)

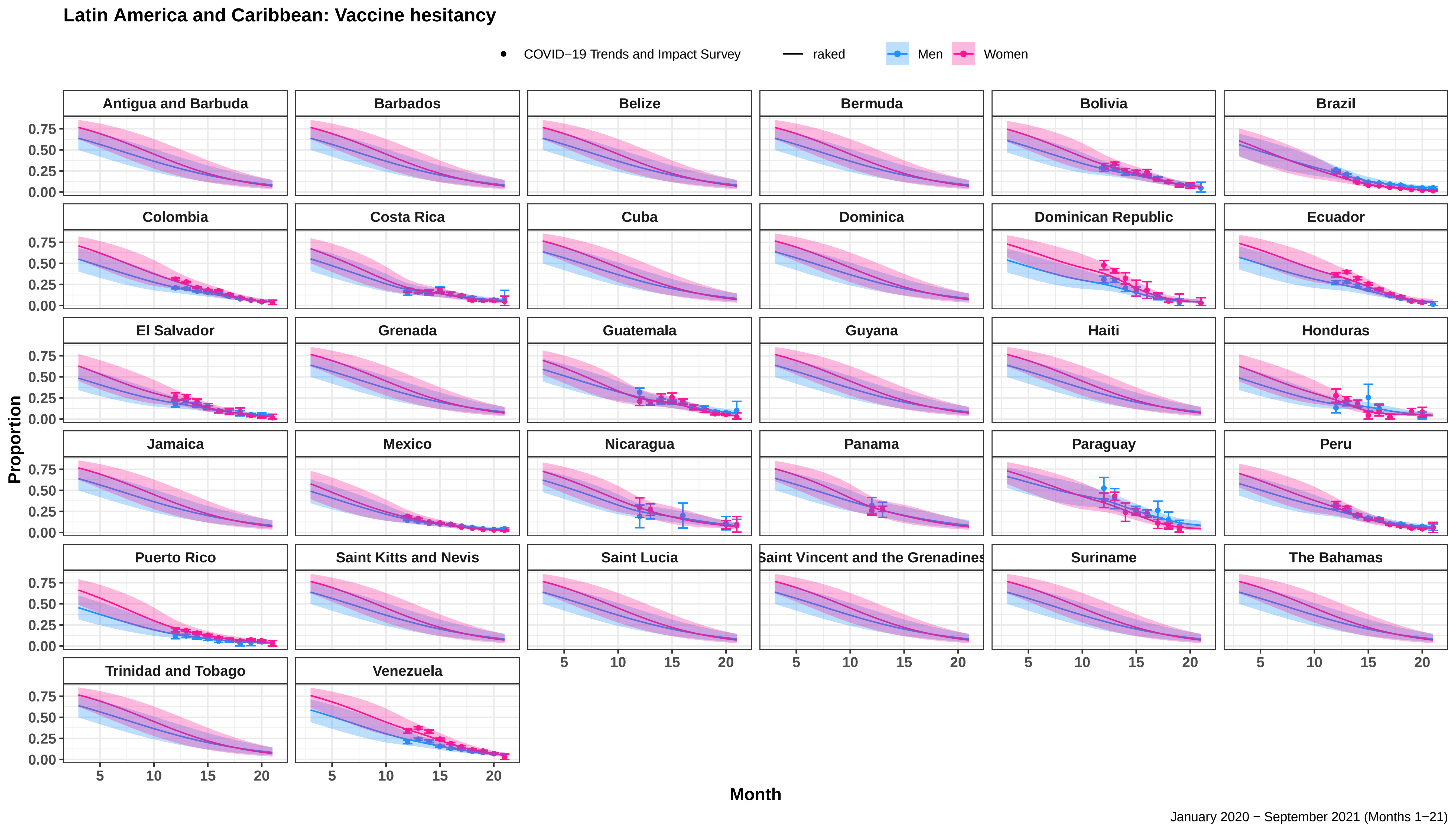
Latin America and Caribbean: Vaccine hesitancy
COVID−19 Trends and Impact Survey
raked
Men
Women
Antigua and Barbuda
Barbados
Belize
Bermuda
Bolivia
Brazil
Colombia
Costa Rica
Cuba
Dominica
Dominican Republic
Ecuador
El Salvador
Grenada
Guatemala
Guyana
Haiti
Honduras
Jamaica
Mexico
Nicaragua
Panama
Paraguay
Peru
Puerto Rico
Saint Kitts and Nevis
Saint Lucia
Saint Vincent and the Grenadines
Suriname
The Bahamas
Trinidad and Tobago
Venezuela
Proportion
Month
0.00
0.25
0.50
0.75
5
10
15
20
January 2020 − September 2021 (Months 1−21)

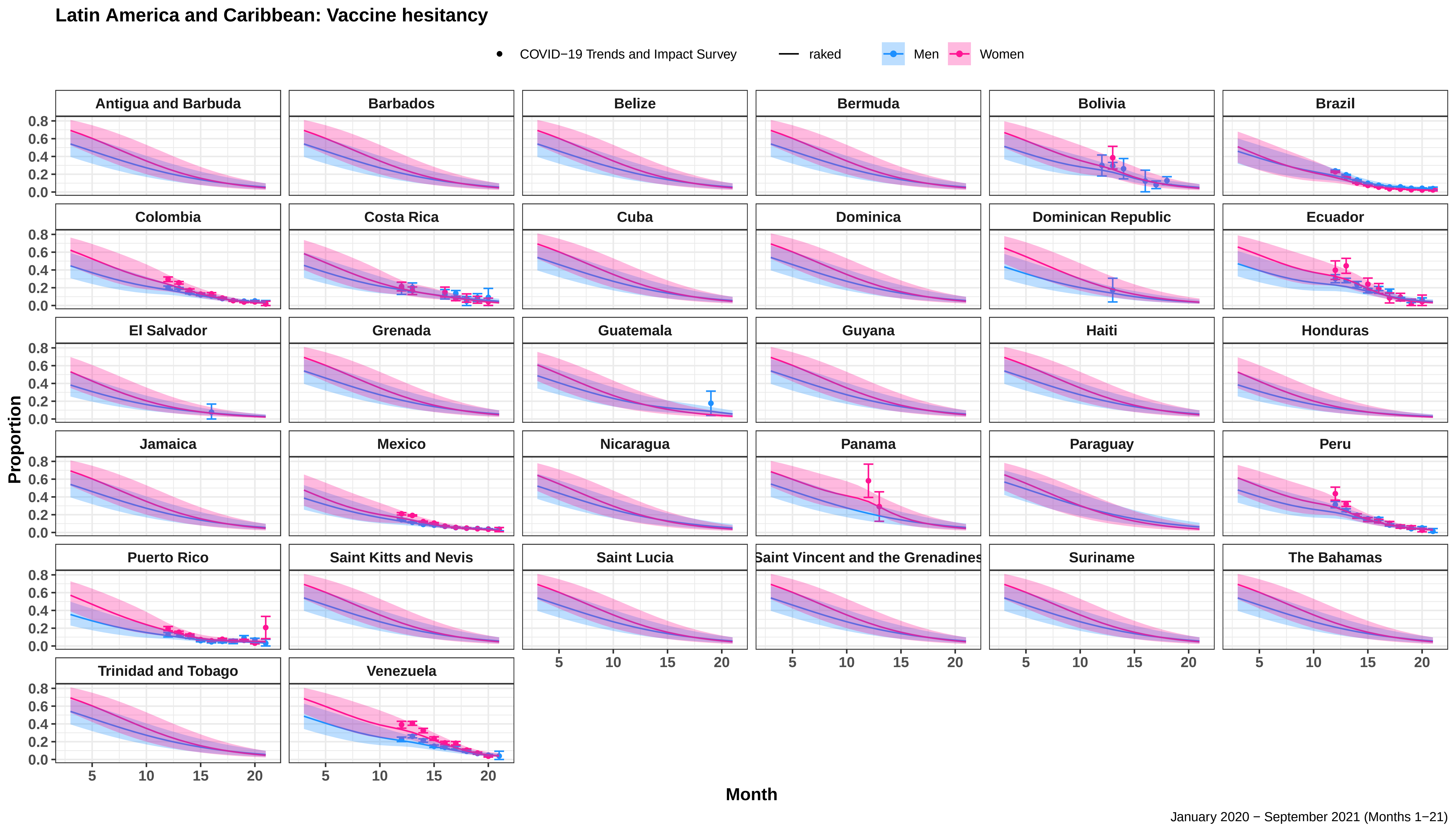
Latin America and Caribbean: Vaccine hesitancy
COVID−19 Trends and Impact Survey
raked
Men
Women
Antigua and Barbuda
Barbados
Belize
Bermuda
Bolivia
Brazil
Colombia
Costa Rica
Cuba
Dominica
Dominican Republic
Ecuador
El Salvador
Grenada
Guatemala
Guyana
Haiti
Honduras
Jamaica
Mexico
Nicaragua
Panama
Paraguay
Peru
Puerto Rico
Saint Kitts and Nevis
Saint Lucia
Saint Vincent and the Grenadines
Suriname
The Bahamas
Trinidad and Tobago
Venezuela
Proportion
Month
January 2020 − September 2021 (Months 1−21)

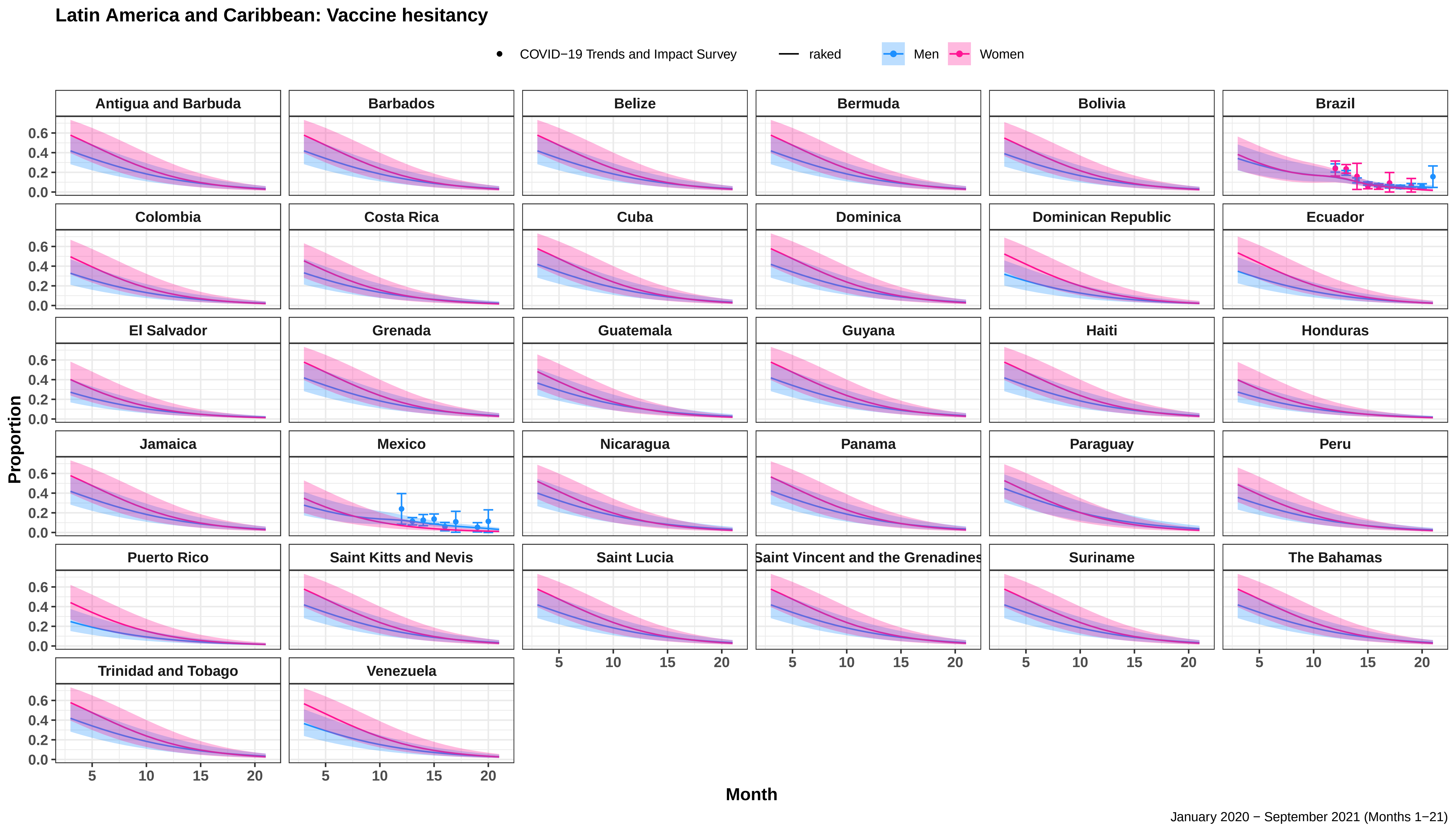

Latin America and Caribbean: Vaccine hesitancy
COVID−19 Trends and Impact Survey
raked
Men
Women
Antigua and Barbuda
Barbados
Belize
Bermuda
Bolivia
Brazil
Colombia
Costa Rica
Cuba
Dominica
Dominican Republic
Ecuador
El Salvador
Grenada
Guatemala
Guyana
Haiti
Honduras
Jamaica
Mexico
Nicaragua
Panama
Paraguay
Peru
Puerto Rico
Saint Kitts and Nevis
Saint Lucia
Saint Vincent and the Grenadines
Suriname
The Bahamas
Trinidad and Tobago
Venezuela
Proportion
0.0
0.2
0.4
0.6
5
10
15
20
Month
January 2020 − September 2021 (Months 1−21)

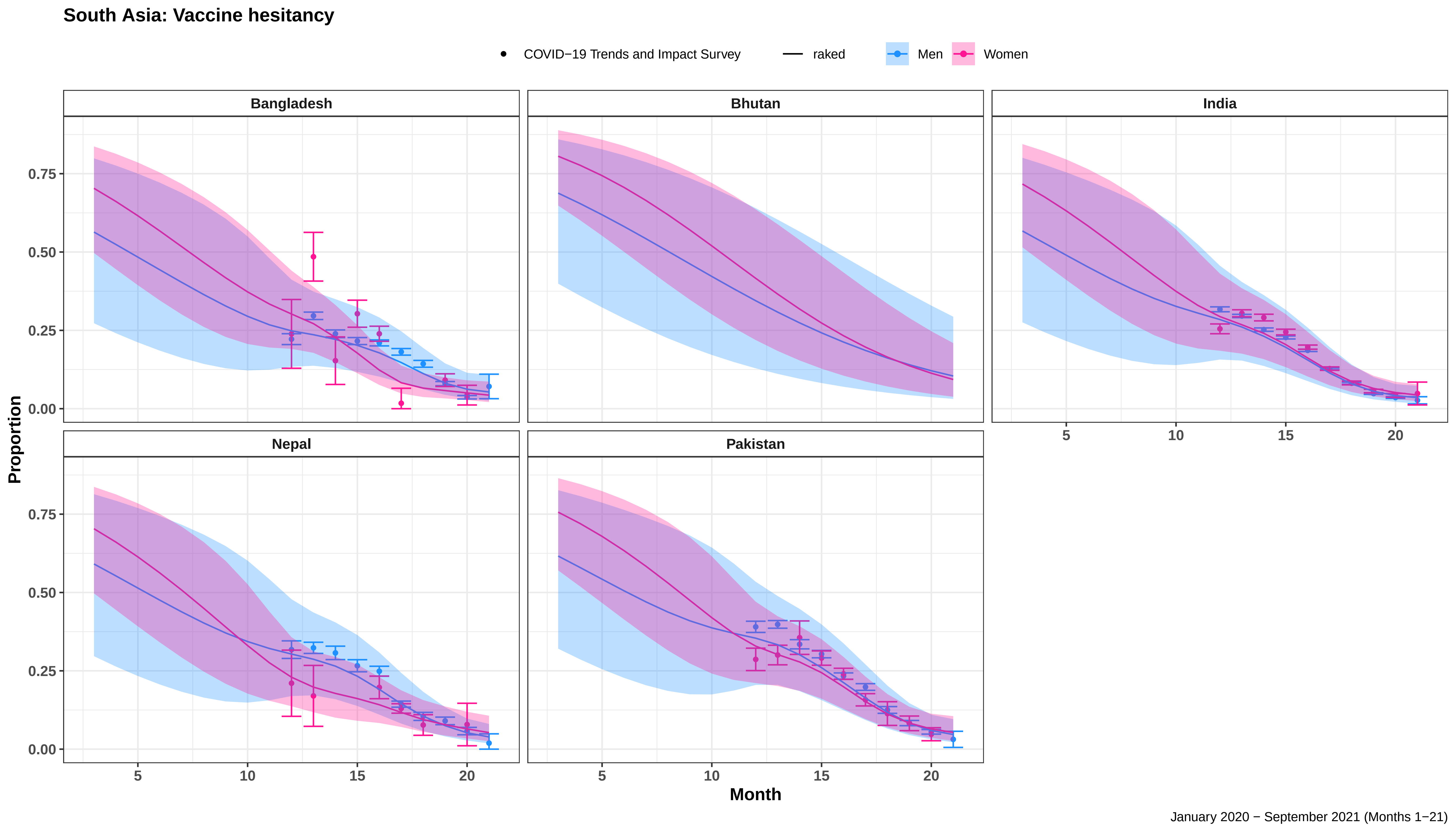
South Asia: Vaccine hesitancy
COVID−19 Trends and Impact Survey
raked
Men
Women
Bangladesh
Bhutan
India
Nepal
Pakistan
Proportion
0.00
0.25
0.50
0.75
5
10
15
20
Month
January 2020 − September 2021 (Months 1−21)

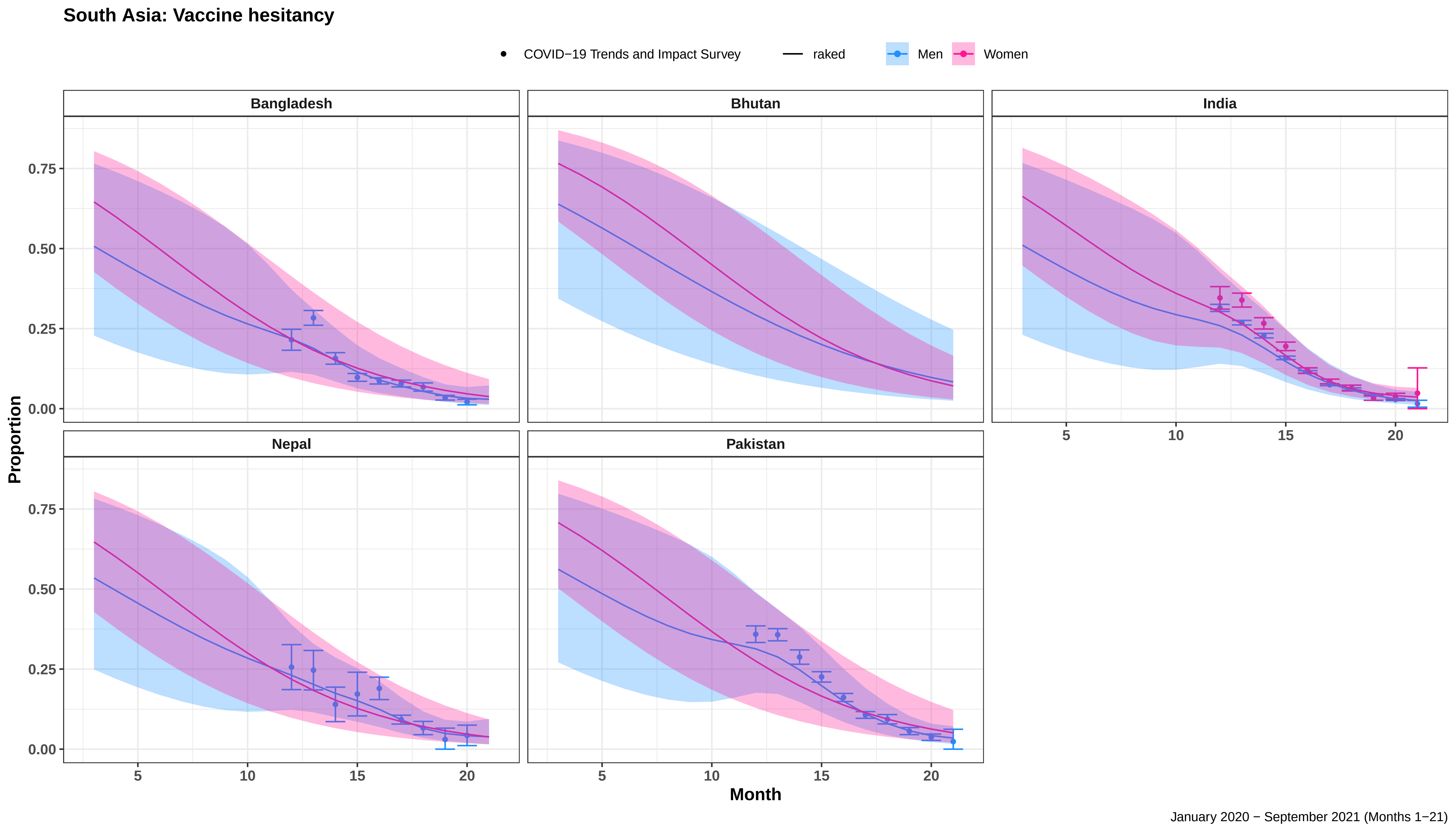

South Asia: Vaccine hesitancy
COVID−19 Trends and Impact Survey
raked
Men
Women
Bangladesh
Bhutan
India
Nepal
Pakistan
Proportion
0.00
0.25
0.50
0.75
5
10
15
20
Month
January 2020 − September 2021 (Months 1−21)

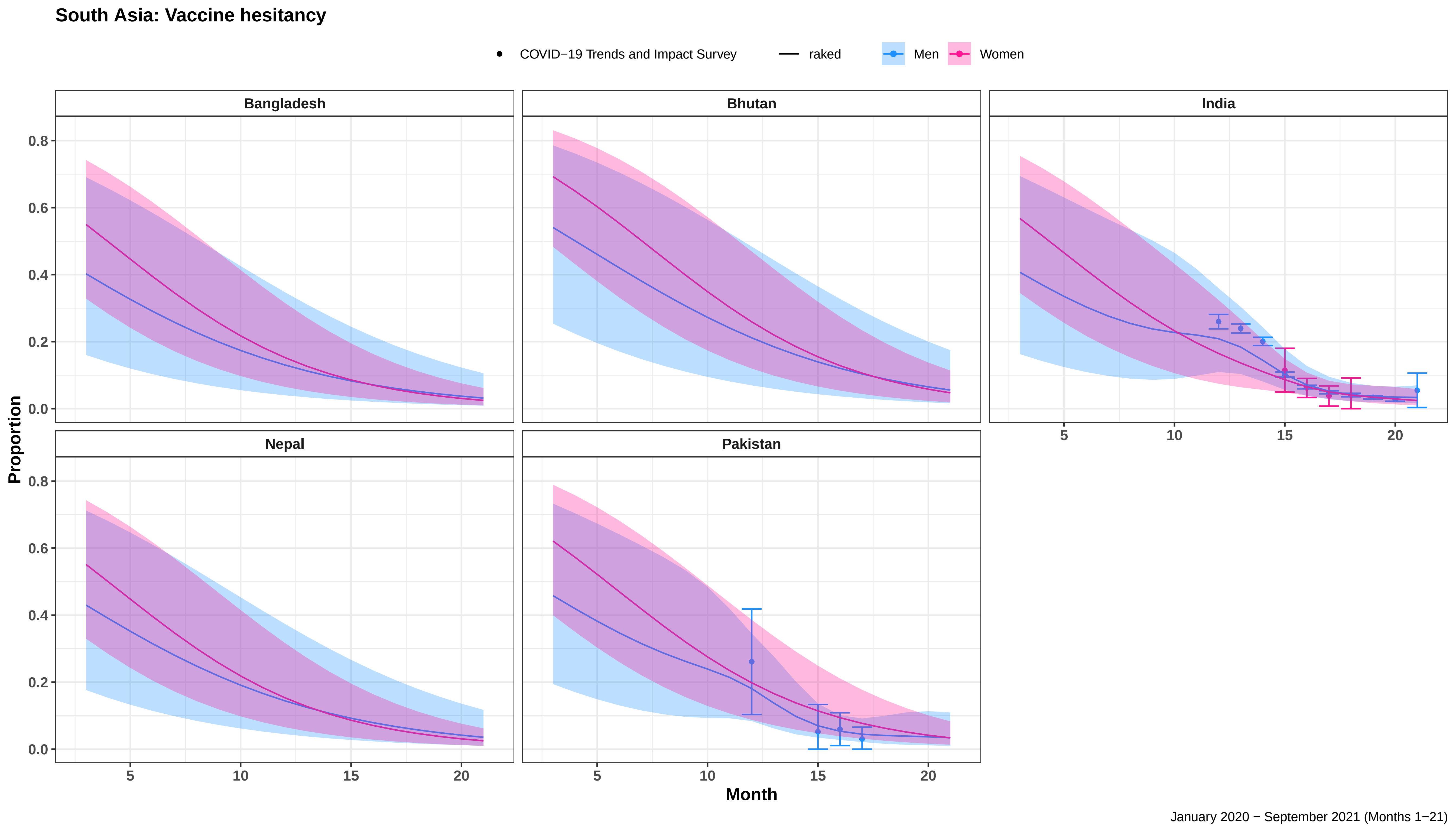

South Asia: Vaccine hesitancy
COVID−19 Trends and Impact Survey
raked
Men
Women
Bangladesh
Bhutan
India
Nepal
Pakistan
Proportion
0.0
0.2
0.4
0.6
0.8
5
10
15
20
Month
January 2020 − September 2021 (Months 1−21)

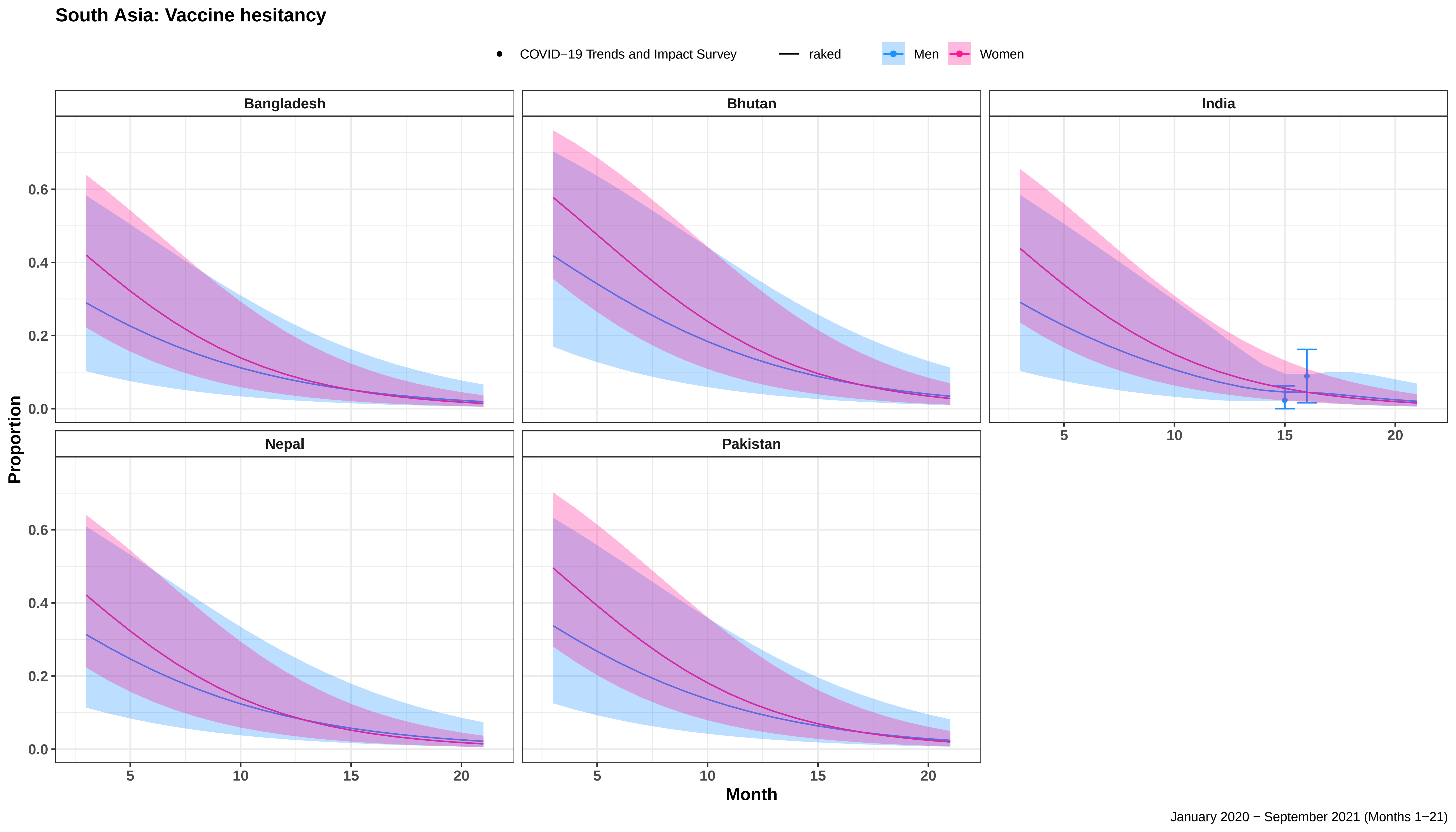
South Asia: Vaccine hesitancy
COVID−19 Trends and Impact Survey
raked
Men
Women
Bangladesh
Bhutan
India
Nepal
Pakistan
Proportion
0.0
0.2
0.4
0.6
Month
5
10
15
20
January 2020 − September 2021 (Months 1−21)

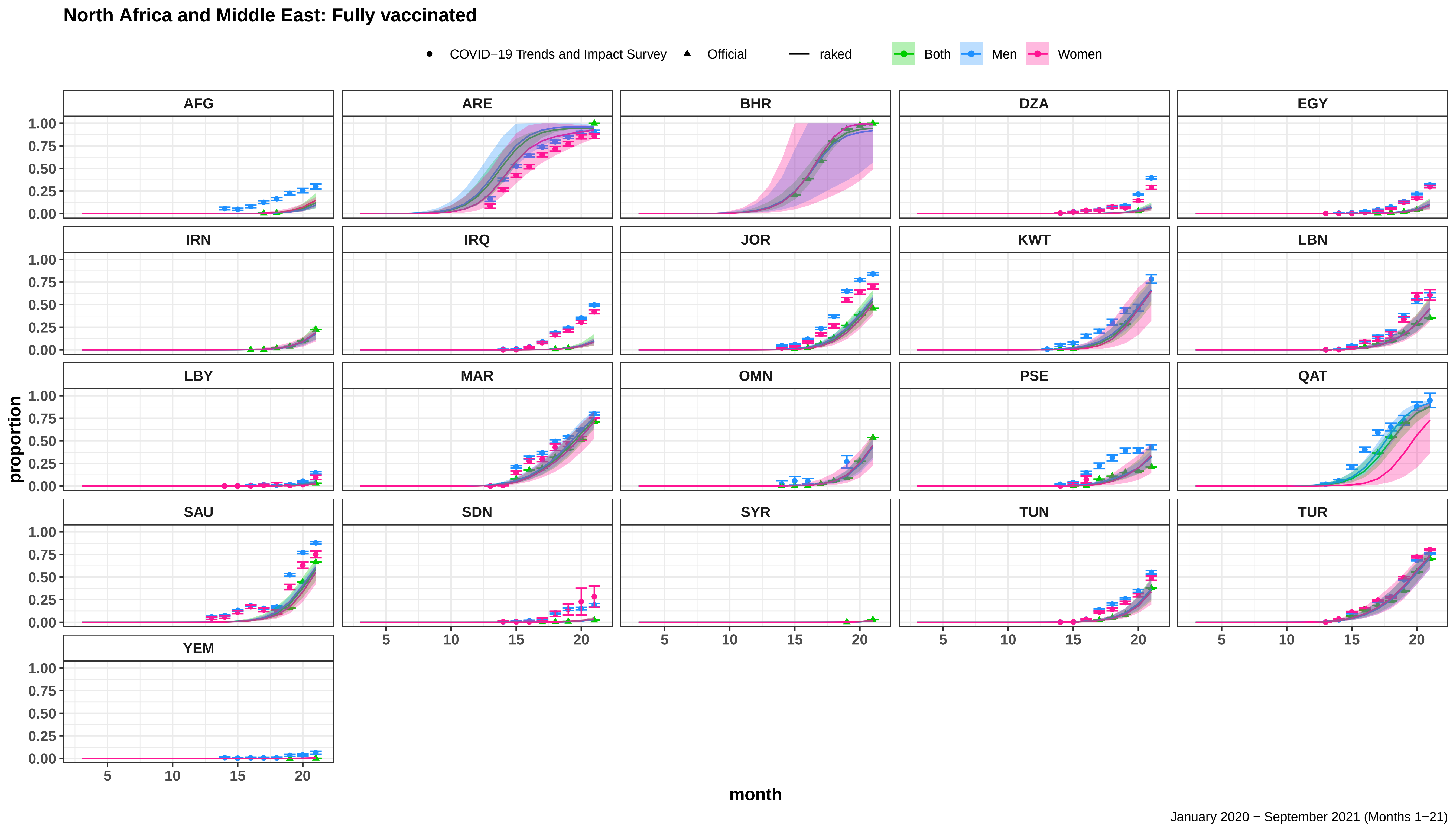
North Africa and Middle East: Fully vaccinated
COVID−19 Trends and Impact Survey
Official
raked
Both
Men
Women
AFG
ARE
BHR
DZA
EGY
IRN
IRQ
JOR
KWT
LBN
LBY
MAR
OMN
PSE
QAT
SAU
SDN
SYR
TUN
TUR
YEM
proportion
month
January 2020 − September 2021 (Months 1−21)

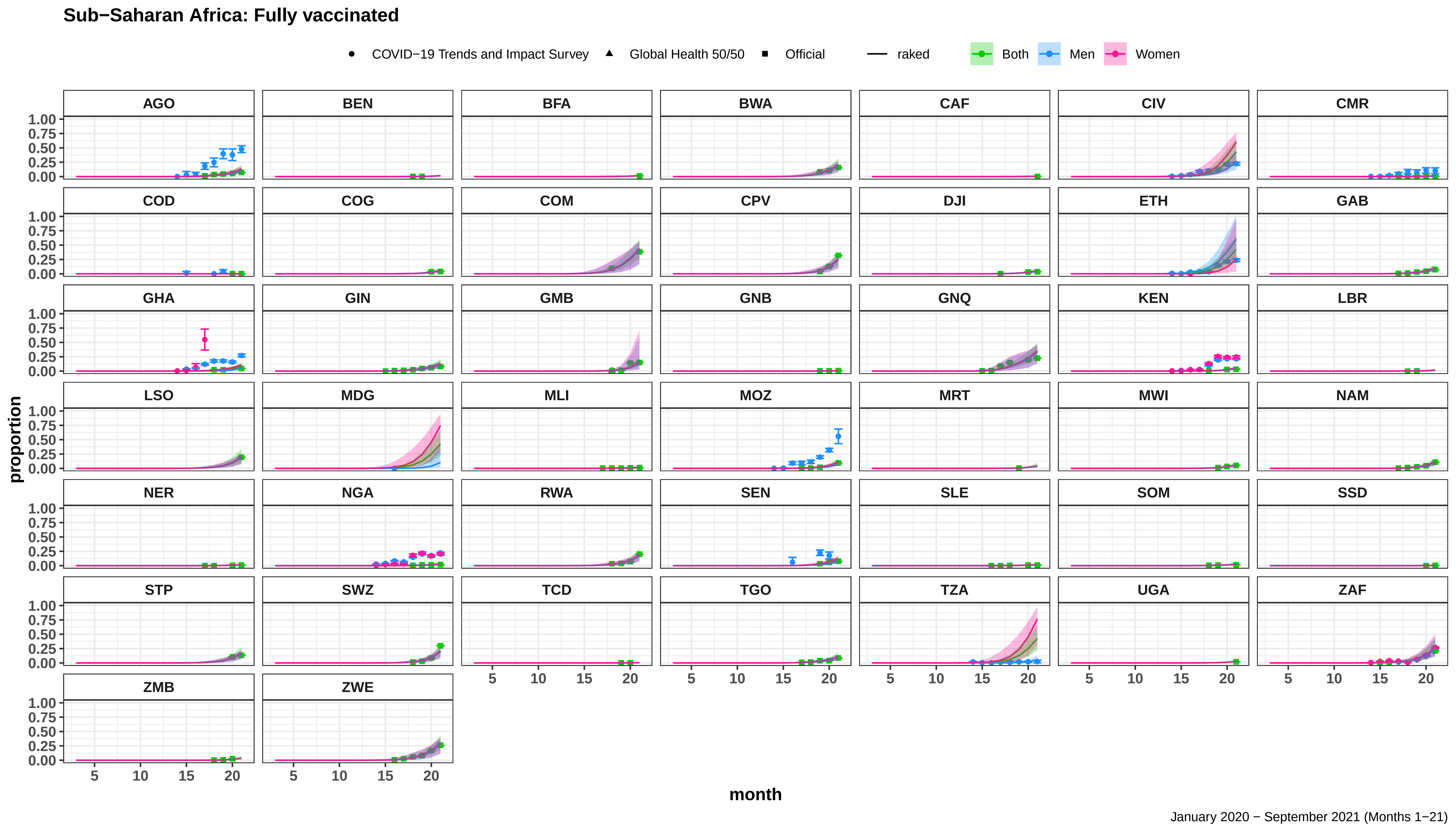
Sub−Saharan Africa: Fully vaccinated
COVID−19 Trends and Impact Survey
Global Health 50/50
Official
raked
Both
Men
Women
AGO
BEN
BFA
BWA
CAF
CIV
CMR
COD
COG
COM
CPV
DJI
ETH
GAB
GHA
GIN
GMB
GNB
GNQ
KEN
LBR
LSO
MDG
MLI
MOZ
MRT
MWI
NAM
NER
NGA
RWA
SEN
SLE
SOM
SSD
STP
SWZ
TCD
TGO
TZA
UGA
ZAF
ZMB
ZWE
proportion
0.00
0.25
0.50
0.75
1.00
month
5
10
15
20
January 2020 − September 2021 (Months 1−21)

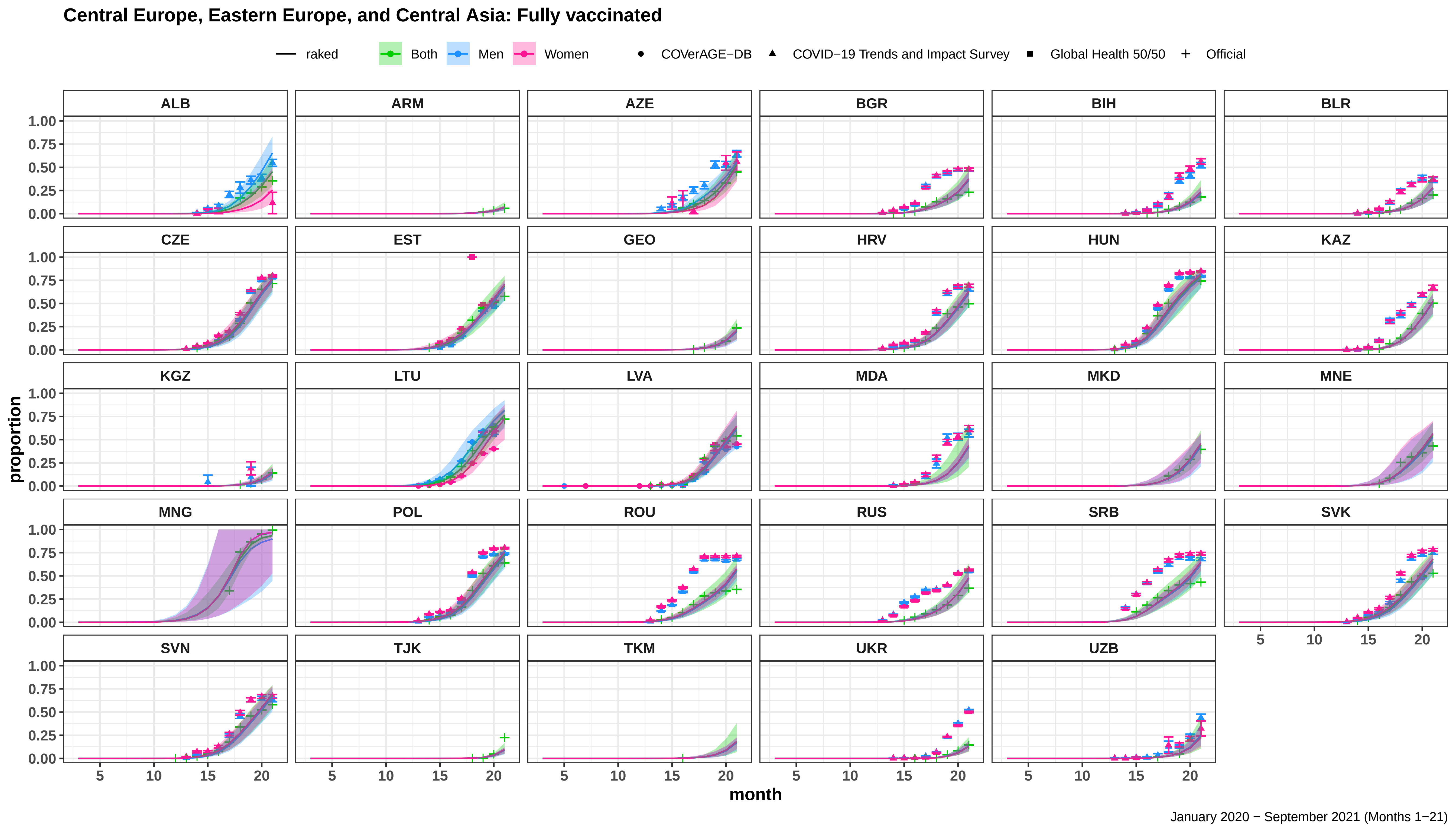

Central Europe, Eastern Europe, and Central Asia: Fully vaccinated
raked
Both
Men
Women
COVerAGE−DB
COVID−19 Trends and Impact Survey
Global Health 50/50
Official
ALB
ARM
AZE
BGR
BIH
BLR
CZE
EST
GEO
HRV
HUN
KAZ
KGZ
LTU
LVA
MDA
MKD
MNE
MNG
POL
ROU
RUS
SRB
SVK
SVN
TJK
TKM
UKR
UZB
proportion
month
January 2020 − September 2021 (Months 1−21)

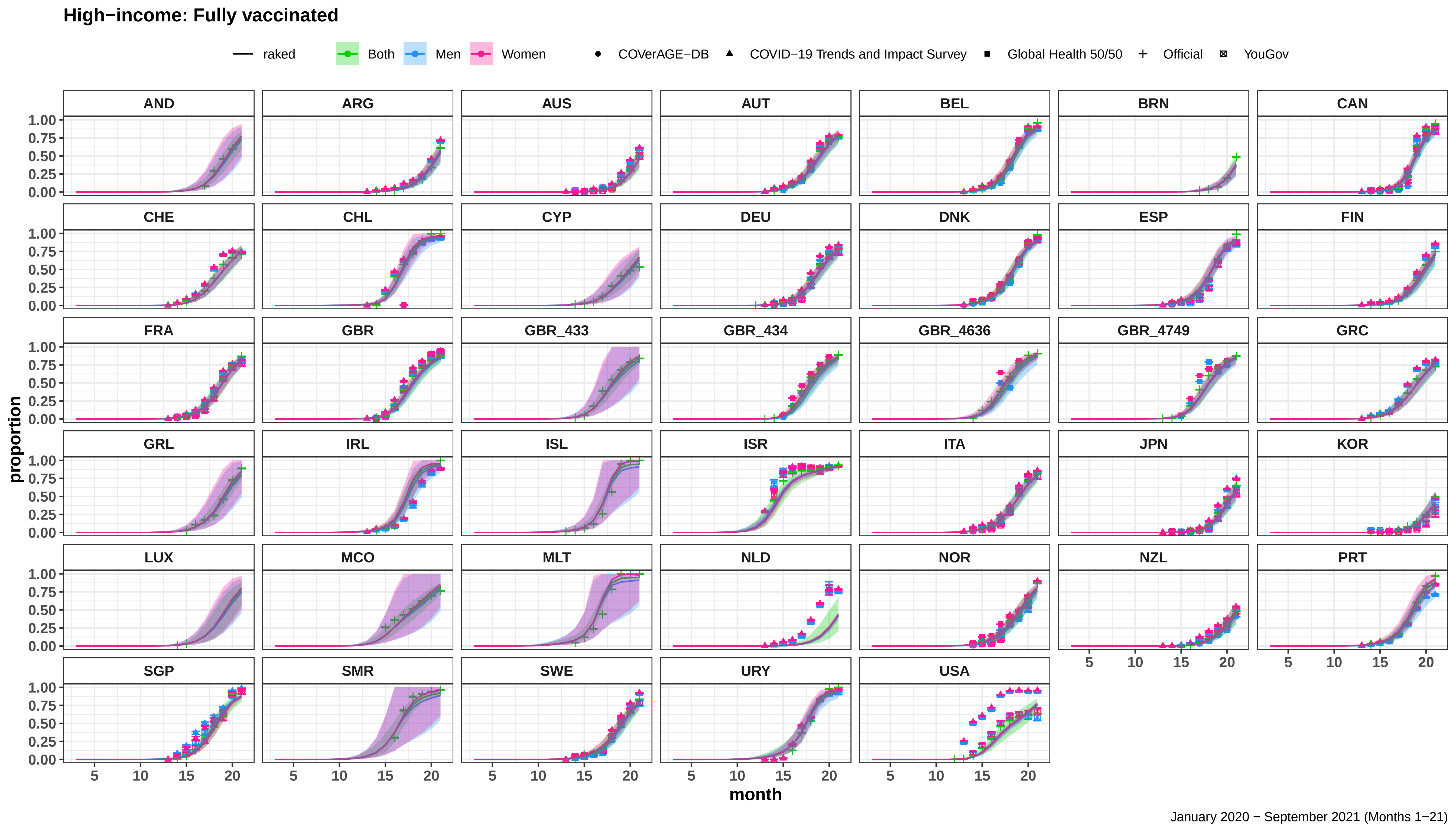
High−income: Fully vaccinated
raked
Both
Men
Women
COVerAGE−DB
COVID−19 Trends and Impact Survey
Global Health 50/50
Official
YouGov
AND
ARG
AUS
AUT
BEL
BRN
CAN
CHE
CHL
CYP
DEU
DNK
ESP
FIN
FRA
GBR
GBR_433
GBR_434
GBR_4636
GBR_4749
GRC
GRL
IRL
ISL
ISR
ITA
JPN
KOR
LUX
MCO
MLT
NLD
NOR
NZL
PRT
SGP
SMR
SWE
URY
USA
proportion
0.00
0.25
0.50
0.75
1.00
month
5
10
15
20
January 2020 − September 2021 (Months 1−21)

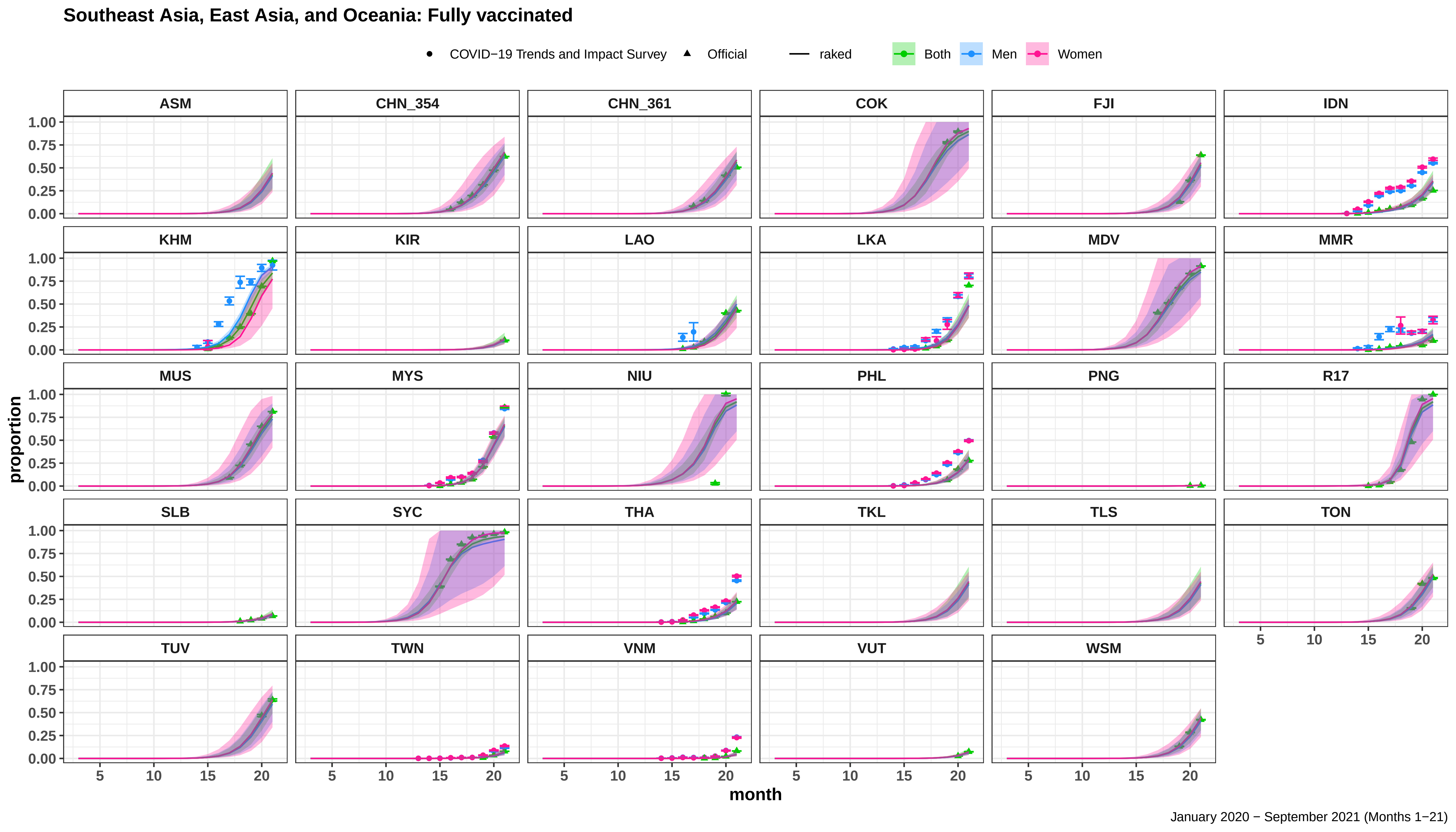
Southeast Asia, East Asia, and Oceania: Fully vaccinated
COVID−19 Trends and Impact Survey
Official
raked
Both
Men
Women
ASM
CHN_354
CHN_361
COK
FJI
IDN
KHM
KIR
LAO
LKA
MDV
MMR
MUS
MYS
NIU
PHL
PNG
R17
SLB
SYC
THA
TKL
TLS
TON
TUV
TWN
VNM
VUT
WSM
proportion
month
0.00
0.25
0.50
0.75
1.00
5
10
15
20
January 2020 − September 2021 (Months 1−21)

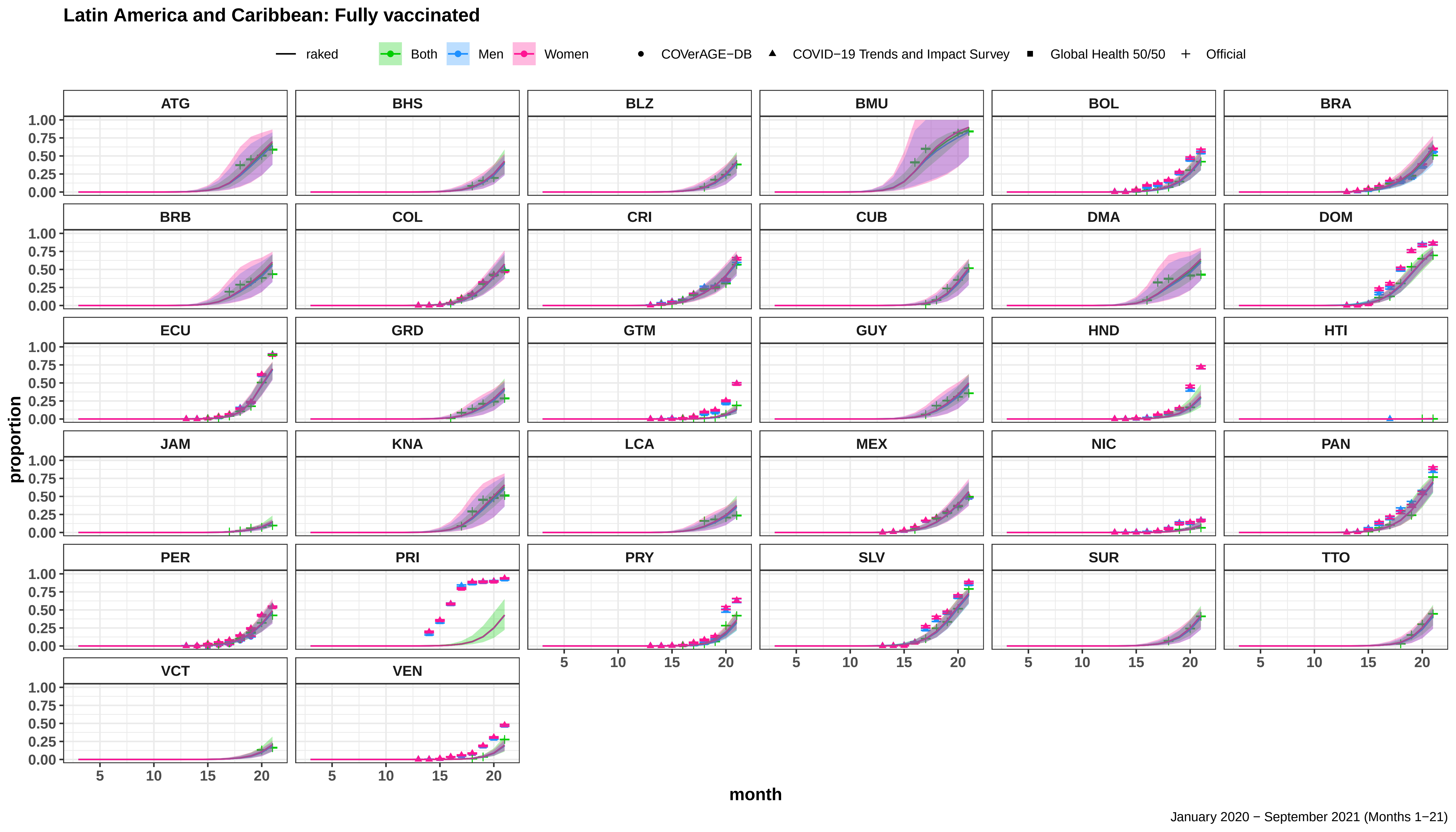

Latin America and Caribbean: Fully vaccinated
raked
Both
Men
Women
COVerAGE−DB
COVID−19 Trends and Impact Survey
Global Health 50/50
Official
ATG
BHS
BLZ
BMU
BOL
BRA
BRB
COL
CRI
CUB
DMA
DOM
ECU
GRD
GTM
GUY
HND
HTI
JAM
KNA
LCA
MEX
NIC
PAN
PER
PRI
PRY
SLV
SUR
TTO
VCT
VEN
proportion
0.00
0.25
0.50
0.75
1.00
month
5
10
15
20
January 2020 − September 2021 (Months 1−21)

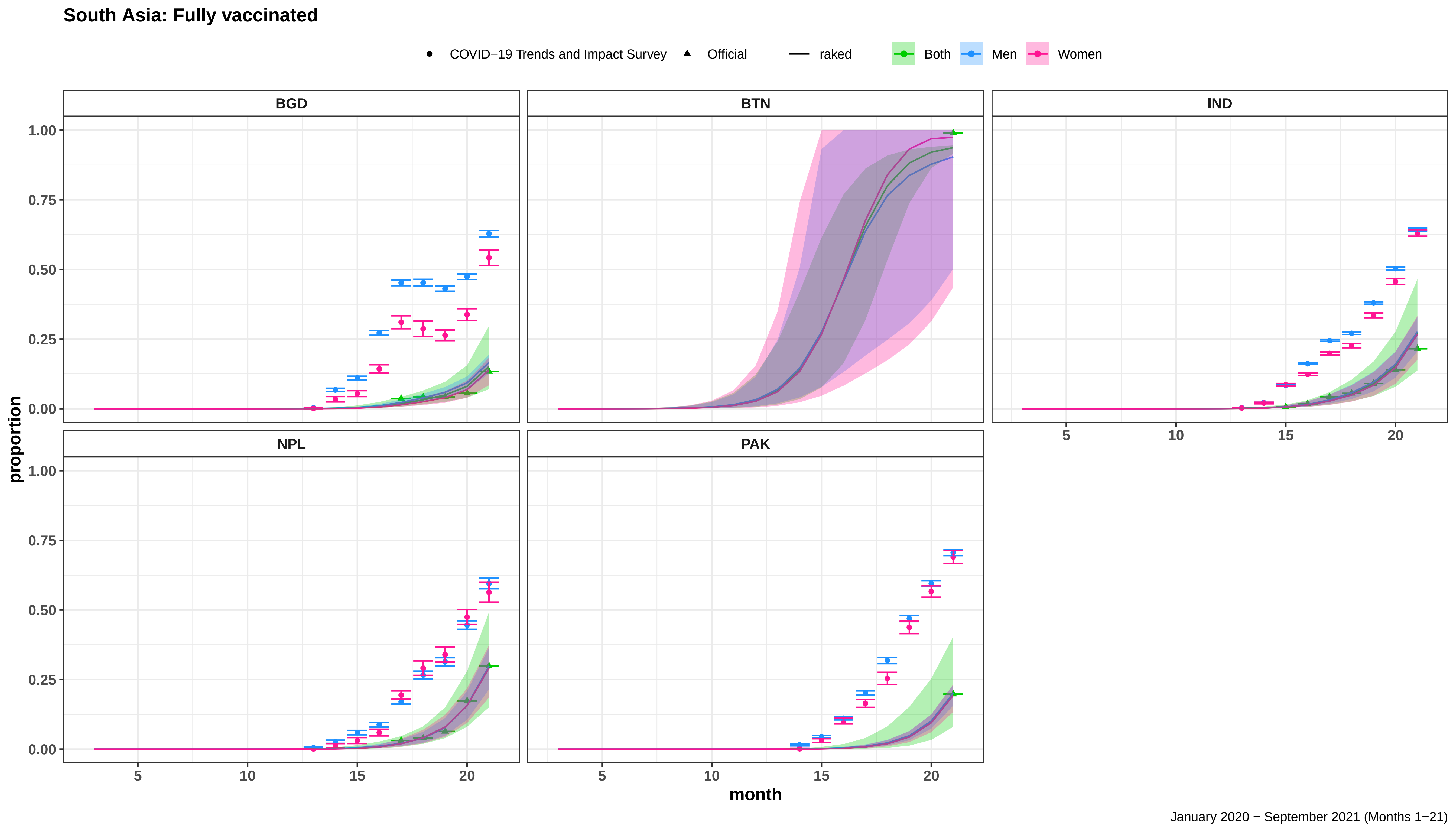

South Asia: Fully vaccinated
COVID−19 Trends and Impact Survey
Official
raked
Both
Men
Women
BGD
BTN
IND
NPL
PAK
proportion
1.00
0.75
0.50
0.25
0.00
5
10
15
20
month
January 2020 − September 2021 (Months 1−21)

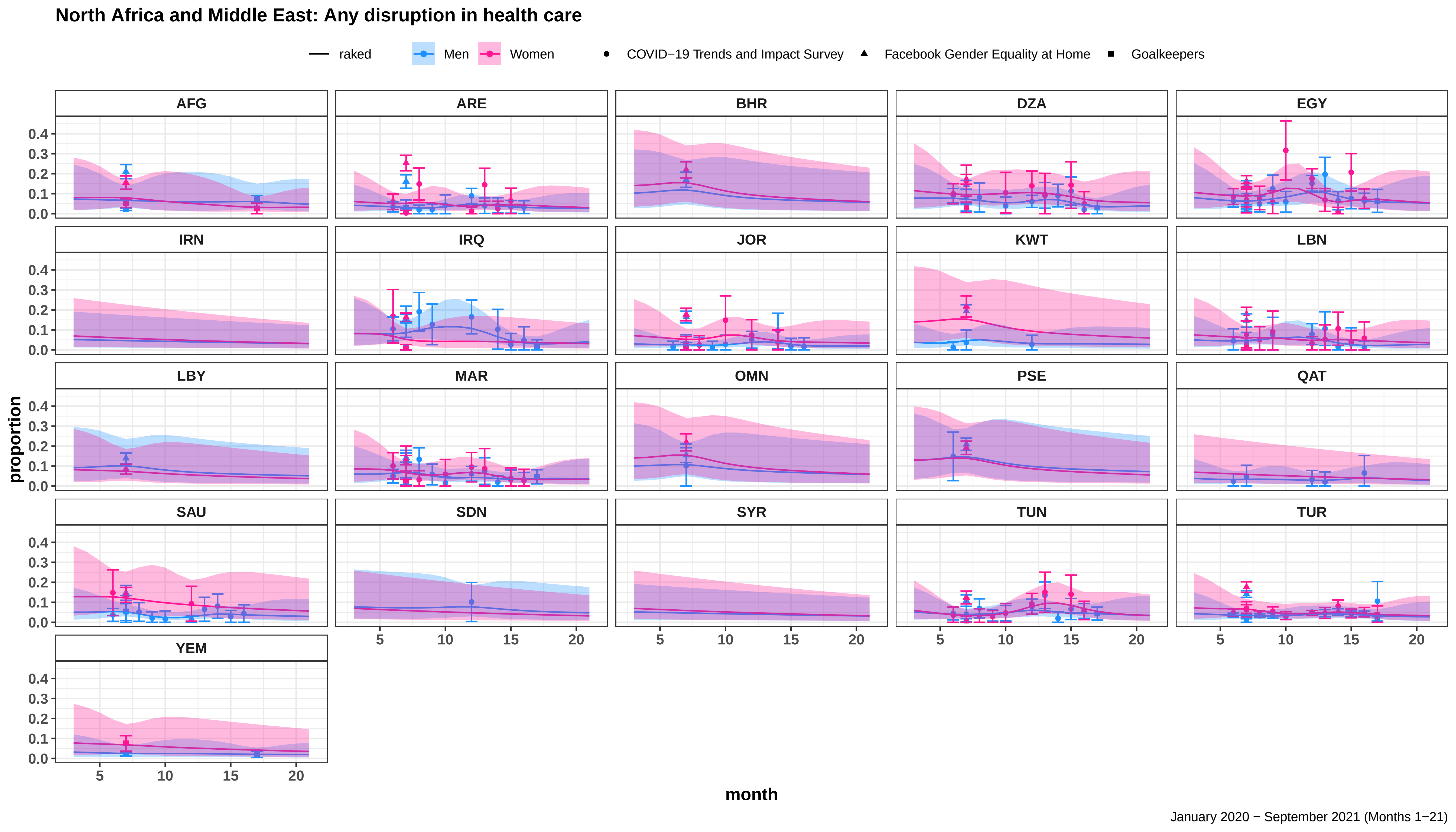
North Africa and Middle East: Any disruption in health care
raked
Men
Women
COVID−19 Trends and Impact Survey
Facebook Gender Equality at Home
Goalkeepers
AFG
ARE
BHR
DZA
EGY
IRN
IRQ
JOR
KWT
LBN
LBY
MAR
OMN
PSE
QAT
SAU
SDN
SYR
TUN
TUR
YEM
proportion
0.0
0.1
0.2
0.3
0.4
month
5
10
15
20
January 2020 − September 2021 (Months 1−21)

Sub−Saharan Africa: Any disruption in health care

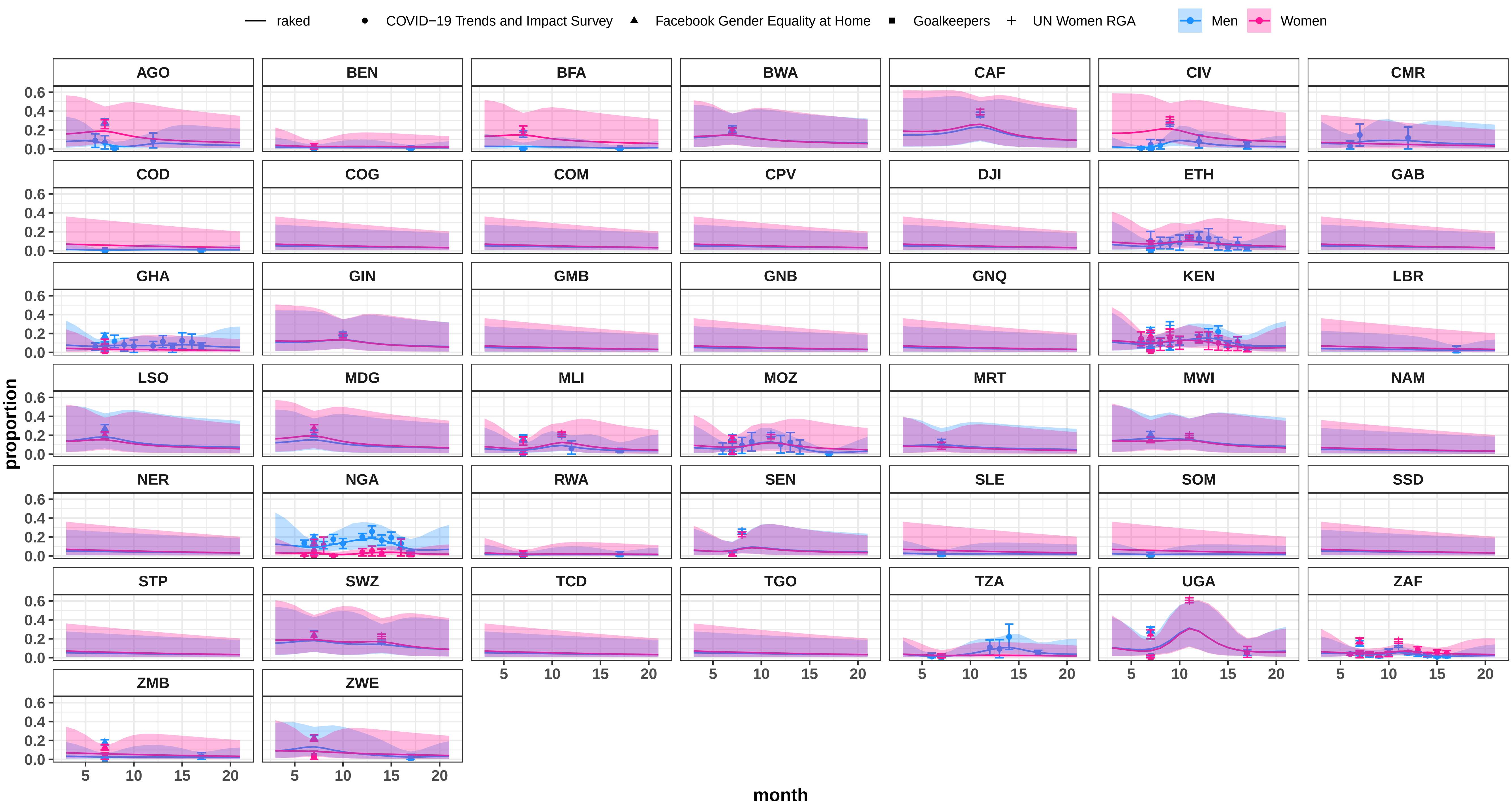

January 2020 − September 2021 (Months 1−21)

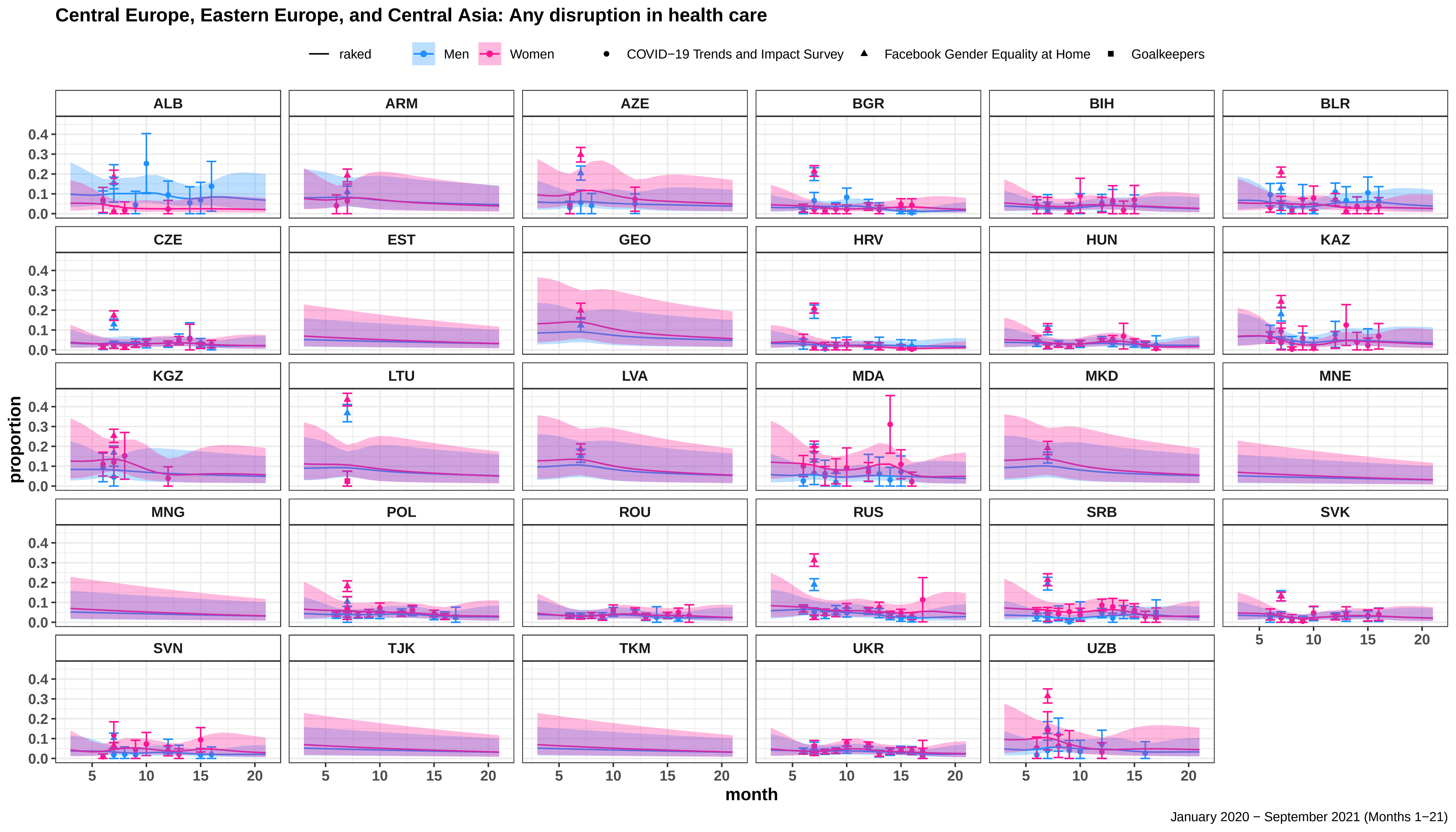
Central Europe, Eastern Europe, and Central Asia: Any disruption in health care
raked
Men
Women
COVID−19 Trends and Impact Survey
Facebook Gender Equality at Home
Goalkeepers
ALB
ARM
AZE
BGR
BIH
BLR
CZE
EST
GEO
HRV
HUN
KAZ
KGZ
LTU
LVA
MDA
MKD
MNE
MNG
POL
ROU
RUS
SRB
SVK
SVN
TJK
TKM
UKR
UZB
proportion
month
January 2020 − September 2021 (Months 1−21)

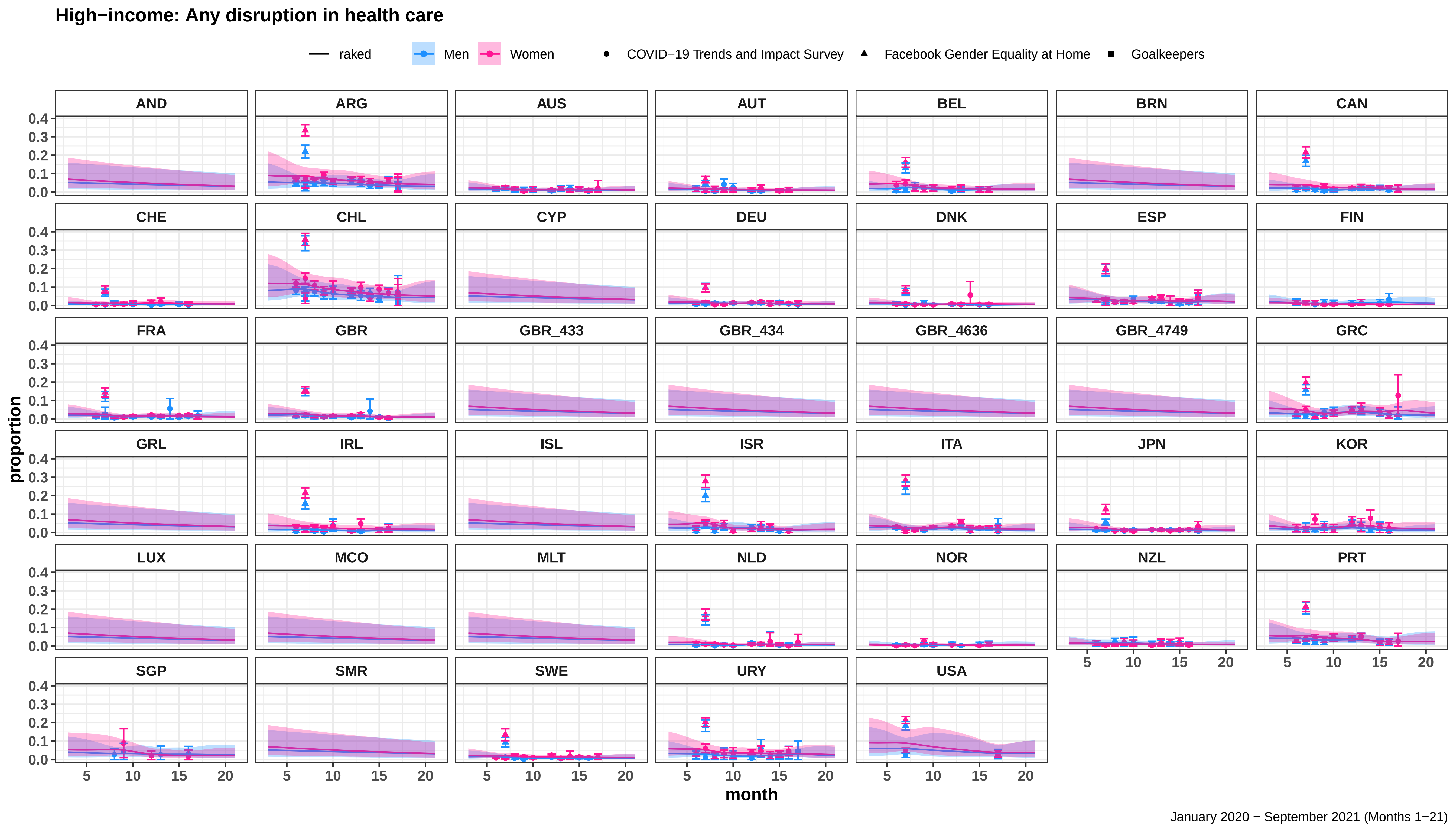

High−income: Any disruption in health care
raked
Men
Women
COVID−19 Trends and Impact Survey
Facebook Gender Equality at Home
Goalkeepers
AND
ARG
AUS
AUT
BEL
BRN
CAN
CHE
CHL
CYP
DEU
DNK
ESP
FIN
FRA
GBR
GBR_433
GBR_434
GBR_4636
GBR_4749
GRC
GRL
IRL
ISL
ISR
ITA
JPN
KOR
LUX
MCO
MLT
NLD
NOR
NZL
PRT
SGP
SMR
SWE
URY
USA
proportion
0.0
0.1
0.2
0.3
0.4
month
5
10
15
20
January 2020 − September 2021 (Months 1−21)

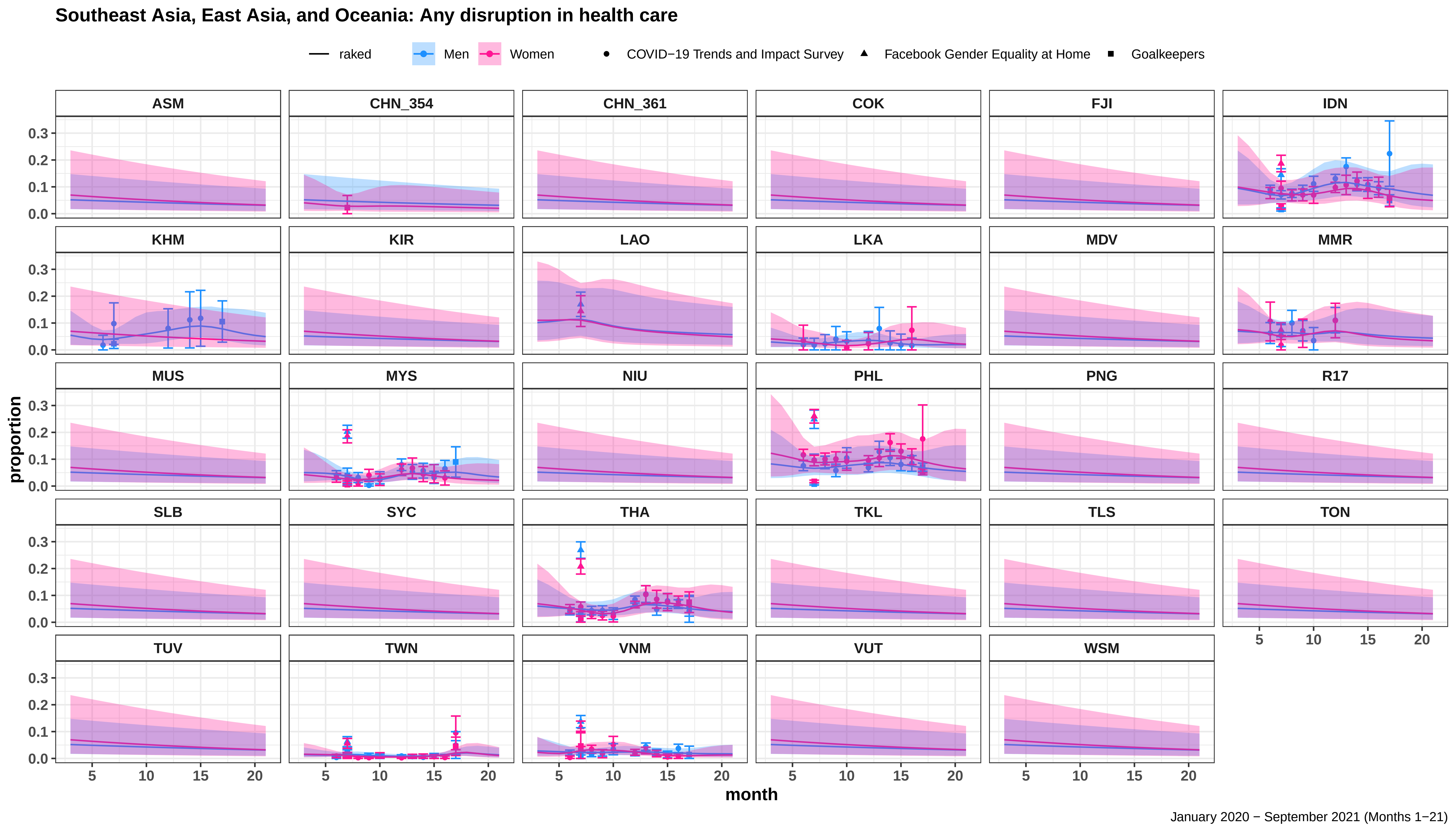
Southeast Asia, East Asia, and Oceania: Any disruption in health care
raked
Men
Women
COVID−19 Trends and Impact Survey
Facebook Gender Equality at Home
Goalkeepers
ASM
CHN_354
CHN_361
COK
FJI
IDN
KHM
KIR
LAO
LKA
MDV
MMR
MUS
MYS
NIU
PHL
PNG
R17
SLB
SYC
THA
TKL
TLS
TON
TUV
TWN
VNM
VUT
WSM
proportion
month
January 2020 − September 2021 (Months 1−21)

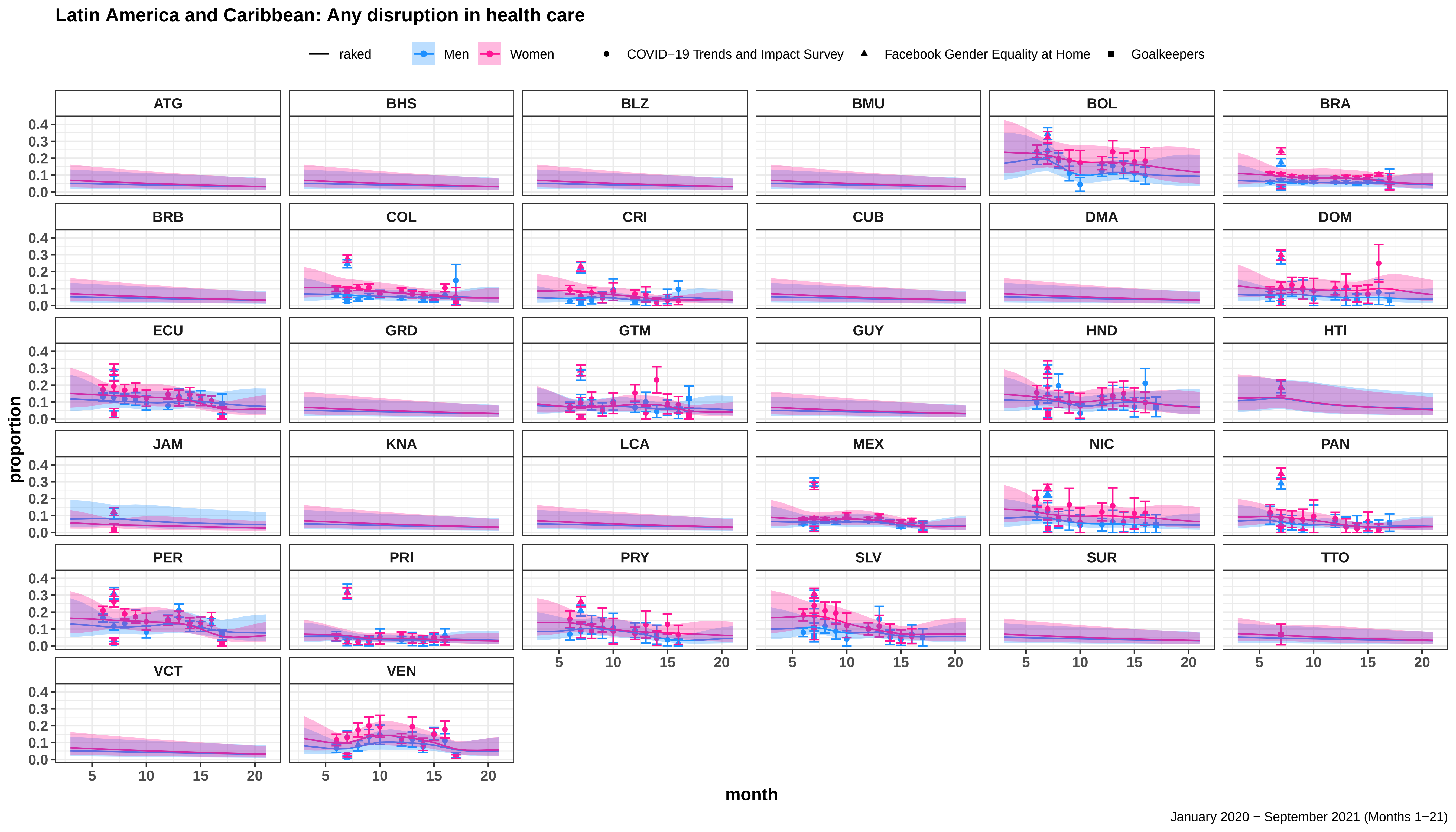

Latin America and Caribbean: Any disruption in health care
raked
Men
Women
COVID−19 Trends and Impact Survey
Facebook Gender Equality at Home
Goalkeepers
ATG
BHS
BLZ
BMU
BOL
BRA
BRB
COL
CRI
CUB
DMA
DOM
ECU
GRD
GTM
GUY
HND
HTI
JAM
KNA
LCA
MEX
NIC
PAN
PER
PRI
PRY
SLV
SUR
TTO
VCT
VEN
proportion
month
January 2020 − September 2021 (Months 1−21)

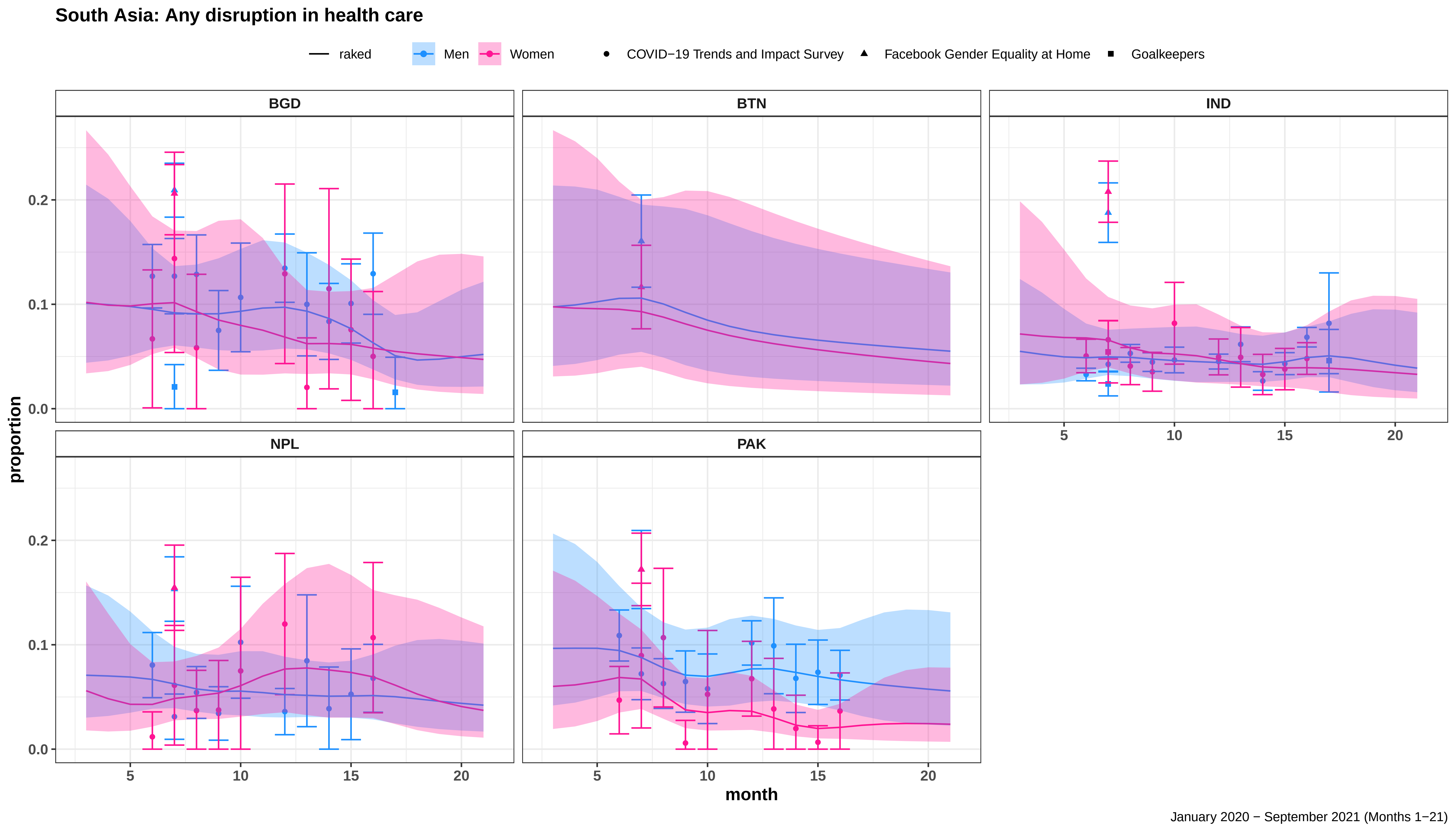
South Asia: Any disruption in health care
raked
Men
Women
COVID−19 Trends and Impact Survey
Facebook Gender Equality at Home
Goalkeepers
BGD
BTN
IND
NPL
PAK
proportion
0.0
0.1
0.2
5
10
15
20
month
January 2020 − September 2021 (Months 1−21)

North Africa and Middle East: Disruption in preventative care

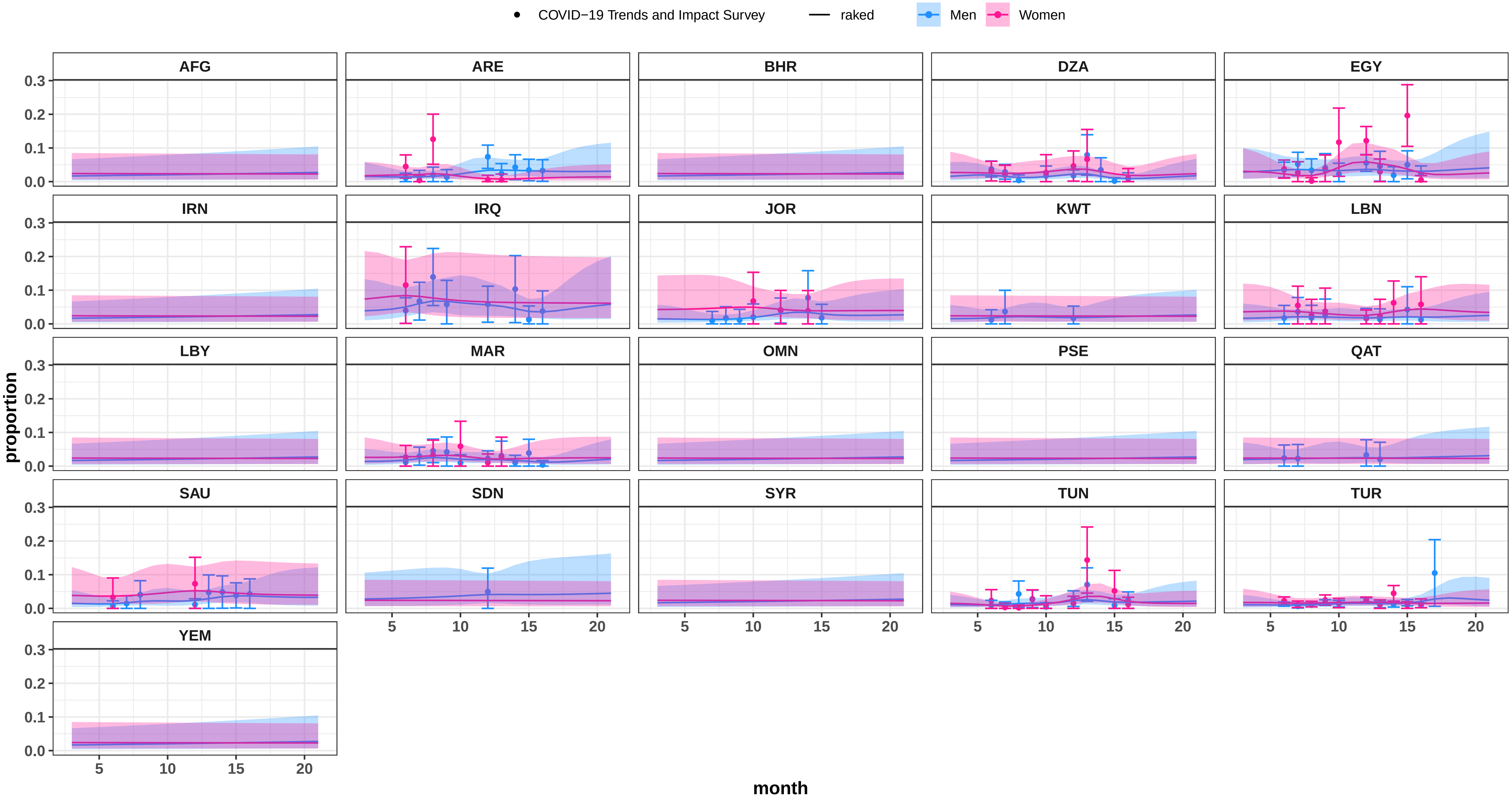

January 2020 − September 2021 (Months 1−21)

Sub−Saharan Africa: Disruption in preventative care

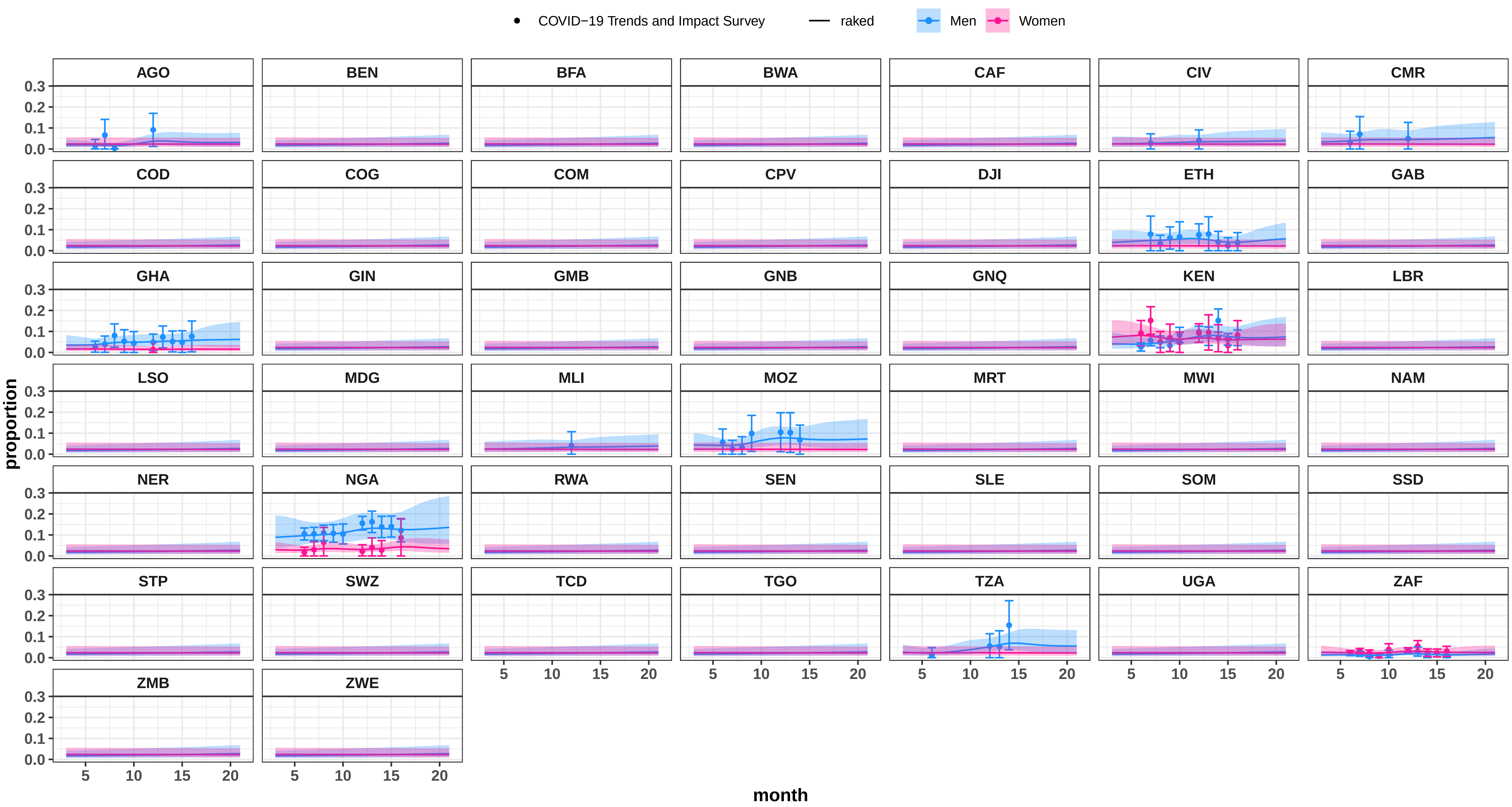

January 2020 − September 2021 (Months 1−21)

Central Europe, Eastern Europe, and Central Asia: Disruption in preventative care

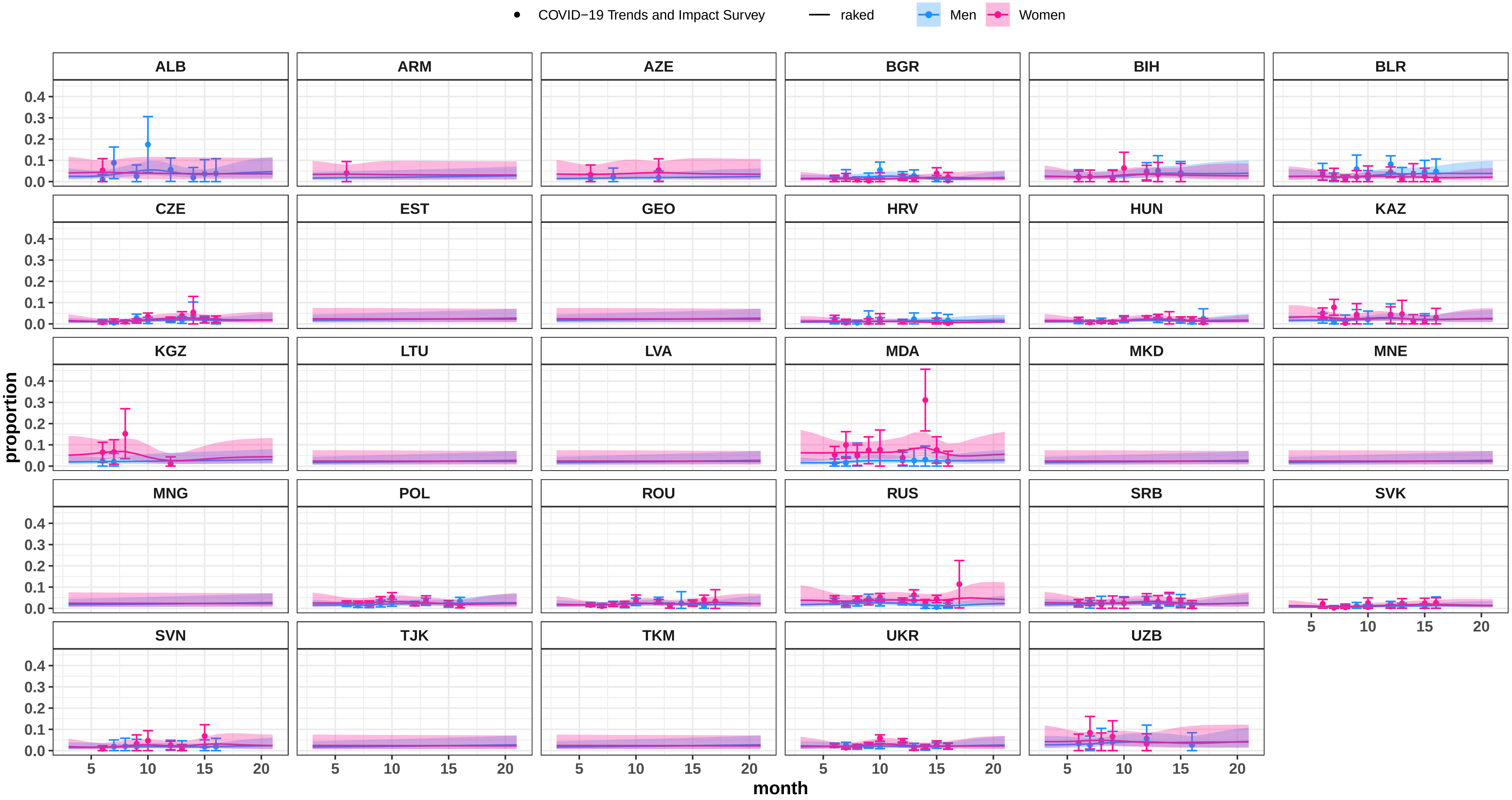

January 2020 − September 2021 (Months 1−21)

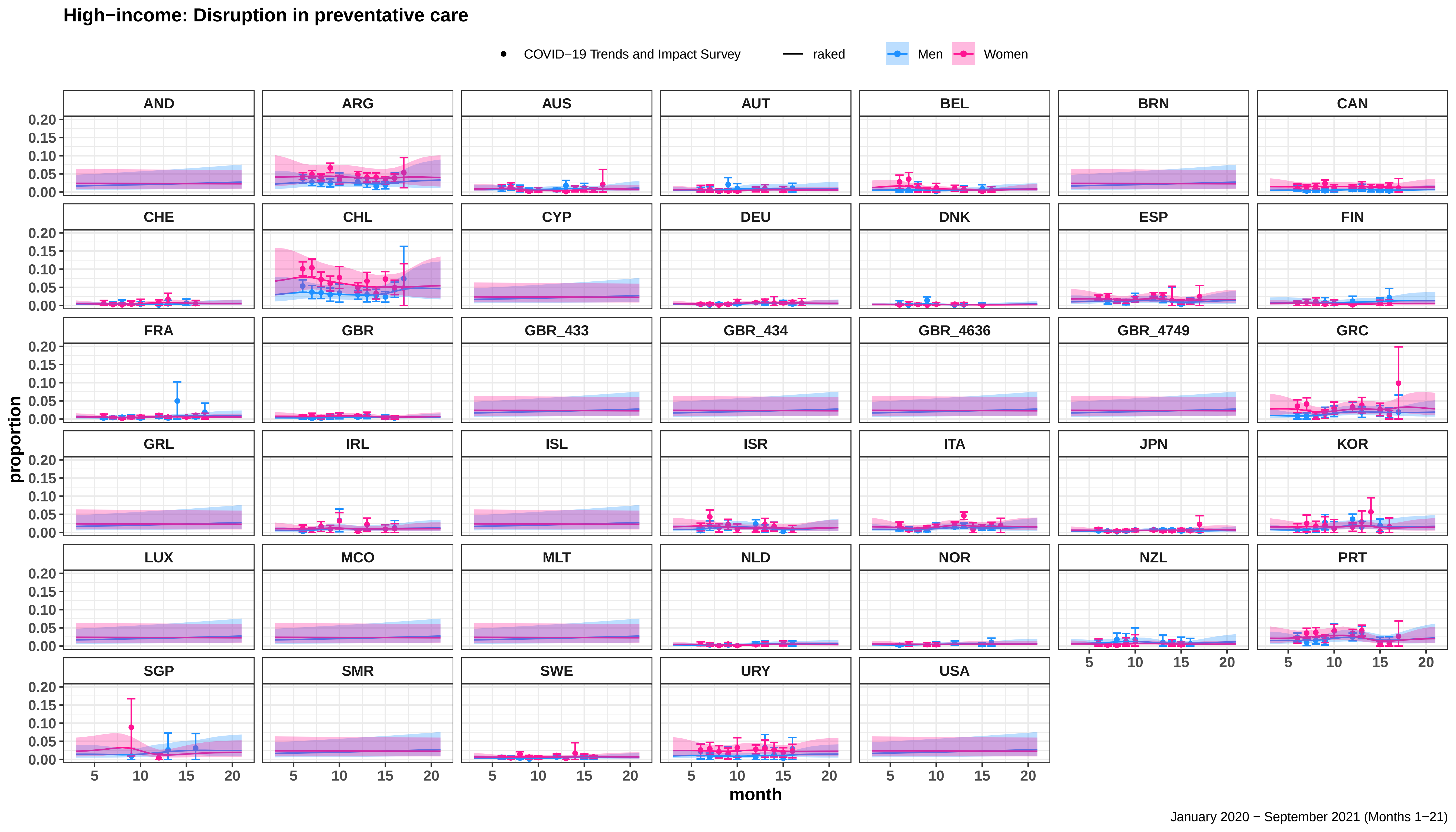

High−income: Disruption in preventative care
COVID−19 Trends and Impact Survey
raked
Men
Women
AND
ARG
AUS
AUT
BEL
BRN
CAN
CHE
CHL
CYP
DEU
DNK
ESP
FIN
FRA
GBR
GBR_433
GBR_434
GBR_4636
GBR_4749
GRC
GRL
IRL
ISL
ISR
ITA
JPN
KOR
LUX
MCO
MLT
NLD
NOR
NZL
PRT
SGP
SMR
SWE
URY
USA
proportion
0.00
0.05
0.10
0.15
0.20
5
10
15
20
month
January 2020 − September 2021 (Months 1−21)

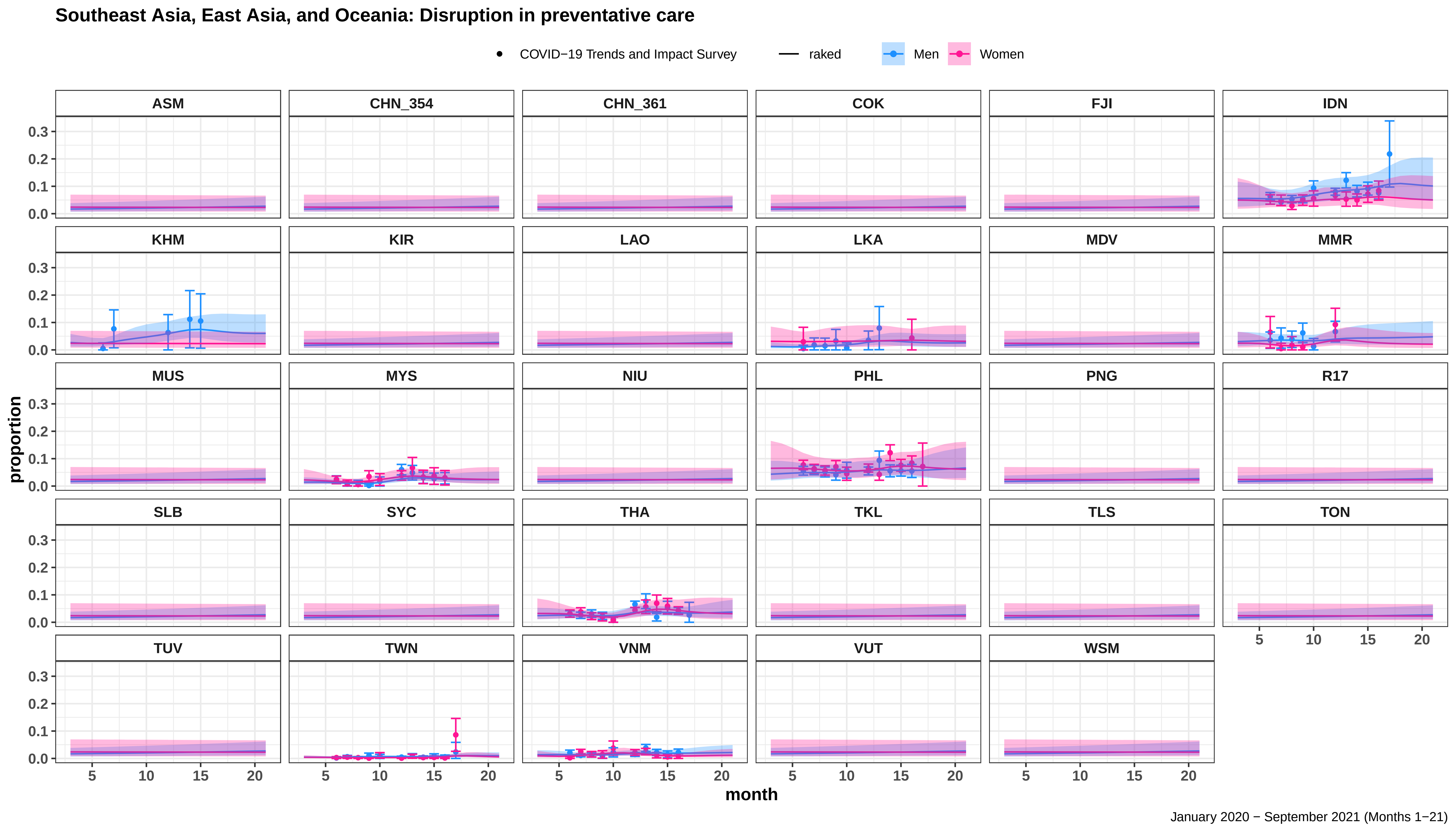

Southeast Asia, East Asia, and Oceania: Disruption in preventative care
COVID−19 Trends and Impact Survey
raked
Men
Women
ASM
CHN_354
CHN_361
COK
FJI
IDN
KHM
KIR
LAO
LKA
MDV
MMR
MUS
MYS
NIU
PHL
PNG
R17
SLB
SYC
THA
TKL
TLS
TON
TUV
TWN
VNM
VUT
WSM
proportion
0.0
0.1
0.2
0.3
month
5
10
15
20
January 2020 − September 2021 (Months 1−21)

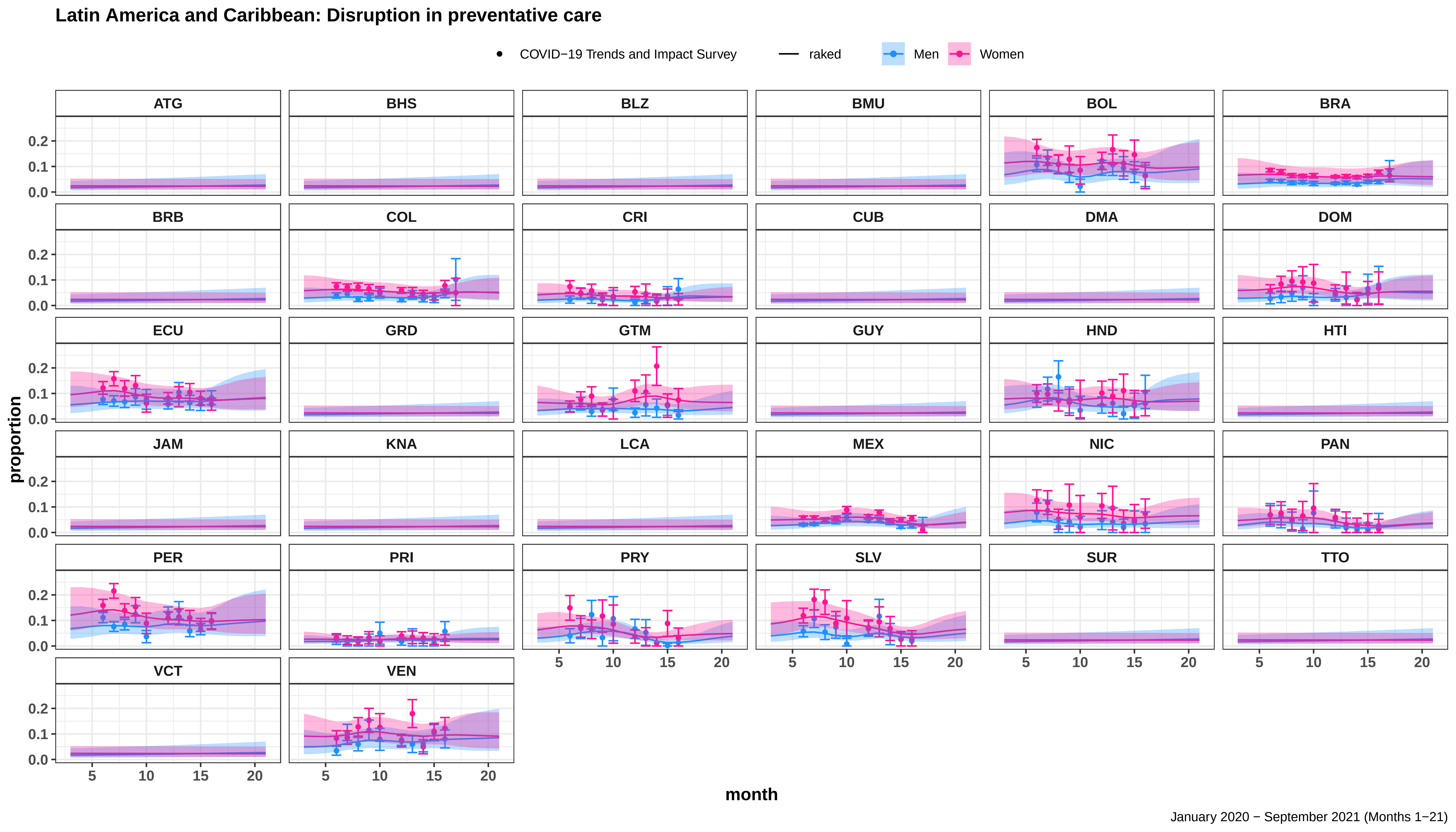

Latin America and Caribbean: Disruption in preventative care
COVID−19 Trends and Impact Survey
raked
Men
Women
ATG
BHS
BLZ
BMU
BOL
BRA
BRB
COL
CRI
CUB
DMA
DOM
ECU
GRD
GTM
GUY
HND
HTI
JAM
KNA
LCA
MEX
NIC
PAN
PER
PRI
PRY
SLV
SUR
TTO
VCT
VEN
proportion
0.0
0.1
0.2
month
5
10
15
20
January 2020 − September 2021 (Months 1−21)

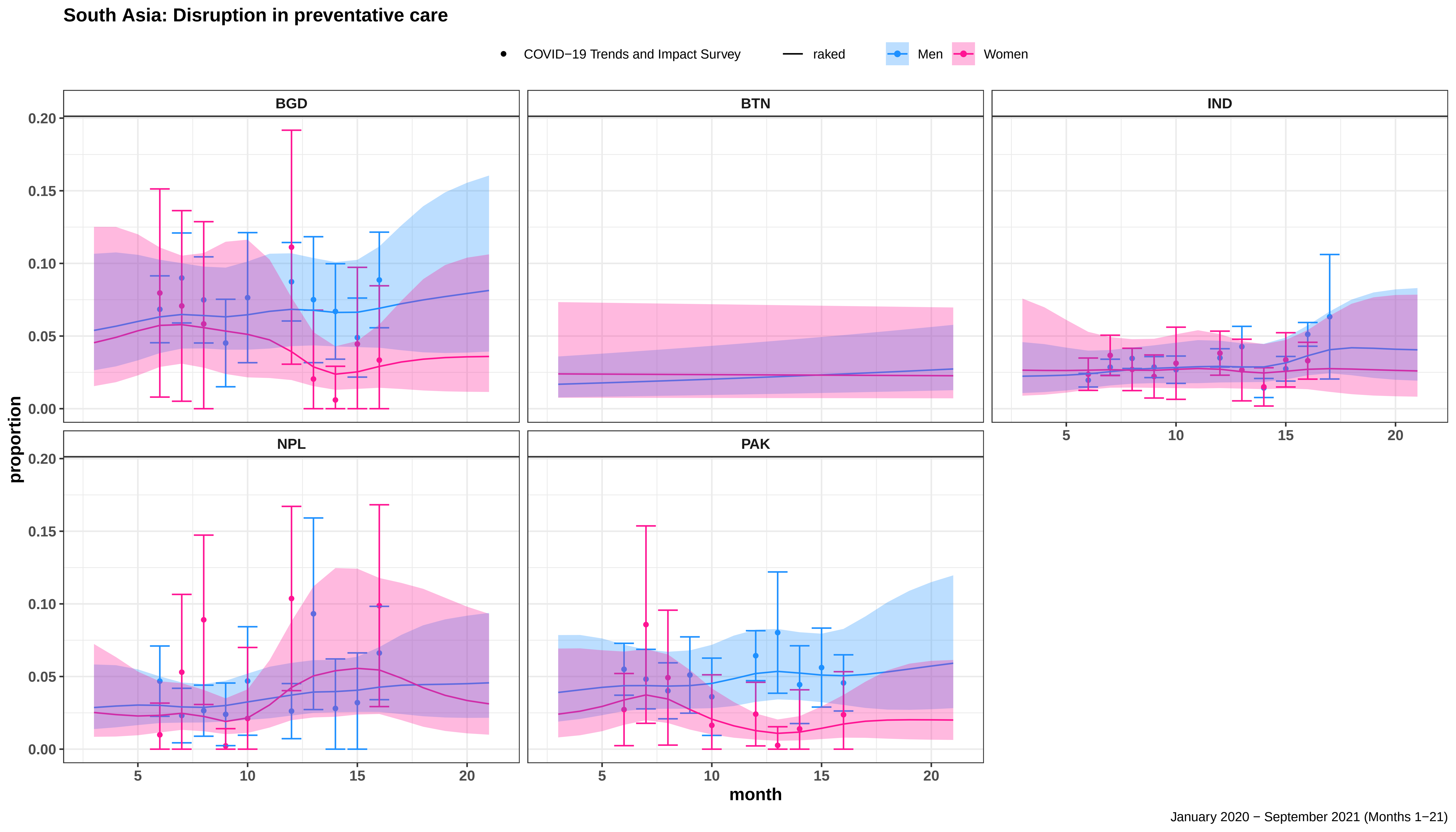

South Asia: Disruption in preventative care
COVID−19 Trends and Impact Survey
raked
Men
Women
BGD
BTN
IND
NPL
PAK
proportion
0.00
0.05
0.10
0.15
0.20
month
5
10
15
20
January 2020 − September 2021 (Months 1−21)

North Africa and Middle East: Disruption in medication access

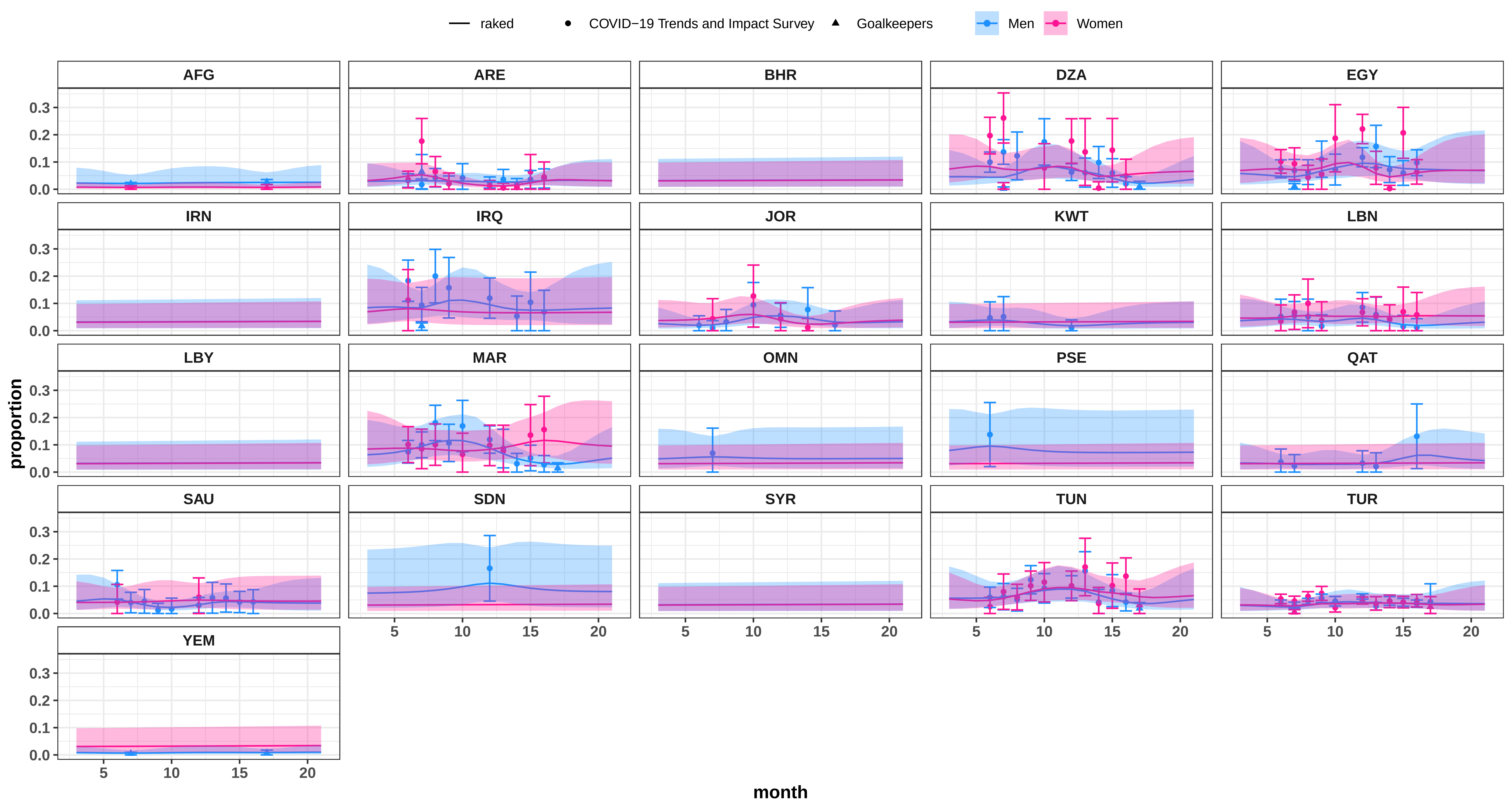

January 2020 − September 2021 (Months 1−21)

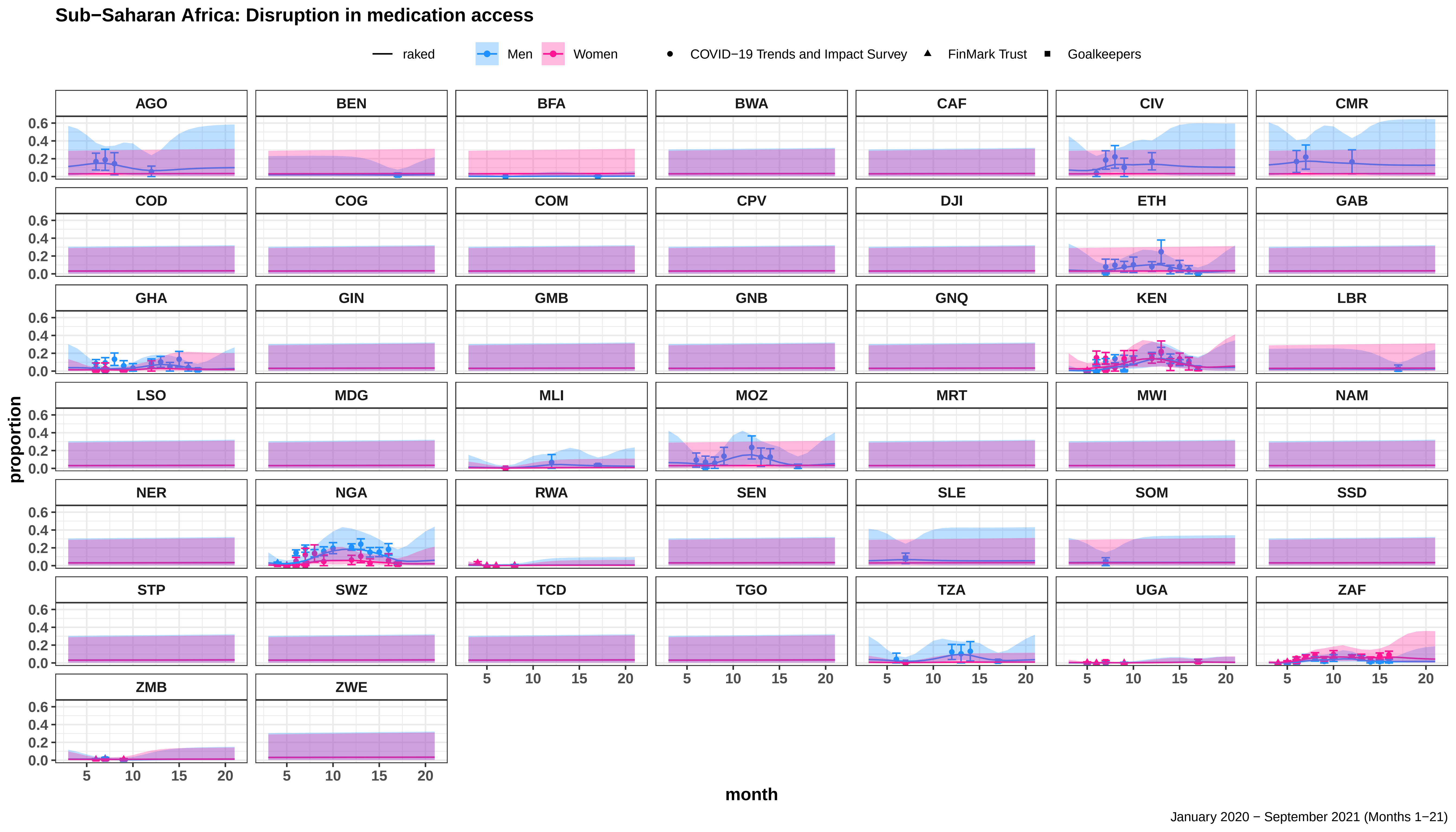

Sub−Saharan Africa: Disruption in medication access
raked
Men
Women
COVID−19 Trends and Impact Survey
FinMark Trust
Goalkeepers
AGO
BEN
BFA
BWA
CAF
CIV
CMR
COD
COG
COM
CPV
DJI
ETH
GAB
GHA
GIN
GMB
GNB
GNQ
KEN
LBR
LSO
MDG
MLI
MOZ
MRT
MWI
NAM
NER
NGA
RWA
SEN
SLE
SOM
SSD
STP
SWZ
TCD
TGO
TZA
UGA
ZAF
ZMB
ZWE
proportion
0.0
0.2
0.4
0.6
month
5
10
15
20
January 2020 − September 2021 (Months 1−21)

Central Europe, Eastern Europe, and Central Asia: Disruption in medication access

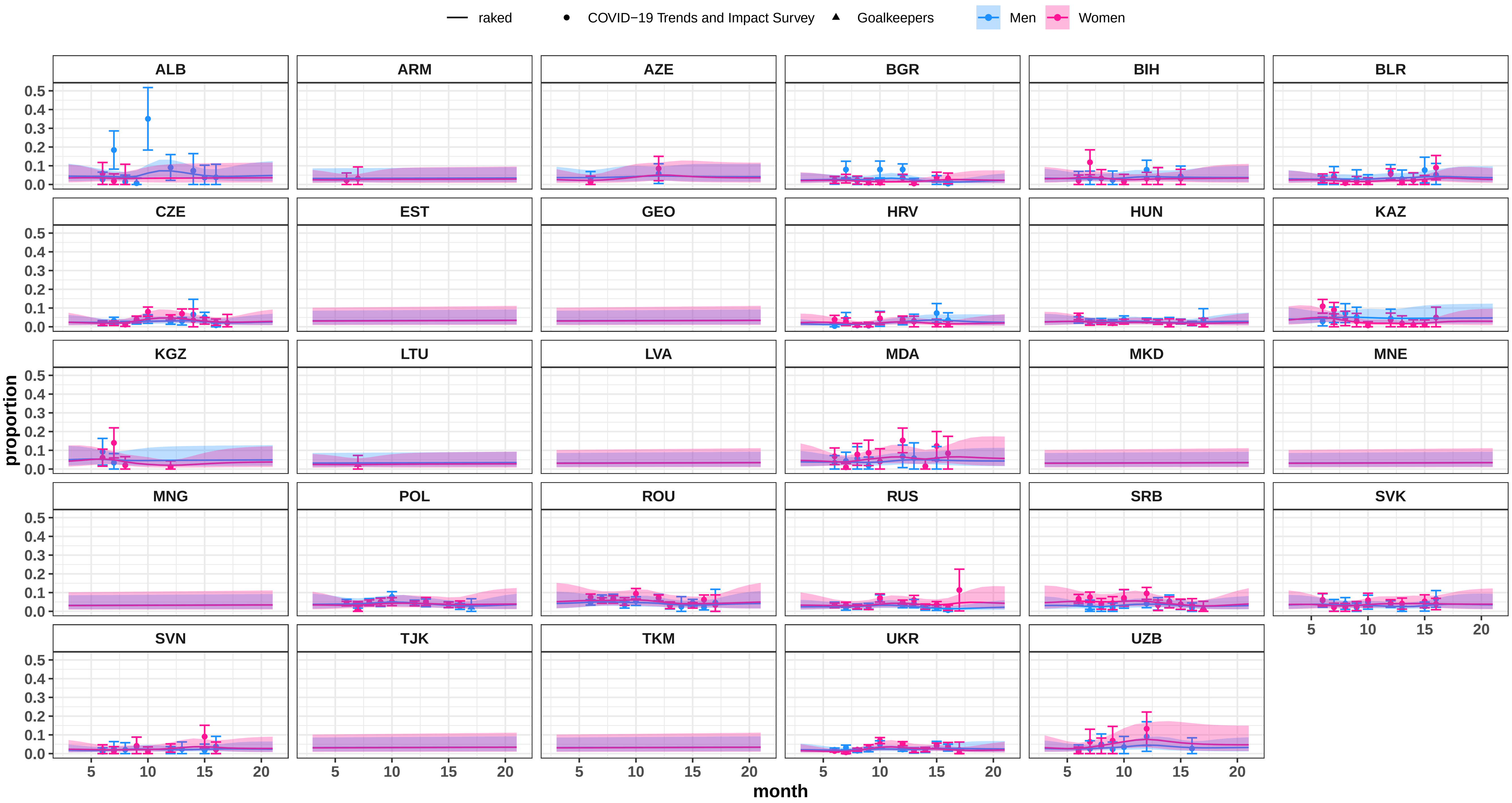

January 2020 − September 2021 (Months 1−21)

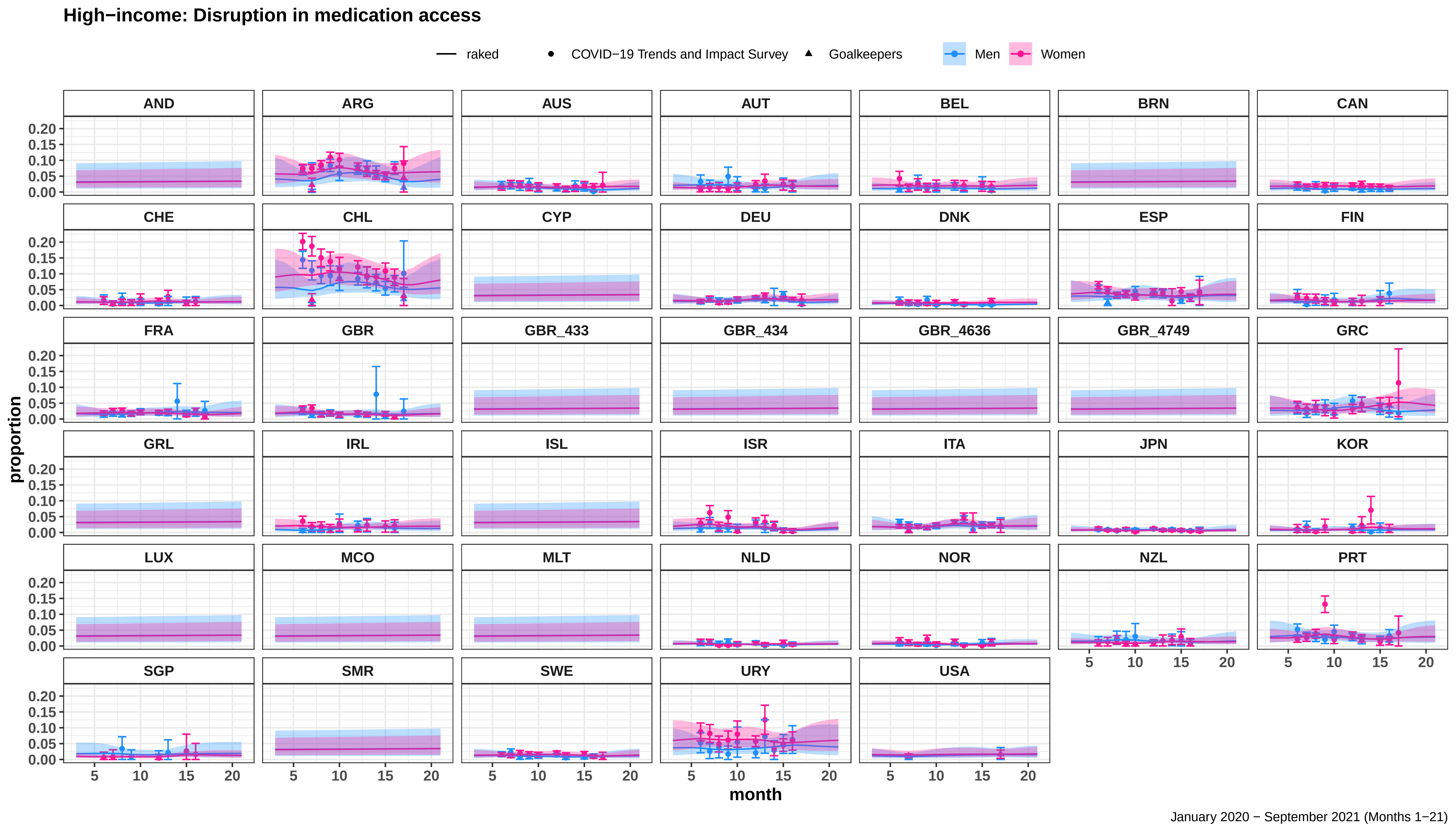
High−income: Disruption in medication access
raked
COVID−19 Trends and Impact Survey
Goalkeepers
Men
Women
AND
ARG
AUS
AUT
BEL
BRN
CAN
CHE
CHL
CYP
DEU
DNK
ESP
FIN
FRA
GBR
GBR_433
GBR_434
GBR_4636
GBR_4749
GRC
GRL
IRL
ISL
ISR
ITA
JPN
KOR
LUX
MCO
MLT
NLD
NOR
NZL
PRT
SGP
SMR
SWE
URY
USA
proportion
month
0.00
0.05
0.10
0.15
0.20
5
10
15
20
January 2020 − September 2021 (Months 1−21)

Southeast Asia, East Asia, and Oceania: Disruption in medication access

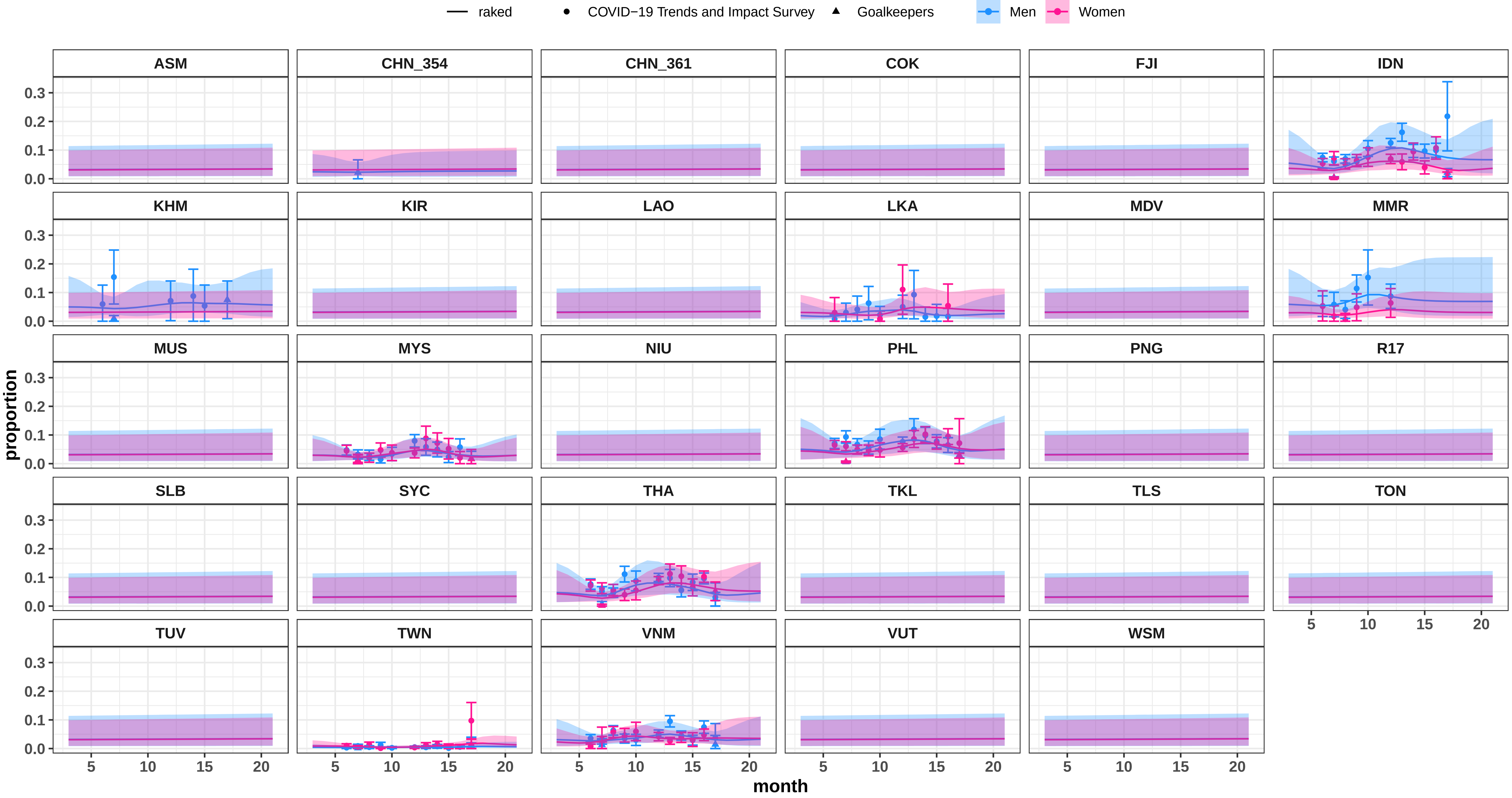

January 2020 − September 2021 (Months 1−21)

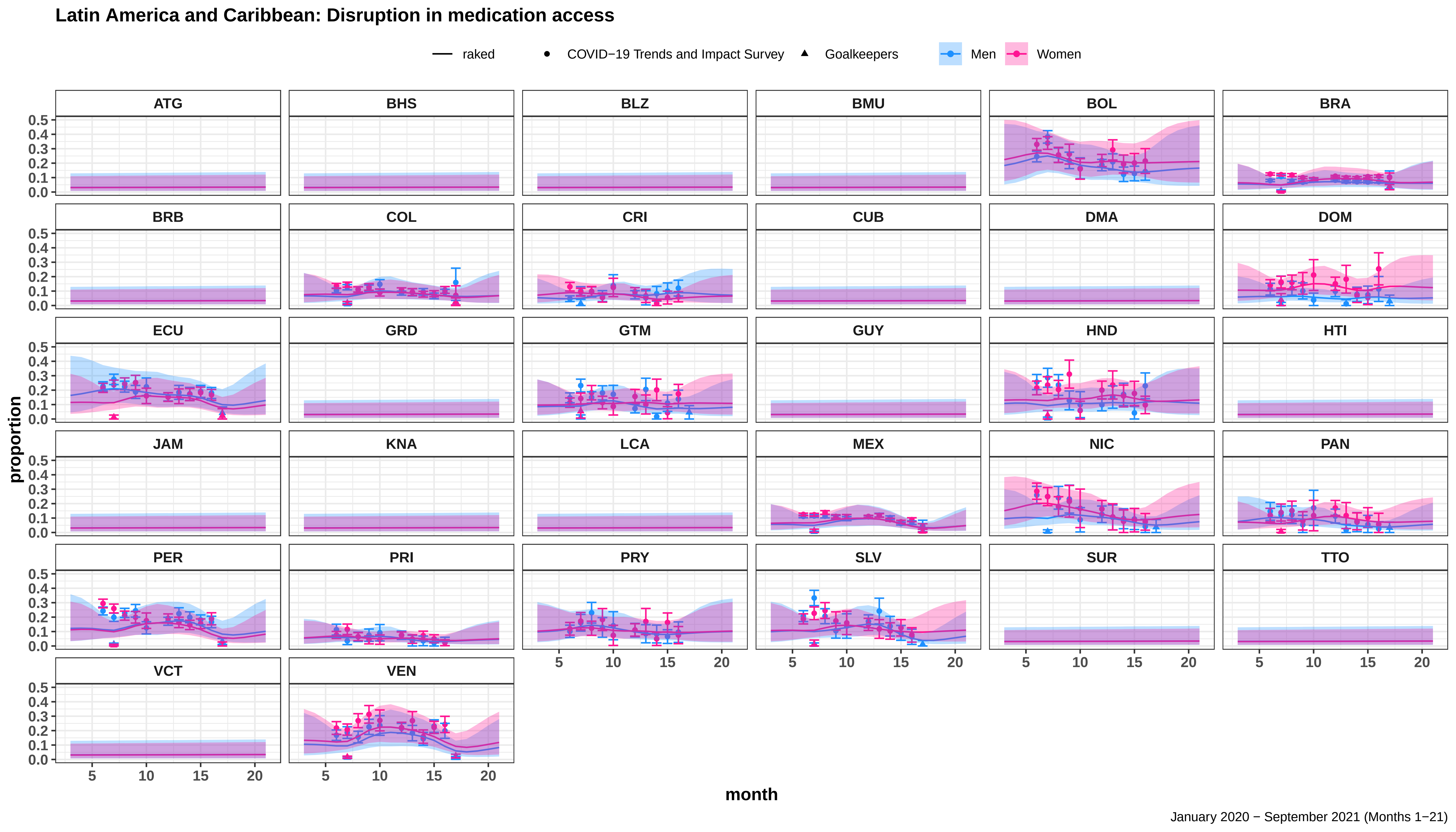
Latin America and Caribbean: Disruption in medication access
raked
COVID−19 Trends and Impact Survey
Goalkeepers
Men
Women
ATG
BHS
BLZ
BMU
BOL
BRA
BRB
COL
CRI
CUB
DMA
DOM
ECU
GRD
GTM
GUY
HND
HTI
JAM
KNA
LCA
MEX
NIC
PAN
PER
PRI
PRY
SLV
SUR
TTO
VCT
VEN
proportion
0.0
0.1
0.2
0.3
0.4
0.5
month
5
10
15
20
January 2020 − September 2021 (Months 1−21)

South Asia: Disruption in medication access

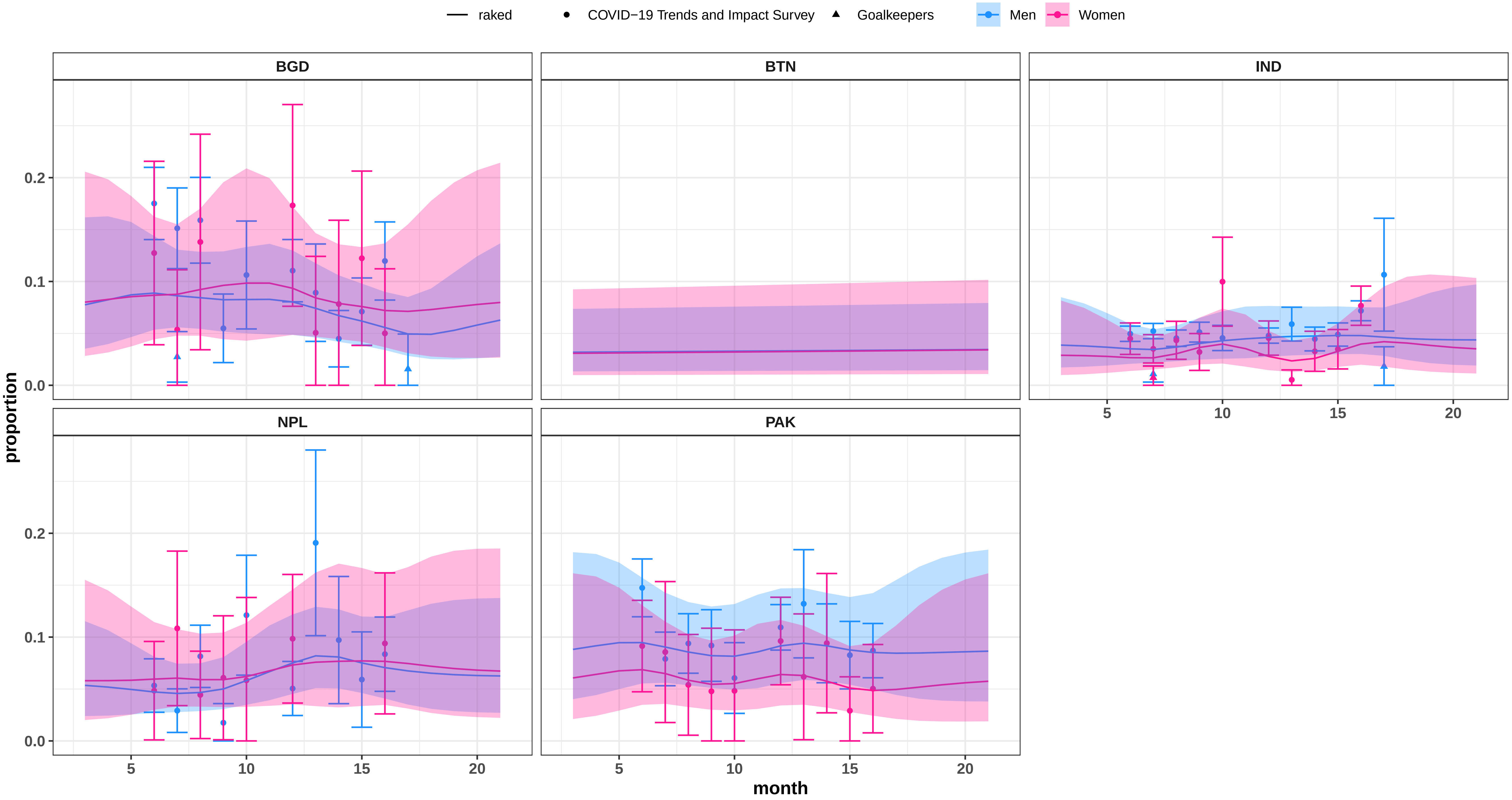

January 2020 − September 2021 (Months 1−21)

North Africa and Middle East: Disruption in health products access

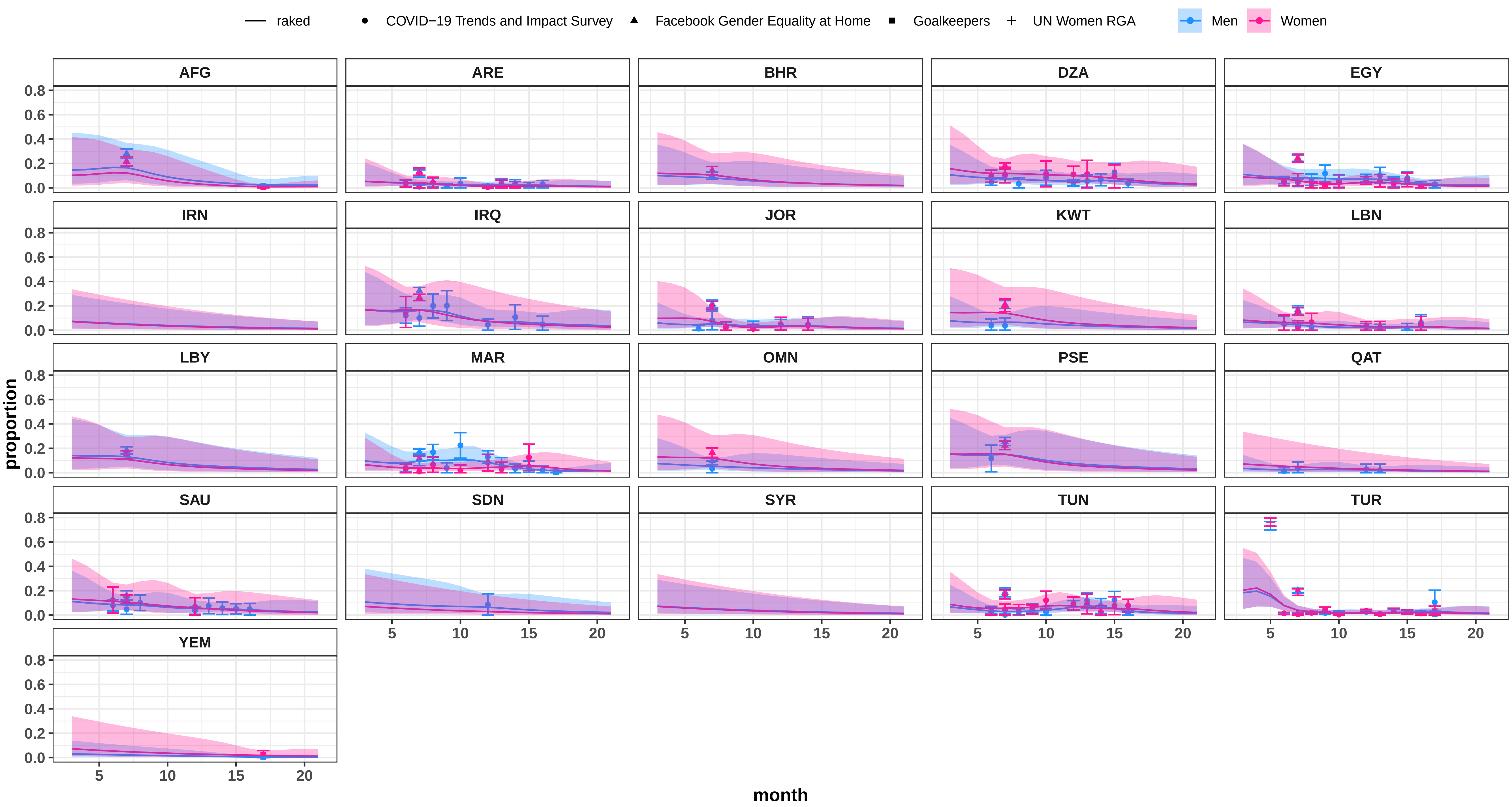

January 2020 − September 2021 (Months 1−21)

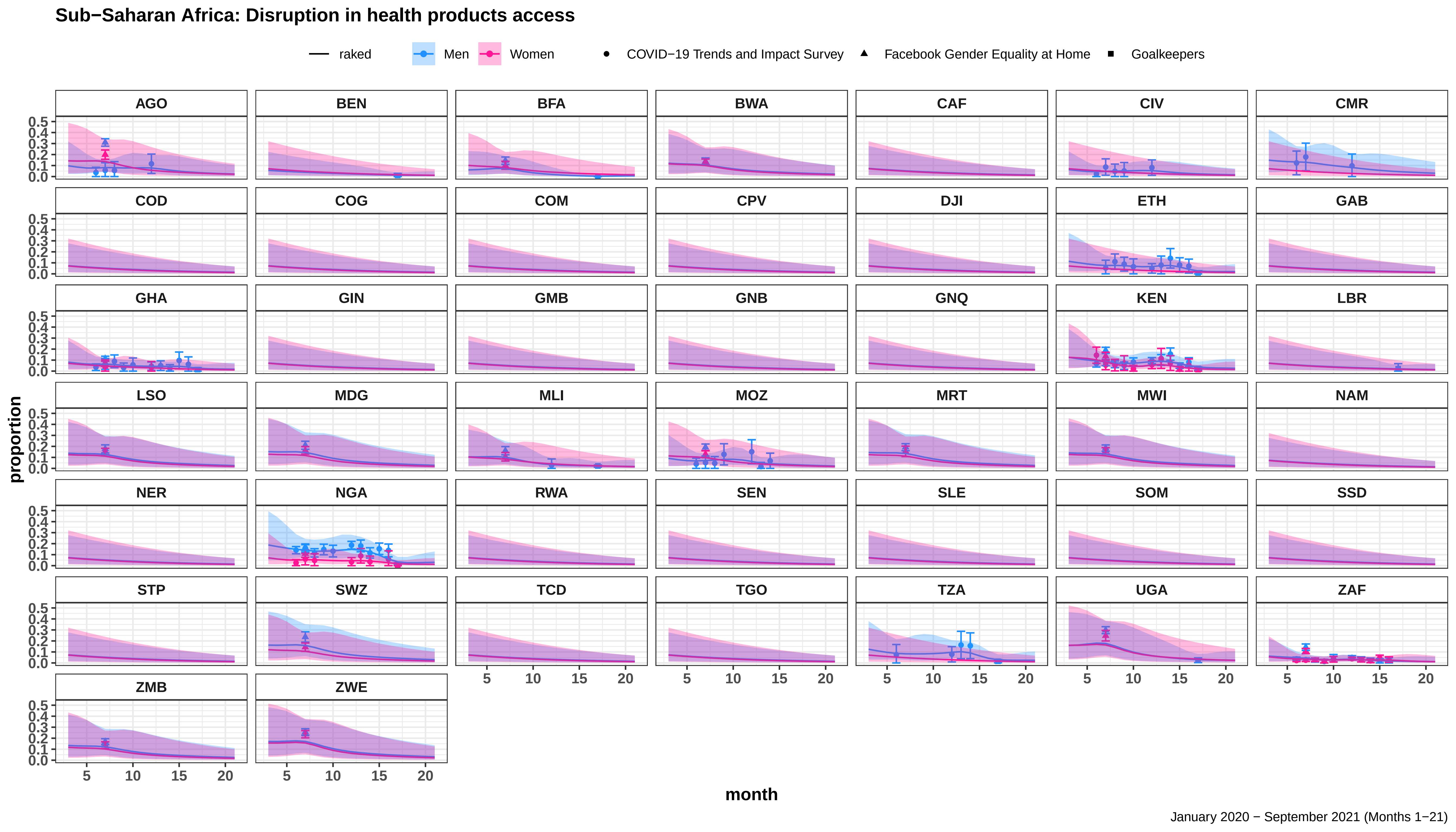

Sub−Saharan Africa: Disruption in health products access
raked
Men
Women
COVID−19 Trends and Impact Survey
Facebook Gender Equality at Home
Goalkeepers
AGO
BEN
BFA
BWA
CAF
CIV
CMR
COD
COG
COM
CPV
DJI
ETH
GAB
GHA
GIN
GMB
GNB
GNQ
KEN
LBR
LSO
MDG
MLI
MOZ
MRT
MWI
NAM
NER
NGA
RWA
SEN
SLE
SOM
SSD
STP
SWZ
TCD
TGO
TZA
UGA
ZAF
ZMB
ZWE
proportion
0.0
0.1
0.2
0.3
0.4
0.5
5
10
15
20
month
January 2020 − September 2021 (Months 1−21)

Central Europe, Eastern Europe, and Central Asia: Disruption in health products access

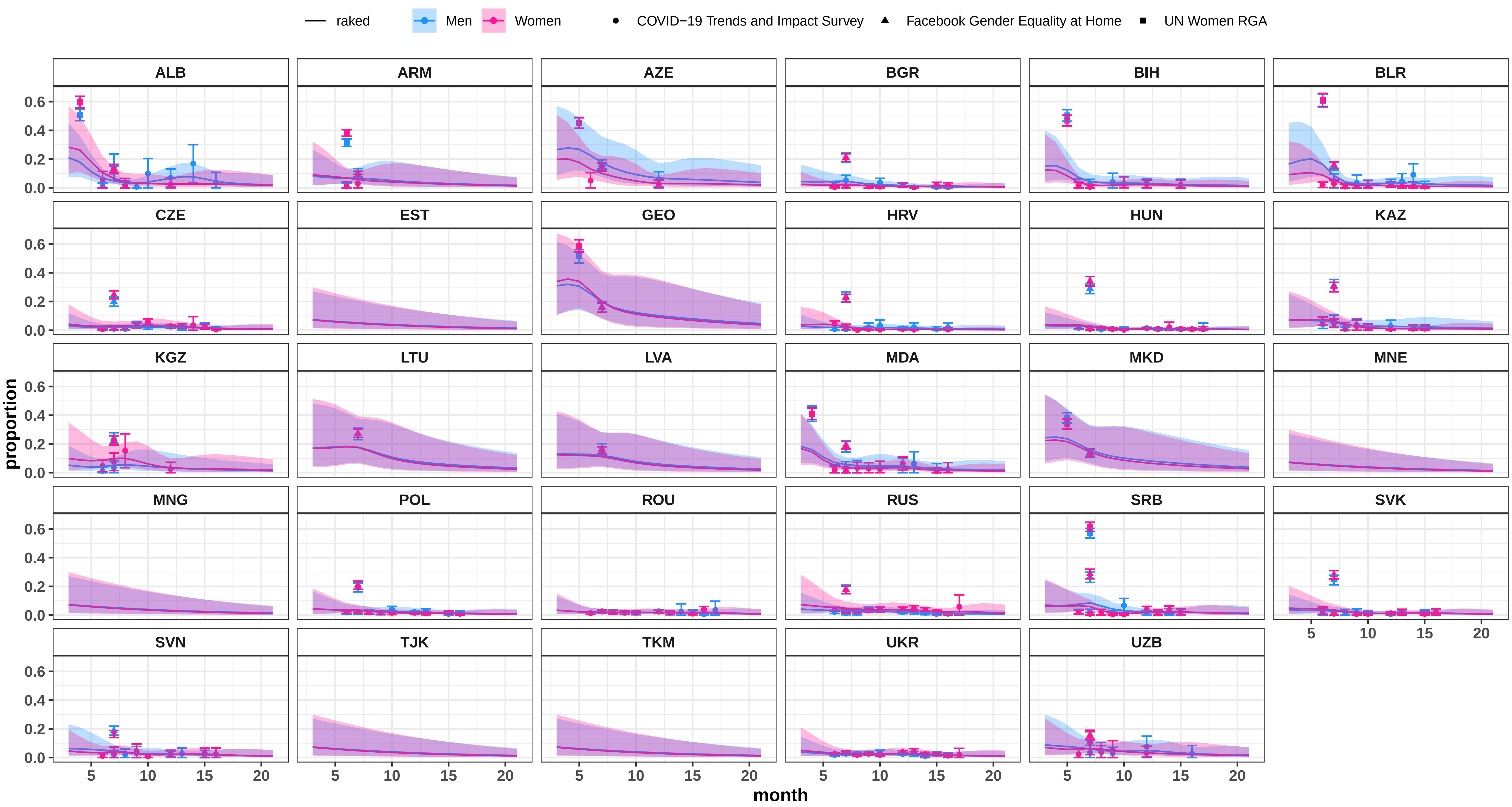

January 2020 − September 2021 (Months 1−21)

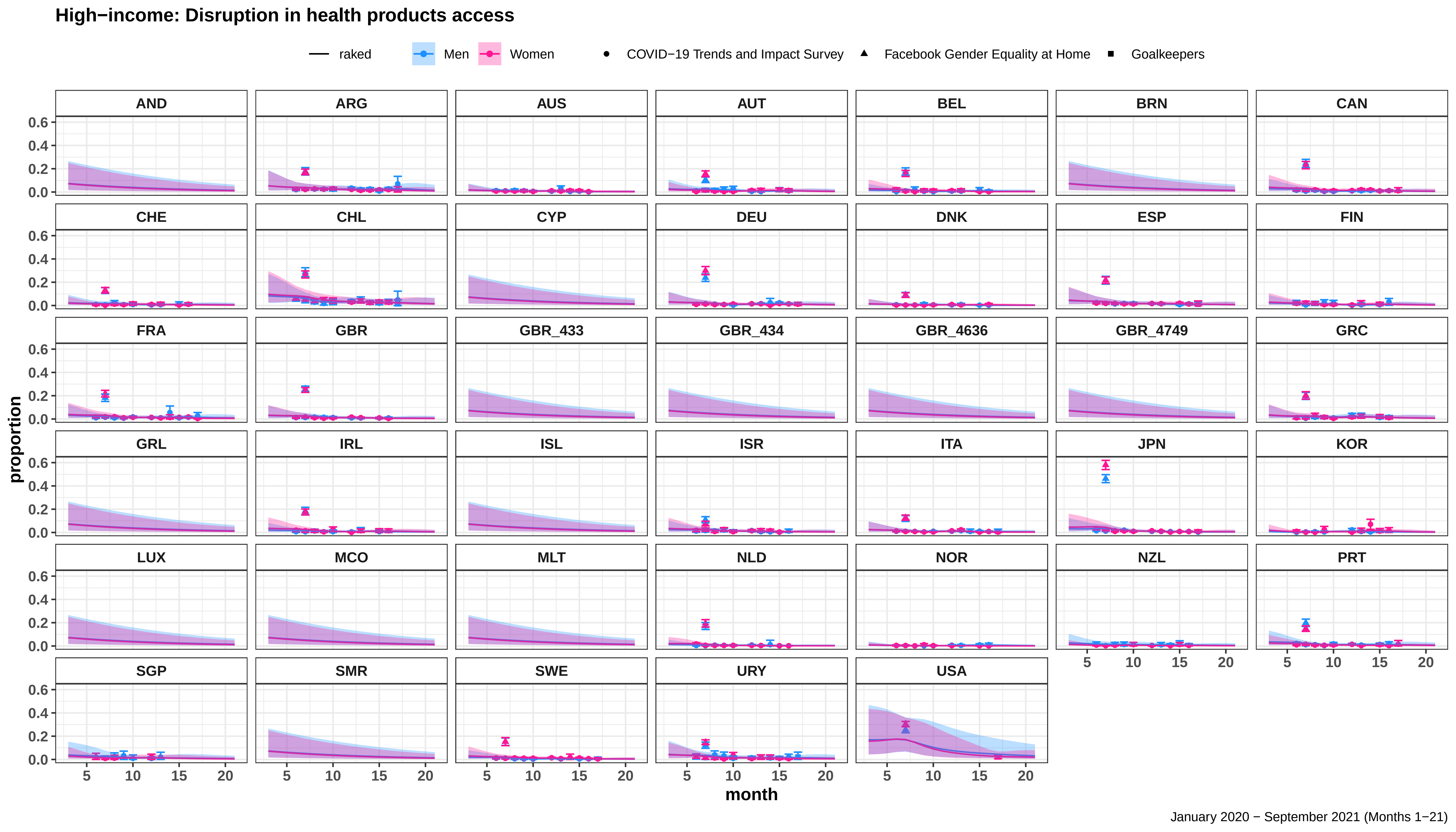
High−income: Disruption in health products access
raked
Men
Women
COVID−19 Trends and Impact Survey
Facebook Gender Equality at Home
Goalkeepers
AND
ARG
AUS
AUT
BEL
BRN
CAN
CHE
CHL
CYP
DEU
DNK
ESP
FIN
FRA
GBR
GBR_433
GBR_434
GBR_4636
GBR_4749
GRC
GRL
IRL
ISL
ISR
ITA
JPN
KOR
LUX
MCO
MLT
NLD
NOR
NZL
PRT
SGP
SMR
SWE
URY
USA
proportion
month
January 2020 − September 2021 (Months 1−21)

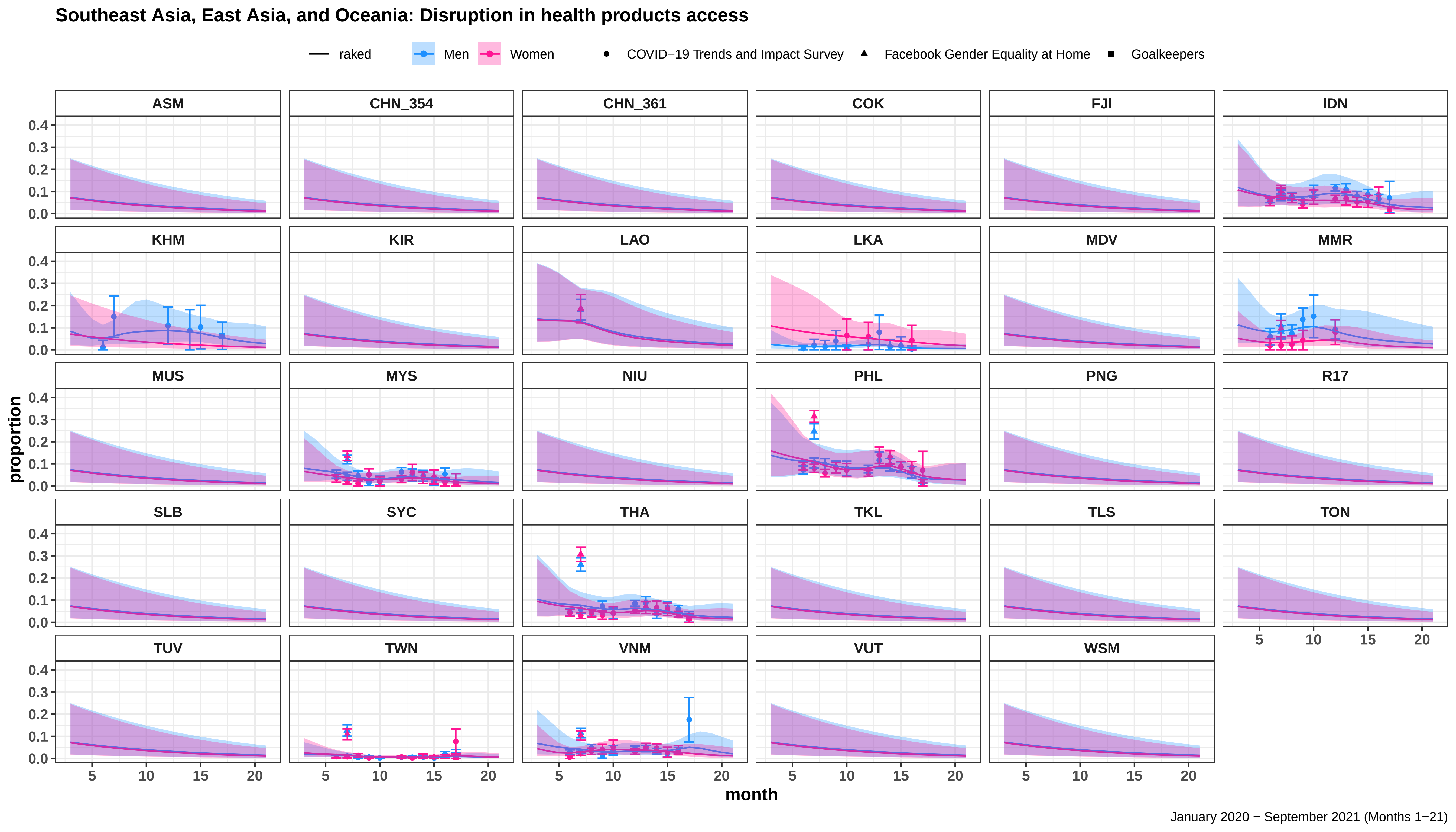

Southeast Asia, East Asia, and Oceania: Disruption in health products access
raked
Men
Women
COVID−19 Trends and Impact Survey
Facebook Gender Equality at Home
Goalkeepers
ASM
CHN_354
CHN_361
COK
FJI
IDN
KHM
KIR
LAO
LKA
MDV
MMR
MUS
MYS
NIU
PHL
PNG
R17
SLB
SYC
THA
TKL
TLS
TON
TUV
TWN
VNM
VUT
WSM
proportion
month
0.0
0.1
0.2
0.3
0.4
5
10
15
20
January 2020 − September 2021 (Months 1−21)

Latin America and Caribbean: Disruption in health products access

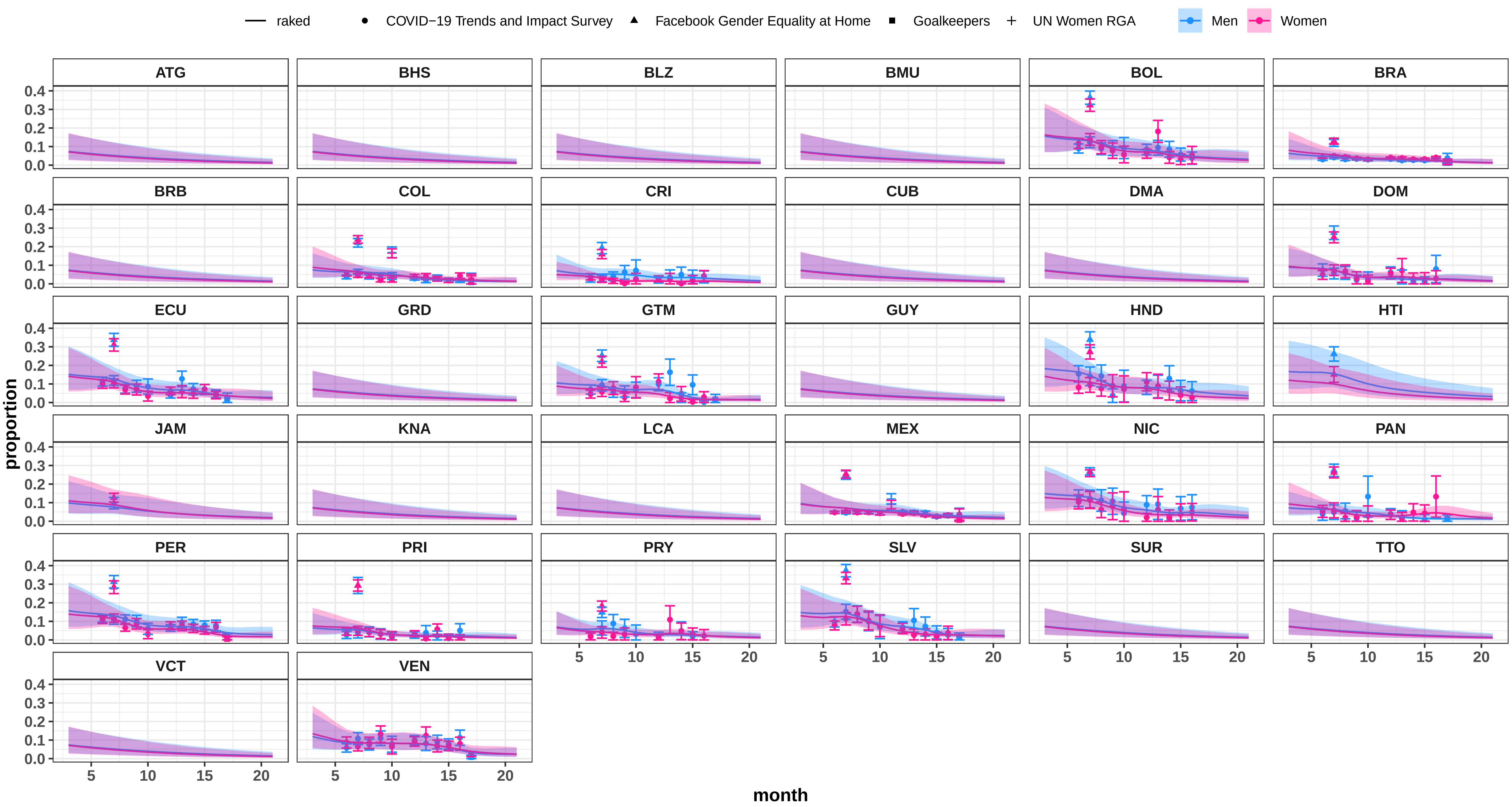

January 2020 − September 2021 (Months 1−21)

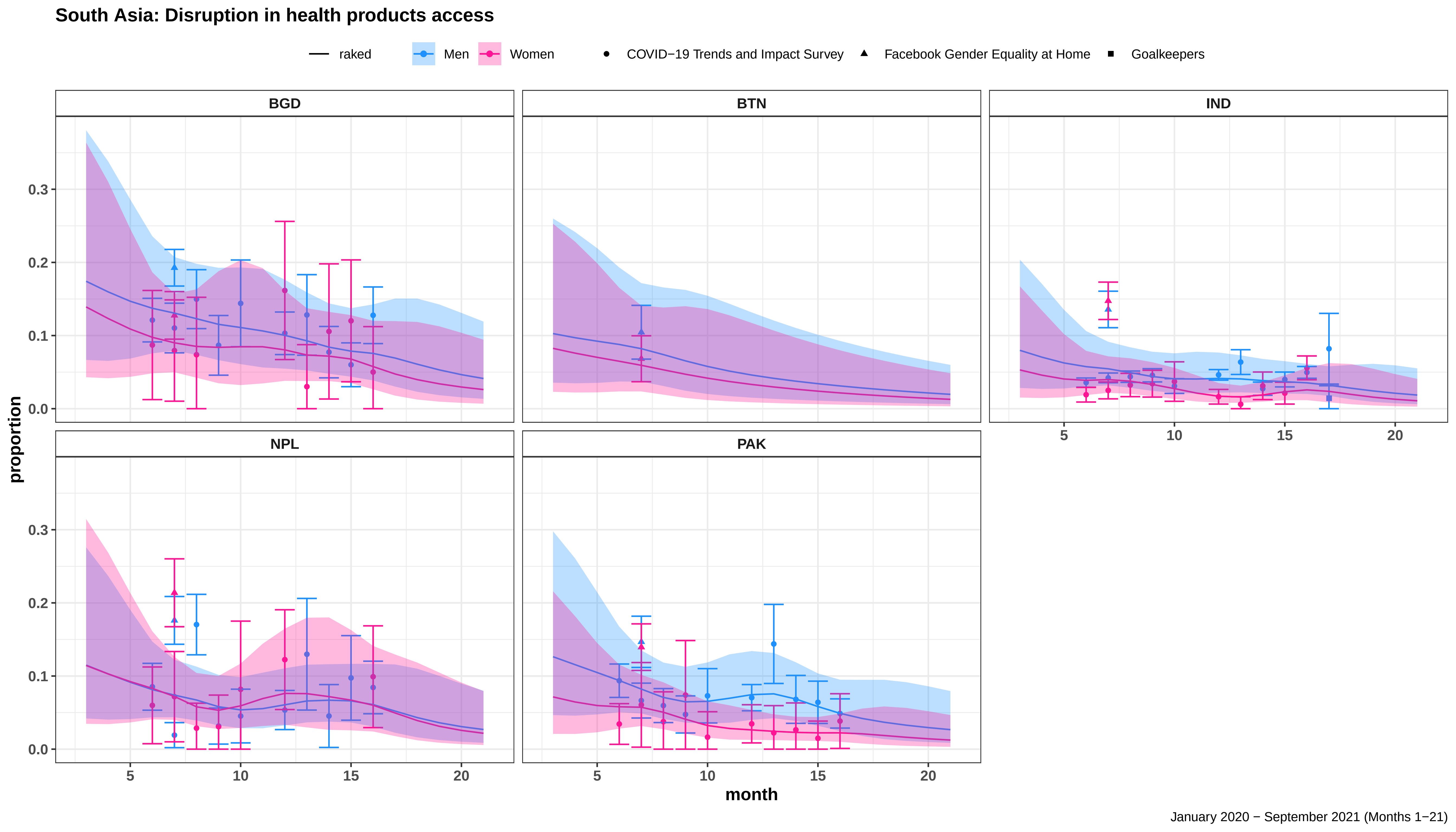
South Asia: Disruption in health products access
raked
Men
Women
COVID−19 Trends and Impact Survey
Facebook Gender Equality at Home
Goalkeepers
BGD
BTN
IND
NPL
PAK
proportion
0.0
0.1
0.2
0.3
month
5
10
15
20
January 2020 − September 2021 (Months 1−21)

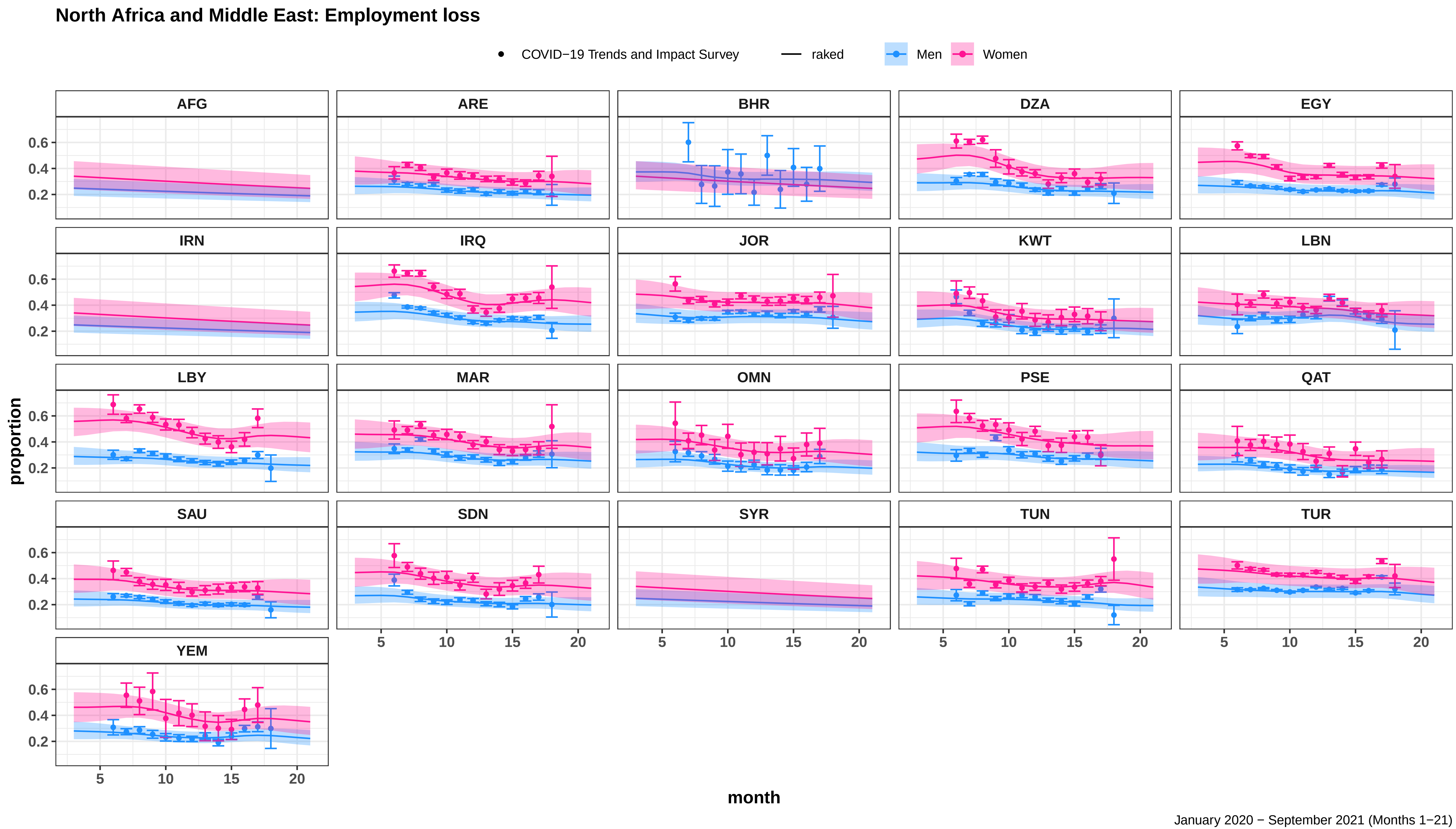
North Africa and Middle East: Employment loss
COVID−19 Trends and Impact Survey
raked
Men
Women
AFG
ARE
BHR
DZA
EGY
IRN
IRQ
JOR
KWT
LBN
LBY
MAR
OMN
PSE
QAT
SAU
SDN
SYR
TUN
TUR
YEM
proportion
month
January 2020 − September 2021 (Months 1−21)

Sub−Saharan Africa: Employment loss

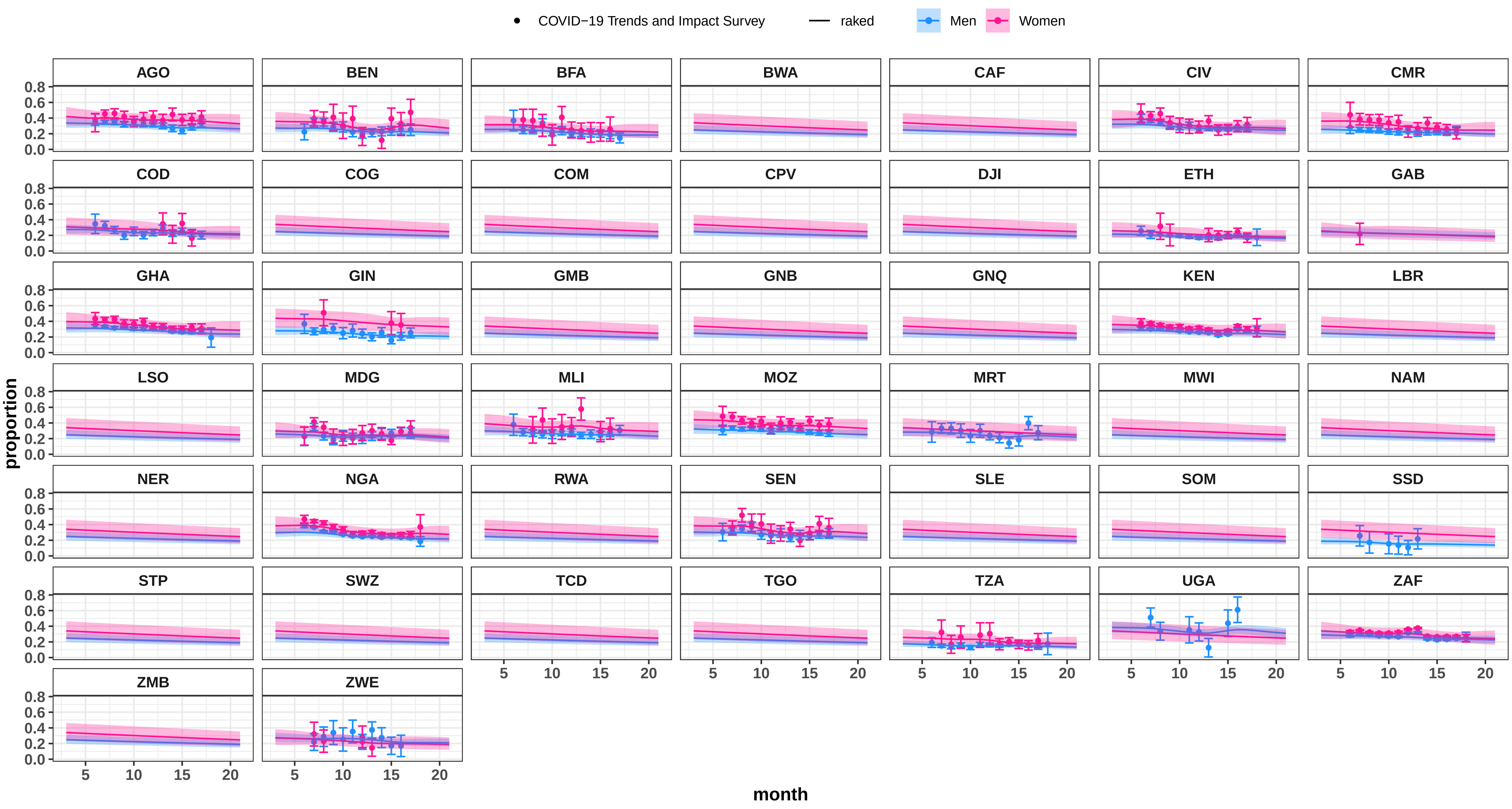

January 2020 − September 2021 (Months 1−21)

Central Europe, Eastern Europe, and Central Asia: Employment loss

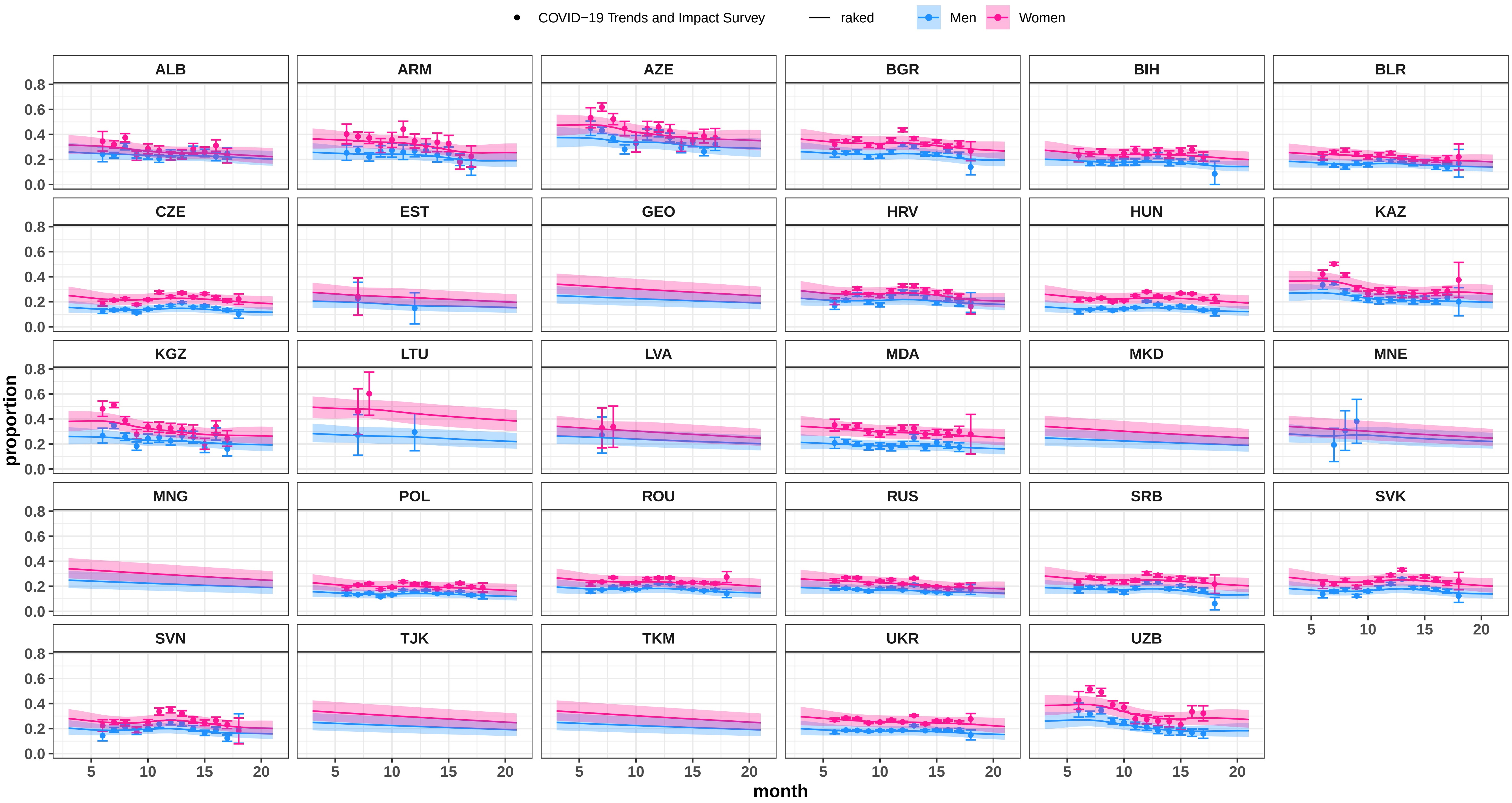

January 2020 − September 2021 (Months 1−21)

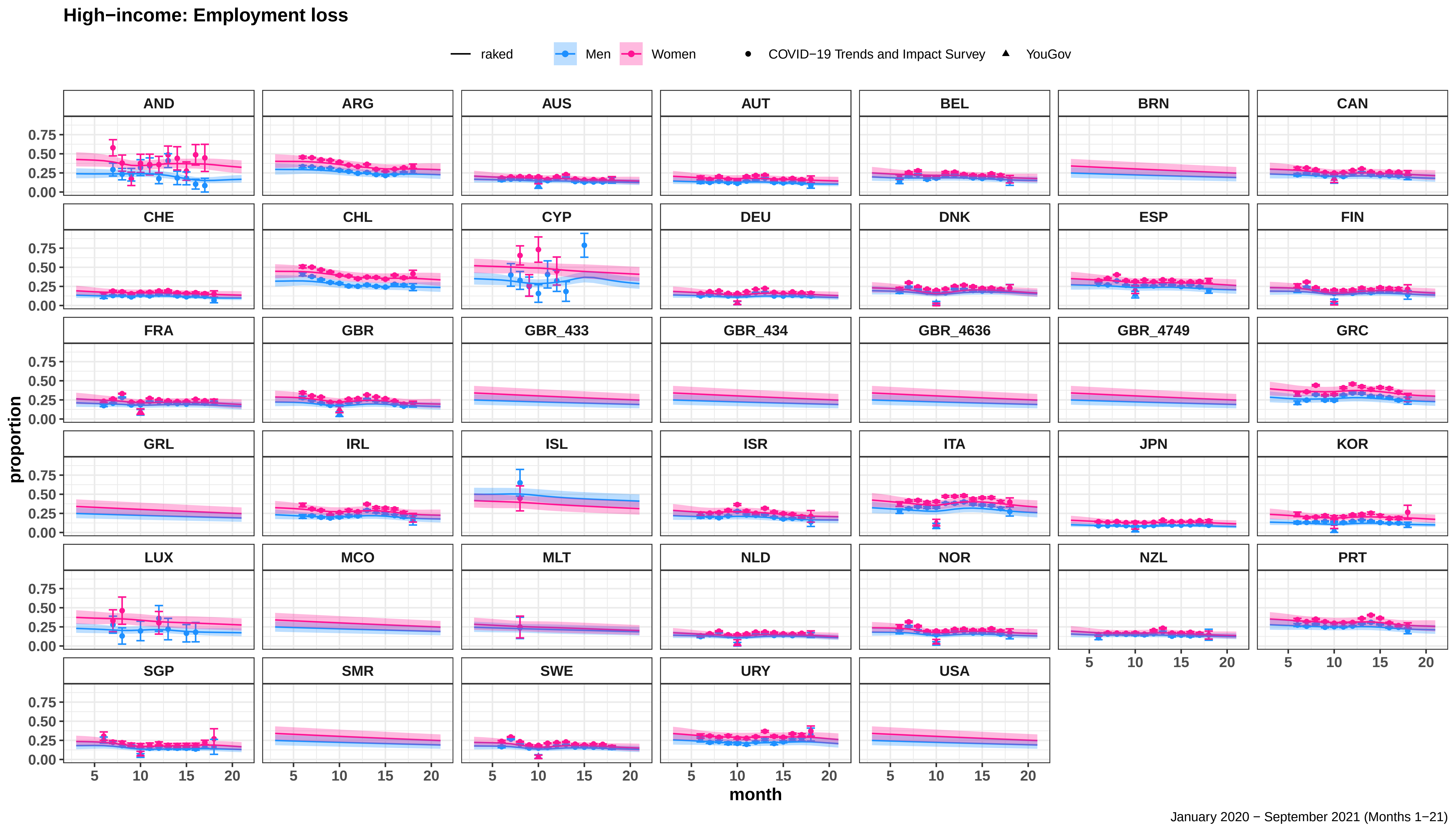
High−income: Employment loss
raked
Men
Women
COVID−19 Trends and Impact Survey
YouGov
AND
ARG
AUS
AUT
BEL
BRN
CAN
CHE
CHL
CYP
DEU
DNK
ESP
FIN
FRA
GBR
GBR_433
GBR_434
GBR_4636
GBR_4749
GRC
GRL
IRL
ISL
ISR
ITA
JPN
KOR
LUX
MCO
MLT
NLD
NOR
NZL
PRT
SGP
SMR
SWE
URY
USA
proportion
0.00
0.25
0.50
0.75
month
5
10
15
20
January 2020 − September 2021 (Months 1−21)

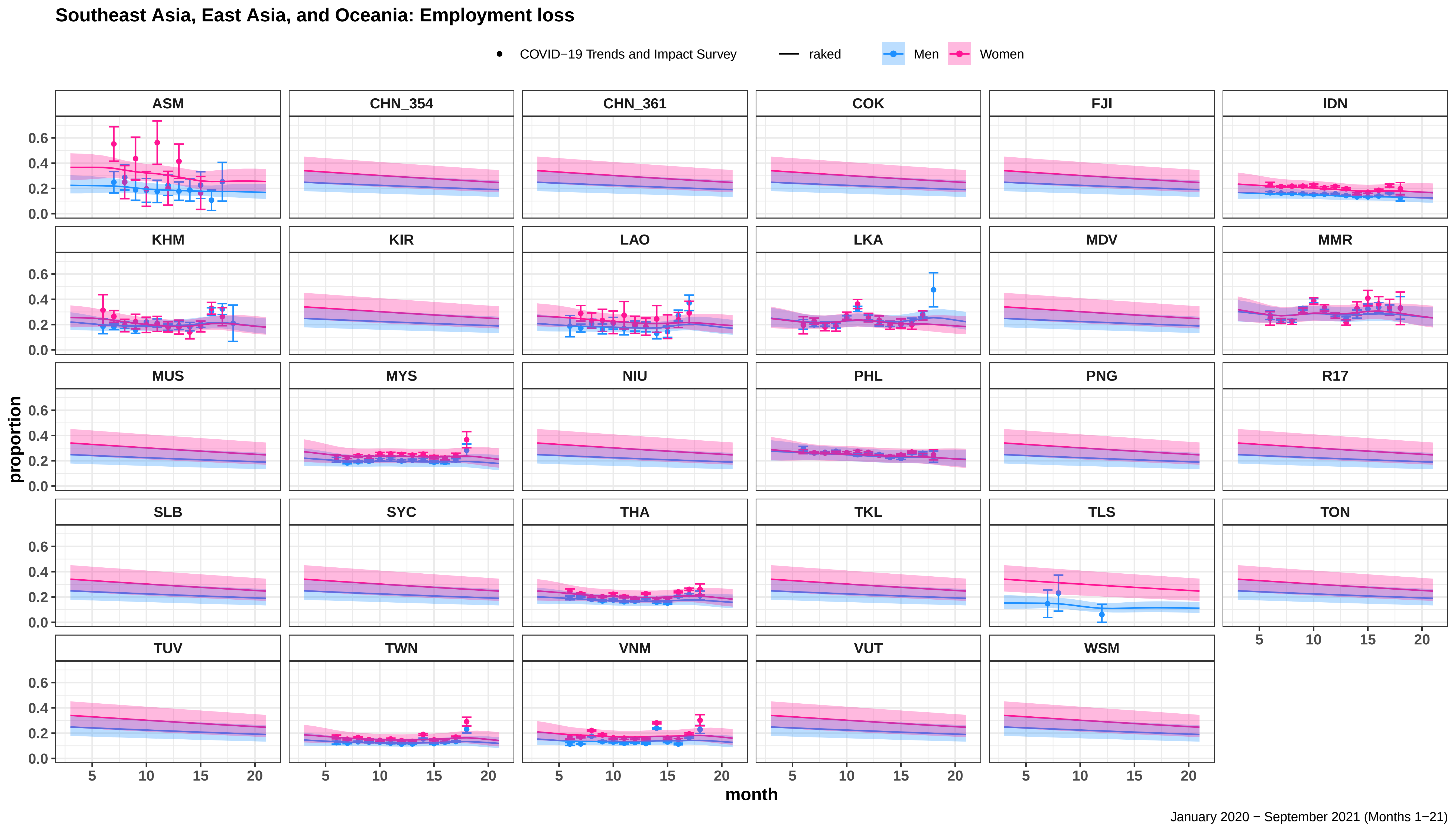

Southeast Asia, East Asia, and Oceania: Employment loss
COVID−19 Trends and Impact Survey
raked
Men
Women
ASM
CHN_354
CHN_361
COK
FJI
IDN
KHM
KIR
LAO
LKA
MDV
MMR
MUS
MYS
NIU
PHL
PNG
R17
SLB
SYC
THA
TKL
TLS
TON
TUV
TWN
VNM
VUT
WSM
proportion
0.0
0.2
0.4
0.6
month
5
10
15
20
January 2020 − September 2021 (Months 1−21)

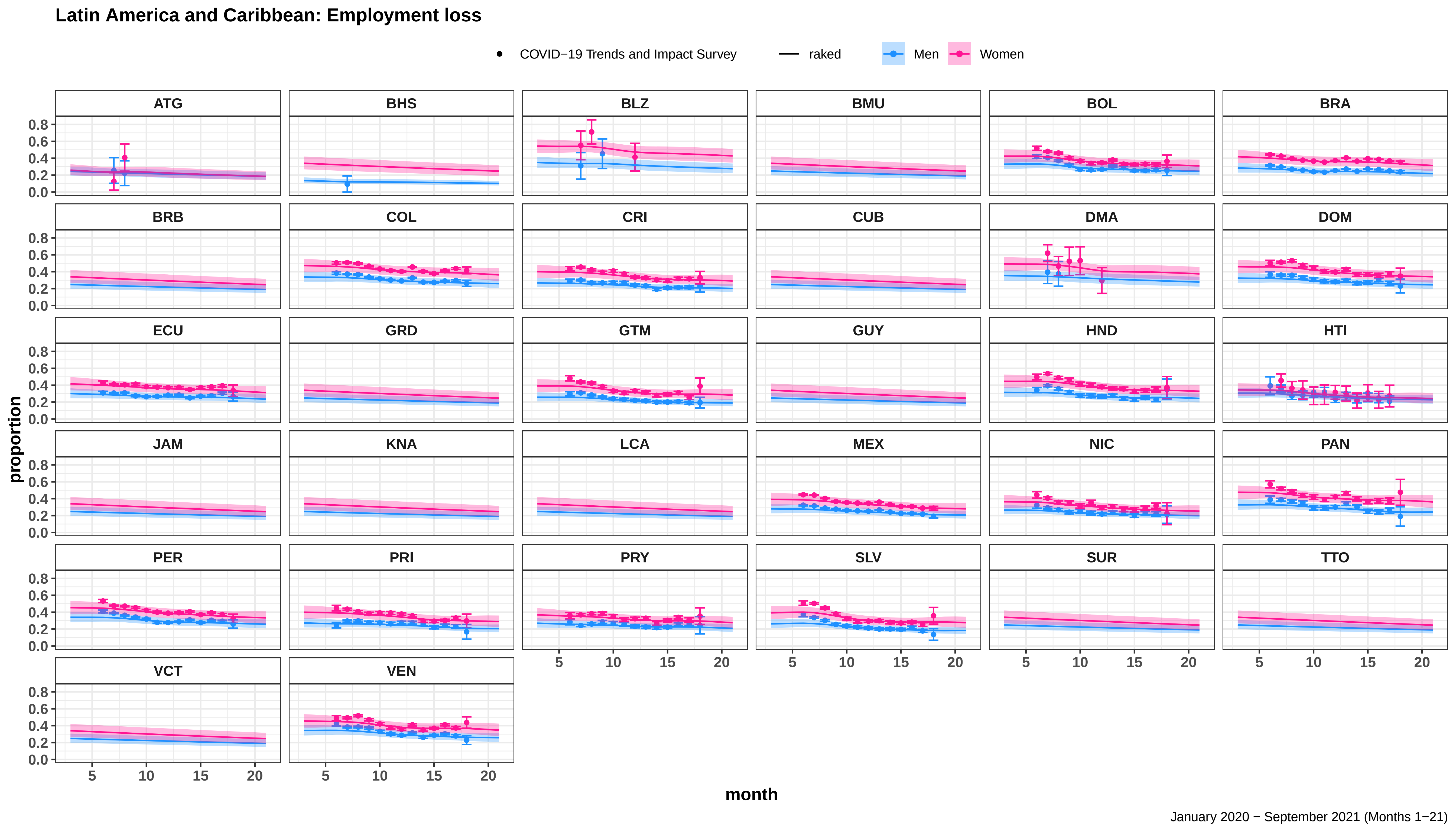
Latin America and Caribbean: Employment loss
COVID−19 Trends and Impact Survey
raked
Men
Women
ATG
BHS
BLZ
BMU
BOL
BRA
BRB
COL
CRI
CUB
DMA
DOM
ECU
GRD
GTM
GUY
HND
HTI
JAM
KNA
LCA
MEX
NIC
PAN
PER
PRI
PRY
SLV
SUR
TTO
VCT
VEN
0.0
0.2
0.4
0.6
0.8
proportion
5
10
15
20
month
January 2020 − September 2021 (Months 1−21)

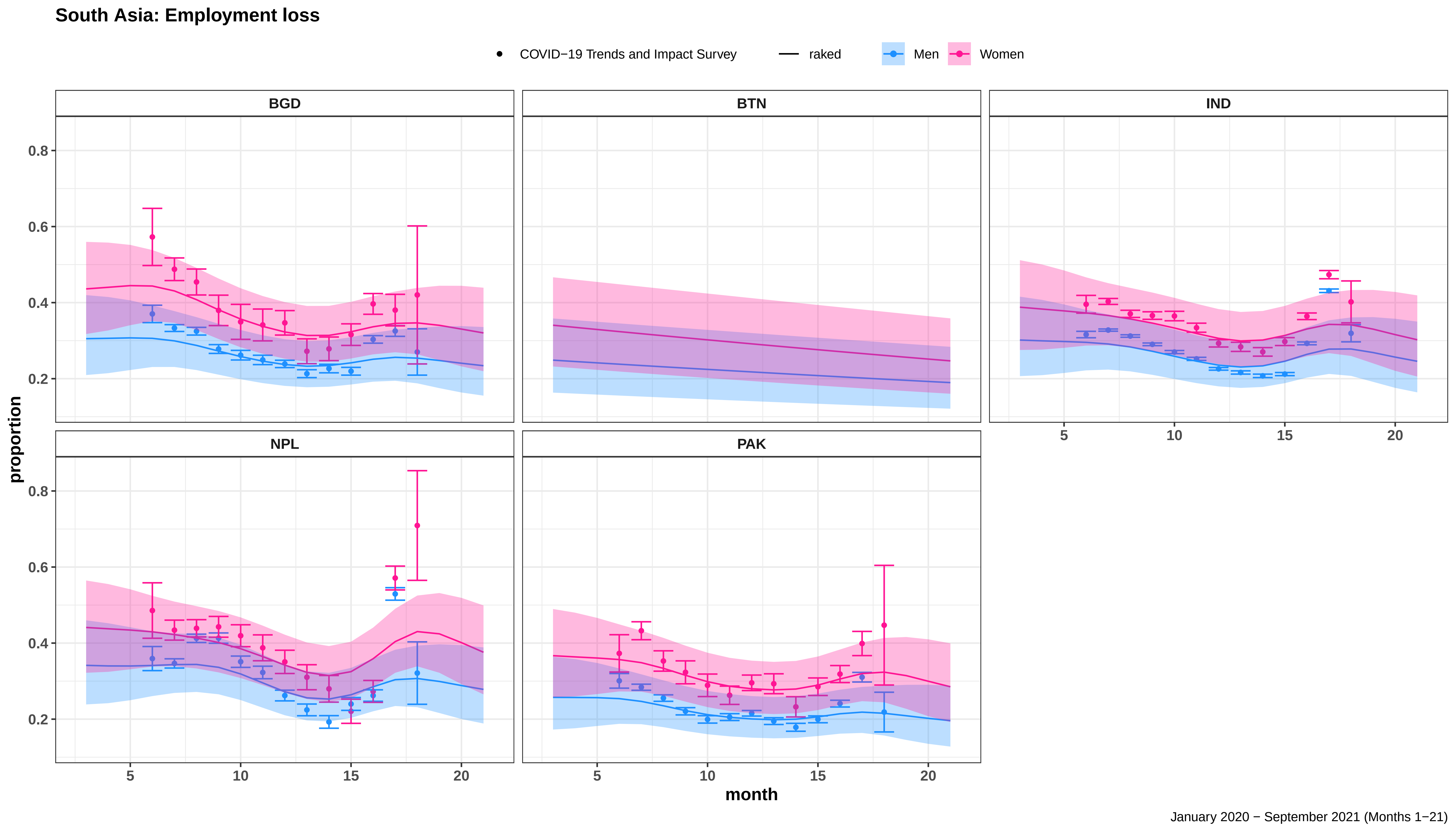
South Asia: Employment loss
COVID−19 Trends and Impact Survey
raked
Men
Women
BGD
BTN
IND
NPL
PAK
proportion
0.2
0.4
0.6
0.8
month
5
10
15
20
January 2020 − September 2021 (Months 1−21)

North Africa and Middle East: Not working to care for others

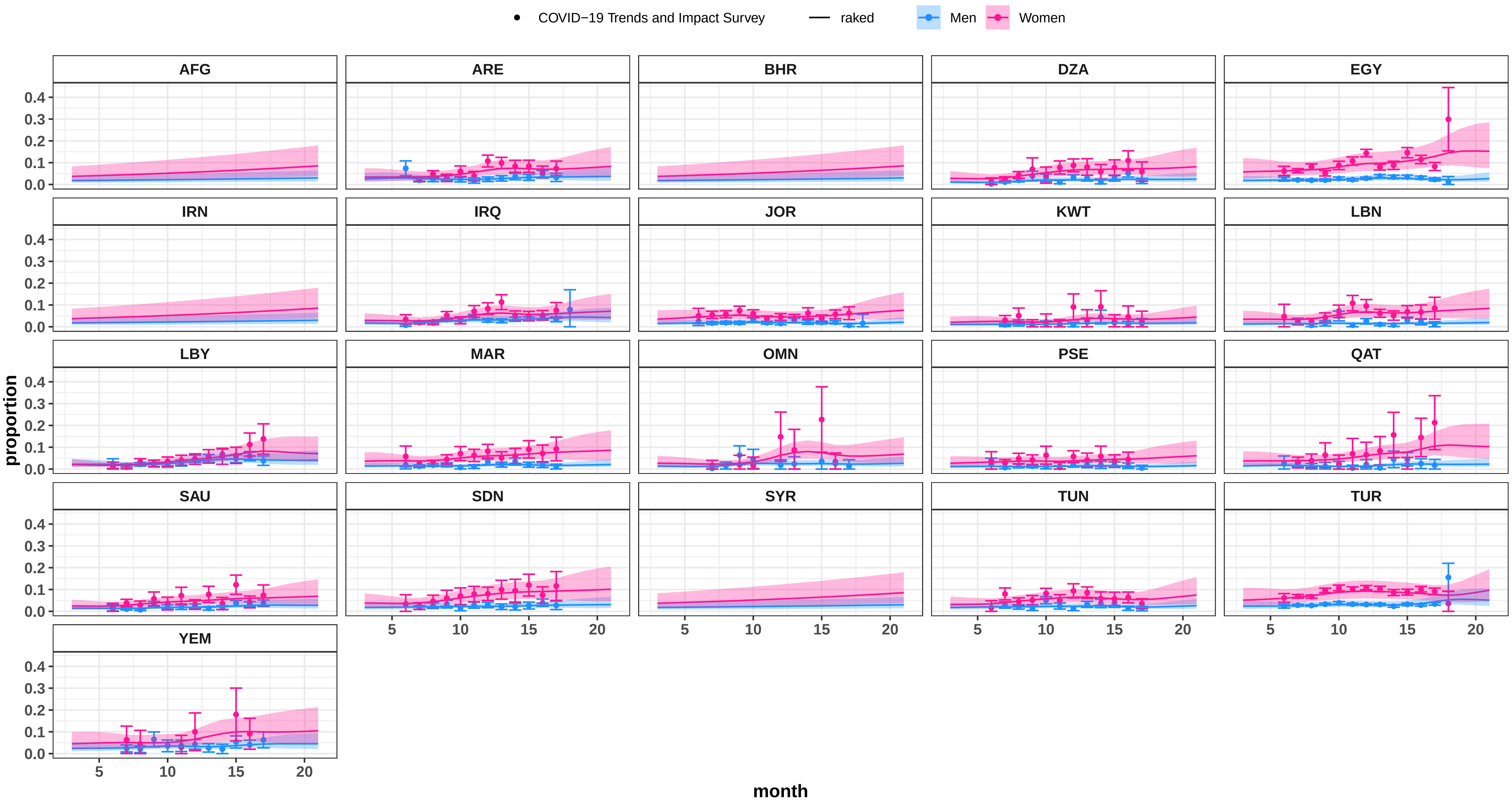

January 2020 − September 2021 (Months 1−21)

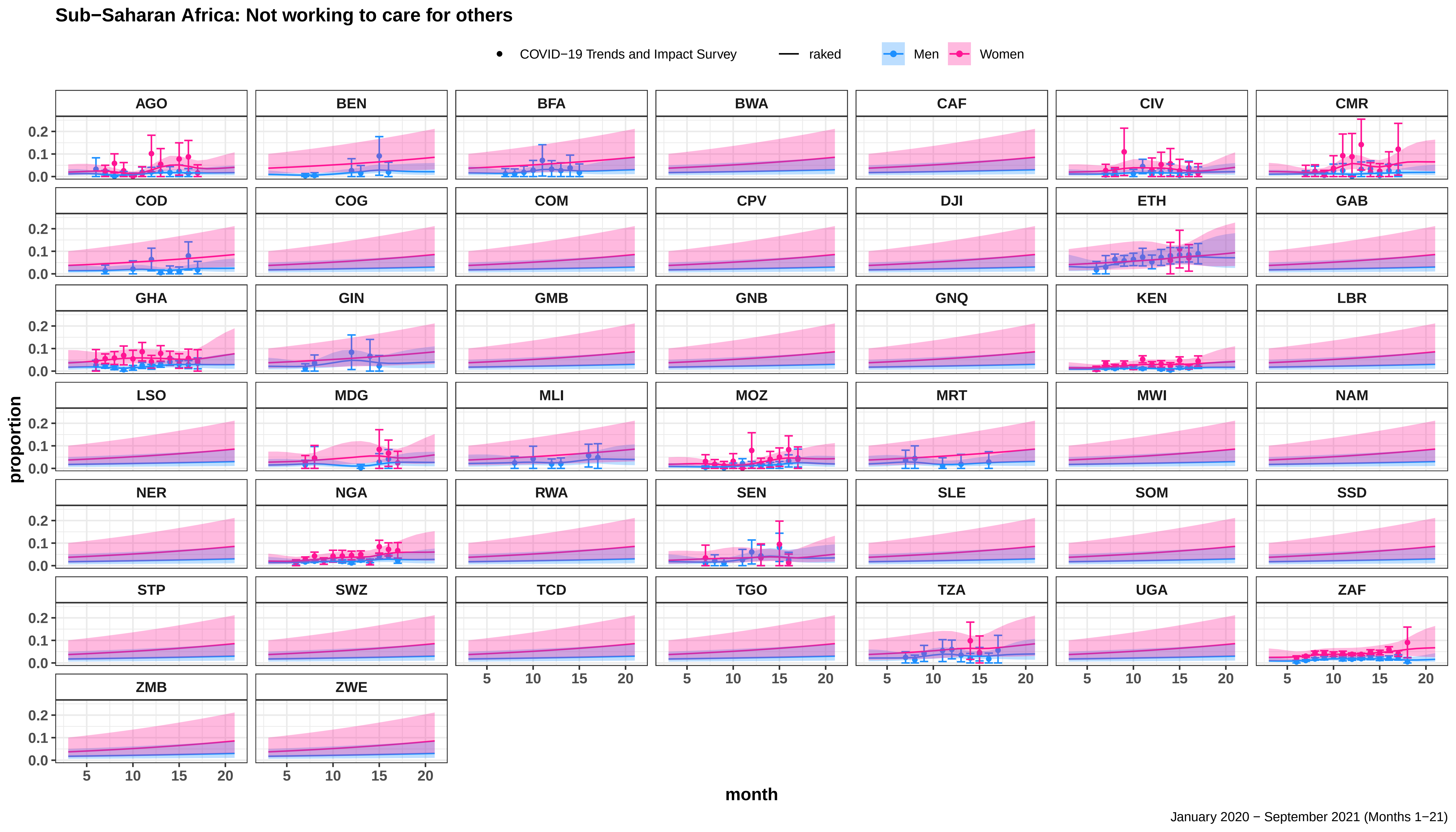

Sub−Saharan Africa: Not working to care for others
COVID−19 Trends and Impact Survey
raked
Men
Women
AGO
BEN
BFA
BWA
CAF
CIV
CMR
COD
COG
COM
CPV
DJI
ETH
GAB
GHA
GIN
GMB
GNB
GNQ
KEN
LBR
LSO
MDG
MLI
MOZ
MRT
MWI
NAM
NER
NGA
RWA
SEN
SLE
SOM
SSD
STP
SWZ
TCD
TGO
TZA
UGA
ZAF
ZMB
ZWE
proportion
0.0
0.1
0.2
month
5
10
15
20
January 2020 − September 2021 (Months 1−21)

Central Europe, Eastern Europe, and Central Asia: Not working to care for others

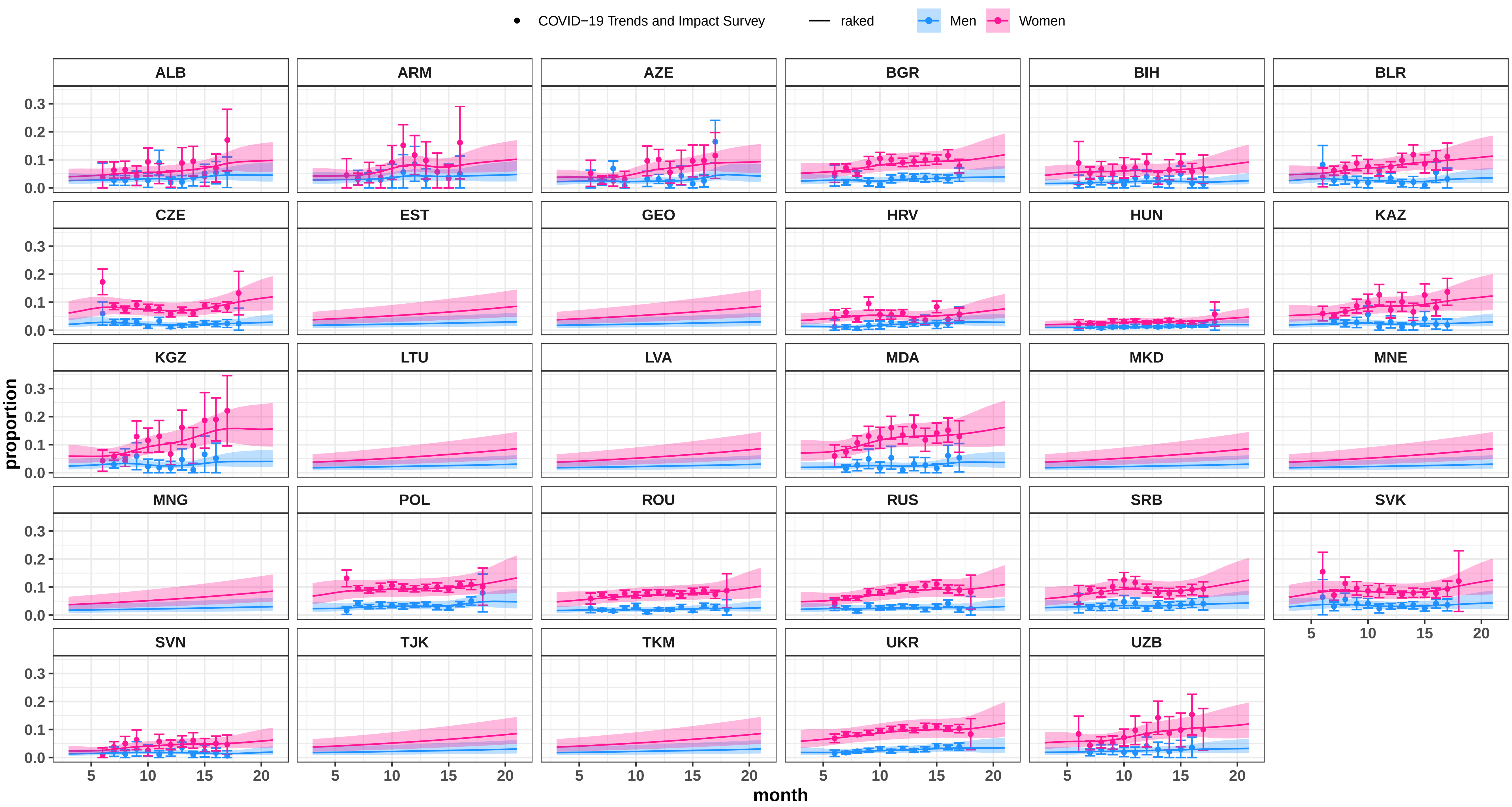

January 2020 − September 2021 (Months 1−21)

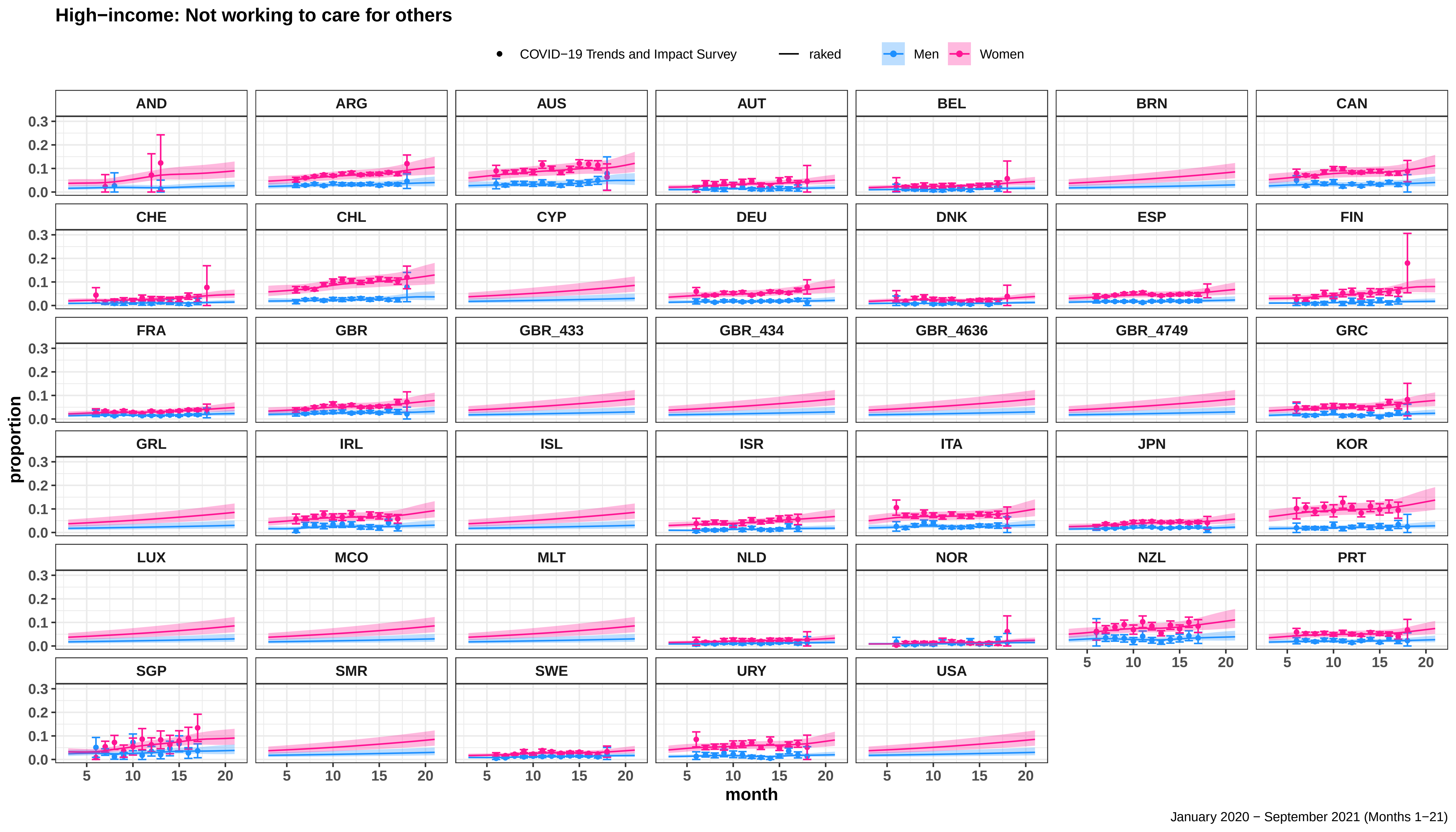

High−income: Not working to care for others
COVID−19 Trends and Impact Survey
raked
Men
Women
AND
ARG
AUS
AUT
BEL
BRN
CAN
CHE
CHL
CYP
DEU
DNK
ESP
FIN
FRA
GBR
GBR_433
GBR_434
GBR_4636
GBR_4749
GRC
GRL
IRL
ISL
ISR
ITA
JPN
KOR
LUX
MCO
MLT
NLD
NOR
NZL
PRT
SGP
SMR
SWE
URY
USA
proportion
0.0
0.1
0.2
0.3
month
5
10
15
20
January 2020 − September 2021 (Months 1−21)

Southeast Asia, East Asia, and Oceania: Not working to care for others

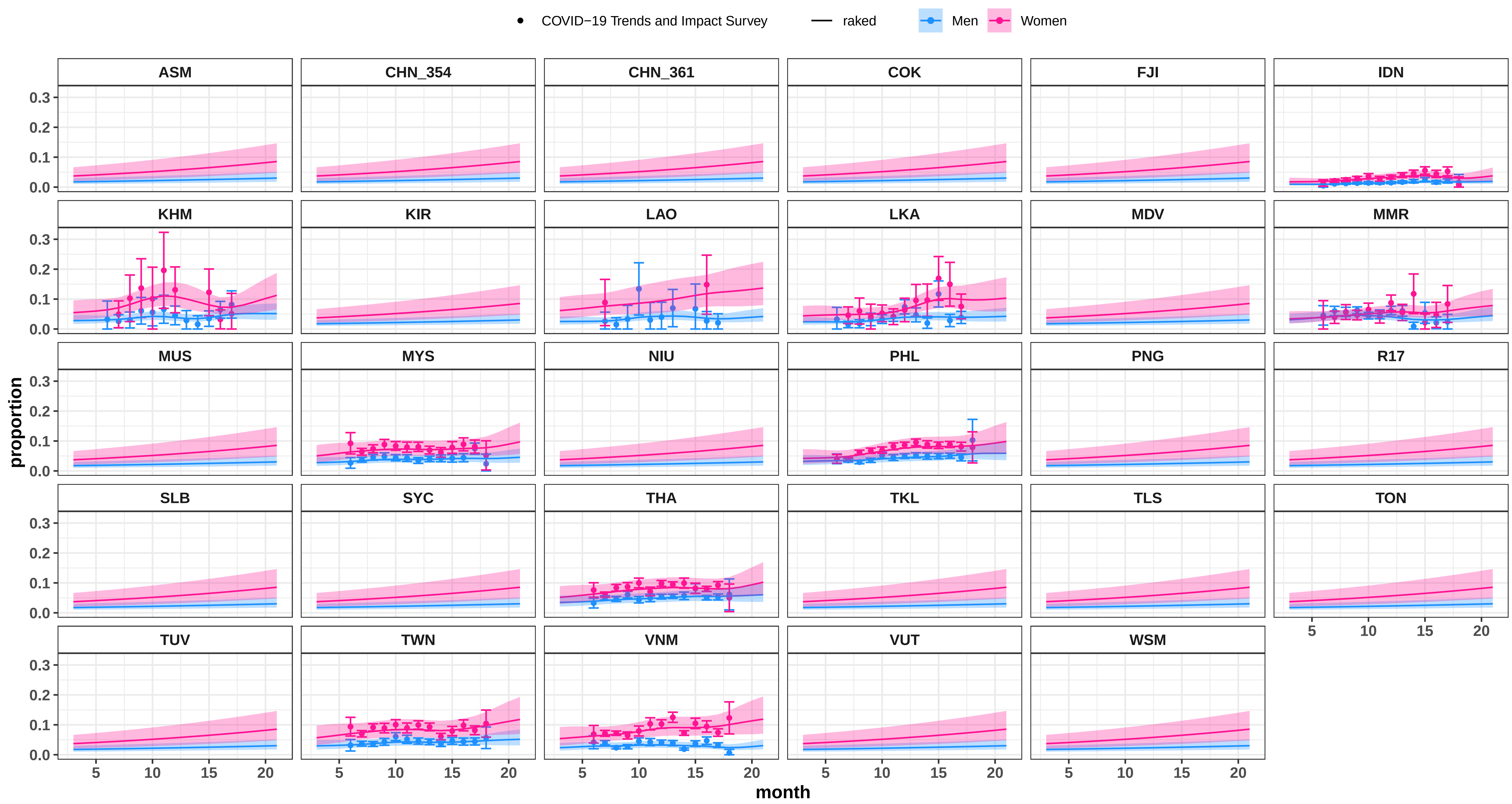

January 2020 − September 2021 (Months 1−21)

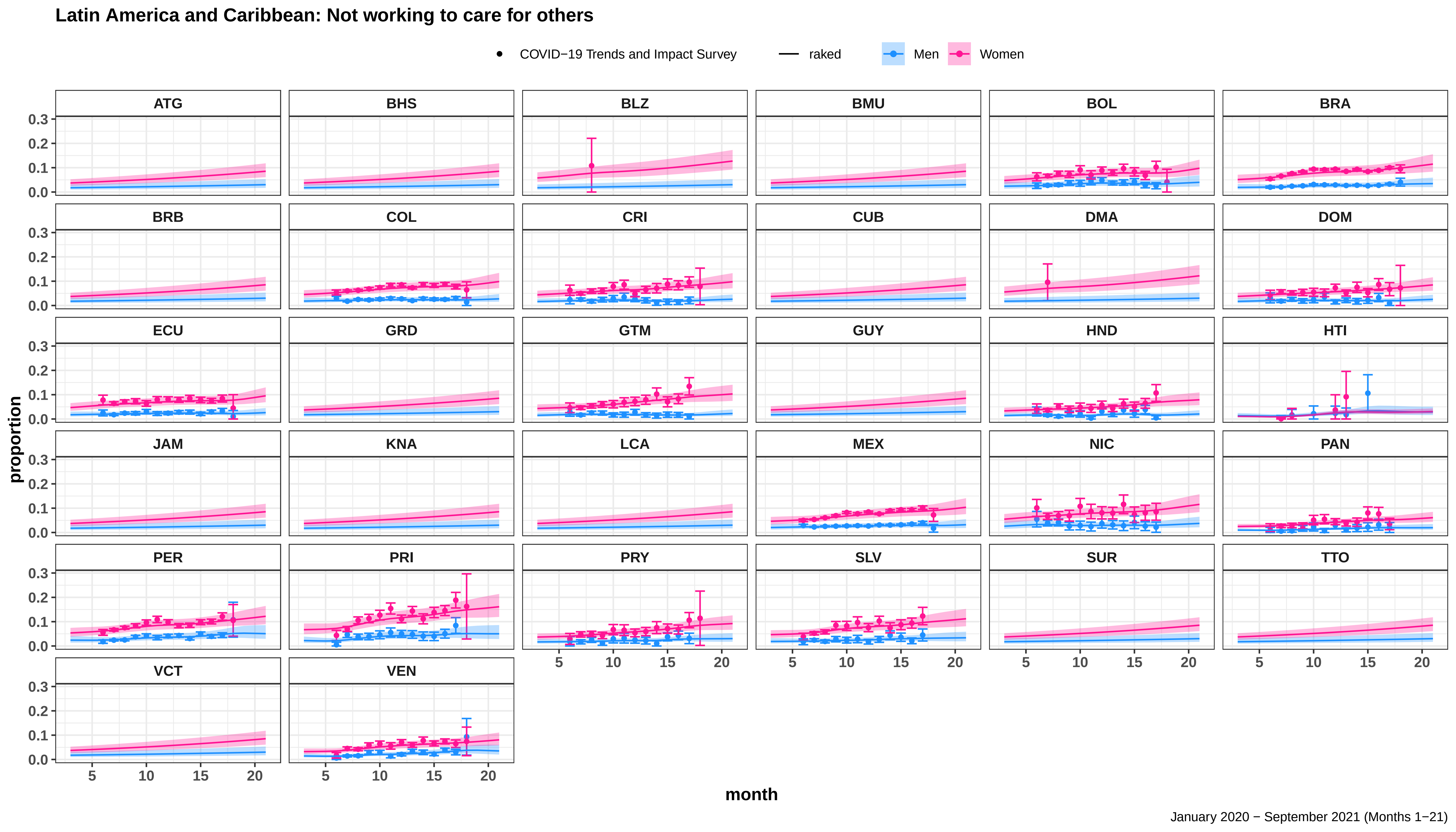
Latin America and Caribbean: Not working to care for others
COVID−19 Trends and Impact Survey
raked
Men
Women
ATG
BHS
BLZ
BMU
BOL
BRA
BRB
COL
CRI
CUB
DMA
DOM
ECU
GRD
GTM
GUY
HND
HTI
JAM
KNA
LCA
MEX
NIC
PAN
PER
PRI
PRY
SLV
SUR
TTO
VCT
VEN
proportion
0.0
0.1
0.2
0.3
month
5
10
15
20
January 2020 − September 2021 (Months 1−21)

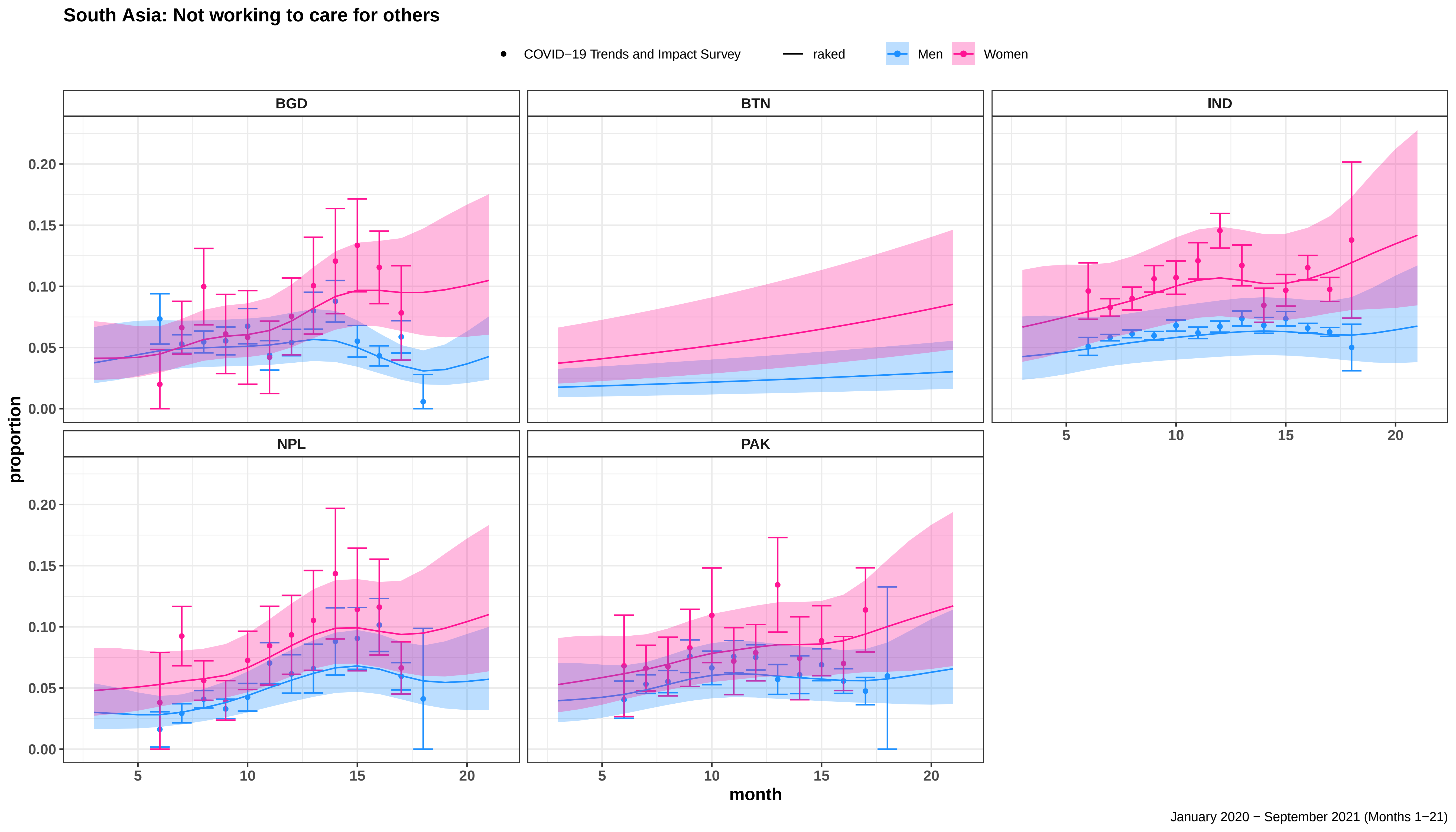
South Asia: Not working to care for others
COVID−19 Trends and Impact Survey
raked
Men
Women
BGD
BTN
IND
NPL
PAK
proportion
0.00
0.05
0.10
0.15
0.20
5
10
15
20
month
January 2020 − September 2021 (Months 1−21)
